# Highly Selective Electrosynthesis of 1*H*-1-Hydroxyquinol-4-ones–Synthetic Access to Versatile Natural Antibiotics

**DOI:** 10.1021/acs.oprd.4c00337

**Published:** 2024-09-24

**Authors:** Tobias Prenzel, Nils Schwarz, Jasmin Hammes, Franziska Krähe, Sarah Pschierer, Johannes Winter, María de Jesús Gálvez-Vázquez, Dieter Schollmeyer, Siegfried R. Waldvogel

**Affiliations:** † Department of Chemistry, Johannes Gutenberg University, Duesbergweg 10−14, 55128 Mainz, Germany; ‡ Max-Planck-Institute for Chemical Energy Conversion, Stiftstraße 34−36, 45470 Mülheim an der Ruhr, Germany; § Institute of Biological and Chemical Systems−Functional Molecular Systems (IBCS-FMS), Karlsruhe Institute of Technology (KIT), Kaiserstraße 12, 76131 Karlsruhe, Germany

**Keywords:** electrochemistry, electro-organic synthesis, N-heterocycles, quinolones, aurachin, reduction, cyclo-condensation

## Abstract

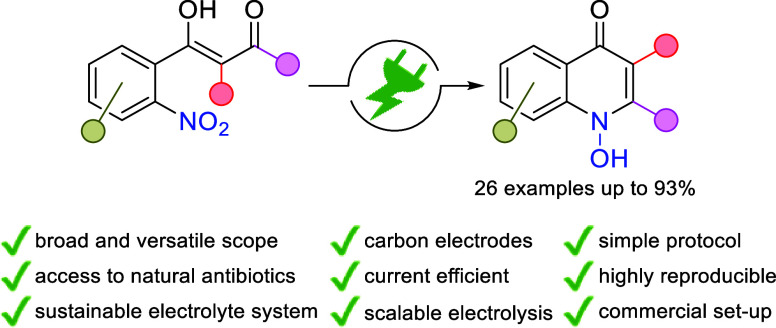

1*H*-1-Hydroxyquinolin-4-ones represent a broad class of biologically active heterocycles having an exocyclic N,O motif. Electrosynthesis offers direct, highly selective, and sustainable access to 1-hydroxyquinol-4-ones by nitro reduction. A versatile synthetic route starting from easily accessible 2-nitrobenzoic acids was established. The broad applicability of this protocol was demonstrated on 26 examples with up to 93% yield, highlighted by the naturally occurring antibiotics Aurachin C and HQNO. The practicability and technical relevance were underlined by multigram scale electrolysis.

## Introduction

**Chart 1:**
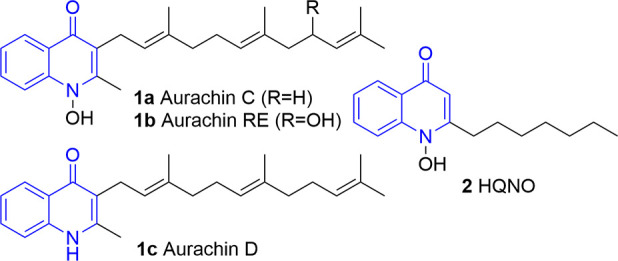
Important Biologically Active Compounds Involving Quinol-4-one Motif

Nitrogen containing heterocycles are ubiquitous in pharmaceuticals and biomolecules as core motifs.^1,2^ One particular example is the family of quinol-4-ones, which are represented in a variety of drugs and biomolecules with anticancer, diuretic, anti-inflammatory, anticonvulsant, and antihypertensive properties (Chart 1).^3−6^ In recent years, natural antibiotics such as the aurachins (**1a**–**c**), first isolated from myxobacteria like *stigmatella aurantiaca*, have taken the center stage in research.^7−12^ Due to the structural similarity of aurachins to vitamin K, they can inhibit the electron transport chains in living organisms and therefore also have a number of antimicrobial properties.^13,14^

**Scheme 1:**
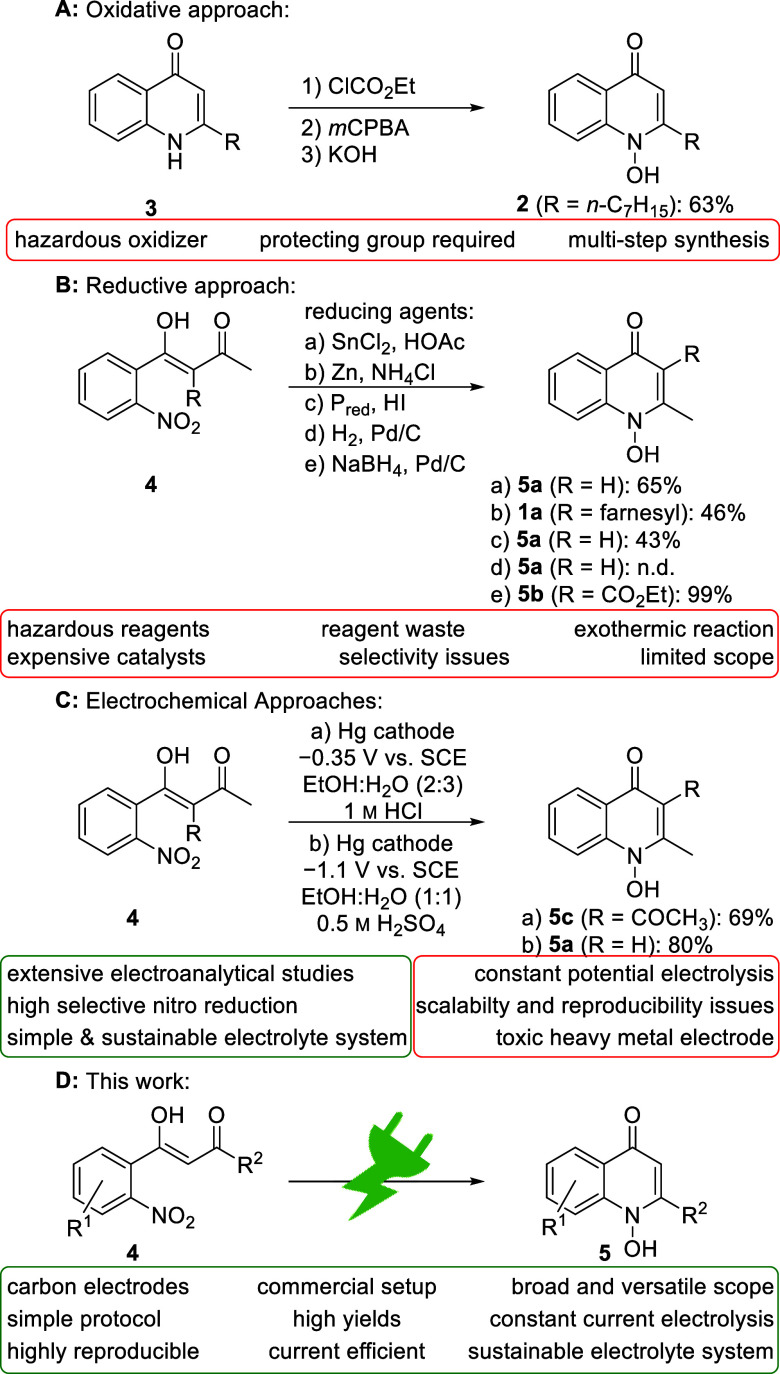
Synthetic Access to 1*H*-1-hydroxyquinol-4-ones

Functionalization and modification of aurachins by biosynthetic processes gained access to a large substance library in the field of quinolone antibiotics.^15−22^ Furthermore, 1*H*-2-heptyl-2-hydroxyquinol-4-one (**2**, HQNO) acts as an NADH oxidase inhibitor and shows antibiotic and antimicrobial properties, making it an ideal candidate for antimalaria and anticancer drugs or as caries prophylaxis.^23−30^ In addition to the biosynthetic approaches, a number of syntheses are reported that provide access to this unique structural motif of *N*-hydroxy quinolinones with its exocyclic N,O bond (Scheme 1).^20,31−35^ HQNO (**2**) is synthesized by oxidation of the corresponding quinolone using equimolar amounts of *m*CPBA (Scheme 1A).^36^ However, this method requires the use of a hazardous oxidant and protection with ethyl chloroformate, resulting in a poor atom economy.

In contrast, reduction of nitro arenes and subsequent cyclo-condensation enables direct access to the *N*-hydroxyquinolines (Scheme 1B).^37−42^ Selective reduction of the nitro group to the corresponding hydroxylamines is crucial. By using stoichiometric amounts of reagent and an acidic additive, the selectivity of the reduction can be controlled, and reducing agents such as tin(II) chloride,^43^ red phosphorus,^44^ and zinc^45^ enable the synthesis in moderate to good yields. Furthermore, the desired products can be obtained in high yields by hydrogenation with H_2_ and NaBH_4_.^46,47^ However, this requires expensive transition-metal catalysts and precisely controlled conditions, as this approach is widely used for reduction to anilines.^45,48,49^ Organic electrosynthesis can address these challenges, as selective reduction to hydroxylamine is well-established under appropriate conditions (Scheme 1C). In 1969, Lund reported the cathodic synthesis of the *N*-hydroxy quinolinone **5c** in a divided cell under potential controlled conditions using a mercury cathode with hydrochloric acid.^50^ Furthermore, an electrochemical protocol for the synthesis of 1*H*-1hydroxy-2-methylquinol-4-one (**5a**) was described by Tallec and co-workers using a mercury cathode with sulfuric acid as the supporting electrolyte.^51^ Both protocols enable in electroanalytic studies the access to a single example using highly toxic metal electrodes, which is nowadays banned in most countries for sensitive technical applications. Due to cathodic corrosion, heavy metal contamination of the product cannot be avoided.^52^

In this work, a simple, scalable, and versatile electrochemical method for the reductive cyclization of easy to prepare nitro arenes into 1*H*-1-hydroxyquinol-4-ones **5** is established (Scheme 1D). The electrolysis was performed in a commercially available electrochemical setup with commonly used sustainable carbon-based electrode materials to ensure high reproducibility and applicability.^53−55^ Organic electrosynthesis is experiencing a renaissance as an alternative to conventional synthetic protocols by considering sustainable aspects.^56−60^ This methodology can easily pay off as a key discipline for future synthetic applications for high value-added products, especially, in the synthesis of APIs.^61,62^ The use of electric current as an alternative to conventional reagents proves to be almost waste- and pollutant-free due to the absence of toxic and hazardous reagents, especially when solvents and supporting electrolytes are reused. Furthermore, these processes prove to be inherently safe due to the precise reaction control, as the conversion is immediately stopped by turning off the electricity and therefore preventing thermal runaway reactions.^63−68^

## Results and Discussion

Substrate **4a** was chosen as a test substrate for the optimization of the cathodic reduction synthesized in two steps from 2-nitrobenzoic acid in a high yield (see the ESI for a detailed description). Based on the work of Tallec and co-workers,^51^ Lund et al.,^50^ and Waldvogel,^69−71^ the initial electrolytic conditions for the cathodic reduction of nitro arene **4a** were chosen (Table 1, entry 1). A water–ethanol mixture (1:1 (*v*:*v*)) was used as a green solvent. Sulfuric acid plays a dual role as a supporting electrolyte and as a catalyst for the cyclo-condensation as well as the selective nitro reduction.^72^ Based on previous work, a sulfuric acid concentration of 0.5 M was used initially.^51,69,73^ Constant current electrolysis was performed in an undivided cell by applying the theoretical amount of charge (4 *F*) and a current density (4.7 mA cm^–2^). Electrolysis was performed utilizing carbon-based electrode materials, with glassy carbon (GC) as the anode and boron-doped diamond (BDD) as the cathode. BDD offers a unique reactivity toward electrochemical conversion of a multitude of substrates and fulfills sustainable requirements through its sustainable production using methane as a carbon source.^54,74−76^ Applying the described conditions, the desired 1-hydroxyquinonlin-4-one **5a** was obtained in 66% yield (Table 1, entry 1). Molecular structure could be unequivocally confirmed by X-ray analysis (CCDC: 2367756). Systematic variation of the electrolytic conditions was carried out in linear screening experiments.^77^ However, several parameters and counter reactions seem to play a crucial role for success.^55,78^ First, the influence of the cathode material was investigated with platinum, lead, and leaded bronzes. Platinum as the cathode material resulted in 59% requiring a precious and rare metal (Table 1, entry 2). To our delight, utilizing a lead cathode led to a shutdown of product formation **5a**, whereas the full reduction of the nitro group was observed resulting in the formation of 1*H*-2-methylquinol-4-one (**6a**) (Table 1, entry 3). Leaded bronzes are robust alternatives to pure lead electrodes, as they are more mechanically and chemically stable. However, by using a leaded bronze cathode, the product was only formed in 20% yield (Table 1, entry 4).^52,79^

**Table 1:**
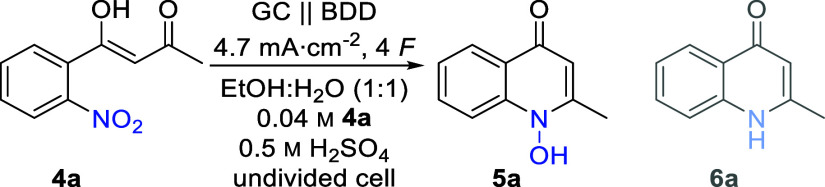
Optimization of the Electrolytic Conditions for the Cathodic Synthesis of 1*H*-1-hydroxy-2-methylquinol-4-one

entry	deviation from standard conditions	yield **5a**^a^
1	none	66%
2	Pt instead of BDD	59%
3	Pb instead of BDD	2% (**6a**: 24%)
4	CuSn5Pb20	20% (**6a**: 9%)
5	DSA (RuO_2_@Ta) instead of GC	64%
6	formic acid instead of H_2_SO_4_	57%
7	acetic acid instead of H_2_SO_4_	30%
8	acetate buffer instead of H_2_SO_4_	38%
9	methanol instead of ethanol	95% (92%^b^)
10	acetone instead of ethanol	52%
11	acetonitrile instead of ethanol	63%
12	MeOH:H_2_O (1:3)	91%
13	*c*(H_2_SO_4_) = 1.0 M; MeOH:H_2_O	86%
14	*c*(H_2_SO_4_) = 0.25 M; MeOH:H_2_O	78%
15	*j* = 3.0 mA cm^–2^; MeOH:H_2_O	76%
16	*j* = 6.0 mA cm^–2^; MeOH:H_2_O	83%

a Yield of **5a** was determined by ^1^H NMR spectroscopy using 1,3,5-trimethoxybenzene as an internal standard.

b Isolated yield.

To investigate the influence of the counter reaction, a dimensionally stable anode (DSA) with low overpotential for oxygen evolution reaction was used, resulting in a similar yield of 64% (Table 1, entry 5). Therefore, no advantage is gained for using an expensive transition-metal-based electrode material instead of glassy carbon. The influence of weaker organic acids was investigated by using formic acid and acetic acid instead of sulfuric acid, resulting in lower yields (57 and 30%) (Table 1, entries 6 and 7). Especially, acetic acid led in a high cell voltage (>32 V), so an acetic buffer was used. However, desired **5a** was obtained only in 38% yield (Table 1, entry 8). To our delight, the yield of **5a** was dramatically increased by using methanol (95%) and could be isolated in 92% yield (Table 1, entry 9). In comparison, aprotic solvents generally led to lower yields, e.g., acetone with 52% and acetonitrile with 63% (Table 1, entry 10 and 11). Lowering the MeOH content to the solubility limit of the starting material (water:methanol (1:3 (*v*:*v*))) resulted in a slightly decreased yield of 91% (Table 1, entry 12). Lower and higher sulfuric acid concentrations (0.25 and 1 M) resulted in a decreased yield of **5a** (86 and 78%), underlining the significant influence of the acid concentration on the outcome of the reaction (Table 1, entries 13 and 14). The increase (6.0 mA cm^–2^) and decrease (3.0 mA cm^–2^) in current density also led to lower yields (76 and 83%) (Table 1, entries 15 and 16). Full conversion of the starting material was observed by applying the theoretical amount of charge, which require no further optimization of this parameter.

**Chart 2:**
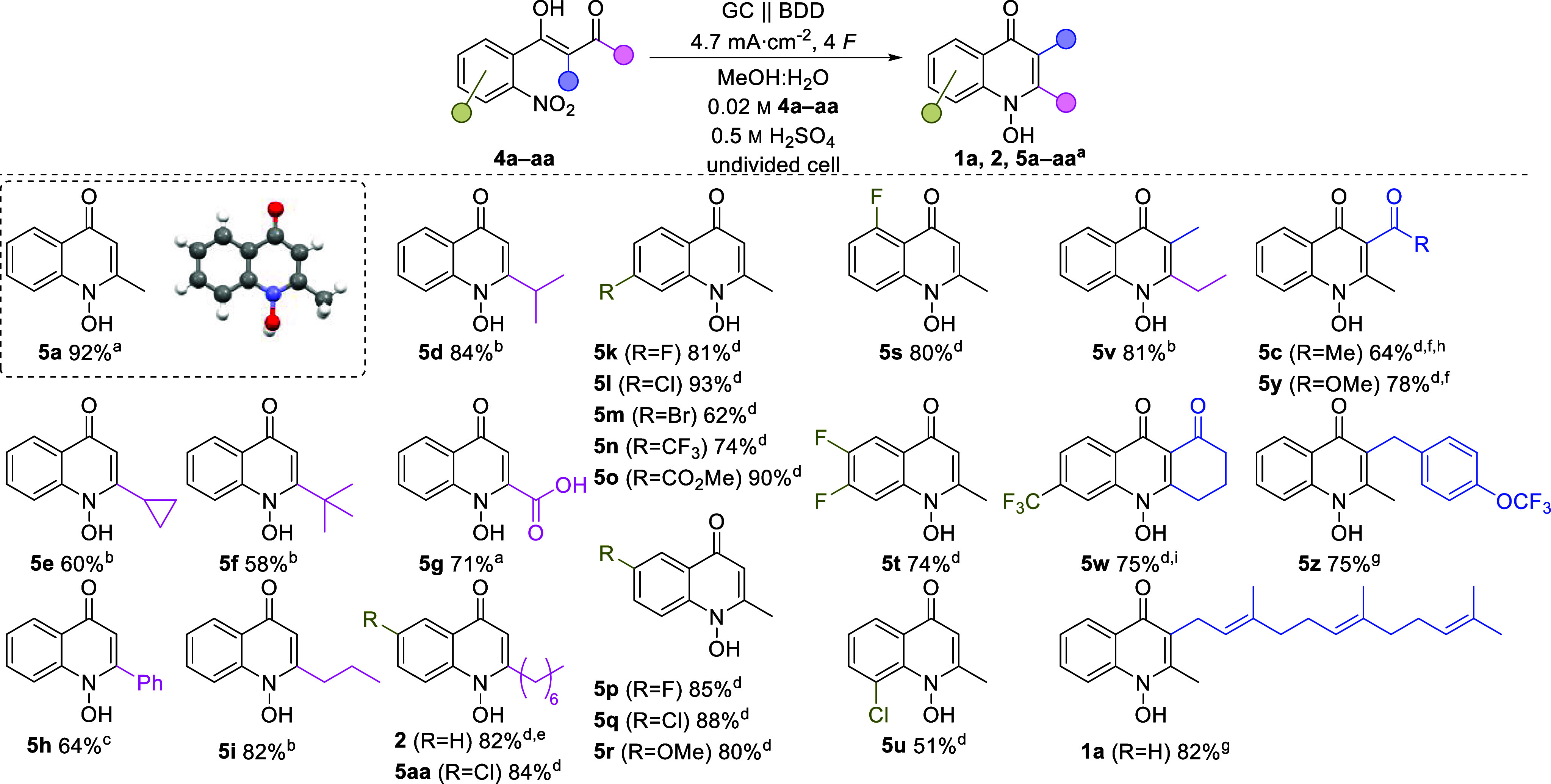
Scope of the Electrosynthesis of 1*H*-1-hydroxyquinol-4-ones with Isolated Yields on a 0.5 mmol Scale in 25 mL Beaker-Type Electrolysis Cells (SynLectro)

The optimized electrolytic conditions were applied to a broad and diverse range of substrates (Chart 2). However, due to the lower solubility in the electrolyte of the substituted starting materials **4c**–**y**, the methanol content needed to be increased, and the substrate concentration needed to be lowered to 0.02 M. The influence of primary, secondary, and tertiary alkyl substitutions was investigated in the synthesis of heterocycles **5d**–**f**, resulting in decreasing yield in accordance with increasing steric bulk (58–84%). Here, *tert*-butyl derivative **5f** gave the lowest yield. Interestingly, 2-cyclopropylquinol-4-one **5e** was obtained in a moderate yield of 60% compared to its corresponding isopropyl derivative **5d** with 84%. Furthermore, 1*H*-1-hydroxy-2-propylquinol-4-one (**5i**) as well as HQNO (**2**) with increasing hydrophobic side chains were obtained both in a good yield of 82%. The carboxylic acid **5g** was obtained in 71% yield by conversion of 2,4-dioxobutanoic acid. Phenyl substituted **5h** was isolated in 64% yield. However, an adapted solvent mixture of methanol, water, and acetone was required due to the low solubility of the starting material. The 2-amino and 2-oxo derivatives could not be obtained possibly due to the decreased reactivity of the corresponding nitrile and ethyl ester starting material in cyclo-condensation reactions.

a MeOH:H_2_O (1:1 (*v*:*v*)), 0.04 M **4a**, 1 mmol. b MeOH:H_2_O (2:1 (*v*:*v*)). c MeOH:acetone:H_2_O (40:9:1 (*v*:*v*:*v*)). d MeOH:H_2_O (3:1 (*v*:*v*)). e 4.5 *F*. f 2.4 mA cm^–2^. g MeOH:H_2_O (5:1 (*v*:*v*)). h Side product: **5a** 25%. i From Nitisinone.

After the influence of the 2-substitution was investigated, the impact of the modification of the nitro arene core (**5k**–**u**) was explored. Fluoro compounds **5k**, **5p**, **5s**, and **5t** were obtained in good yields of 85%, whereby no influence of the substitution pattern on the yield was determined. The chloro compounds **5l** and **5q** were obtained in very good yields of 93% and 88% respectively. The moderate yield (51%) of chloro compound **5u** should be attributed to the steric and electronic influence. Bromo compound **5m** was obtained in a good yield of 62%. The derivative bearing a trifluoromethyl group **5n** was obtained in a good yield of 74%. Derivatives with an electron-withdrawing group and an electron-donating group were obtained in very good yields. In particular, 6-methoxyquinol-4-one **5r** was obtained in 80% yield and the methyl ester **5o** in 90%. Resulting *N*-hydroxyquinolones (**5a**–**u**) could serve as novel precursors for biocatalytic prenylation, resulting in novel Aurachin C-based antibiotics.^35^

In addition, 2,3-disubstituted quinol-4-ones (**1a**, **5c**, and **5v**–**z**) were obtained in moderate to good yields. In particular, 2-ethyl-3-methylquinol-4-one **5v** bearing two alkyl substitutions was isolated in 81% yield. The tolerance of electrolytic conditions for 3-prenyl derivatives was demonstrated by the synthesis of a 3-farnesyl *N*-hydroxyquinolone (**1a**) in 82% yield, emphasized by the synthesis of the natural antibiotic Aurachin C (**1a**). **5w** was obtained in a good yield of 75% from Nitisinone, a medication to treat hereditary tyrosinemia type 1.^80^**5c** and **5y** containing a carbonyl moiety in 3-position were obtained in 64 and 78% yields, respectively. However, the synthesis of **5c** leads to an additional yield of **5a** (25%) by decarboxylation of the starting material **4c** under electrolytic conditions. **5z**, a novel drug candidate for tuberculosis therapy as a cytochrome oxidase inhibitor, was obtained in 75% yield.^81−83^ With **5aa**, a chloro-functionalized derivative of the natural antibiotic **2** was obtained in 84% yield, demonstrating the potential for the synthesis of novel drug candidates.

Considering preparative aspects, glassy carbon was tested as an alternative cathode material for BDD in the synthesis of **5a**, which led to slightly decreased selectivity and a yield of 87%. Furthermore, we focused on the multigram synthesis of the Aurachin C precursor **5a** (Table 2). Initially, a 4-fold scale-up was performed on a 4.0 mmol scale in a batch-type electrolysis using a 100 mL glass cell, resulting in a constant yield of 92% of **5a** accordingly, 643 mg. Importantly, gram-scale electrolysis was performed on a 12-fold scale (12.0 mmol) in a 300 mL glass cell, affording 1.871 g (89%) of the desired **5a**. Multigram electrolysis was conducted with a doubled substrate concentration, resulting in a slightly decreased yield of 87%, accordingly 3.651 g. The trend of decreased yield at higher concentrations coincides with previous work.^69,73^

**Table 2:**
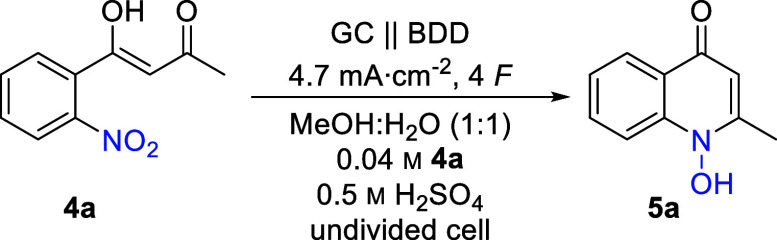
Scale-Up of the Electrochemical Synthesis of 1*H*-1-hydroxy-2-methylquinol-4-on (**5a**)

cell volume^a^	scale	yield **5a**
25 mL	1.0 mmol	161 mg (92%)
25 mL	1.0 mmol^b^	152 mg (87%)
100 mL	4.0 mmol	643 mg (92%)
300 mL	12 mmol	1.871 g (89%)
300 mL	24 mmol^c^	3.651 g (87%)

a Commercially available electrochemical cell (SynLectro).

b GC instead of BDD.

c 0.08 M **4a** in MeOH:H_2_O (1:1).

**Scheme 2:**
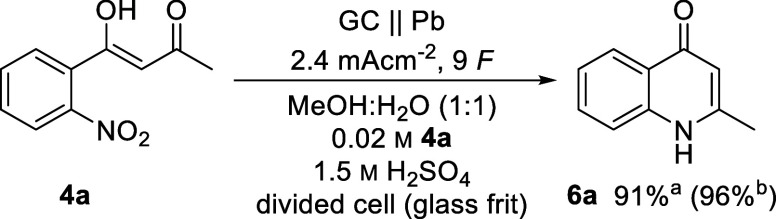
Optimized Conditions of the Electrochemical Synthesis of 1*H*-2-methylquinol-4-one (**6a**) Isolated yield. Yield determined by LC-MS using 8-hydroxyquinoline as an internal standard.

Based on the observation of selective deoxygenation of nitro arenes to their anilines by using lead or leaded bronzes, we investigated in addition in a proof-of-concept study the electrochemical synthesis of 1*H*-2-methylquinol-4-one **6a**. In contrast, reduction to **6a** on BDD cathodes could not be carried out in good yields. The linear optimization of the electrolytic conditions (see Tables S6–S13) resulted in deviated electrolytic conditions. Crucial for the full reduction of the nitro group proved to be stronger cathodic conditions by utilizing a lead cathode. As reported, a higher sulfuric acid concentration proved to be favorable for the nitro reduction to the aniline.^84^ A divided setup as well as lower current densities had a positive influence on the yield by preventing side reactions. In addition to the direct nitro reduction, a telescoped approach resulting from deoxygenation of **5a** into the desired product **6a** was possible. Full conversion was observed with 9 *F*, resulting in an isolated yield of 91% (Scheme 2).

**Scheme 3:**
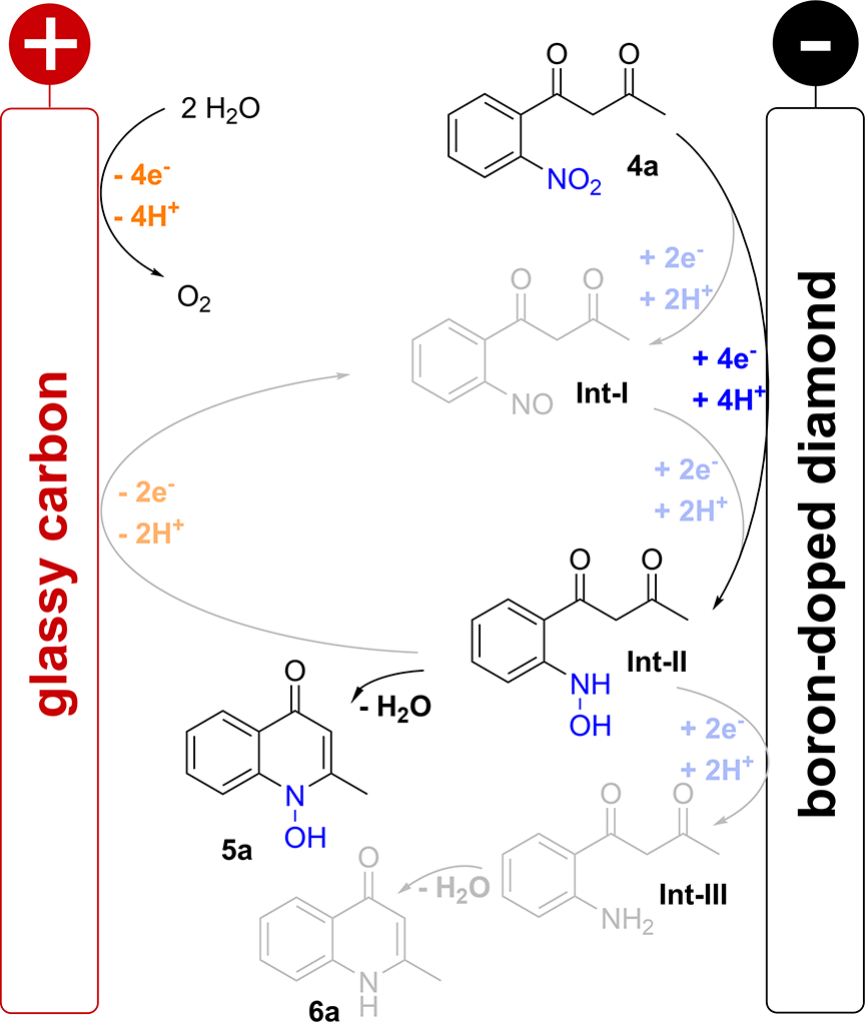
Proposed Mechanism for the Electrosynthesis of 1*H*-1-hydroxyquinol-4-ones **5a** and Over-Reduction to **6a**

Mechanistic considerations were performed based on reported data for the reduction of nitro arene **4a** and our previously reported studies on nitro reductions in *N*-hydroxy and *N*-oxy heterocycles synthesis as well as cyclic voltammetry measurements, resulting in a postulated mechanism (Scheme 3). The reduction of the nitro arene **4a** in two two-electron reduction steps at BDD electrodes results in the corresponding hydroxylamine **Int-II** via the nitroso intermediate **Int-I**. Two-electron steps in protic solvents have been occupied in multiple studies.^42,50−52,69,84^ However, the cyclic voltammogram (Figure S5) shows only a single broad reduction wave at −0.91 V, which accounts for the high selectivity to the hydroxylamine **Int-II**. A subsequent reduction of the product does not take place at the BDD. In contrast, a direct and selective reduction to the amine **Int-III** is observed at lead cathodes. This heavy metal electrode material possesses a high selectivity toward deoxygenation reactions and possesses a high overpotential for hydrogen evolution reaction.^54^ Subsequently, cyclo-condensation is accomplished in the desired product (**5a** or **6a**).

## Conclusions

In summary, the established electrochemical method provides simple, direct, and sustainable access to *N*-hydroxyquinol-4-ones **5** by selective cathodic nitro reduction and subsequent cyclo-condensation. In addition to the previous reports on the simple electrochemical synthesis of N,O heterocycles by reduction of nitro arenes, the protocol described adds up to these as a sustainable tool for the modern organic chemist in heterocycle chemistry highlighted by utilizing broadly available and sustainable carbon-based electrodes as well as an environmentally benign solvent. Sulfuric acid serves in a multiple role as a supporting electrolyte, a selectivity criterion for the nitro reduction, and an acidic catalyst for the subsequent cyclo-condensation. Applying a simple and commercially available electrochemical setup ensured high reproducibility. The broad applicability of the reported electrochemical protocol was demonstrated by diverse 26 examples in up to 93% isolated yield. The electrolytic conditions tolerate various functional groups, including sterically demanding, redox-labile substituents, as well as electron-withdrawing and -donating groups. The natural antibiotics Aurachin C (**1a**) and HQNO (**2**) with multiple potential pharmaceutical applications were isolated both in 82% yield. In proof-of-concept studies, electrochemical synthesis of 2-methylquinol-4-one **6a** was obtained in 91% by full nitro reduction. In mechanistic considerations, selective nitro reduction to the hydroxylamine at the BDD cathode was confirmed by cyclic voltammetry measurements as a key step. Robust scalability of this electrochemical protocol was demonstrated by multigram electrolysis.

## Supporting Information Available

General information, optimization data, electrochemical setup, experimental procedures, cyclic voltammetry data, compound characterization, and crystal structure data (PDF)
Molecular structure 5a (CIF)

## Supplementary Material

op4c00337_si_001.pdf

op4c00337_si_002.cif

## Abbreviations

BDD: boron-doped diamond
CCDC: Cambridge Crystallographic Data Center
DSA: dimensionally stable anode
GC: glassy carbon
HQNO: 1*H*-2-heptyl-1-hydroxyquinol-4-one
NADH: nicotinamide adenine dinucleotide phosphate
*m*CPBA: *meta*-chloroperoxybenzoic acid
