# Supplementary material for: Highly Selective Electrosynthesis of 1H-1-Hydroxyquinol-4-ones–Synthetic Access to Versatile Natural Antibiotics
Source: Org Process Res Dev. 2024 Sep 24;28(10):3922–8. doi: 10.1021/acs.oprd.4c00337 (PMC11494660; doi:10.1021/acs.oprd.4c00337)
Supplement: Supplementary file 1 — op4c00337_si_001.pdf [file op4c00337_si_001.pdf]

Supporting Information

Highly Selective Electrosynthesis of 1*H*-1-Hydroxyquinol-4-ones – Synthetic Access to Versatile Natural Antibiotics

Tobias Prenzel,[a] Nils Schwarz,[a] Jasmin Hammes,[a] Franziska Krähe,[a] Sarah Pschierer,[a] Johannes Winter,[a] María de Jesús Gálvez-Vázquez,[a] Dieter Schollmeyer,[a] Siegfried R. Waldvogel[a,b,c]*

[a] *Department of Chemistry, Johannes Gutenberg University, Duesbergweg 10–14, 55128 Mainz, Germany.*

[b] *Max-Planck-Institute for Chemical Energy Conversion, Mülheim an der Ruhr 45470, Stiftstraße 34 - 36, Germany.*

[c] *Institute of Biological and Chemical Systems - Functional Molecular Systems (IBCS-FMS), Karlsruhe Institute of Technology (KIT), Kaiserstraße 12, 76131 Karlsruhe, Germany*

E-Mail: siegfried.waldvogel@cec.mpg.de

1. General Information 2
2. General Protocols 6
   2.1. General Protocol for the Synthesis of 1-Hydroxy-1-(2-nitrophenyl)but-3-en-2-ones by Condensation and Decarboxylation of 2-Nitrobenzoyl chlorides with β-Keto acetates (GPI) 6
   2.2. General Protocol for the Synthesis of 1-Hydroxy-1-(2-nitrophenyl)but-1-en-3-ones by Condensation of 2-Nitrobenzoyl chloride with Ketones (GPII) 6
   2.3. General Protocol for the Electrochemical Synthesis of substituted 1*H*-1-Hydroxyquinol-4-ones (GPIII) 7
   2.4. General Protocol for the Electrochemical Synthesis of 1*H*-2-Methylquinol-4-one (GPIV) 7
3. Optimization of the Electrolytic Reaction Conditions 8
   3.1. Electrolytic Conditions for the Synthesis of 1*H*-1-Hydroxy-2-methylquinol-4-one (5a) 8
   3.2. Electrolytic Conditions for the Synthesis of 2-Methylquinol-4-one (6a) 11
4. Cyclic Voltammetry Studies 14
5. Preparation of Products and Analytical Data 15
   5.1. 1-Hydroxy-1-(2-nitrophenyl)but-3-en-2-ones (4a–aa) 15
   5.2. 1*H*-1-Hydroxyquinol-4-ones (1a, 2 ,5a–aa) 25
   5.3. 1*H*-Quinol-4-ones (6) 32
   5.4. *β*-Ketoesters (7) 32
   5.5. Prenyl halogenide (8) 33
6. Crystallographic Data 34
7. NMR Spectra 36
8. References 97

1. General Information

If not stated otherwise, all reactions were performed under ambient conditions and chemicals in analytical grade were used as purchased without further purification. Cyclohexane and ethyl acetate were purchased in technical grade and purified by distillation under reduced pressure prior to use. Milli-Q® water was obtained using Simplicity® System (UV) (*Merck KGaA*, Darmstadt, Germany) for chromatography purposes. Anhydrous solvents were obtained from a solvent purification system SPS-5 (*M. Braun Incorporated*, Stratham, USA).

Chromatography
Thin layer chromatography was performed using DC Kieselgel 60 F254 on aluminum plates (*Merck KGaA,* Darmstadt, Germany). A UV lamp (λ = 254 nm, NU-4 KL, *Benda*, Wiesloch, Germany). Preparative flash column chromatography was performed on silica gel 60 M (0.040–0.063 mm, 80 g, *Macherey-Nagel GmbH & Co*, Düren, Germany) using a Büchi Pure C-815 Flash (*Büchi-Labortechnik GmbH*, Essen, Germany) or on a prepacked puriFlash® SI-HP silica PF-25SIHC-F0080 column (*Interchim SAS*, Montluçon Cedex, France) using a puriFlash® XS 520 Plus system (*Interchim SAS*, Montluçon Cedex, France). Reversed phase column chromatography of the different 1*H*-1-hydroxyquinol-4-ones derivatives was performed on Puriflash® PF-30C18HP-F0080 (*Interchim SAS*, Montluçon Cedex, France) using a Sepacore® system with a Büchi Control Unit C-620, Büchi Pump Modules C-605, a UV detector Büchi UV photometer C-635, and Büchi Fraction Collector C-660 (*Büchi-Labortechnik GmbH*, Essen, Germany) using different mixtures of water (0.1% formic acid (*v/v*)) and acetonitrile as eluents.

High Resolution Mass Spectrometry
Mass spectra *via* electrospray-ionization (ESI+/ESI–) mass spectrometry were recorded using an Agilent 6545 QTOF-MS (*Agilent*, Santa Clara (CA), USA). Mass-to-charge ratios (*m*/*z*) were obtained for the characterized compounds.

X-ray Crystallography
The measurements of the crystal structures were carried out on a STOE IPDS-2T (*STOE & Cie GmbH*, Darmstadt, Germany) using a Mo source with graphite tube monochromator.

High Performance Liquid Chromatography (HPLC)
Analysis of crude reaction mixtures and purified products was performed using a modular system LC-20A *Prominence* (*Shimadzu Deutschland GmbH*, Duisburg, Germany), UV/VIS-detector SPD-20A/AV (*Shimadzu Deutschland GmbH*, Duisburg, Germany), and LCMS-2020 Single Quadrupole (*Shimadzu Deutschland GmbH*, Duisburg, Germany). Analytical separation was performed using an Eurospher II 100-5 C-18-Trennsäule (*Knauer Wissenschaftliche Geräte GmbH*, Berlin, Germany) column (length of 150 mm, diameter of 4 mm, pore size of 100 Å, particle size 5 µm). As eluents, acetonitrile and water with 5% (*v*/*v*) acetonitrile and formic acid (0.1% (*v*/*v*)) were used. Given retention times were obtained at λ = 254 nm.

Nuclear Magnetic Resonance (NMR) Spectroscopy
Nuclear magnetic resonance experiments were performed using a nuclear magnetic resonance spectrometer Avance II 400 (*Bruker*, Karlsruhe, Germany) ^{1}H NMR (400 MHz), ^{13}C NMR (101 MHz) and ^{19}F NMR (376 MHz) and Avance III 600 (*Bruker*, Karlsruhe, Germany) ^{1}H NMR (600 MHz), ^{13}C NMR (151 MHz) The spectra were recorded using deuterated solvents. To normalize the spectra obtained, reference was made to the existing solvent signal of non-deuterated fractions according to the data provided by *Fulmer et al.*[1]: chloroform-d_1 (^{1}H NMR: δ = 7.26 ppm, ^{13}C NMR: δ = 77.2 ppm), DMSO-d_6 (^{1}H NMR δ = 2.50 ppm, ^{13}C NMR: δ =39.5 ppm) and water-d_2 (^{1}H NMR δ = 4.79 ppm). Besides ^{1}H, ^{13}C and ^{19}F NMR experiments, the 2D techniques ^{1}H,^{1}H COSY, ^{1}H,^{13}C HSQC and ^{1}H,^{13}C HMBC were used assisting to assign the signals. The following abbreviations were used to describe the signals: s (singlet), d (doublet), t (triplet), m (multiplet), q (quartet), sep (septet), dd (doublet of doublets), ddd (doublet of doublets of doublets), td (triplet of doublets), tt (triplet of triplets), tq (triplet of quartets), and bs (broader singlet). The spectra obtained were evaluated with MestReNova 14.2.0-26256 (*Mestrelab Research S.L.*, Spain). ^{19}F NMR spectra were obtained with α-trifluorotoluene as external standard (δ = 63.9 ppm) and recorded without ^{1}H decoupling.

Cyclic Voltammetry (CV) Measurements
The mechanism of the reaction was studied by cyclic voltammetry using an electrochemical glass cell (figure S1) (*Metrohm AG*, Herisau, Switzerland) equipped with a BDD (with a diameter of 4 mm), a glassy carbon rod and an Ag/AgCl (saturated LiCl in ethanol, *Metrohm AG*, Herisau, Switzerland) as

working, counter and reference electrode, respectively. The electrode potentials are reported with reference to the redox system ferrocene/ferrocenium ion (FcH/FcH^+).
Cyclic voltammograms were measured using a potentiostat/galvanostat PGSTAT302N (*Metrohm AG*, Herisau, Switzerland) with a scan rate of 50 mV s^{-1} in a methanol (HPLC LC-MS grade, *VWR International GmbH*, Darmstadt, Germany) and water solution (1:1 v/v) containing 0.5 M of H_2SO_4 (analytical reagent grade, 96%, *Fisher Scientific GmbH*, Schwerte, Germany) and 5 mM of the corresponding molecule. Prior to the CV measurements, the electrolyte was degassed with an Ar flow for 25 min. An Ar atmosphere was kept flowing over the electrolyte during the measurements.

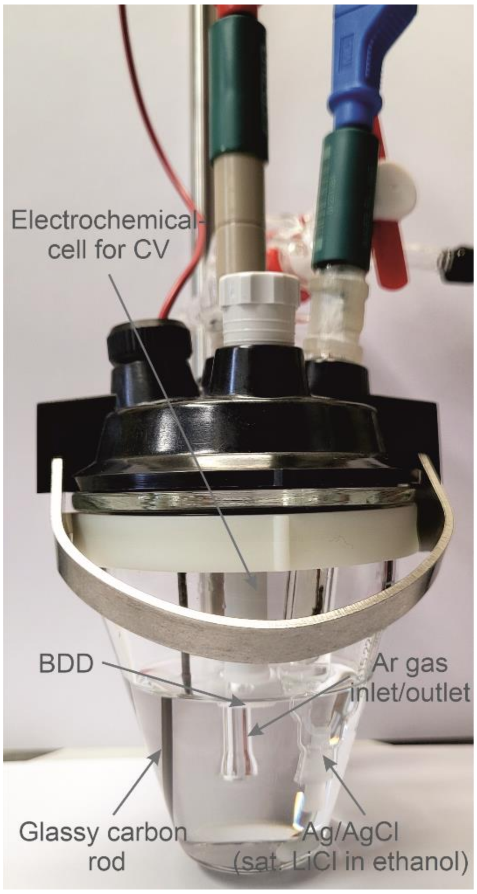

Figure S1: Electrochemical cell for CV measurements.

Electrochemical Set-Up
Electrochemical reactions were carried out using a multichannel galvanostat HMP4040 (*Rohde & Schwarz*, München, Germany) or a TDK-Lambda Z+ series (*TDK-Lambda UK Limited*, Devon, United Kingdom). The different cells used for screening or batch reactions are described below.

Electrochemical Screening or Batch-type Reactions
Teflon™ cells with a volume of 5 mL were used for the undivided set-up (figure S2, left). Divided Teflon™ screening cells with a volume of 7 mL were equipped with a glass frit or Nafion™ as separator material as shown (figure S2, right). Glass frits and Nafion™ used as separator materials were pre-treated in the corresponding electrolyte prior to use. Stirring bars were used during electrolysis in each cell. The described screening systems are commercially available as IKA Screening System Package (*IKA™ Werke GmbH & Co. KG*, Staufen, Germany).

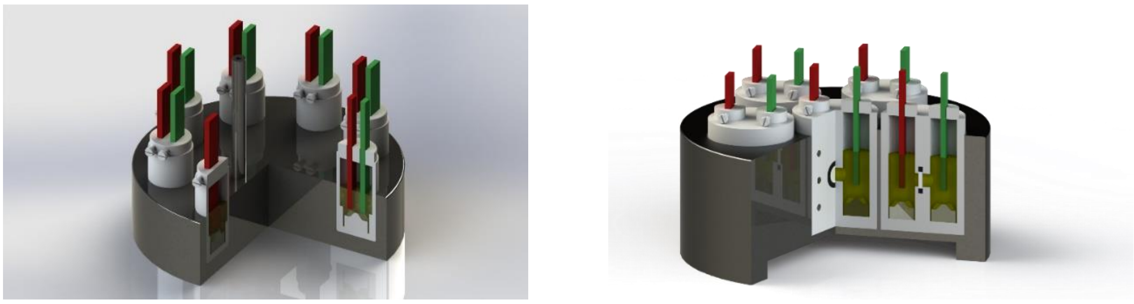

Figure S2: Undivided screening set-up (left) and divided screening set-up (right).[2]

Scale-up experiments were performed in 25 mL, 100 mL, and 300 mL batch-type cells with a PTFE stopper and sleeve, electrodes, and electrode holders (figure S3), which are commercially available as SynLectro™ Starter Kit (*Merck KGaA*, Darmstadt, Germany).[3] In the 25 mL electrolysis set-up glassy carbon and BDD electrodes with dimensions of 6 cm x 2 cm were used. In the 100 mL electrolysis set-up glassy carbon and BDD electrodes with identical dimensions of 6 cm x 2 cm were used. In the 300 mL electrolysis set-up glassy carbon and BDD electrodes with identical dimensions of 12 cm x 4 cm were used.

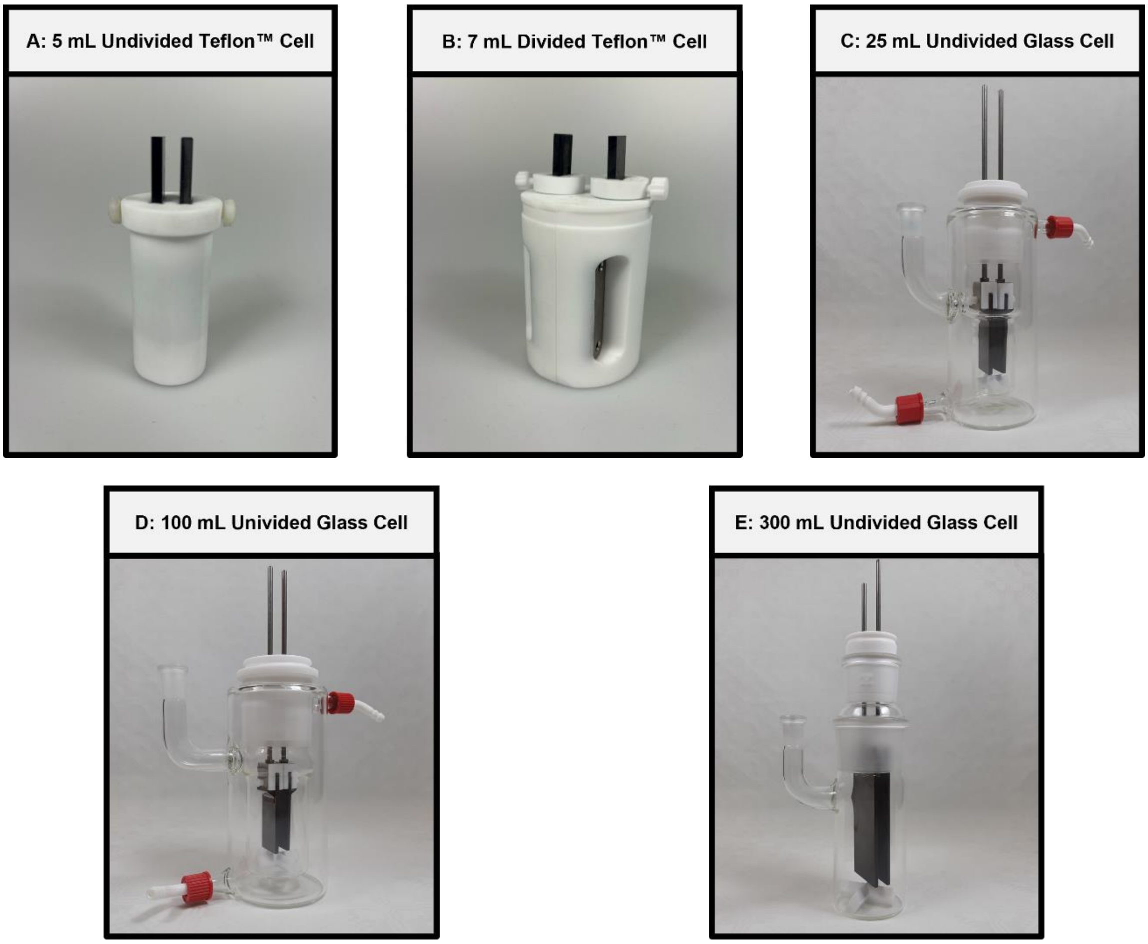

Figure S3: Different batch-type cells: **A** 5 mL undivided Teflon™ screening cell with glassy carbon and BDD electrodes; **B** 7 mL divided Teflon™ screening cell with glassy carbon and BDD electrodes; **C** 25 mL undivided glass cell with glassy carbon and BDD electrodes; **D** 100 mL undivided glass cell with glassy carbon and BDD electrodes; **E** 300 mL divided glass cell with glassy carbon and BDD electrodes.

Electrode Materials

Table S1: Electrode materials, purity, and their supplier.

Entry	Electrode Material	Purity	Supplier
1	Boron-doped diamond (DIACHEM™)	15 µm boron-doped diamond layer on silicon	*CONDIAS GmbH*, Itzehoe, Germany
2	Glassy carbon (Sigradur G)	-	*HTW*, Thierhaupten, Germany
3	Platinum	>99%	*OEGUSSA*, Vienna, Austria
4	Lead	Roofer's lead (>98% Pb)	*Globus Fachmärkte GmbH & Co. KG,* Völklingen, Germany
5	Dimensionally Stable Anode (DSA)	IrOx on tantalum	*DeNora S.p.A.*, Milano, Italy
6	Stainless steel (1.4571)		*Rheinischer Eisenhandel GmbH*, Alzey, Germany
7	Tin		*Goodfellow GmbH,* Hamburg, Germany
8	CuSn5Pb20		*Metallwerk Langenau GmbH,* Langenau, Germany
9	CuSn7Pb15		*Metallwerk Langenau GmbH,* Langenau, Germany
10	CuSn10Pb10		*Metallwerk Langenau GmbH,* Langenau, Germany

2. General Protocols

2.1. General Protocol for the Synthesis of 1-Hydroxy-1-(2-nitrophenyl)but-3-en-2-ones by Condensation and Decarboxylation of 2-Nitrobenzoyl chlorides with β-Keto acetates (GPI)

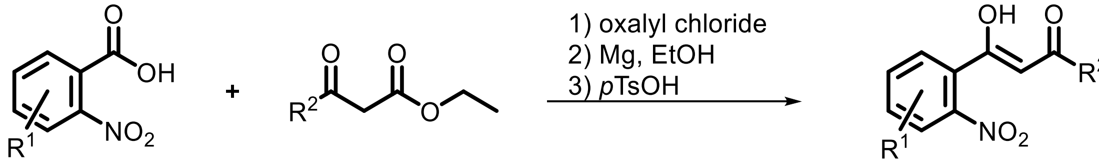

Scheme S1: Synthesis of 1-hydroxy-1-(2-nitrophenyl)but-1-en-3-ones.

Benzoic acid (18 mmol, 1 equiv.) was suspended in anhydrous dichloromethane and two drops of *N*,*N*-dimethylformamide was added. Oxalyl chloride (27 mmol, 1.5 equiv.) was added dropwise, and the reaction solution was stirred at room temperature until no more gas evolution was observed. After completion of the reaction, the solvents and excess oxalyl chloride were removed under reduced pressure. The crude benzoyl chloride was used without further purification. Magnesium turnings (18 mmol, 1 equiv.) were placed in 1 mL of dry ethanol and mixed with 0.1 mL carbon tetrachloride. 7 mL toluene, ethanol (30.8 mmol, 1.7 equiv.) and ethyl acetoacetate (18 mmol, 1 equiv.) were added so that the reaction mixture boiled slightly. The reaction mixture was then refluxed until the magnesium was consumed. The benzoyl chloride was then dissolved in 5 mL anhydrous THF, added dropwise, and the mixture was stirred overnight at room temperature. The resulting solution was neutralized with a mixture of 18 g ice and 1 mL concentrated sulfuric acid. The aqueous fraction was then extracted twice with 100 mL ethyl acetate. The combined organic fractions were diluted with water and dried over sodium sulfate. The solvent was removed under reduced pressure. The crude ethyl 2-(2-nitrobenzoyl)-acetoacetate (18 mmol, 1 equiv.) was dissolved in 100 mL of water, *para*-toluenesulfonic acid (0.9 mmol, 0.05 equiv.) was added and the mixture was heated under reflux until no gas evolution was recognizable. The aqueous phase was then extracted twice with 100 mL ethyl acetate. The combined organic fractions were extracted twice with 150 mL 10% potassium hydroxide solution. The combined aqueous fractions were neutralized with concentrated hydrochloric acid and extracted twice with 150 mL ethyl acetate. The combined organic fractions were washed with water, brine, dried over anhydrous sodium sulfate and the solvent was removed under reduced pressure. The crude product was purified by column chromatography or crystallization.

2.2. General Protocol for the Synthesis of 1-Hydroxy-1-(2-nitrophenyl)but-1-en-3-ones by Condensation of 2-Nitrobenzoyl chloride with Ketones (GPII)

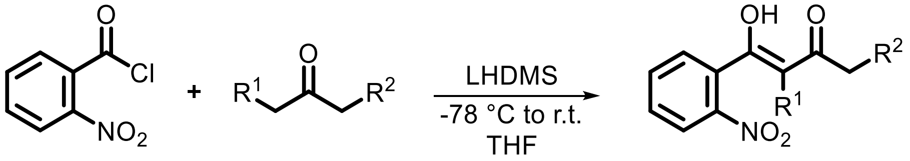

Scheme S2: Synthesis of 1-hydroxy-1-(2-nitrophenyl)but-1-en-3-ones.

A solution of the corresponding ketone (10 mmol, 1 eq) in 50 mL anhydrous THF was cooled to -78 °C and a 1 M solution of LHMDS in THF (20 mL, 1.9 eq) was added. After stirring at -78 °C for 1 h, the solution was warmed to room temperature and stirred for 2 h. The solution was cooled to -78 °C, 2-nitrobenzoyl chloride (10 mmol, 1 eq) was added and the solution was allowed to rise to room temperature. After stirring at room temperature overnight, the reaction was quenched with 50 mL saturated NH_4Cl solution, and the pH was adjusted to 8. The aqueous fraction was extracted three-times with ethyl acetate. The combined organic fractions were washed with brine, dried over sodium sulfate and concentrated in vacuo. The crude product was purified by column chromatography or crystallization.

2.3. General Protocol for the Electrochemical Synthesis of substituted 1*H*-1-Hydroxyquinol-4-ones (GPIII)

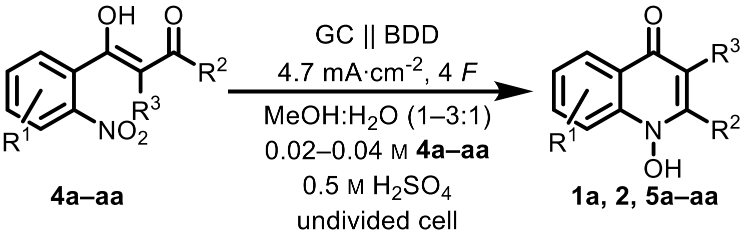

Scheme S3: Synthesis of substituted 1*H*-1hydroxyquinol-4-ones.

5 mL undivided Teflon™ screening cell: 0.1–0.2 mmol of 1-hydroxy-1-(2-nitrophenyl)but-1-en-3 ones (1 equiv.) were dissolved in 2.5–4.2 mL methanol. Subsequently, 0.8–2.5 mL of 1–3 M sulfuric acid was added. The Teflon™ cell was equipped with two electrodes, a glassy carbon electrode as anode and a BDD electrode as cathode (immersion depth: 1.7 cm, cathode surface: 1.7 cm^2). While stirring, the appropriate amount of charge was applied (current density j = 4.7 mA·cm^{-2} for 38.6 C–77.2 C (4.0 *F*)). After electrolysis, the reaction mixture was transferred to a round bottom flask and neutralized with 1 M aqueous sodium hydrogen carbonate. The solvent was removed under reduced pressure, the residue was redissolved in methanol and then separated from the sodium sulfate by filtration. The crude product was quantified by ^{1}H NMR spectroscopy using 1,3,5-trimethoxybenzene (0.2 mmol, 33.6 mg) as internal standard. The mixture was completely dissolved in 1 mL of DMSO-d_6 and analysed by ^{1}H NMR.

25 mL/100 mL undivided glass cell: 1-Hydroxy-1-(2-nitrophenyl)but-1-en-3 one (**4a**) was dissolved in 12.5/50 mL methanol and 12.5/50 mL 1 M sulfuric acid was added. The electrolysis was performed under constant current conditions (current density j = 4.7 mA·cm^{-2} (immersion depth 3/4 cm, electrode surface 6/8 cm^2) for 396/1544 C (4.0 *F*)). After electrolysis, the reaction solution was transferred to a round bottom flask and neutralized with 1 M sodium hydrogen carbonate solution. The solvent was removed under reduced pressure, the residue was redissolved in methanol and then separated from the sodium sulfate by filtration. The crude product was purified by reverse phase column chromatography (C18).

300 mL undivided glass cell: 1-Hydroxy-1-(2-nitrophenyl)but-1-en-3 one (**4a**) was dissolved in 150 mL methanol and 150 mL 1 M sulfuric acid was added. The electrolysis was performed under constant current conditions (current density j = 4.7 mA·cm^{-2} (immersion depth 8 cm, electrode surface 32 cm^2) for 4631/9263 C (4.0 *F*)). After electrolysis, the reaction solution was transferred to a round bottom flask and neutralized with 1 M sodium hydrogen carbonate solution. The solvent was removed under reduced pressure, the residue was redissolved in methanol and filtered. The crude product was purified by crystallization (50 mL EtOH; crystallization at -24 °C) or reverse phase column chromatography (C18).

2.4. General Protocol for the Electrochemical Synthesis of 1*H*-2-Methylquinol-4-one (GPIV)

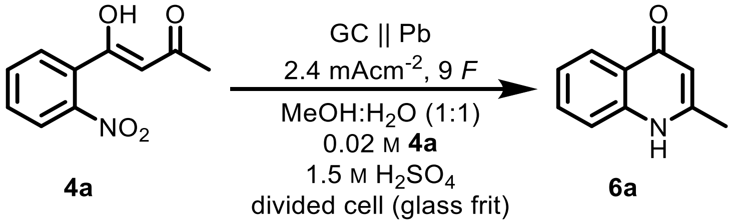

Scheme S4: Synthesis of 1*H*-2-methylquinol-4-one.

5 mL divided Teflon™ screening cell: 0.1 mmol of 1-hydroxy-1-(2-nitrophenyl)but-1-en-3 one (1 equiv.) was dissolved in 2.5 mL methanol. Subsequently, 2.5 mL of 3 M sulfuric acid was added. The Teflon™ cell was equipped with two electrodes, a glassy carbon electrode as anode and a BDD electrode as cathode. The anodic chamber was separated with a glass frit (Robu™ Por. 4, *ROBU Glasfilter-Geräte GmbH*, Hattert, Germany). While stirring, the appropriate amount of current was applied (current density j = 2.4 mA·cm^{-2} for 86.8 C (9.0 *F*)). After electrolysis, the reaction solution was transferred to a round bottom flask and neutralized with 1 M sodium hydrogen carbonate solution. The solvent was removed under reduced pressure, the residue was redissolved in methanol and then separated from the sodium sulfate by filtration. The crude product was purified by reverse phase column chromatography (C18).

3. Optimization of the Electrolytic Reaction Conditions

3.1. Electrolytic Conditions for the Synthesis of 1*H*-1-Hydroxy-2-methylquinol-4-one (5a)

The optimization experiments of the electrolytic conditions for the cathodic synthesis of 1*H*-1-hydroxy-2-methylquinol-4-one (**5a**) were carried out according to **GPIII** using 4-hydroxy-4-(2-nitrophenyl)but-3-en-2-one (**4a**) as test substrate. Initial electrolytic conditions were chosen based on previous publications. The yield of 1*H*-1-hydroxy-2-methylquinol-4-one (**5a**) was determined by ^{1}H NMR spectroscopy using 1,3,5-trimethoxybenzene as internal standard according to **GPIII**. The signals were assigned according to figure S4.

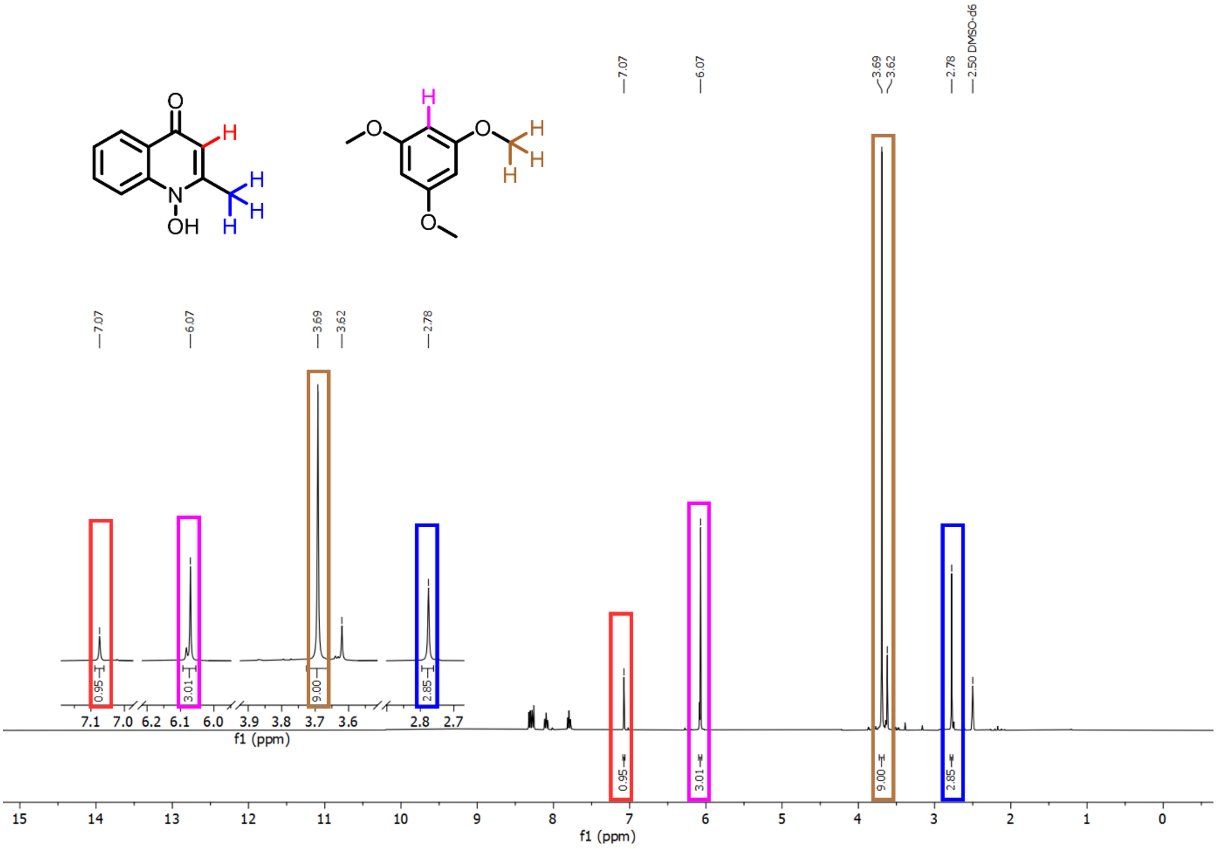

Figure S4: Assignment of the ^{1}H NMR signals for NMR quantification of the electrolytic conditions.

Table S2: Influence of the cathode material.

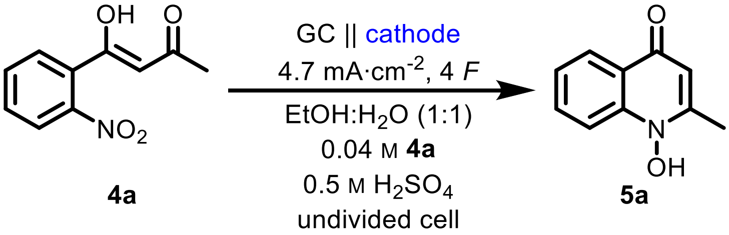

Entry	Cathode	Anode	Yield[a] **5a** [%]
1	BDD	GC	66
2	Pt	GC	59
3	Pb	GC	2[b]
4	CuSn5Pb20	GC	20[c]
5	BDD	DSA	64

[a]Yield determined by ^{1}H NMR, internal standard: 1,3,5-trimethoxybenzene; [b] 24% of **6a**; [c] 9% of **6a**.

Table S3: Influence of the supporting electrolyte.

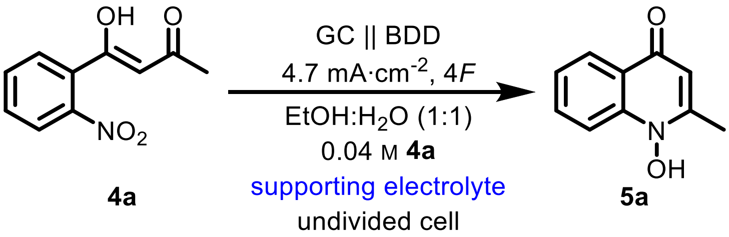

Entry	Supporting Electrolyte	Yield[a] **5a** [%]
1	0.5 M H_2SO_4	66
2	1.0 M formic acid	57
3	1.0 M acetic acid[b]	30
4	0.5 M NaOAc/AcOH buffer[b]	38

[a]Yield determined by ^{1}H NMR, internal standard: 1,3,5-trimethoxybenzene; [b]Cell voltage >32 V; [c]Prepared as 1 M aqueous stock solution from 180 mmol AcOH and 20 mmol NaOAc in 200 mL water.

Table S4: Influence of the organic solvent.

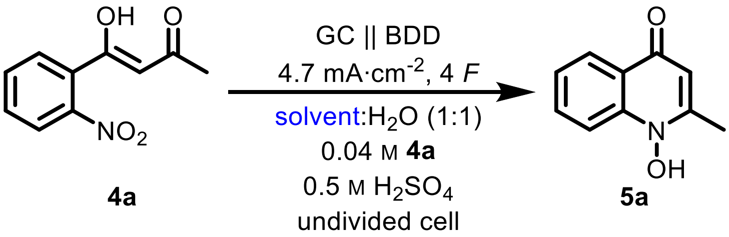

Entry	Organic Solvent	Yield[a] **5a** [%]
1	MeOH	95
2	EtOH	66
3	2-propanol	83
4	HFIP	92
5	acetonitrile	63
6	acetone	52
7	MeOH (1:3)[b]	91

[a]Yield determined by ^{1}H NMR, internal standard: 1,3,5-trimethoxybenzene; [b]Solubility limit of the starting material, precipitation of the starting material with lower methanol content.

Table S5: Influence of the sulfuric acid concentration.

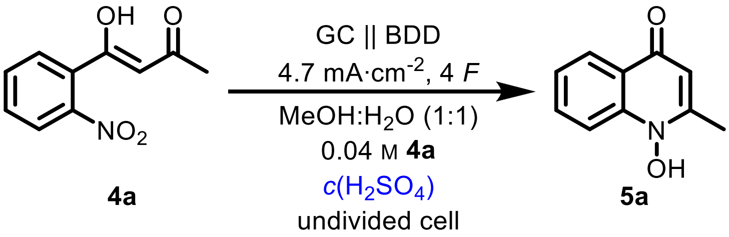

Entry	$c(H_2SO_4)$ / M	Yield[a] **5a** [%]
1	1.0	86
2	0.5	95
3	0.25	78
4	0.05	73
5	0.025	69
6	0.005	32

[a]Yield determined by ^{1}H NMR, internal standard: 1,3,5-trimethoxybenzene.

Table S6: Influence of the current density.

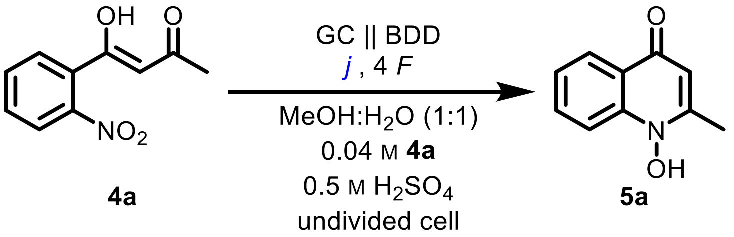

Entry	*j* / mA·cm^{-2}	Yield[a] **5a** [%]
1	3.0	76
2	4.7	95
3	6.0	83

[a]Yield determined by ^{1}H NMR, internal standard: 1,3,5-trimethoxybenzene.

3.2. Electrolytic Conditions for the Synthesis of 2-Methylquinol-4-one (6a)

The optimization experiments of the electrolytic conditions for the cathodic synthesis of 2-methylquinol-4-one (**6a**) were carried out according to **GPIV** using 4-hydroxy-4-(2-nitrophenyl)but-3-en-2-one (**4a**) as test substrate. Initial electrolytic conditions were adjusted from the previous optimization, applying higher current densities and higher amounts of charge to perform the following N,O reduction. The yield of 2-methylquinol-4-one (**6a**) beside 1*H*-1-hydroxy-2-methylquinol-4-one (**5a**) were determined by HPLC using 8-hydroxyquinoline as internal standard and an external calibration (figure S5). For calibration, 0.1 M stock solutions of **5a**, **6a** and 8-hydroxyquinoline in methanol were used to provide samples in the substance ratio of 10–100% of **5a** and **6a** to 8-hydroxychinoline by a concentration of 0.02 M from 8-hydroxyquinoline. According to the general protocol for the electrochemical synthesis of 1*H*-2-methylquinol-4-one (**GPIV**) after electrolysis 0.1 mmol (14.5 mg) of 8-hydroxyquinoline was added. From the calibration samples or the electrolysis samples approximately 0.05 mL was taken and diluted to 1.5 mL with acetonitrile. Evaluation was performed with peak areas at 254 nm.

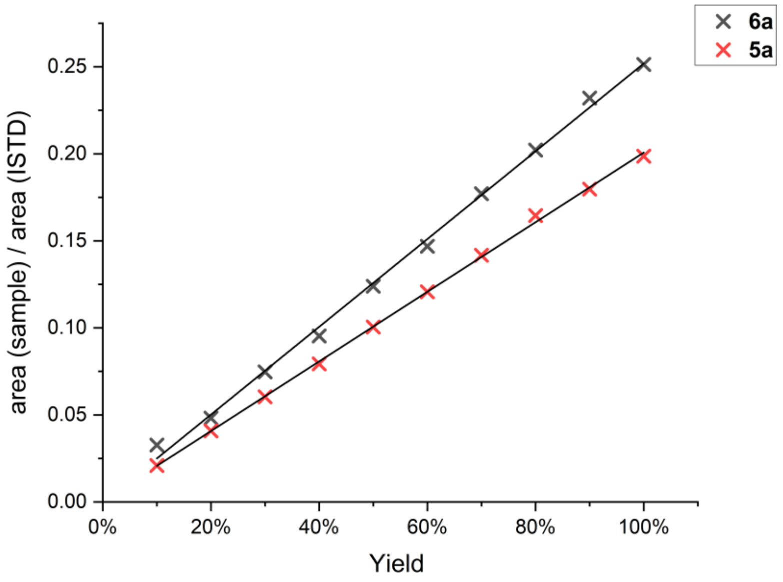

Figure S5: External calibration by LC-MS of 1*H*-2-methylquinol-4-one (**6a**) and 1*H*-1-hydroxy-2-methylquinol-4-one (**5a**) using 8-hydroxyquinoline as internal standard (ISTD).

Table S7: Initial screening experiments by adjusting *j* and *Q* to the previously optimized electrolytic conditions.

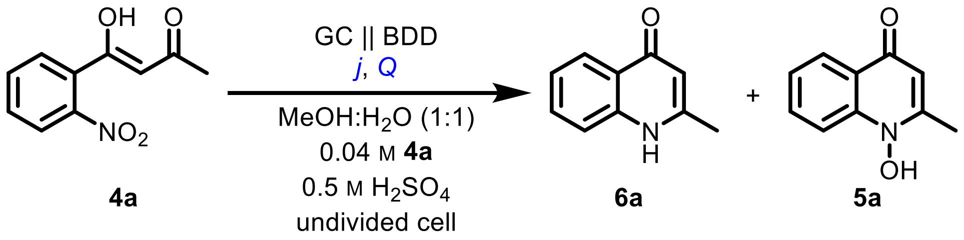

Entry	*j* / mA·cm^{-2}	*Q* / *F*	Yield[a] **6a** [%]	Yield[a] **5a** [%]
1	4.8	6	10	43
2	7.2	6	19	44
3	9.6	6	8	54
4	12.0	6	5	22
5	7.2	8	21	54
6	7.2	10	25	49
7	7.2	12	9	45
8	7.2	14	7	1

[a]Yield determined by LC-MS using 8-hydroxyquinoline as internal standard and external calibration.

Table S8: Influence of the cathode material in an undivided electrolysis cell.

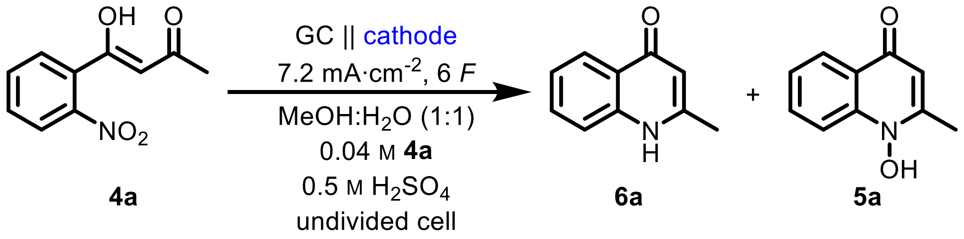

Entry	Cathode	Yield[a] **6a** [%]	Yield[a] **5a** [%]
1	BDD	21	54
2	GC	14	93
3	Pt	6	72
4	stainless steel (VA 1.4571)	9	88
5	Sn	38	83
6	Pb	47	0
7	CuSn7Pb15	27	54
8	CuSn10Pb10	28	80
9	CuSn5Pb20	35	69

[a]Yield determined by LC-MS using 8-hydroxyquinoline as internal standard and external calibration.

Table S9: Influence of the cathode material in the telescoped synthesis in an undivided electrolysis cell.

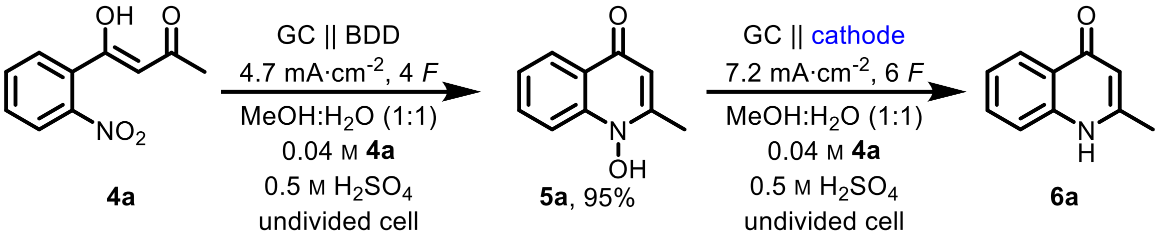

Entry	Cathode	Yield[a] **6a** [%]	Yield[a] **5a** [%]
1	BDD	13	76
2	GC	11	87
3	Pt	12	71
4	Sn	21	82
5	Pb	54	24
6	CuSn7Pb15	27	52
9	CuSn5Pb20	33	69

[a]Yield determined by LC-MS using 8-hydroxyquinoline as internal standard and external calibration.

Table S10: Influence of the cathode material in a divided electrolysis cell using a glass frit as separator.

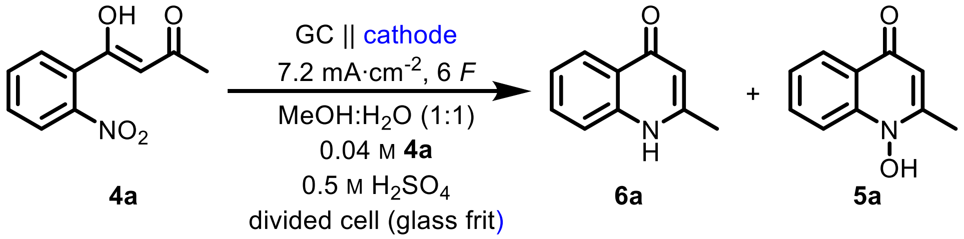

Entry	Cathode	Yield[a] **6a** [%]	Yield[a] **5a** [%]
1	BDD	44	57
2	Sn	44	56
3	Pb	57	6
4	CuSn7Pb15	30	71
5	CuSn10Pb10	31	33
6	CuSn5Pb20	35	24

[a]Yield determined by LC-MS using 8-hydroxyquinoline as internal standard and external calibration.

Table S11: Influence of the concentration of the sulfuric acid.

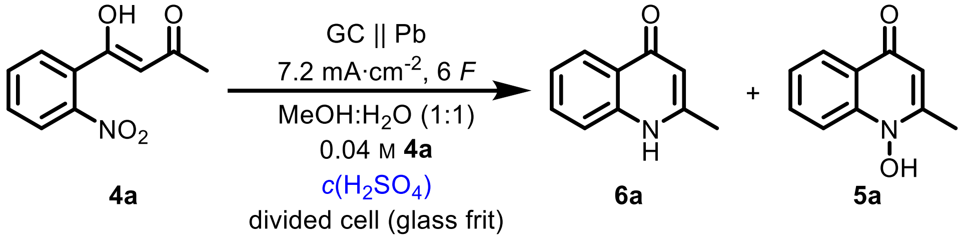

Entry	$c(H_2SO_4)$ / M	Yield[a] **6a** [%]	Yield[a] **5a** [%]
1	1.5	63	3
2	1.0	59	4
3	0.5	57	6
4	0.25	32	1

[a]Yield determined by LC-MS using 8-hydroxyquinoline as internal standard and external calibration.

Table S12: Influence of the amount of applied charge.

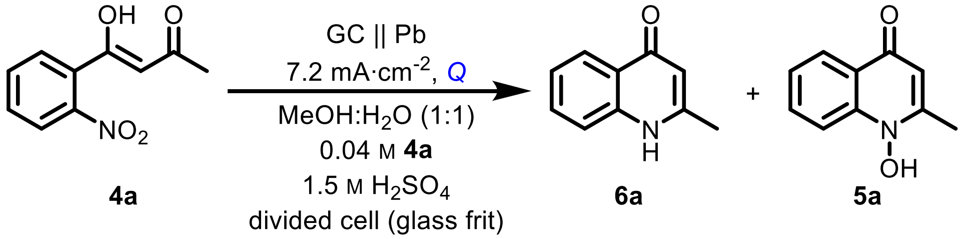

Entry	*Q* / *F*	Yield[a] **6a** [%]	Yield[a] **5a** [%]
1	6	63	3
2	7	65	2
3	8	71	1
4	9	75	3
5	10	63	0

[a]Yield determined by LC-MS using 8-hydroxyquinoline as internal standard and external calibration.

Table S13: Influence of the current density.

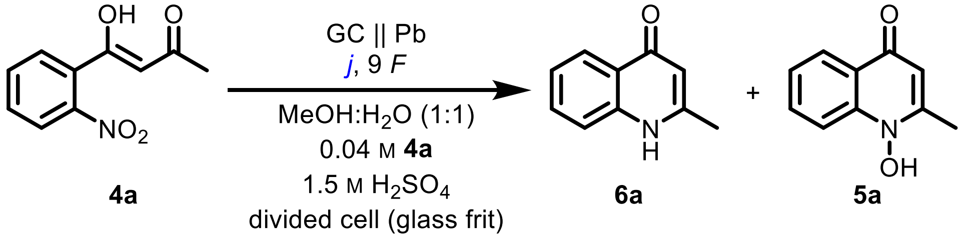

Entry	j / $mA \cdot cm^{-2}$	Yield[a] **6a** [%]	Yielda **5a** [%]
1	2.4	96	0
2	3.2	94	0
3	4.8	83	0
4	6.0	78	0
5	7.2	75	3
7	9.6	36	5

[a]Yield determined by LC-MS using 8-hydroxyquinoline as internal standard and external calibration.

4. Cyclic Voltammetry Studies

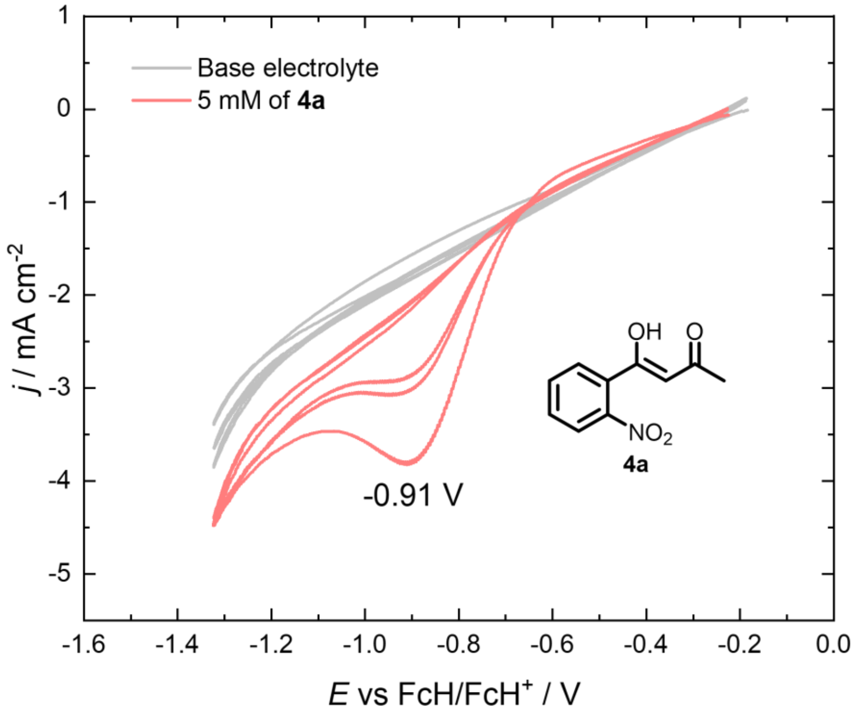

Figure S6: Cyclic voltammogram of **4a** with 0.5 M H_2SO_4 in methanol and water (1:1 (*v*:*v*)).

5. Preparation of Products and Analytical Data

5.1. 1-Hydroxy-1-(2-nitrophenyl)but-3-en-2-ones (4a–aa)

The synthesized (2-nitrophenyl)-1,3-butandiones (**4a–aa**) showed keto-enol tautomerization. Chemical shifts of both tautomers are reported separately. However, in some cases only traces of the keto form were observed by NMR spectroscopy.

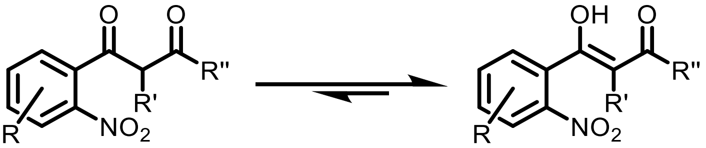

Scheme S5: Keto-enol tautomerization of (2-nitrophenyl)-1,3-butandiones (**4a–aa**).

1-Hydroxy-1-(2-nitrophenyl)but-1-en-3-one (4a)

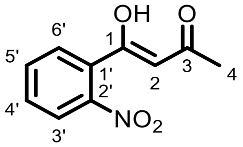

According to general protocol **GPI** 2-nitrobenzoic acid (5.01 g, 30 mmol, 1.0 eq.) was reacted. 5.70 g (27.5 mmol, 92%) of the product was obtained was obtained by column chromatography (80 g silica (irregular); cyclohexane:ethyl acetate 0 → 20% ethyl acetate) as beige solid.

^{1}H NMR (400 MHz, CDCl$_3$, enol form) *δ* [ppm]: 15.20 (bs, 1H, O*H*), 7.92 (dd, *J* = 8.0, 1.3 Hz, 1H, *H*-3'), 7.71–7.45 (m, 3H, *H*-4', *H*-5', *H*-6'), 5.81 (s, 1H, *H*-2), 2.16 (s, 3H, *H*-4).
^{1}H NMR (400 MHz, CDCl$_3$, keto form) *δ* [ppm]: 8.15 (dd, *J* = 8.3, 1.2 Hz, 1H, *H*-3'), 7.76 (ddd, *J* = 7.7, 7.5 1.2 Hz, 1H, *H*-5'), 7.71–7.45 (m, 2H, *H*-4', *H*-6'), 3.98 (s, 2H, *H*-2), 2.33 (s, 3H, *H*-4).
^{13}C NMR (101 MHz, CDCl$_3$, enol form) *δ* [ppm]: 190.0, 187.2, 147.9, 133.1 (2C), 131.3, 129.2, 124.5, 100.1, 24.5.
^{13}C NMR (101 MHz, CDCl$_3$, keto form) *δ* [ppm]: 201.7, 195.9, 145.6, 137.3, 134.9, 131.1, 128.1, 124.5, 57.7, 30.8.
HR-MS (ESI-): *m/z* for [$C_{10}H_9NO_4$-H]$^-$, [M-H]$^-$ calculated: 206.0459; found: 206.0461.
The spectroscopic data are in accordance with those reported in the literature.[4]

3-(Hydroxy(2-nitrophenyl)methylene)pentane-2,4-dione (4c)

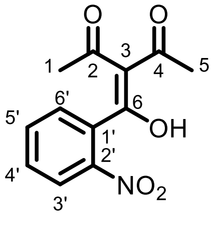

Pyridine (2.4 mL, 2.37 g, 30 mmol, 2 eq.) was added dropwise at 0 °C to a stirred solution of magnesium chloride (1.42 g, 15 mmol, 1.0 eq.) and acetylacetone (1.51, 15.0 mmol, 1.0 eq.) in CH_2Cl_2 (20 mL) followed by 2-nitrobenzoyl chloride (2.78 g, 15 mmol, 1.0 eq.) and stirred for 6 h at room temperature. The reaction mixture was cooled to 0 °C and 3 M HCl (50 mL) was added. The aqueous fraction was extracted twice with ethyl acetate (2x50 mL). The combined organic fractions were washed with brine, dried over anhydrous Na_2SO_4, filtered, and concentrated in vacuo. The crude product was purified by filtration over silica (120 g silica (regular); cyclohexane:ethyl acetate 1:1 (*v*:*v*)). 2.42 g (9.68 mmol, 65%) of the product was obtained by crystallization (30 mL cyclohexane:EtOH 9:1 (*v*:*v*); crystallization at 6 °C) as colorless solid.

^{1}H NMR (400 MHz, DMSO-*d*$_6$, enol form) *δ* [ppm] 8.35–7.99 (m, 1H, *H*-3'), 7.89–7.66 (m, 3H, *H*-4', *H*-5', *H*-6'), 2.13 (s, 6H, *H*-1, *H*-5).
^{13}C NMR (101 MHz, DMSO-*d*$_6$, enol form) *δ* [ppm]: 195.9, 191.2, 147.5, 136.1, 133.9, 132.3, 129.4, 124.8, 116.1, 25.9.
HR-MS (ESI+): *m/z* for $C_{12}H_{11}NO_5$+H$^+$, [M+H]$^+$ calculated: 250.0710; found: 250.0720.
m.p. (MeOH:cyclohexane): 74.2–75.8 °C.

1-Hydroxy-4-methyl-1-(2-nitrophenyl)pent-1-en-3-one (4d)

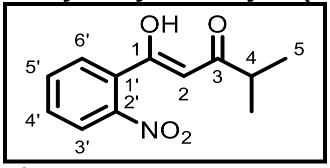

According to general protocol **GPI** 2-nitrobenzoic acid (4.66 g, 25.1 mmol, 1.0 eq.) and ethyl 4-methyl-3-oxopentanoate (3.97 g, 25.1 mmol, 1.0 eq.) were reacted. 4.03 g (17.13 mmol, 68%) of the product was obtained was obtained by column chromatography (80 g silica (irregular); cyclohexane:ethyl acetate 0 → 20% ethyl acetate) as yellow oil.

^{1}H NMR (400 MHz, CDCl$_3$, enol form) *δ* [ppm]: 15.01 (bs, 1H, O*H*), 7.60 (dd, *J* = 8.0, 1.3 Hz, 1H, *H*-3'), 7.41–7.20 (m, 3H, *H*-4', *H*-5', *H*-6'), 5.52 (s, 1H, *H*-2), 2.27 (hept, *J* = 7.0 Hz, 1H, *H*-4), 0.90 (d, *J* = 6.9 Hz, 6H, *H*-5)

^{1}H NMR (400 MHz, CDCl$_3$, keto form) *δ* [ppm]: 7.83 (dd, *J* = 8.3, 1.2 Hz, 1H, *H*-3'), 7.45 (ddd, *J* = 7.7, 7.5 1.2 Hz, 1H, *H*-5'), 7.41–7.18 (m, 2H, *H*-4', *H*-6'), 3.75 (s, 2H, *H*-2), 2.47 (hept, *J* = 7.0 Hz, 1H, *H*-4), 0.82 (d, *J* = 7.0 Hz, 6H, *H*-5).

^{13}C NMR (101 MHz, CDCl, enol form $_3$) *δ* [ppm]: 197.7, 187.3, 148.0, 133.1, 133.0, 131.2, 129.3, 124.4, 97.3, 36.5, 19.4.

^{13}C NMR (101 MHz, CDCl$_3$, keto form) *δ* [ppm]: 207.8, 196.4, 145.3, 137.5, 134.8, 130.9, 128.3, 124.3, 54.6, 41.8, 17.9.

HR-MS (ESI+): *m/z* for $C_{12}H_{13}NO_4+H^+$, $[M+H]^+$ calculated: 236.0917; found: 236.0914.

1-Cyclopropyl-3-hydroxy-3-(2-nitrophenyl)prop-2-en-1-one (4e)

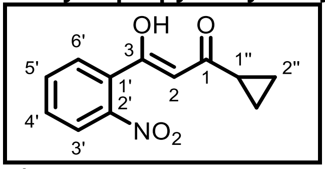

According to general protocol **GPI** 2-nitrobenzoic acid (4.66 g, 25.1 mmol, 1.0 eq.) and ethyl 3-cyclopropyl-3-oxopropanoate (3.92 g, 25.1 mmol, 1.0 eq.) were reacted. 4.26 g (18.27 mmol, 73%) of the product was obtained was obtained by column chromatography (80 g silica (irregular); cyclohexane:ethyl acetate 0 → 20% ethyl acetate) as beige solid.

^{1}H NMR (400 MHz, CDCl$_3$, enol form) *δ* [ppm]: 15.63 (bs, 1H, O*H*), 7.94–7.85 (m, 1H, *H*-3'), 7.68–7.55 (m, 3H, *H*-4', *H*-5', *H*-6'), 5.95 (s, 1H, *H*-2), 1.72 (tt, *J* = 7.9, 4.6 Hz, 1H, *H*-1''), 1.28–1.19 (m, 2H, *H*-2''), 1.13–0.92 (m, 2H, *H*-2'').

^{1}H NMR (400 MHz, CDCl$_3$, keto form) *δ* [ppm]: 8.14 (dd, *J* = 8.3, 1.2 Hz, 1H, *H*-3'), 7.74 (dd, *J* = 7.7, 7.5, 1.1 Hz, 1H, *H*-5'), 7.68–7.55 (m, 1H, *H*-4'), 7.52 (dd, *J* = 7.5, 1.5 Hz, 1H, *H*-6'), 4.11 (s, 2H, *H*-2), 2.12 (tt, *J* = 7.8, 4.6 Hz, 1H, *H*-1''), 1.13–0.92 (m, 4H, *H*-2'').

^{13}C NMR (101 MHz, CDCl$_3$, enol form) *δ* [ppm]: 197.9, 181.2, 148.3, 132.9, 131.9, 131.2, 129.6, 124.4, 99.3, 18.7, 11.2.

^{13}C NMR (101 MHz, CDCl$_3$, keto form) *δ* [ppm]: 203.9, 196.0, 145.4, 137.4, 134.8, 131.0, 128.3, 124.3, 57.6, 21.5, 12.2.

HR-MS (ESI+): *m/z* for $C_{12}H_{11}NO_4+H^+$, $[M+H]^+$ calculated: 234.0761; found: 234.0758.

1-Hydroxy-4,4-dimethyl-1-(2-nitrophenyl)pent-1-en-3-one (4f)

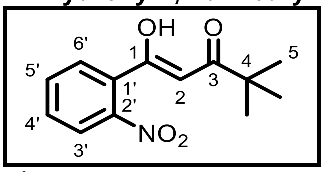

According to general protocol **GPI** 2-nitrobenzoic acid (4.66 g, 25.1 mmol, 1.0 eq.) and ethyl 4,4-dimethyl-3-oxopentanoate (4.32 g, 25.1 mmol, 1.0 eq.) were reacted. 3.31 g (13.28 mmol, 53%) of the product was obtained was obtained by column chromatography (80 g silica (irregular); cyclohexane:ethyl acetate 0 → 20% ethyl acetate) as yellow oil.

^{1}H NMR (400 MHz, CDCl$_3$, enol form) *δ* [ppm]: 15.56 (bs, 1H, O*H*), 7.93–7.86 (m, 1H, *H*-3'), 7.71–7.63 (m, 1H, *H*-5'), 7.62–7.54 (m, 2H, *H*-4', *H*-6'), 5.90 (s, 1H, *H*-2), 1.22 (s, 9H, *H*-5).

^{1}H NMR (400 MHz, CDCl$_3$, keto form) *δ* [ppm]: 8.35–8.00 (m, 1H, *H*-3'), 7.80–7.70 (m, 1H, *H*-5'), 7.71–7.52 (m, 2H, *H*-4', *H*-6'), 4.09 (s, 2H, *H*-2), 1.14 (s, 9H, *H*-5).

^{13}C NMR (101 MHz, CDCl$_3$, enol form) *δ* [ppm]: 200.0, 187.6, 148.0, 133.3, 133.0, 131.2, 129.3, 124.4, 95.5, 39.2, 27.4.

^{13}C NMR (101 MHz, CDCl$_3$, keto form) *δ* [ppm]: 209.7, 197.0, 145.3, 137.7, 134.8, 130.8, 128.7, 124.2, 51.2, 45.2, 26.0.

HR-MS (ESI+): *m/z* for $C_{13}H_{15}NO_4+H^+$, $[M+H]^+$ calculated: 250.1074; found: 250.1073.

4-Hydroxy-4-(2-nitrophenyl)-2-oxo-but-3-enoic acid (4g)

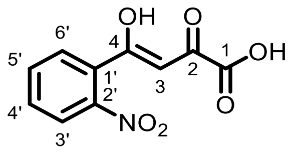

2'-Nitroacetophenone (3.30 g, 20.0 mmol, 1.0 eq.) and diethyl oxalate (3.0 mL, 3.22 g, 22.0 mmol, 1.1 eq.) was stirred at 0 °C in dry THF (20 mL). A sodium ethoxide solution in ethanol (freshly prepared from 0.92 g sodium in 30 mL ethanol) was added and the mixture was stirred for 4 h at 0 °C. After completion of the reaction, the mixture was poured onto ice-cold 2 M HCl (100 mL) The precipitated crude ethyl 4-(2-nitrophenyl)-2,4-dioxobutanoate was collected by filtration, treated with 40 mL 1 M sodium hydroxide solution and stirred for 30 min. After filtration the filtrate was neutralized with concentrated hydrochloric acid and the precipitated crude product was obtained. 3.40 g (14.34 mmol, 72%) of the product was isolated by crystallization (40 mL EtOH; crystallization at -18 °C) as colorless solid.

^{1}H NMR (400 MHz, DMSO-*d*$_6$, enol form) δ [ppm]: 8.16–8.08 (m, 1H, *H*-3'), 7.90–7.80 (m, 1H, *H*-4'), 7.80–7.72 (m, 1H, *H*-5'), 7.73–7.66 (m, 1H, *H*-3'), 6.42 (s, 1H, *H*-3)
^{13}C NMR (101 MHz, DMSO-*d*$_6$, enol form) δ [ppm]: 191.9, 163.8, 161.3, 146.7, 134.8, 134.2, 131.7, 128.9, 124.3, 103.1kk.
HR-MS (ESI+): *m/z* for $C_{10}H_7NO_6+H^+$, $[M+H]^+$ calculated: 238.0346; found: 234.0360.
m.p. (EtOH): 154.3–156.2 °C.
The spectroscopic data are in accordance with those reported in the literature.[5]

3-Hydroxy-3-(2-nitrophenyl)-1-phenylprop-2-en-1-one (4h)

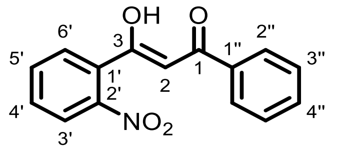

According to general protocol **GPII** 2-nitrobenzoyl chloride (6.68 g, 36.0 mmol, 1.2 eq.) and acetophenone (3.60 g, 30.0 mmol, 1.0 eq.) was reacted. 2.20 g (8.2 mmol, 27%) of the product was obtained was obtained by column chromatography (80 g silica (irregular); cyclohexane:ethyl acetate 10 → 30% ethyl acetate) as beige solid.

^{1}H NMR (400 MHz, CDCl$_3$, enol form) δ [ppm]: 8.03–7.87 (m, 3H, *H*-3', *H*-2''), 7.78–7.51 (m, 4H, *H*-4', *H*-5', *H*-6', *H*-4''), 7.51–7.41 (m, 2H, *H*-3''), 6.47 (s, 1H, *H*-2).
^{13}C NMR (101 MHz, CDCl$_3$, enol form) δ [ppm]: 188.8, 182.8, 147.9, 134.0, 133.6, 133.2, 133.0, 131.3, 129.3, 128.9, 127.3, 124.5, 96.4.
HR-MS (ESI+): *m/z* for $C_{15}H_{11}NO_4+H^+$, $[M+H]^+$ calculated: 270,0761; found: 270.0767.
The spectroscopic data are in accordance with those reported in the literature.[6]

1-Hydroxy-1-(2-nitrophenyl)hex-1-en-3-one (4i)

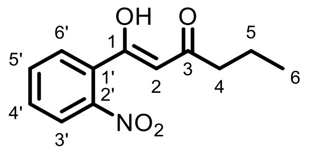

According to general protocol **GPI** 2-nitrobenzoyl chloride (4.66 g, 25.1 mmol, 1.0 eq.) and ethyl 3-oxohexanoate (3.97 g, 25.1 mmol, 1.0 eq.) were reacted. 4.21 g (17.90 mmol, 71%) of the product was obtained was obtained by column chromatography (80 g silica (irregular); cyclohexane:ethyl acetate 0 → 20% ethyl acetate) as beige solid.

^{1}H NMR (400 MHz, CDCl$_3$, enol form) δ [ppm]:15.22 (bs, 1H, O*H*), 7.91 (dd, *J* = 8.1, 1.3 Hz, 1H, *H*-3'), 7.71–7.51 (m, 3H, *H*-4', *H*-5', *H*-5'), 5.79 (s, 1H, *H*-2), 2.35 (d, *J* = 7.5 Hz, 2H, *H*-4), 1.78–1.56 (m, 2H, *H*-5), 0.98 (t, *J* = 7.4 Hz, 3H, *H*-6).
^{1}H NMR (400 MHz, CDCl$_3$, keto form) δ [ppm]: 8.14 (dd, *J* = 8.3, 1.2 Hz, 1H, *H*-3'), 7.75 (ddd, *J* = 7.7, 7.5, 1.2 Hz, 1H, *H*-5'), 7.71–7.44 (m, 2H, *H*-4', *H*-6'), 3.97 (s, 2H, *H*-2), 2.58 (t, *J* = 7.3 Hz, 2H, *H*-4), 1.77–1.48 (m, 2H, *H*-5), 0.91 (t, *J* = 7.4 Hz, 3H, *H*-6).
^{13}C NMR (101 MHz, CDCl$_3$, enol form) δ [ppm]: 193.0, 187.3, 148.0, 133.2, 133.0, 131.2, 129.2, 124.5, 99.4, 39.8, 19.4, 13.8.
^{13}C NMR (101 MHz, CDCl$_3$, keto form) δ [ppm]: 203.9, 196.1, 145.4, 137.4, 134.8, 131.0, 128.2, 124.4, 56.9, 45.6, 16.9, 13.6.
HR-MS (ESI+): *m/z* for $C_{12}H_{13}NO_4+H^+$, $[M+H]^+$ calculated: 236.0917; found: 236.0908.
The spectroscopic data are in accordance with those reported in the literature.[4]

1-Hydroxy-1-(2-nitrophenyl)dec-1-en-3-one (4j)

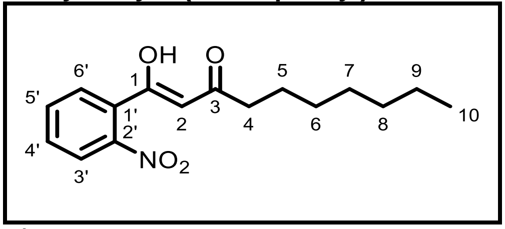

According to general protocol **GPI** 2-nitrobenzoyl chloride (8.34 g, 45 mmol, 1.0 eq.) and ethyl 3-oxodecanoate (9.64 g, 45 mmol, 1.0 eq.) were reacted. 7.25 g (24.9 mmol, 55%) of the product was obtained was obtained by column chromatography (120 g silica (regular); cyclohexane:ethyl acetate 10 → 50% ethyl acetate) as yellow oil.

^{1}H NMR (400 MHz, CDCl$_3$, enol form) δ [ppm]: 15.24 (bs, 1H, O*H*), 7.91 (dd, *J* = 8.0, 1.3 Hz, 1H, *H*-3'), 7.72–7.48 (m, 4H, *H*-4', *H*-5', *H*-6'), 5.79 (s, 1H, *H*-2), 2.47–2.30 (m, 2H, *H*-4), 1.75–1.60 (m, 2H, *H*-5), 1.49–1.17 (m, 8H, *H*-6, *H*-7, *H*-8, *H*-9), 0.94–0.79 (m, 3H, *H*-10).

^{1}H NMR (400 MHz, CDCl$_3$, keto form) δ [ppm]: 8.14 (dd, *J* = 8.3, 1.2 Hz, 1H, *H*-3'), 7.75 (ddd, *J* = 7.7, 7.5, 1.2 Hz, 1H *H*-5'), 7.70 – 7.48 (m, 2H, *H*-4', *H*-6'), 3.97 (s, 2H, *H*-2), 2.59 (t, *J* = 7.4 Hz, 2H, *H*-4), 1.60–1.52 (m, 2H, *H*-5), 1.43–1.17 (m, 8H, , *H*-6, *H*-7, *H*-8, *H*-9), 0.99–0.79 (m, 3H, , *H*-10).

^{13}C NMR (101 MHz, CDCl$_3$, enol form) δ [ppm]: 193.5, 187.1, 148.0, 133.2, 133.0, 131.2, 129.2, 124.5, 99.3, 38.0, 31.8, 29.3, 29.1, 25.9, 22.7, 14.2.

^{13}C NMR (101 MHz, CDCl$_3$, keto form) δ [ppm]: 204.1, 196.1, 145.4, 137.4, 134.8, 131.0, 128.2, 124.4, 56.9, 43.7, 31.7, 29.1, 29.1, 23.5, 22.7, 14.2.

HR-MS (ESI+): *m/z* for $C_{16}H_{21}NO_4+H^+$, $[M+H]^+$ calculated: 292.1543; found: 292.1534.

1-(4-Fluoro-2-nitrophenyl)-1-hydroxybut-1-en-3-one (4k)

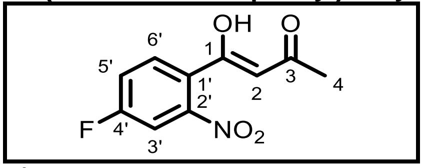

According to general protocol **GPI** 4-fluoro-2-nitrobenzoic acid (3.33 g, 18 mmol, 1.0 eq.) was reacted. 1.78 g (7.88 mmol, 44%) of the product was obtained was obtained by column chromatography (80 g silica (irregular); cyclohexane:ethyl acetate 0 → 20% ethyl acetate) as colourless solid.

^{1}H NMR (400 MHz, CDCl$_3$, enol form) δ [ppm]: 7.65–7.55 (m, 2H, *H*-3', *H*-6'), 7.37 (ddd, *J* = 8.6, 7.4, 2.5 Hz, 1H, *H*-5'), 5.79 (s, 1H, *H*-2), 2.16 (s, 3H, *H*-4).

^{13}C NMR (101 MHz, CDCl$_3$, enol form) δ [ppm]: 190.1, 186.0, 163.0 (d, *J* = 256.1 Hz), 149.0 (d, *J* = 8.4 Hz), 131.2 (d, *J* = 8.6 Hz), 129.2 (d, *J* = 4.0 Hz), 120.1 (d, *J* = 21.4 Hz), 112.6 (d, *J* = 26.8 Hz), 99.9, 24.4.

^{19}F NMR (376 MHz, CDCl$_3$, enol form) δ [ppm]: -106.39 (ddd, *J* = 7.8, 7.4, 5.2 Hz).

HR-MS (ESI-): *m/z* for $[C_{10}H_8FNO_4-H]^-$, $[M-H]^-$ calculated: 224.0365; found: 224.0362.

1-(4-Chloro-2-nitrophenyl)-1-hydroxybut-1-en-3-one (4l)

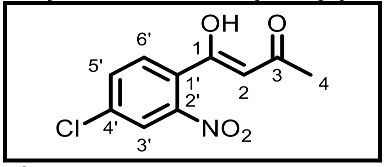

According to general protocol **GPI** 4-chloro-2-nitrobenzoic acid (3.63 g, 18 mmol, 1.0 eq.) was reacted. 7.438 g (8.30 mmol, 46%) of the product was obtained was obtained by column chromatography (80 g silica (irregular); cyclohexane:ethyl acetate 0 → 20% ethyl acetate) as beige solid.

^{1}H NMR (400 MHz, CDCl$_3$, enol form) δ [ppm]: 15.14 (s, 1H, O*H*), 7.89 (d, *J* = 2.0 Hz, 1H, *H*-3'),7.63 (dd, *J* = 8.2, 2.0 Hz, 1H, *H*-5'), 7.51 (d, *J* = 8.2 Hz, 1H, *H*-6'), 5.79 (s, 1H, *H*-2), 2.16 (s, 3H, *H*-4).

^{1}H NMR (400 MHz, CDCl$_3$, keto form) δ [ppm]: 8.12 (d, *J* = 2.0 Hz, 1H, *H*-3'), 7.72 (dd, *J* = 8.2, 2.0 Hz, 1H, *H*-5'), 7.56–7.41 (m, 1H, *H*-6'), 3.97 (s, 2H, *H*-2), 2.31 (s, 3H, *H*-4).

^{13}C NMR (101 MHz, CDCl$_3$, enol form) δ [ppm]: 190.4, 185.8, 148.5, 137.3, 133.0, 131.2, 130.3, 124.8, 99.9, 24.5.

^{13}C NMR (101 MHz, CDCl$_3$, keto form) δ [ppm]: 201.6, 194.8, 146.1, 137.2, 135.4, 134.8, 129.6, 124.6, 57.5, 30.8.

HR-MS (ESI-): *m/z* for $[C_{10}H_8{}^{35}ClNO_4-H]^-$, $[M-H]^-$ calculated: 240.0069; found: 240.0067.

4-(4-Bromo-2-nitrophenyl)-4-hydroxybut-3-en-2-one (4m)

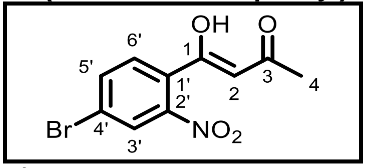

According to general protocol **GPI** 4-bromo-2-nitrobenzoic acid (4.43 g, 18 mmol, 1.0 eq.) was reacted. 3.12 g (10.90 mmol, 61%) of the product was obtained was obtained by column chromatography (80 g silica (irregular); cyclohexane:ethyl acetate 0 → 20% ethyl acetate) as beige solid.

^{1}H NMR (400 MHz, CDCl$_3$, enol form) *δ* [ppm]: 15.14ii (s, 1H, O*H*), 8.04 (d, *J* = 1.9 Hz, 1H, *H*-3'), 7.78 (dd, *J* = 8.2, 1.9 Hz, 1H, *H*-5'),7.44 (d, *J* = 8.2 Hz, *H*-6'), 5.78 (s, 1H, *H*-2), 2.16 (s, 3H, *H*-4).
^{1}H NMR (400 MHz, CDCl$_3$, keto form) *δ* [ppm]: 8.27 (d, *J* = 1.9 Hz, 1H, *H*-3'), 7.87 (dd, *J* = 8.2, 1.9 Hz, 1 *H*-5'), 7.46–7.38 (m, 1H, *H*-6'), 3.97 (s, 2H, *H*-2), 2.31 (s, 3H, *H*-4).
^{13}C NMR (101 MHz, CDCl$_3$, enol form) *δ* [ppm]: 190.4, 185.8, 148.40, 136.0, 131.6, 130.4, 127.5, 124.9, 99.8, 24.5.
^{13}C NMR (101 MHz, CDCl$_3$, keto form) *δ* [ppm]: 201.5, 194.9, 146.0, 137.8, 131.7, 129.6, 127.4, 124.8, 57.5, 30.8.
HR-MS (ESI-): *m/z* for [$C_{10}H_8{}^{79}BrNO_4$-H]$^-$, [M-H]$^-$ calculated: 283.9564; found: 283.9555.

1-Hydroxy-1-(2-nitro-4-(trifluoromethyl)-phenyl)but-1-en-3-one (4n)

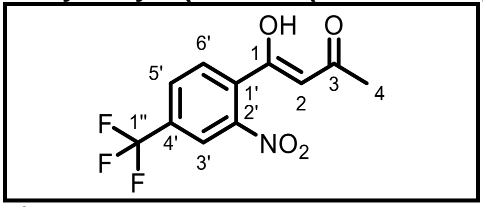

According to general protocol **GPI** 2-nitro-4-(trifluoromethyl)benzoic acid (4.23 g, 18 mmol, 1.0 eq.) was reacted. 1.51 g (5.49 mmol, 31%) of the product was obtained was obtained by column chromatography (80 g silica (irregular); cyclohexane:ethyl acetate 0 → 20% ethyl acetate) as colorless solid.

^{1}H NMR (400 MHz, CDCl$_3$, enol form) *δ* [ppm]: 15.06 (bs, 1H, O*H*), 8.20 (d, *J* = 1.2 Hz, 1H, *H*-3'), 7.94 (dd, *J* = 7.9, 1.2 Hz, 1H, *H*-5'), 7.70 (d, *J* = 7.9 Hz, 1H, *H*-6'), 5.80 (s, 1H, *H*-2), 2.18 (s, 3H, *H*-4).
^{13}C NMR (101 MHz, CDCl$_3$, enol form) *δ* [ppm]: 190.4, 186.0, 147.8, 136.3, 133.5 (q, *J* = 34.5 Hz), 130.1, 129.9 (q, *J* = 3.5 Hz), 122.5 (q, *J* = 273.1 Hz), 121.9 (q, *J* = 3.8 Hz), 100.1, 24.4.
^{19}F NMR (376 MHz, CDCl$_3$, enol form) *δ* [ppm]: -64.24 (s).
HR-MS (ESI+): *m/z* for $C_{11}H_8F_3NO_4$+H$^+$, [M+H]$^+$ calculated: 276.0478; found: 276.0474.

Methyl 4-(1-hydroxy-3-oxobut-1-en-1-yl)-3-nitrobenzoate (4o)

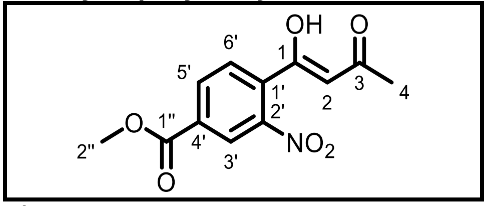

According to general protocol **GPI** 4-(methoxycarbonyl)-2-nitrobenzoic acid (4.05 g, 18 mmol, 1.0 eq.) was reacted. 1.21 g (4.56 mmol, 25%) of the product was obtained was obtained by column chromatography (80 g silica (irregular); cyclohexane:ethyl acetate 0 → 20% ethyl acetate) as beige solid.

^{1}H NMR (400 MHz, CDCl$_3$, enol form) *δ* [ppm]: 15.09 (bs, 1H, O*H*), 8.54 (d, *J* = 1.6 Hz, 1H, *H*-3'), 8.29 (dd, *J* = 7.9, 1.6 Hz, 1H, *H*-5'), 7.62 (d, *J* = 7.9 Hz, 1H, *H*-6'), 5.81 (s, 1H, *H*-2), 3.98 (s, 2H, *H*-2''), 2.17 (s, 3H, *H*-4).
^{1}H NMR (400 MHz, CDCl$_3$, keto form) *δ* [ppm]: n.d.
^{13}C NMR (101 MHz, CDCl$_3$, enol form) *δ* [ppm]: 190.4, 186.2, 164.4, 147.9, 136.5, 133.8, 133.1, 129.5, 125.5, 100.1, 53.1, 24.5.
^{13}C NMR (101 MHz, CDCl$_3$, keto form) *δ* [ppm]: 201.4, 195.3, 164.2, 145.4, 140.7, 135.5, 133.1, 133.0, 128.5, 57.5, 53.2, 30.9.
HR-MS (ESI+): *m/z* for $C_{12}H_{11}NO_6$+H$^-$, [M+H]$^+$ calculated: 266.0659; found: 266.0660.

1-(5-Fluoro-2-nitrophenyl)-1-hydroxybut-1-en-3-one (4p)

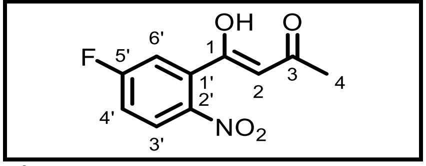

According to general protocol **GPI** 5-fluoro-2-nitrobenzoic acid (3.33 g, 18 mmol, 1.0 eq.) was reacted. 1.69 g (7.52 mmol, 42%) of the product was obtained was obtained by column chromatography (80 g silica (irregular); cyclohexane:ethyl acetate 0 → 20% ethyl acetate) as beige solid.

^{1}H NMR (400 MHz, $CDCl_3$, enol form) *δ* [ppm]: 14.98 (bs, 1H, O*H*), 8.04 (dd, *J* = 8.9, 4.7 Hz, 1H, *H*-3'), 7.35–7.07 (m, 2H, *H*-4', *H*-6'), 5.77 (s, 1H, *H*-2), 2.18 (s, 3H, *H*-4).
^{1}H NMR (400 MHz, $CDCl_3$, keto form) *δ* [ppm]: 8.24 (dd, *J* = 9.1, 4.6 Hz, 1H, *H*-3'), 7.35–7.07 (m, 2H, *H*-4', *H*-6'), 4.01 (s, 2H, *H*-2), 2.34 (s, 3H, *H*-4).
^{13}C NMR (101 MHz, $CDCl_3$, enol form) *δ* [ppm]: (enol-tautomer) 189.5, 186.7, 164.6 (d, *J* = 258.7 Hz), 143.6, 136.3 (d, *J* = 8.0 Hz), 127.4 (d, *J* = 9.5 Hz), 117.8 (d, *J* = 23.2 Hz), 116.5 (d, *J* = 25.0 Hz), 99.9, 24.2.
^{13}C NMR (101 MHz, $CDCl_3$, keto form) *δ* [ppm]: 201.6, 194.4, 165.8 (d, *J* = 265.2 Hz), 141.2, 140.4, 127.5 (d, *J* = 9.4 Hzj), 117.8 (d, *J* = 25.9 Hz), 115.7 (d, *J* = 25.5 Hz), 57.5, 30.8.
^{19}F NMR (376 MHz, $CDCl_3$, enol form) *δ* [ppm]: -103.68 (ddd, *J* = 7.8, 7.7, 4.7 Hz).
^{19}F NMR (376 MHz, $CDCl_3$, keto form) *δ* [ppm]: -100.34 (ddd, *J* = 7.5, 7.4, 4.4 Hz).
HR-MS (ESI+): *m/z* for $C_{10}H_8FNO_4$+H^+, $[M+H]^+$ calculated: 226.0510; found: 226.0520.

1-(5-Chloro-2-nitrophenyl)-1-hydroxybut-1-en-3-one (4q)

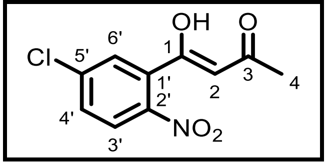

According to general protocol **GPI** 5-chloro-2-nitrobenzoic acid (3.63 g, 18 mmol, 1.0 eq.) was reacted. 7.438 g (9.84 mmol, 55%) of the product was obtained was obtained by column chromatography (80 g silica (irregular); cyclohexane:ethyl acetate 0 → 20% ethyl acetate) as beige solid.

^{1}H NMR (400 MHz, $CDCl_3$, enol form) *δ* [ppm]: 15.02 (bs, 1H, O*H*), 7.92 (d, *J* = 8.6 Hz, 1H, *H*-3'), 7.55 (dd, *J* = 8.6, 2.3 Hz, 1H, *H*-4'), 7.51 (d, *J* = 2.3 Hz, 1H, *H*-6'), 5.76 (s, 1H, *H*-2), 2.16 (s, 3H, *H*-4).
^{13}C NMR (101 MHz, $CDCl_3$, enol form) *δ* [ppm]: 189.8, 186.4, 145.9, 139.8, 135.0, 131.0, 129.3, 126.1, 100.0, 24.3.
HR-MS (ESI-): *m/z* for $[C_{10}H_8{}^{35}ClNO_4-H]^-$, $[M-H]^-$ calculated: 240.0069; found: 240.0061.

4-Hydroxy-4-(5-methoxy-2-nitrophenyl)but-3-en-2-one (4r)

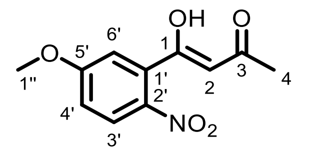

According to general protocol **GPI** 5-methoxy-2-nitrobenzoic acid (7.10 g, 36 mmol, 1.0 eq.) was reacted. 5.13 g (21.63 mmol, 60%) of the product was obtained was obtained by column chromatography (80 g silica (irregular); cyclohexane:ethyl acetate 0 → 20% ethyl acetate) as off-white solid.

^{1}H NMR (400 MHz, $CDCl_3$, enol form) *δ* [ppm]: 14.98 (bs, 1H, O*H*), 8.02 (d, *J* = 9.0 Hz, 1H, *H*-3'), 6.99 (dd, *J* = 9.0, 2.8 Hz, 1H, *H*-4'), 6.91 (d, *J* = 2.8 Hz, H, *H*-6'), 5.69 (s, 1H, *H*-2), 3.91 (s, 3H, *H*-1''), 2.13 (s, 3H, *H*-4).
^{1}H NMR (400 MHz, $CDCl_3$, keto form) *δ* [ppm]: 8.15 (d, *J* = 9.2 Hz, 1H, *H*-3'), 7.02 (dd, *J* = 9.2, 2.8 Hz, 1H, *H*-4'), 6.88 (d, *J* = 2.8 Hz, 1H, *H*-6'), 3.95 (s, 2H, *H*-2), 3.92 (s, 3H, *H*-1''), 2.32 (s, 3H, *H*-4).
^{13}C NMR (101 MHz, $CDCl_3$, enol form) *δ* [ppm]: 189.2, 188.2, 163.5, 140.0, 136.4, 127.2, 115.2, 114.2, 100.2, 56.3, 24.0.
^{13}C NMR (101 MHz, $CDCl_3$, keto form) *δ* [ppm]: 201.9, 195.9, 164.8, 140.3, 137.9, 127.1, 115.8, 112.5, 57.7, 56.5, 30.7.
HR-MS (ESI-): *m/z* for $[C_{11}H_{11}NO_5-H]^-$, $[M-H]^-$ calculated: 236.0564; found: 236.0565.

1-(6-Fluoro-2-nitrophenyl)-1-hydroxybut-1-en-3-one (4s)

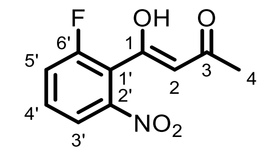

According to general protocol **GPI** 6-fluoro-2-nitrobenzoic acid (3.33 g, 18 mmol, 1.0 eq.) was reacted. 1.61 g (7.15 mmol, 40%) of the product was obtained was obtained by column chromatography (80 g silica (irregular); cyclohexane:ethyl acetate 0 → 20% ethyl acetate) as beige solid.

^{1}H NMR (400 MHz, CDCl$_3$, enol form) δ [ppm]: 14.82 (bs, 1H, O*H*), 7.84 (ddd, *J* = 8.0, 1.2, 0.9 Hz, 1H, *H*-3'), 7.65–7.46 (m, 1H, *H*-5'), 7.43 (ddd, *J* = 8.7, 8.0, 1.6 Hz, 1H, *H*-4'), 5.79 (d, *J* = 1.5 Hz, 1H, *H*-2), 2.15 (s, 3H, *H*-4).
^{1}H NMR (400 MHz, CDCl$_3$, keto form) δ [ppm]: 8.04–7.99 (m, 1H, *H*-3'), 7.71–7.47 (m, 2H, *H*-4', *H*-5'), 4.00 (s, 2H, *H*-2), 2.39 (s, 3H, *H*-4).
^{13}C NMR (101 MHz, CDCl$_3$, enol form) δ [ppm]: 187.9, 184.2, 159.1 (d, *J* = 253.7 Hz), 148.0, 131.5 (d, *J* = 8.5 Hz), 131.5 (d, *J* = 9.0 Hz), 121.6 (d, *J* = 22.8 Hz), 120.4 (d, *J* = 3.5 Hz), 102.3 (d, *J* = 2.5 Hz), 23.8.
^{13}C NMR (101 MHz, CDCl$_3$, keto form) δ [ppm]: 200.5, 191.6, 158.5 (d, *J* = 251.6 Hz), 146.0, 125.5 (d, *J* = 23.2 Hz), 122.7 (d, *J* = 3.7 Hz), 122.5 (d, *J* = 2.9 Hz), 120.5 (d, *J* = 3.3 Hz), 58.7, 30.9.
^{19}F NMR (376 MHz, CDCl$_3$, enol form) δ [ppm]: -112.47 (dd, *J* = 8.6, 5.5 Hz)
^{19}F NMR (376 MHz, CDCl$_3$, keto form) δ [ppm]: -114.79 (dd, *J* = 8.3, 5.5 Hz).
HR-MS (ESI-): *m/z* for [$C_{10}H_8FNO_4$-H]$^-$, [M-H]$^-$ calculated: 224.0365; found: 224.0367.

1-(4,5-Difluoro-2-nitrophenyl)-1-hydroxybut-1-en-3-one (4t)

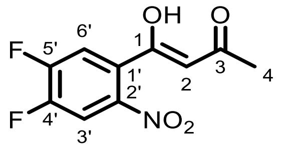

According to general protocol **GPI** 4,5-difluoro-2-nitrobenzoic acid (7.24 g, 36 mmol, 1.0 eq.) was reacted. 3.02g (12.43 mmol, 35%) of the product was obtained was obtained by column chromatography (80 g silica (irregular); cyclohexane:ethyl acetate 0 → 20% ethyl acetate) as beige solid.

^{1}H NMR (400 MHz, CDCl$_3$, enol form) δ [ppm]: 14.99 (s, 1H, O*H*), 7.85 (dd, *J* = 9.2, 6.7 Hz, 1H, *H*-3'), 7.45 – 7.33 (m, 1H, *H*-6'), 5.73 (s, 1H), 2.16 (s, 3H).
^{1}H NMR (400 MHz, CDCl$_3$, keto form) δ [ppm]: 8.04 (dd, *J* = 9.4, 6.6 Hz, 1H, *H*-3'), 7.47–7.32 (m, 1H, *H*-6'), 3.99 (s, 2H, *H*-2), 2.30 (s, 3H, *H*-4).
^{13}C NMR (101 MHz, CDCl$_3$, enol form) δ [ppm]: 189.7, 185.7, 152.9 (dd, *J* = 231.5, 12.9 Hz), 150.3 (dd, *J* = 230.1, 11.8 Hz), 143.4, 131.1 (dd, *J* = 5.9, 4.3 Hz), 118.4 (dd, *J* = 20.0, 1.9 Hz), 115.2 (dd, *J* = 22.2, 2.0 Hz), 99.9, 24.2.
^{13}C NMR (101 MHz, CDCl$_3$, keto form) δ [ppm]: 201.6, 193.5, 154.1 (dd, *J* = 264.1, 12.8 Hz), 150.4 (dd, *J* = 258.6, 13.9 Hz), 141.1 135.1 (dd, *J* = 6.0, 4.1 Hz), 117.7 (dd, *J* = 21.1, 2.1 Hz), 114.7 (dd, *J* = 22.0, 2.3 Hz), 57.5, 30.8.
^{19}F NMR (376 MHz, CDCl$_3$, enol form) δ [ppm]: -127.76 (ddd, *J* = 21.0, 9.3, 6.5 Hz), -130.46 (ddd, *J* = 20.6, 9.4, 7.5 Hz)
^{19}F NMR (376 MHz, CDCl$_3$, keto form) δ [ppm]: -124.32 (ddd, *J* = 20.9, 9.1, 6.7 Hz), -130.33 (ddd, *J* = 20.5, 9.6, 7.6 Hz).
HR-MS (ESI-): *m/z* for [$C_{10}H_7F_2NO_4$-H]$^-$, [M-H]$^-$ calculated: 242.0270; found: 242.0271.

4-(3-Chloro-2-nitrophenyl)-4-hydroxybut-3-en-2-one (4u)

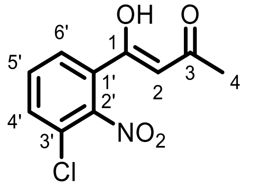

According to general protocol **GPI** 3-chloro-2-nitrobenzoic acid (3.63 g, 18 mmol, 1.0 eq.) was reacted. 1.26 g (5.22 mmol, 29%) of the product was obtained was obtained by column chromatography (80 g silica (irregular); cyclohexane:ethyl acetate 0 → 20% ethyl acetate) as beige solid.

^{1}H NMR (400 MHz, CDCl$_3$, enol form) δ [ppm]: 15.40 (s, 1H, O*H*), 7.69–7.57 (m, 2H, *H*-4', *H*-6'), 7.51 (dd, *J* = 8.0, 7.8 Hz, 1H, *H*-5'), 5.96 (s, 1H, *H*-2), 2.18 (s, 3H, *H*-4).
^{13}C NMR (101 MHz, CDCl$_3$, enol form) δ [ppm]: 191.9, 183.0, 147.7, 133.5, 131.3, 131.0, 127.4, 126.8, 99.0, 24.9.
HR-MS (ESI-): *m/z* for [$C_{10}H_8{}^{35}ClNO_4$-H]$^-$, [M-H]$^-$ calculated: 240.0069; found: 240.0072.

1-Hydroxy-2-methyl-1-(2-nitrophenyl)pent-1-en-3-one (4v)

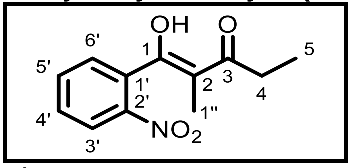

According to general protocol **GPII** 2-nitrobenzoyl chloride (2.78 g, 15.0 mmol, 1.2 eq.) and 3-pentanone (1.08 g, 12.5 mmol, 1.0 eq.) was reacted. 1.20 g (5.11 mmol, 41%) of the product was obtained was obtained by column chromatography (80 g silica (irregular); cyclohexane:ethyl acetate 0 → 20% ethyl acetate) as beige solid.

^{1}H NMR (400 MHz, $CDCl_3$, enol form) *δ* [ppm]: 8.20–8.07 (m, 1H; *H*-3'), 7.78–7.66 (m, 1H, *H*-5'), 7.65–7.53 (m, 1H, *H*-4'), 7.45–7.35 (m, 2H, *H*-6'), 2.70–2.45 (m, 2H, *H*-4), 1.64 (s, 3H, *H*-1''), 1.18 (t, *J* = 7.4 Hz, 2H, *H*-5).

^{1}H NMR (400 MHz, $CDCl_3$, keto form) *δ* [ppm]: 8.20–8.07 (m, 1H; *H*-3'), 7.78–7.66 (m, 1H, *H*-5'), 7.65–7.53 (m, 1H, *H*-4'), 7.45–7.35 (m, 2H, *H*-6'), 4.09 (q, *J* = 7.1 Hz, 1H, *H*-2), 2.70 – 2.45 (m, 4H, *H*-4), 1.46 (d, *J* = 7.1 Hz, 3H, *H*-3), 1.01 (t, *J* = 7.2 Hz, 3H, *H*-5).

^{13}C NMR (101 MHz, $CDCl_3$, enol form) *δ* [ppm]: 196.6, 184.1, 146.4, 134.0, 132.9, 130.2, 129.2, 124.6, 104.6, 29.9, 12.9, 8.9.

^{13}C NMR (101 MHz, $CDCl_3$, keto form) *δ* [ppm]: 207.8, 199.4, 145.4, 137.1, 134.7, 130.8, 128.5, 124.5, 60.1, 35.0, 13.8, 7.6.

HR-MS (ESI+): *m/z* for $C_{12}H_{13}NO_4+H^+$, $[M+H]^+$ calculated: 236.0917; found: 236.0913.

The spectroscopic data are in accordance with those reported in the literature.[4]

2-((*E*,*E*)-Farnesyl)-1-hydroxy-1-(2-nitrophenyl)but-1-en-3-one (4x)

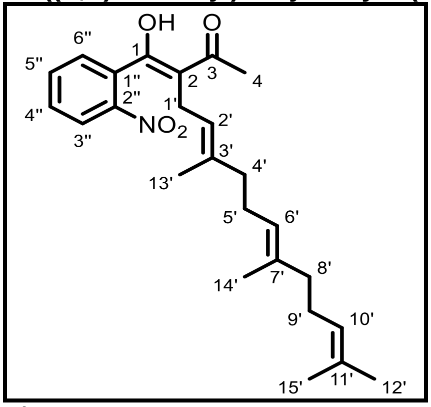

To a suspension of **4a** (1.04 g, 5.00 mmol, 1.0 eq.), potassium carbonate (760 mg, 5.5 mmol, 1.1 eq.) and potassium iodide (83 mg, 0.5 mmol, 0.1 eq.) in 25 mL DMF at 0 °C was added (*E*,*E*)-farnesyl bromide (1.57 g, 5.5 mmol, 1.1 eq.). The mixture was stirred at room temperature for 6 h. 1 M HCl (50 mL) and ethyl acetate (50 mL) were added, and the aqueous phase was extracted twice with ethyl acetate (2x50 mL). The combined organic fractions were washed with water, brine, dried over anhydrous Na_2SO_4, filtered, and concentrated in vacuo. 627 mg (1.53 mmol, 31%) of the product was obtained by column chromatography (80 g silica (regular); cyclohexane:ethyl acetate 0 → 5% ethyl acetate) as yellowish oil.

^{1}H NMR (400 MHz, $CDCl_3$, enol form) *δ* [ppm]: 16.13 (s, 1H, O*H*), 8.18–8.10 (m, 1H, *H*-3''), 7.76–7.52 (m, 2H, *H*-4'', *H*-5''), 7.40–7.32 (m, 1H, *H*-6''), 5.12–5.00 (m, 2H, , *H*-6', *H*-10'), 4.05 (dd, *J* = 8.4, 6.1 Hz, 1H, *H*-2'), 2.78–2.60 (m, 2H, *H*-1'), 2.24 (s, 3H, *H*-4), 2.09–1.85 (m, 8H, *H*-4', *H*-5', *H*-8', *H*-9'), 1.69–1.66 (m, 12H, *H*-12', *H*-13', *H*-14', *H*-15').

^{1}H NMR (400 MHz, $CDCl_3$, keto form) *δ* [ppm]: 8.18–8.10 (m, 1H, *H*-3''), 7.76–7.52 (m, 2H, *H*-4'', *H*-5''), 7.40–7.32 (m, 1H, *H*-6''), 5.12–5.00 (m, 3H, *H*-2, *H*-6', *H*-10'), 4.89 (td, *J* = 6.4, 1.3 Hz, 1H, *H*-2'), 2.78–2.60 (m, 2H, *H*-1'), 2.20 (s, 3H, *H*-4), 2.09–1.85 (m, 8H, *H*-4', *H*-5', *H*-8', *H*-9'), 1.69–1.66 (m, 12H, *H*-12', *H*-14', *H*-15'), 1.24 (d, *J* = 1.3 Hz, 3H, *H*-13').

^{13}C NMR (101 MHz, $CDCl_3$, keto & enol form) *δ* [ppm]: 204.2, 198.1, 190.8, 188.7, 146.0, 145.6, 139.1, 137.0, 136.2, 135.4, 135.4, 134.5, 133.9, 133.5, 131.5, 131.4, 131.0, 130.0, 128.9, 128.4, 124.6, 124.6, 124.4, 124.4, 124.0, 123.9, 122.4, 119.4, 109.6, 67.1, 39.8, 39.8, 39.8, 39.6, 29.7, 28.2, 26.8, 26.8, 26.8, 26.7, 26.7, 26.5, 26.4, 25.8, 22.9, 17.8, 16.3, 16.1, 16.1, 15.8.

HR-MS (ESI+): *m/z* for $C_{25}H_{33}NO_4+Na^+$, $[M+Na]^+$ calculated: 434.2302; found: 434.2290.

The spectroscopic data are in accordance with those reported in the literature.[7]

Methyl 2-(hydroxy(2-nitrophenyl)methylene)-3-oxobutanoate (4y)

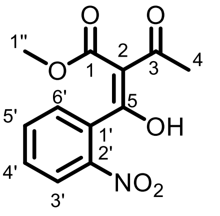

Pyridine (2.4 mL, 2.37 g, 30 mmol, 2 eq.) was added dropwise at 0 °C to a stirred solution of magnesium chloride (1.42 g, 15 mmol, 1.0 eq.) and methyl acetoacetate (1.74, 15.0 mmol, 1.0 eq.) in CH_2Cl_2 (20 mL) followed by 2-nitrobenzoyl chloride (2.78 g, 15 mmol, 1.0 eq.) and stirred for 6 h at room temperature. The reaction mixture was cooled to 0 °C and 3 M HCl (50 mL) was added. The aqueous fraction was extracted twice with ethyl acetate (2x50 mL). The combined organic fractions were washed with brine, dried over anhydrous Na_2SO_4, filtered, and concentrated in vacuo. The crude product was purified by filtration over silica (120 g silica (irregular); cyclohexane:ethyl acetate 1:1 (*v*:*v*)). 2.63 g (10.01 mmol, 67%) of the product was obtained by crystallization (30 mL cyclohexane:MeOH 19:1 (*v*:*v*); crystallization at 6 °C) as colorless solid.

^{1}H NMR (400 MHz, DMSO-*d*$_6$, enol form) *δ* [ppm]: *δ* [ppm]: 8.21 (dd, *J* = 8.3, 1.3 Hz, 1H, *H*-3'), 7.85 (ddd, *J* = 7.6, 7.5, 1.3 Hz, 1H, *H*-5'), 7.79 (ddd, *J* = 8.3, 7.6, 1.5 Hz, 1H, *H*-4'), 7.52 (dd, *J* = 7.5, 1.5 Hz, 1H, *H*-6'), 3.38 (s, 3H, *H*-1''), 2.45 (s, 3H, *H*-4).

^{13}C NMR (101 MHz, DMSO-*d*$_6$, enol form) *δ* [ppm]: 193.5, 192.7, 165.9, 145.3, 134.5, 130.8, 127.6, 124.2, 124.0, 107.6, 51.5, 24.4.

HR-MS (ESI+): *m/z* for $C_{12}H_{11}NO_6$+H^+, $[M+H]^+$ calculated: 266.0659; found: 266.0674.

m.p. (MeOH): 90.2–92.5 °C.

1-Hydroxy-1-(2-nitrophenyl)-2-(4-(trifluoromethoxy)benzyl)but-1-en-3-one (4z)

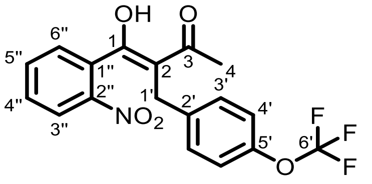

To a suspension of **4a** (2.07 g, 10.00 mmol, 1.5 eq.), potassium carbonate (2.07 mg, 15.0 mmol, 2.3 eq.) and potassium iodide (166 mg, 1.0 mmol, 0.15 eq.) in 15 mL DMF at 0 °C was added 4-(trifluoromethoxy)benzyl bromide (1.66 g, 6.5 mmol, 1.0 eq.). The mixture was stirred at room temperature for 4 h. 1 M HCl (50 mL) and ethyl acetate (50 mL) were added and the aqueous phase was extracted twice with ethyl acetate (2x50 mL). The combined organic fractions were washed with water, brine, dried over anhydrous Na_2SO_4, filtered, and concentrated in vacuo. 965 mg of crude product was obtained by column chromatography (80 g silica (regular); cyclohexane:ethyl acetate 5 → 20% ethyl acetate). 760 mg (1.99 mmol, 31%) of the product was obtained by reversed phase column chromatography (C 18 silica gel, gradient: 55% acetonitrile → 65% acetonitrile) as yellowish oil.

^{1}H NMR (400 MHz, $CDCl_3$, enol form) *δ* [ppm]: 8.16–8.05 (m, 1H, *H*-3''), 7.75–7.42 (m, 2H, *H*-4'', *H*-5''), 7.23–6.89 (m, 5H, *H*-3', *H*-4', *H*-6''), 3.47 (s, 2H, *H*-1'), 2.13 (s, 3H, *H*-4).

^{1}H NMR (400 MHz, $CDCl_3$, keto form) *δ* [ppm]: 8.16–8.05 (m, 1H, *H*-3''), 7.75–7.42 (m, 2H, *H*-4'', *H*-5''), 7.23–6.89 (m, 5H, *H*-3', *H*-4', *H*-6''), 4.38 (t, *J* = 7.5 Hz, 1H, *H*-2), 3.30 (d, *J* = 7.5 Hz, 2H, *H*-1'), 2.11 (s, 3H, *H*-4).

^{13}C NMR (101 MHz, $CDCl_3$, keto & enol form) *δ* [ppm]: 203.0, 197.0, 191.4, 189.5, 148.2, 147.8, 145.9, 145.6, 138.3, 136.7, 136.6, 134.7, 133.9, 133.0, 131.3, 130.4, 130.4, 128.9, 128.5, 128.2, 124.8, 124.7, 121.4, 121.2, 120.5 (q, 120.5 Hz, 2C (keto & enol form)) 108.3, 68.7, 34.7, 32.9, 30.3, 23.3.

^{19}F NMR (376 MHz, $CDCl_3$, enol form) *δ* [ppm]: -59.14.

^{19}F NMR (376 MHz, $CDCl_3$, keto form) *δ* [ppm]: -59.10,

HR-MS (ESI+): *m/z* for $C_{18}H_{14}F_3NO_5$+H^+, $[M+H]^+$ calculated: 382.0897; found: 382.0896.

1-Hydroxy-1-(5-chloro-2-nitrophenyl)dec-1-en-3-one (4aa)

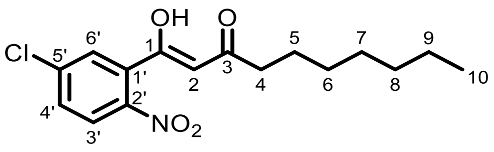

According to general protocol **GPI** 5-chloro-2-nitrobenzoic acid (5.04 g, 25 mmol, 1.0 eq.) and oxalyl chloride (3.2 mL, 4.8 g, 37.5 mmol, 1.5 eq.) were reacted to obtain 5-chloro-2-nitrobenzoyl chloride. Pyridine (4.0 mL, 3.96 g, 50 mmol, 2 eq.) was added dropwise at 0 °C to a stirred solution of magnesium chloride (2.38 g, 25 mmol, 1.0 eq.) and ethyl 3-oxodecanoate (5.36 g, 25 mmol, 1.0 eq.) in CH_2Cl_2 (50 mL) followed by the previously synthesized 5-chloro-2-nitrobenzoyl chloride in CH_2Cl_2 (10 mL) and stirred for 6 h at room temperature. The reaction mixture was cooled to 0 °C and 3 M HCl (100 mL) was added. The aqueous fraction was extracted twice with ethyl acetate (2x100 mL). The combined organic fractions were washed with brine, dried over anhydrous Na_2SO_4, filtered, and concentrated in vacuo. The crude was dissolved in 200 mL of water, *para*-toluenesulfonic acid monohydrate (0.476 g, 2.5 mmol, 0.1 eq.) was added, and the mixture was heated under reflux for 24 h. The aqueous phase was then extracted twice with 100 mL ethyl acetate. The combined organic fractions were washed with water, brine, dried over anhydrous sodium sulfate and the solvent was removed under reduced pressure. 2.71 g (8.3 mmol, 33%) of the product was obtained was obtained by column chromatography (80 g silica (regular); cyclohexane:ethyl acetate 5 → 25% ethyl acetate) as colourless solid.

^{1}H NMR (400 MHz, $CDCl_3$, enol form) δ [ppm]: 15.07 (s, 1H, O*H*), 7.91 (d, *J* = 8.6 Hz, 1H, *H*-3'), 7.60–7.48 (m, 2H, *H*-4', *H*-6'), 5.74 (s, 1H, *H*-2), 2.41–2.32 (m, 2H, *H*-4), 1.72–1.54 (m, 2H, *H*-5), 1.42–1.20 (m, 8H, *H*-6, *H*-7, *H*-8, *H*-9), 0.88 (t, *J* = 6.5 Hz, 3H, *H*-10).
^{13}C NMR (101 MHz, $CDCl_3$, enol form) δ [ppm]: 193.3, 186.4, 145.9, 139.7, 135.1, 131.0, 129.3, 126.0, 99.3, 37.8, 31.8, 29.3, 29.1, 26.0, 22.7, 14.2.
HR-MS (ESI+): *m/z* for $C_{16}H_{20}{}^{35}ClNO_4+H^+$, $[M+H]^+$ calculated: 326.1154; found: 326.1149.

5.2. 1*H*-1-Hydroxyquinol-4-ones (1a, 2 ,5a–aa)

The synthesized 1*H*-1-hydroxyquinol-4-ones (**1a, 2, 5a–aa**) show poor solubility after purification in common organic solvents. Therefore, the NMR spectra could only be measured in low concentrations in deuterium oxide with sodium deuteroxide or dimelthylsulfoxide-d_6.

Aurachine C (1a)

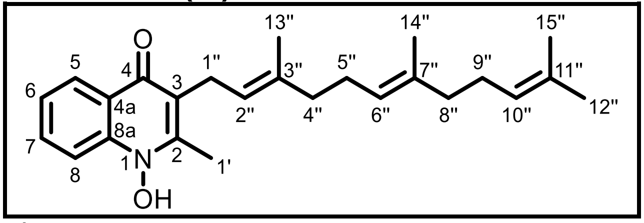

According to general protocol **GPIII 4x** (205.8 mg, 0.50 mmol) were reacted. 156.3 mg (0.41 mmol, 82%) of the product were obtained as colorless solid by reversed phase column chromatography (C 18 silica gel, gradient: 70% acetonitrile → 90% acetonitrile).

^{1}H NMR (400 MHz, DMSO-d_6) δ [ppm]: 11.59 (bs, 1H, O*H*), 8.13 (dd, *J* = 8.1, 1.5 Hz, 1H, *H*-5), 7.79 (dd, *J* = 8.5, 1.1 Hz, 1H, *H*-8), 7.68 (ddd, *J* = 8.5, 6.9, 1.5 Hz, 1H, *H*-7), 7.32 (ddd, *J* = 8.1, 6.9, 1.1 Hz, 1H, *H*-6), 5.08 – 4.96 (m, 3H, *H*-2'', *H*-6'', *H*-10''), 3.31 (d, *J* = 6.9 Hz, 2H, *H*-1''), 2.43 (s, 3H, , *H*-1'), 2.07–1.81 (m, 8H, *H*-4'', *H*-5'', *H*-8'', *H*-9''), 1.73 (d, *J* = 1.4 Hz, 3H, *H*-13''), 1.62–1.42 (m, 9H *H*-12'' *H*-14'' *H*-15'').
^{13}C NMR (101 MHz, DMSO-d_6) δ [ppm]: 173.2, 147.6, 139.5, 134.3, 134.1, 131.5, 130.6, 125.4, 124.1, 123.8, 123.3, 122.9, 122.8, 117.8, 114.4, 39.2, 39.2, 26.2, 26.0, 25.5, 24.0, 17.5, 16.0, 15.8, 14.5.
HR-MS (ESI+): *m/z* for $C_{25}H_{33}NO_2$+H^+, $[M+H]^+$ calculated: 380.2584; found: 380.2575.
The spectroscopic data are in accordance with those reported in the literature.[7]

1*H*-2-Heptyl-1-hydroxyquinol-4-one (2)

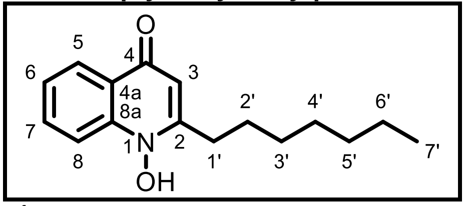

According to general protocol **GPIII 4j** (145.7 mg, 0.50 mmol) were reacted. 106.7mg (0.41 mmol, 82%) of the product were obtained as colorless solid by reversed phase column chromatography (C 18 silica gel, gradient: 40% acetonitrile → 55% acetonitrile).

^{1}H NMR (600 MHz, DMSO-d_6) δ [ppm]: 11.79 (bs, 1H, O*H*), 8.08 (dd, J = 8.1, 1.7 Hz, 1H, *H*-5), 7.85 (dd, *J* = 8.6 Hz, 1.5 Hz, 1H, *H*-8), 7.72 (ddd, *J* = 8.6, 7.1, 1.7 Hz, 1H, *H*-7), 7.36 (ddd, *J* = 8.1, 7.1, 1.5 Hz, 1H, *H*-6), 5.96 (s, 1H, *H*-3), 2.73 (t, *J* = 7.7 Hz, 2H, *H*-1'), 1.66 (tt, *J* = 7.7, 7.1 Hz, 2H, *H*-2'), 1.47–1.15 (m, 8H, *H*-3', *H*-4', *H*-5', *H*-6'), 0.85 (t, *J* = 6.9 Hz, 3H, *H*-7').
^{13}C NMR (151 MHz, DMSO-d_6) δ [ppm]: 173.9, 153.5, 140.5, 132.0, 125.0, 124.8, 123.4, 115.0, 106.7, 31.2, 30.9, 28.7, 28.5, 27.4, 22.1, 14.0.
HR-MS (ESI+): *m/z* for $C_{16}H_{21}NO_2$+H^+, $[M+H]^+$ calculated: 260.1645; found: 260.1634.
The spectroscopic data are in accordance with those reported in the literature.[8]

1*H*-1-Hydroxy-2-methylquinol-4-one (5a)

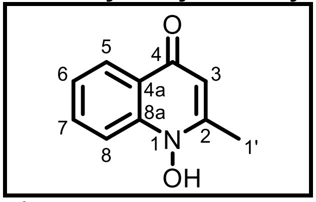

According to general protocol **GPIII 4a** (207.2 mg, 1.00 mmol) were reacted. 160.7 mg (0.92 mmol, 92%) of the product were obtained as colorless solid by reversed phase column chromatography (C 18 silica gel, gradient: 10% acetonitrile → 55% acetonitrile).

^{1}H NMR (400 MHz, DMSO-d_6) δ [ppm]: 8.36–8.19 (m, 2H, *H*-5, *H*-8), 8.08 (ddd, *J* = 8.6, 7.0, 1.4 Hz, 1H, *H*-7), 7.78 (ddd, *J* = 8.3, 7.0, 1.1 Hz, 1H. *H*-6), 7.18 (s, 1H, *H*-3), 2.79 (s, 3H, *H*-1').
^{13}C NMR (101 MHz, DMSO-d_6) δ [ppm]: 166.2, 155.7, 139.4, 134.56, 127.4, 123.8, 120.7, 116.7, 105.8, 18.9.
HR-MS (ESI+): *m/z* for $C_{10}H_9NO_2$+H^+, $[M+H]^+$ calculated: 176.0706; found: 176.0699.
m.p. (MeOH): 244.4–247.6 °C.
The analytical data are in accordance with those reported in the literature.[9]

1*H*-3-Acetyl-1-hydroxy-2-methylquinol-4-one (5c)

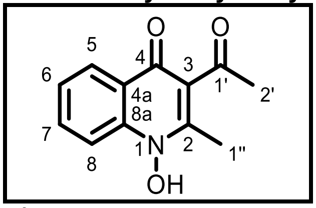

According to general protocol **GPIII 4c** (124.6 mg, 0.50 mmol) were reacted. 69.5 mg (0.32 mmol, 64%) of the product were obtained as colorless solid by reversed phase column chromatography (C 18 silica gel, gradient: 20% acetonitrile → 40% acetonitrile).

^{1}H NMR (600 MHz, DMSO-*d_6*) δ [ppm]: 12.08 (bs, 1H, O*H*), 8.17 (dd, *J* = 8.0, 1.5 Hz, 1H, *H*-5), 7.86 (dd, , *J* = 8.5, 1.1 Hz, 1H, *H*-8), 7.79 (ddd, *J* = 8.5, 7.0, 1.5 Hz, 1H, *H*-7), 7.45 (ddd, *J* = 8.0, 7.0, 1.1 Hz, 1H, *H*-6), 2.51 (s, 3H, *H*-2'), 2.46 (s, 3H, *H*-1').
^{13}C NMR (151 MHz, DMSO-*d_6*) δ [ppm]: 202.3, 173.1, 150.7, 139.7, 132.94, 125.41, 125.3, 124.5, 120.2, 115.2, 32.0, 15.2.
HR-MS (ESI+): *m/z* for $C_{12}H_{11}NO_3$+H$^+$, [M+H]+ calculated: 218.0812; found: 218.0815.

1*H*-1-Hydroxy-2-isopropylquinol-4-one (5d)

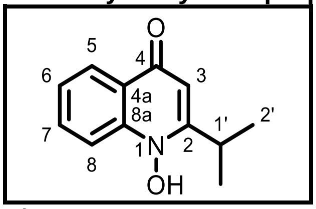

According to general protocol **GPIII 4d** (117.6 mg, 0.50 mmol) were reacted. 85.2 mg (0.42 mmol, 84%) of the product were obtained as colorless solid by reversed phase column chromatography (C 18 silica gel, gradient: 20% acetonitrile → 80% acetonitrile).

^{1}H NMR (400 MHz, D_2O+ 5% NaOD) δ [ppm]: 8.36–8.17 (m, 1H, *H*-5), 8.17–7.97 (m, 1H, *H*-8), 7.91–7.54 (m, 1H, *H*-7), 7.56–7.19 (m, 1H, *H*-6), 6.42 (s, 1H, *H*-3), 3.66 (hept, *J* = 6.9 Hz, 1H, *H*-1'), 1.24 (d, *J* = 6.9 Hz, 6H, *H*-2').
^{13}C NMR (101 MHz, D_2O+ 5% NaOD) δ [ppm]: 170.2, 159.2, 140.5, 131.3, 124.7, 124.6, 123.8, 117.5, 103.1, 28.1, 20.3.
HR-MS (ESI+): *m/z* for $C_{12}H_{13}NO_2$+H$^+$, [M+H]$^+$ calculated: 204.1019; found: 204.1012.

1*H*-2-Cyclopropyl-1-hydroxyquinol-4-one (5e)

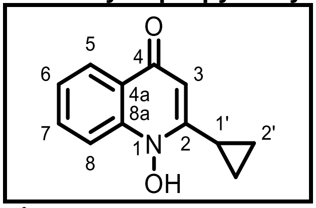

According to general protocol **GPIII 4e** (116.6 mg, 0.50 mmol) were reacted. 60.9 mg (0.30 mmol, 60%) of the product were obtained as colorless solid by reversed phase column chromatography (C 18 silica gel, gradient: 10% acetonitrile → 90% acetonitrile).

^{1}H NMR (400 MHz, D_2O+ 5% NaOD) δ [ppm]: 8.20–8.16 (m, 1H, *H*-5), 8.08–8.01 (m, 1H, *H*-8), 7.70–7.61 (m, 1H, *H*-7), 7.40–7.31 (m, 1H, *H*-6), 5.97 (s, 1H, *H*-3), 2.52–2.27 (m, 1H, *H*-1'), 1.03–0.92 (m, 2H, *H*-2'), 0.75–0.62 (m, 2H, *H*-2').
^{13}C NMR (101 MHz, D_2O+ 5% NaOD) δ [ppm]: 170.0, 154.8, 140.4, 131.2, 124.6, 124.4, 123.7, 117.0, 102.0, 11.2, 7.6.
HR-MS (ESI+): *m/z* for $C_{12}H_{11}NO_2$+H$^+$, [M+H]$^+$ calculated: 202.0863; found: 202.0859.

1*H*-2-(*tert*-Butyl)-1-hydroxyquinol-4-one (5f)

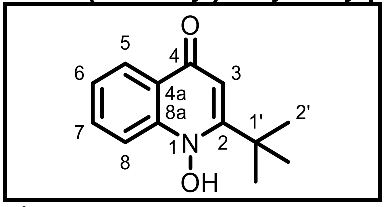

According to general protocol **GPIII 4f** (124.7 mg, 0.50 mmol) were reacted. 63.3 mg (0.29 mmol, 58%) of the product were obtained as colorless solid by reversed phase column chromatography (C 18 silica gel, gradient: 20% acetonitrile → 90% acetonitrile).

^{1}H NMR (400 MHz, D_2O+ 5% NaOD) δ [ppm]: 8.23 (dd, *J* = 8.8, 1.1 Hz, 1H, *H*-5), 8.06 (dd, *J* = 8.1, 1.5 Hz, 1H, *H*-8), 7.66 (ddd, *J* = 8.8, 6.9, 1.5 Hz, 1H, *H*-6), 7.39 (ddd, *J* = 8.1, 6.9, 1.1 Hz, 1H, *H*-7), 6.52 (s, 1H, *H*-3), 1.44 (s, 9H, *H*-2‘).
^{13}C NMR (101 MHz, D_2O+ 5% NaOD) δ [ppm]: 169.8, 159.2, 141.9, 131.2, 124.7, 124.5, 123.6, 117.7, 104.6, 36.1 ,27.6.
HR-MS (ESI+): *m/z* for $C_{13}H_{15}NO_2$+H$^+$, [M+H]$^+$ calculated: 218.1176; found: 218.1176.

1*H*-1-Hydroxy-4-oxo-1,4-dihydroquinolin-2-carboxylic acid (5g)

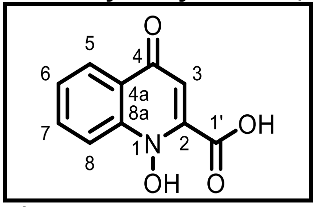

According to general protocol **GPIII 4g** (118.6 mg, 0.50 mmol) were reacted. 73.1 mg (0.36 mmol, 71%) of the product were obtained as colorless solid by reversed phase column chromatography (C 18 silica gel, gradient: 20% acetonitrile → 50% acetonitrile).

^{1}H NMR (600 MHz, DMSO-*d*$_6$) δ [ppm]: 8.54–8.43 (m, 1H, *H*-5), 8.33–8.27 (m, 1H, *H*-8), 8.10–8.00 (m, 1H, *H*-7), 7.88–7.79 (m, 1H, *H*-6), 7.48 (s, 1H, *H*-3).
^{13}C NMR (151 MHz, DMSO-*d*$_6$) δ [ppm]: 162.0, 160.5, 137.8, 136.3, 133.6, 128.9, 123.6, 122.9, 118.0, 104.3.
HR-MS (ESI-): *m/z* for $[C_{10}H_7NO_4\text{-}H]^-$, $[M\text{-}H]^-$ calculated: 204.0302; found: 204.0308.

1*H*-1-Hydroxy-2-phenylquinol-4-one (5h)

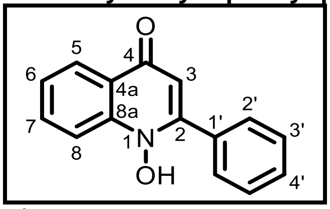

According to general protocol **GPIII 4h** (134.7 mg, 0.50 mmol) were reacted. 75.8 mg (0.32 mmol, 64%) of the product were obtained as light-yellow solid by reversed phase column chromatography (C 18 silica gel, gradient: 30% acetonitrile → 60% acetonitrile).

^{1}H NMR (600 MHz, DMSO-*d*$_6$) δ [ppm]: 11.87 (bs, 1H, O*H*), 8.25–8.07 (m, 1H, *H*-5), 7.96–7.85 (m, 1H, *H*-8), 7.82–7.72 (m, 1H, *H*-7), 7.69–7.61 (m, 2H, *H*-2'), 7.56–7.48 (m, 3H, *H*-2', *H*-3'), 7.47–7.41 (m, 1H, *H*-6), 6.04 (s, 1H, *H*-3).
^{13}C NMR (151 MHz, DMSO-*d*$_6$) δ [ppm]: 174.1, 151.4, 140.6, 133.0, 132.4, 129.6, 129.4, 128.2, 125.2, 125.1, 123.9, 115.5, 108.6.
HR-MS (ESI+): *m/z* for $C_{15}H_{11}NO_2+H^+$, $[M+H]^+$ calculated: 238.0683; found: 238.0856.
The spectroscopic data are in accordance with those reported in the literature.[10]

1*H*-1-Hydroxy-2-propylquinol-4-one (5i)

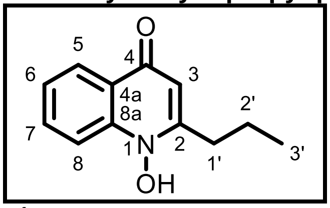

According to general protocol **GPIII 4i** (117.6 mg, 0.50 mmol) were reacted. 83.6 mg (0.41 mmol, 82%) of the product were obtained as colorless solid by reversed phase column chromatography (C 18 silica gel, gradient: 20% acetonitrile → 90% acetonitrile).

^{1}H NMR (400 MHz, D_2O+ 5% NaOD) δ [ppm]: 8.28–8.16 (m, 1H, *H*-5), 8.14–8.06 (m, 1H, *H*-8), 7.76–7.58 (m, 1H, *H*-7), 7.49–7.34 (m, 1H, *H*-6), 6.34 (s, 1H, *H*-3), 2.79 (t, *J* = 7.6 Hz, 2H, *H*-1'), 1.67 (tq, *J* = 7.6, 7.4 Hz, 2H, *H*-2'), 0.92 (t, *J* = 7.4 Hz, 3H, *H*-3').
^{13}C NMR (101 MHz, D_2O+ 5% NaOD) δ [ppm]: 169.7, 153.8, 140.4, 131.1, 125.0, 124.5, 123.8, 117.2, 106.8, 33.4, 20.2, 13.0.
HR-MS (ESI+): *m/z* for $C_{12}H_{13}NO_2+H^+$, $[M+H]^+$ calculated: 204.1019; found: 204.0135.

1*H*-7-Fluoro-1-hydroxy-2-methylquinol-4-one (5k)

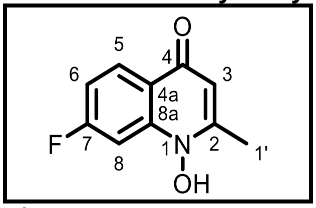

According to general protocol **GPIII 4k** (112.6 mg, 0.50 mmol) were reacted. 78.6 mg (0.41 mmol, 81%) of the product were obtained as colorless solid by reversed phase column chromatography (C 18 silica gel, gradient: 10% acetonitrile → 55% acetonitrile).

^{1}H NMR (400 MHz, DMSO-*d*$_6$) δ [ppm]: 8.18–7.94 (m, 1H, *H*-5), 7.64–7.40 (m, 1H, *H*-8), 7.14–6.79 (m, 1H, *H*-6), 5,81 (s, 1H, *H*-3), 2.24 (s, 3H, *H*-1')
^{13}C NMR (101 MHz, DMSO-*d*$_6$) δ [ppm]: 170.5, 163.7 (d, *J* = 183.0 Hz), 148.8, 142.0 (d, *J* = 11.6 Hz), 127.9 (d, *J* = 10.3 Hz), 123.0, 110.3 (d, *J* = 23.8 Hz), 106.4, 101.5 (d, *J* = 26.4 Hz), 18.6.
^{19}F NMR (376 MHz, DMSO-*d*$_6$) δ [ppm]: -111.75 (m).
HR-MS (ESI+): *m/z* for $C_{10}H_8FNO_2+H^+$, $[M+H]^+$ calculated: 194.0612; found: 194.0606.

1*H*-7-Chloro-1-hydroxy-2-methylquinol-4-one (5l)

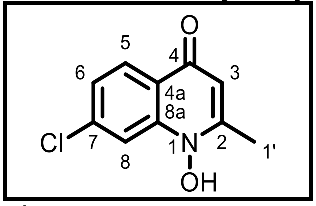

According to general protocol **GPIII 4l** (120.8 mg, 0.50 mmol) were reacted. 97.4 mg (0.046 mmol, 93%) of the product were obtained as colorless solid by reversed phase column chromatography (C 18 silica gel, gradient: 10% acetonitrile → 55% acetonitrile).

^{1}H NMR (400 MHz, DMSO-*d*$_6$) δ [ppm]: 11.80 (bs, 1H, O*H*), 8.07 (d, *J* = 8.6 Hz, 1H, *H*-5), 7.90–7.78 (m, 1H, *H*-8), 7.38 (dd, *J* = 8.6, 2.0 Hz, 1H, *H*-6), 6.02 (s, 1H; *H*-3), 2.40 (s, 3H, *H*-1').
^{13}C NMR (101 MHz, DMSO-*d*$_6$) δ [ppm]: 172.7, 151.0, 141.0, 136.9, 127.5, 123.6, 123.4, 114.1, 108.1, 17.9.
HR-MS (ESI+): *m/z* for $C_{10}H_8{}^{35}ClNO_2$+H$^+$, [M+H]$^+$ calculated: 210.0317; found: 210.0323.

1*H*-7-Bromo-1-hydroxy-2-methylquinol-4-one (5m)

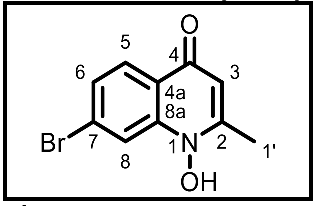

According to general protocol **GPIII 4m** (143.1 mg, 0.50 mmol) were reacted. 79.3 mg (0.31 mmol, 62%) of the product were obtained as colorless solid by reversed phase column chromatography (C 18 silica gel, gradient: 20% acetonitrile → 90% acetonitrile).

^{1}H NMR (400 MHz, D$_2$O+ 5% NaOD) δ [ppm]: 8.28–8.22 (m, 1H, *H*-8), 7.88–7.80 (m, 1H, *H*-5), 7.42–7.33 (m, 1H, *H*-6), 6.30 (s, 1H, *H*-3), 2.42 (s, 3H, *H*-1').
^{13}C NMR (101 MHz, D$_2$O+ 5% NaOD) δ [ppm]: 169.7, 151.3, 140.5, 127.3, 125.7, 125.2, 123.7, 119.4, 108.0, 18.5.
HR-MS (ESI+): *m/z* for $C_{10}H_8{}^{79}BrNO_2$+H$^+$, [M+H]$^+$ calculated: 253.9811; found: 253.9808.

1*H*-1-Hydroxy-2-methyl-7-(trifluoromethyl)quinol-4-one (5n)

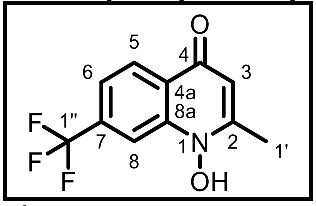

According to general protocol **GPIII 4n** (137.6 mg, 0.50 mmol) were reacted. 89.7 mg (0.37 mmol, 74%) of the product were obtained as colorless solid by reversed phase column chromatography (C 18 silica gel, gradient: 15% acetonitrile → 50% acetonitrile).

^{1}H NMR (600 MHz, DMSO-*d*$_6$) δ [ppm]: δ 11.97 (bs, 1H, O*H*), 8.29 (d, *J* = 8.3 Hz, 1H, *H*-5), 8.22–8.01 (m, 1H, *H*-8), 7.67 (dd, *J* = 8.3, 1.8 Hz, 1H, *H*-6), 6.14 (s, 1H, *H*-3), 2.45 (s, 3H, *H*-1').
^{13}C NMR (151 MHz, DMSO-*d*$_6$) δ [ppm]: 173.3, 151.7, 139.9, 131.8 (q, *J* = 32.2 Hz), 127.1, 123.8 (q, *J* = 272.4 Hz), 119.1, 112.4, 108.8, 18.0.
^{19}F NMR (376 MHz, DMSO-*d*$_6$) δ [ppm]: -62.55 (s).
HR-MS (ESI+): *m/z* for $C_{11}H_8F_3NO_2$+H$^+$, [M+H]$^+$ calculated: 244.0580; found: 244.0573.

Methyl 1-hydroxy-2-methyl-4-oxo-1,4-dihydroquinoline-7-carboxylate (5o)

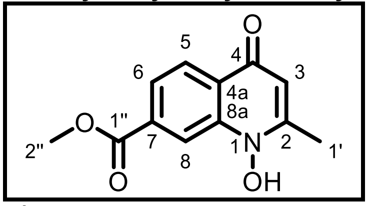

According to general protocol **GPIII 4o** (132.6 mg, 0.50 mmol) were reacted. 104.8 mg (0.45 mmol, 90%) of the product were obtained as yellow solid by reversed phase column chromatography (C 18 silica gel, gradient: 15% acetonitrile → 35% acetonitrile).

^{1}H NMR (600 MHz, DMSO-*d*$_6$) δ [ppm]: 11.93 (bs, 1H, O*H*), 8.42–8.38 (m, 1H, *H*-8), 8.20 (d, *J* = 8.3 Hz, 1H, *H*-5), 7.87 (dd, *J* = 8.4, 1.5 Hz, 1H, *H*-6), 6.10 (s, 1H, *H*-3), 3.93 (s, 3H, *H*-2''), 2.45 (s, 3H, *H*-1').
^{13}C NMR (151 MHz, DMSO-*d*$_6$) δ [ppm]: 174.2, 165.7, 151.5, 140.0, 132.3, 127.4, 126.1, 122.9, 116.3, 108.5, 52.7, 18.0.
HR-MS (ESI+): *m/z* for $C_{12}H_{11}NO_4$+H$^+$, [M+H]$^+$ calculated: 234.0761; found: 234.0755.

1*H*-6-Fluoro-1-hydroxy-2-methylquinol-4-one (5p)

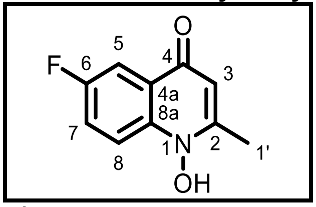

According to general protocol **GPIII 4p** (112.6 mg, 0.50 mmol) were reacted. 82.1 mg (0.42 mmol, 85%) of the product were obtained as colorless solid by reversed phase column chromatography (C 18 silica gel, gradient: 15% acetonitrile → 40% acetonitrile).

^{1}H NMR (400 MHz, DMSO-*d*$_6$) δ [ppm]: 11.82 (bs, 1H;), 7.97–7.82 (m, 1H, *H*-5), 7.73 (dd, *J* = 9.0, 3.7 Hz, 1H, *H*-8), 7.63 (ddd, *J* = 9.0, 8.7, 3.1 Hz, 1H, *H*-7), 6.03 (s, 1H, *H*-3), 2.42 (s, 3H, *H*-1').
^{13}C NMR (101 MHz, DMSO-*d*$_6$) δ [ppm]: 173.0, 158.5 (d, *J* = 242.4 Hz), 150.2, 137.1, 126.1, 120.5 (d, *J* = 25.2 Hz), 118.0, 109.2 (d, *J* = 22.5 Hz), 107.0, 17.8.
^{19}F NMR (376 MHz, DMSO-*d*$_6$) δ [ppm]: -119.54 (m).
HR-MS (ESI+): *m/z* for $C_{10}H_8FNO_2+H^+$, $[M+H]^+$ calculated: 194.0612; found: 194.0606.

1*H*-6-Chloro-1-hydroxy-2-methylquinol-4-one (5q)

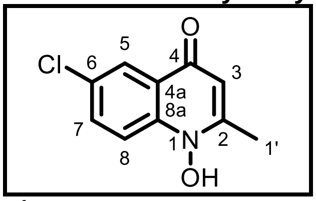

According to general protocol **GPIII 4q** (120.8 mg, 0.50 mmol) were reacted. 91.8 mg (0.44 mmol, 88%) of the product were obtained as colorless solid by reversed phase column chromatography (C 18 silica gel, gradient: 20% acetonitrile → 60% acetonitrile).

^{1}H NMR (400 MHz, DMSO-*d*$_6$) δ [ppm]: 11.83 (bs, 1H, O*H*), 8.01 (d, *J* = 2.4 Hz, 1H, *H*-5), 7.94–7.81 (m, 1H, H-*8*), 7.76 (dd, *J* = 9.0, 2.4 Hz, 1H, *H*-7), 6.04 (s, 1H, *H*-3), 2.41 (s, 3H, *H*-1').
^{13}C NMR (101 MHz, DMSO-*d*$_6$) δ [ppm]: 173.2, 150.8, 138.9, 132.0, 128.1, 125.6, 124.0, 117.3, 107.9, 17.9.
HR-MS (ESI+): *m/z* for $C_{10}H_8{}^{35}ClNO_2+H^+$, $[M+H]^+$ calculated: 210.0316; found: 210.0309.

1*H*-1-Hydroxy-6-methoxy-2-methylquinol-4-one (5r)

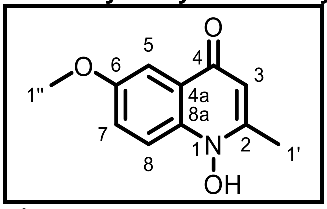

According to general protocol **GPIII 4r** (118.6 mg, 0.50 mmol) were reacted. 81.9 mg (0.40 mmol, 80%) of the product were obtained as beige solid by reversed phase column chromatography (C 18 silica gel, gradient: 10% acetonitrile → 55% acetonitrile).

^{1}H NMR (400 MHz, DMSO-*d*$_6$) δ [ppm]: 8.07 (d, *J* = 9.3 Hz, 1H, *H*-8), 7.46 (d, *J* = 2.9 Hz, 1H, *H*-5), 7.07 (dd, *J* = 9.3, 2.9 Hz, 1H, *H*-7), 5.76 (s, 1H, *H*-3), 3.79 (s, 3H, *H*-1''), 2.24 (s, 3H, *H*-1').
^{13}C NMR (101 MHz, DMSO-*d*$_6$) δ [ppm]: 168.0, 154.6, 145.5, 136.6, 127.6, 119.6, 119.3, 105.1, 103.9, 55.1, 18.7.
HR-MS (ESI+): *m/z* for $C_{11}H_{11}NO_3+H^+$, $[M+H]^+$ calculated: 206.0812; found: 206.0826.

1*H*-5-Fluoro-1-hydroxy-2-methylquinol-4-one (5s)

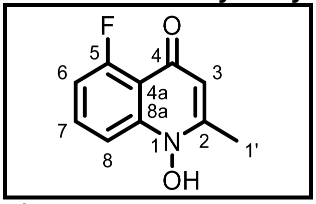

According to general protocol **GPIII 4s** (112.6 mg, 0.50 mmol) were reacted. 77.3 mg (0.40 mmol, 80%) of the product were obtained as colorless solid by reversed phase column chromatography (C 18 silica gel, gradient: 20% acetonitrile → 80% acetonitrile).

^{1}H NMR (400 MHz, D_2O+ 5% NaOD) δ [ppm]: 7.92–7.84 (m, 1H, *H*-8), 7.54–7.44 (m, 1H, *H*-7), 6.98–6.88 (m, 1H, *H*-6), 6.20 (s, 1H, *H*-3), 2.32 (s, 6H, *H*-1').
^{13}C NMR (101 MHz, D_2O+ 5% NaOD) δ [ppm]: 169.7, 159.9 (d, *J* = 255.2 Hz), 150.8, 142.0 (d, *J* = 4.3 Hz), 131.1 (d, *J* = 11.0 Hz), 115.1 (d, *J* = 9.8 Hz), 113.0 (d, *J* = 4.3 Hz), 109.8 (d, J = 22.0 Hz), 109.0, 18.3.
^{19}F NMR (376 MHz, D_2O+ 5% NaOD) δ [ppm]: -117.40 (dd, *J* = 12.9, 5.5 Hz).
HR-MS (ESI+): *m/z* for $C_{10}H_8FNO_2+H^+$, $[M+H]^+$ calculated: 194.0612; found: 194.0606.

1*H*-6,7-Difluoro-1-hydroxy-2-methylquinol-4-one (5t)

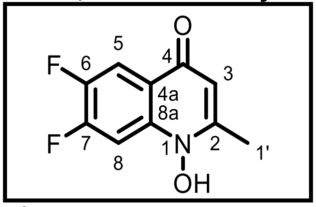

According to general protocol **GPIII 4t** (121.6 mg, 0.50 mmol) were reacted. 78.1 mg (0.37 mmol, 74%) of the product were obtained as colorless solid by reversed phase column chromatography (C 18 silica gel, gradient: 15% acetonitrile → 30% acetonitrile).

^{1}H NMR (400 MHz, DMSO-*d*$_6$) δ [ppm]: 12.14 (bs, 1H, O*H*), 8.12–7.57 (m, 2H, *H*-5, *H*-8), 6.02 (s, 1H, *H*-3), 2.40 (s, 3H, *H*-1').
^{13}C NMR (101 MHz, DMSO-*d*$_6$) δ [ppm]: 172.3, 152.8 (dd, *J* = 251.0, 15.7 Hz), 151.0, 146.8 (dd, *J* = 245.6, 14.2 Hz), 137.6 (d, *J* = 10.3 Hz), 121.7, 112.5 (d, *J* = 18.1 Hz), 107.4, 103.9 (d, *J* = 23.0 Hz), 17.9.
^{19}F NMR (376 MHz, DMSO-*d*$_6$) δ [ppm]: -131.97 (m), -143.84 (m).
HR-MS (ESI+): *m/z* for $C_{10}H_7F_2NO_2$+H$^+$, [M+H]$^+$ calculated: 212.0518; found: 212.0512.

1*H*-8-Chloro-1-hydroxy-2-methylquinol-4-one (5u)

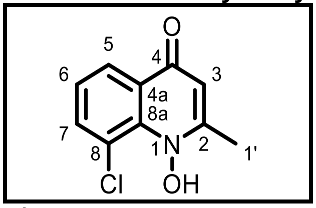

According to general protocol **GPIII 4u** (120.8 mg, 0.50 mmol) were reacted. 53.7 mg (0.26 mmol, 51%) of the product were obtained as colorless solid by reversed phase column chromatography (C 18 silica gel, gradient: 15% acetonitrile → 40% acetonitrile).

^{1}H NMR (400 MHz, DMSO-*d*$_6$) δ [ppm]: 11.52 (bs, 1H, O*H*), 8.11 (dd, *J* = 7.9, 1.7 Hz, 1H, *H*-5), 7.79 (dd, *J* = 7.7, 1.7 Hz, 1H, *H*-7), 7.31 (dd, *J* = 7.9, 7.7 Hz, 1H, *H*-6), 6.06 (s, 1H, *H*-3), 2.39 (s, 3H, *H*-1').
^{13}C NMR (101 MHz, DMSO-*d*$_6$) δ [ppm]: 173.9, 153.4, 137.0, 135.8, 127.9, 125.2, 123.9, 119.8, 108.5, 18.6.
HR-MS (ESI+): *m/z* for $C_{10}H_8{}^{35}ClNO_2$+H$^+$, [M+H]$^+$ calculated: 210.0316; found: 210.0319.

1*H*-2-Ethyl-3-methyl-1-hydroxyquinol-4-one (5v)

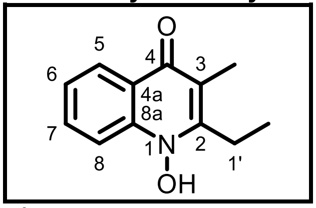

According to general protocol **GPIII 4v** (117.6 mg, 0.50 mmol) were reacted. 82.3 mg (0.041 mmol, 81%) of the product were obtained as colorless solid by reversed phase column chromatography (C 18 silica gel, gradient: 20% acetonitrile → 90% acetonitrile).

^{1}H NMR (400 MHz, D$_2$O+ 5% NaOD) δ [ppm]: 8.02–7.94 (m, 2H, *H*-5, *H*-8), 7.45 (ddd, *J* = 8.6, 6.8, 1.5 Hz, 1H, *H*-7), 7.19 (ddd, *J* = 8.2, 6.8, 1.2 Hz, 1H, *H*-6), 2.82 (q, *J* = 7.5 Hz, 2H, *H*-1'), 2.00 (s, 3H, *H*-1''), 0.99 (t, *J* = 7.5 Hz, 3H, *H*-2').
^{13}C NMR (101 MHz, D$_2$O+ 5% NaOD) δ [ppm]: 168.1, 154.0, 138.7, 130.1, 124.0, 123.8, 123.7, 117.0, 114.0, 22.0, 11.3, 10.6.
HR-MS (ESI-): *m/z* for [$C_{12}H_{13}NO_2$-H]$^-$, [M-H]$^-$ calculated: 202.0874; found: 202.0880.

2*H*,10*H*-10-hydroxy-6-(trifluoromethyl)-3,4-dihydroacridine-1,9-dione (5w)

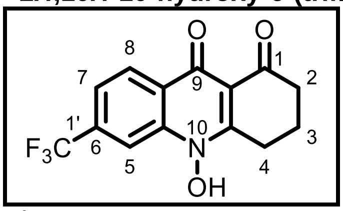

According to general protocol **GPIII** Nitisinone™ (164.6 mg, 0.50 mmol) were reacted. 110.9 mg (0.37 mmol, 75%) of the product were obtained as colorless solid by reversed phase column chromatography (C 18 silica gel, gradient: 30% acetonitrile → 40% acetonitrile).

^{1}H NMR (600 MHz, DMSO-*d*$_6$) δ [ppm]: 12.46 (bs, 1H, O*H*), 8.35 (d, *J* = 8.3 Hz, 1H, *H*-8), 8.16–8.12 (m, 1H, *H*-5), 7.76 (dd, *J* = 8.3, 1.7 Hz, 1H, *H*-7), 3.18 (t, *J* = 6.2 Hz, 2H, *H*-2), 2.42 (t, *J* = 6.6 Hz, 2H, *H*-4), 2.03 (tt, *J* = 6.6, 6.2 Hz, 2H, *H*-3).
^{13}C NMR (151 MHz, DMSO-*d*$_6$) δ [ppm]: 192.7, 171.0, 161.2, 139.4, 132.5 (q, *J* = 32.2 Hz), 129.1, 127.9, 123.6 (q, *J* = 273.0 Hz), 120.8, 113.1, 112.8, 38.1, 25.5, 19.8.
^{19}F NMR (376 MHz, DMSO-*d*$_6$) δ [ppm]: -62.64.
HR-MS (ESI+): *m/z* for $C_{14}H_{10}F_3NO_3$+H$^+$, [M+H]$^+$ calculated: 298.0686; found: 298.0683.

Methyl 1-hydroxy-2-methyl-4-oxo-1,4-dihydroquinoline-3-carboxylate (5y)

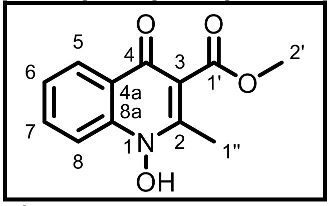

According to general protocol **GPIII 4y** (132.6 mg, 0.50 mmol) were reacted. 91.1 mg (0.39 mmol, 78%) of the product were obtained as colorless solid by reversed phase column chromatography (C 18 silica gel, gradient: 20% acetonitrile → 40% acetonitrile).

^{1}H NMR (400 MHz, DMSO-*d*$_6$) δ [ppm]: 12.04 (bs, 1H, O*H*), 8.12 (dd, *J* = 8.1, 1.5 Hz, 1H, *H*-5), 7.88–7.70 (m, 2H, *H*-7, *H*-8), 7.43 (ddd, *J* = 8.1, 7.0, 1.2 Hz, 1H, *H*-6), 3.78 (s, 3H, *H*-2'), 2.43 (s, 3H, *H*-1').
^{13}C NMR (101 MHz, DMSO-*d*$_6$) δ [ppm]: 171.4, 167.0, 149.1, 139.8, 132.8, 125.3, 124.8, 124.2, 115.0, 114.0, 52.0, 15.8.
HR-MS (ESI+): *m/z* for $C_{12}H_{11}NO_4+H^+$, $[M+H]^+$ calculated: 234.0761; found: 234.0756.

1*H*-1-Hydroxy-2-methyl-3-(4-(trifluoromethoxy)benzyl)quinol-4-one (5z)

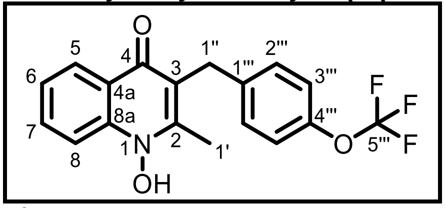

According to general protocol **GPIII 4z** (190.7 mg, 0.50 mmol) were reacted. 131.3 mg (0.38 mmol, 75%) of the product were obtained as colorless solid by reversed phase column chromatography (C 18 silica gel, gradient: 50% acetonitrile → 60% acetonitrile).

^{1}H NMR (400 MHz, DMSO-*d*$_6$) δ [ppm]: 11.73 (s, 1H, O*H*), 8.17 (dd, *J* = 8.1, 1.5 Hz, 1H, *H*-5), 7.86–7.79 (dd, 8.6, 1.7 Hz 1H, *H*-8), 7.72 (ddd, *J* = 8.6, 7.0, 1.5 Hz, 1H, *H*-7), 7.43–7.26 (m, 3H, *H*-6, *H*-2'''), 7.26–7.17 (m, 2H, *H*-3'''), 4.00 (s, 2H, *H*-2''), 2.44 (s, 3H, *H*-1').
^{13}C NMR (101 MHz, DMSO-*d*$_6$) δ [ppm]: 173.6, 148.5, 146.4, 140.8, 139.7, 131.9, 129.6, 125.4, 123.5, 123.1, 120.9, 120.1 (q, 160.8 Hz) 117.2, 114.6, 30.1, 14.9.
^{19}F NMR (376 MHz, DMSO-*d*$_6$) δ [ppm]: -53.27.
HR-MS (ESI+): *m/z* for $C_{18}H_{14}F_3NO_3+H^+$, $[M+H]^+$ calculated: 350.0999; found: 350.0981.

1*H*-6-chloro-2-Heptyl-1-hydroxyquinol-4-one (5aa)

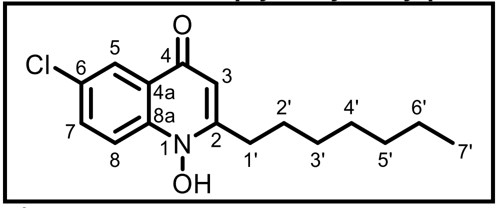

According to general protocol **GPIII 4aa** (145.7 mg, 0.50 mmol) were reacted. 122.9 mg (0.42 mmol, 84%) of the product were obtained as colorless solid by reversed phase column chromatography (C 18 silica gel, gradient: 50% acetonitrile → 90% acetonitrile).

^{1}H NMR (400 MHz, DMSO-*d*$_6$) δ [ppm]: 8.01–7.97 (m, 1H, *H*-5), 7.88–7.81 (m, 1H, *H*-8), 7.73–7.66 (m, 1H, *H*-7), 5.96 (s, 1H, *H*-3), 2.68 (t, *J* = 7.6 Hz, 2H, *H*-1'), 1.64–1.57 (m, 2H, *H*-2'), 1.29 – 1.22 (m, 8H, *H*-3', *H*-4', *H*-5', *H*-6'), 0.85 (t, *J* = 6.4 Hz, 3H, *H*-7').
^{13}C NMR (101 MHz, DMSO-*d*$_6$) δ [ppm]: 172.5, 154.0, 139.1, 131.8, 128.1, 125.9, 123.9, 117.7, 107.1, 31.2, 30.8, 28.7, 28.4, 27.3, 22.1, 14.0.
HR-MS (ESI+): *m/z* for $C_{16}H_{20}{}^{35}ClNO_2+H^+$, $[M+H]^+$ calculated: 294.1255; found: 294.1242.

5.3. 1*H*-Quinol-4-ones (6)

1*H*-2-Methylquinolin-4-one (6a)

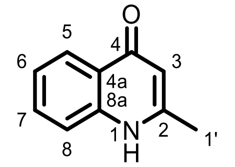

According to general protocol **GPIV 4a** (20.7 mg, 0.10 mmol) were reacted. 14.5 mg (0.091 mmol, 91%) of the product were obtained as colorless solid by reversed phase column chromatography (C 18 silica gel, gradient: 10% acetonitrile → 90% acetonitrile).

^{1}H NMR (400 MHz, DMSO-*d*$_6$) δ [ppm]: 11.57 (bs, 1H, N*H*), 8.03 (dd, 1H, *J* = 8.0; 1.6 Hz, *H*-5), 7.60 (ddd, 1H, *J* = 8.4; 6.9; 1.6 Hz, *H*-7), 7,48 (dd, 1H, *J* = 8.4; 1.2 Hz, *H*-8), 7,26 (ddd, 1H, *J* = 8.0; 6.9; 1.2 Hz, *H*-6), 5,91 (s, 1H; *H*-3), 2,33 (s, 3H, *H*-1')
^{13}C NMR (101 MHz, DMSO-*d*$_6$) δ [ppm]: 176.8, 149.7, 140.1, 131.45, 124.8, 124.5, 122.7, 117.7, 108.4, 19.5.
HR-MS (ESI+): *m/z* for $C_{10}H_9NO+H^+$, $[M+H]^+$ calculated: 160.0757; found: 160.0752.

5.4. *β*-Ketoesters (7)

Ethyl 3-oxodecanoate (7a)

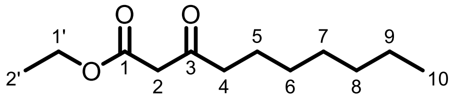

Octanoyl chloride (22.0 mL, 21.15 g 130 mmol) was added dropwise at 0 °C to a solution of 2,2-dimethyl-1,3-dioxan-4,6-dione (18.74 g, 130 mmol, 1.0 eq.), 4-dimethylamino-pyridine (3,18 g, 26 mmol, 0.2 eq.), in pyridine (150 mL) and CH_2Cl_2 (350 mL) and stirred for 16 h at room temperature. After completion of the reaction CH_2Cl_2 (350 mL) and 2 M HCl (300 mL) were added, and the organic fraction was washed three-times with 2 M HCl (3x300 mL) and brine (300 mL), dried over anhydrous Na_2SO_4, filtered, and concentrated in vacuo. The residue was dissolved in ethanol (200 mL) and refluxed for 16 h and concentrated in vacuo. The crude product was purified by filtration over silica (120 g silica (irregular); cyclohexane:ethyl acetate 1:1 (*v*:*v*)). 21.39 g (99.8 mmol, 77%) of the product were obtained as colorless liquid by distillation (90–92 °C at $1.2 \cdot 10^{-1}$ mbar).

^{1}H NMR (400 MHz, CDCl$_3$) δ [ppm]: 4.17 (q, *J* = 7.1 Hz, 2H, *H*-1'), 3.40 (s, 2H, *H*-2), 2.51 (t, *J* = 7.4 Hz, 2H, *H*-4), 1.67–1.46 (m, 2H, *H*-5), 1.38–1.04 (m, 11H, *H*-6, *H*-7, *H*-8, *H*-9, *H*-2'), 0.95–0.73 (m, 3H, *H*-10).
^{13}C NMR (101 MHz, CDCl$_3$) δ [ppm]: 203.1, 167.4, 61.4, 49.4, 43.1, 31.7, 29.1, 29.1, 23.6, 22.7, 14.2, 14.1.
HR-MS (ESI+): *m/z* for $C_{12}H_{22}O_3+H^+$, $[M+H]^+$ calculated: 215.1642; found: 215.1640.
The spectroscopic data are in accordance with those reported in the literature.[11]

5.5. Prenyl halogenide (8)

(*E*, *E*)- Farnesylbromide (8a)

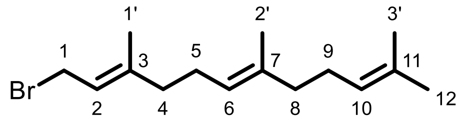

To a stirred solution of (*E*, *E*)-farnesol (10.0 g, 45.0 mmol, 1.0 eq) in 500 mL dry diethyl ether at 0 °C phosphorus tribromide (2.56 ml, 7.31 g, 27.0 mmol, 1.0 eq) was added dropwise and the reaction was kept for 2 h at 0 °C. After completion of the reaction the mixture was poured on ice (300 g). The aqueous phase was then extracted twice with 200 mL diethyl ether, the combined organic fractions were washed with water (500 mL), brine (500 mL) and dried over anhydrous sodium sulfate. The solvent was removed under reduced pressure. The residue was dissolved in hexane and filtered through a pad of sodium sulfate. The solvent was removed under reduced pressure and the crude product as colourless oil (11.24 g, 39.40 mmol, 88%) was used without further purification.

^{1}H NMR (400 MHz, $CDCl_3$) δ [ppm]: 5.53 (t, *J* = 8.5 Hz, 1H, *H*-2), 5.13–5.02 (m, 2H, *H*-6, *H*-10), 4.02 (d, *J* = 8.5 Hz, 2H, *H*-1), 2.17–1.93 (m, 8H, *H*-4, *H*-5, *H*-8, *H*-9), 1.73 (d, *J* = 1.4 Hz, 3H, *H*-1'), 1.68 (d, *J* = 1.5 Hz, 3H, *H*-2'), 1.60 (d, *J* = 1.5 Hz, 6H, *H*-12 *H*-3').
^{13}C NMR (101 MHz, $CDCl_3$) δ [ppm]: 143.7, 135.7, 131.4, 124.4, 123.5, 120.7, 39.8, 39.6, 29.8, 26.8, 26.2, 25.8, 17.8, 16.2, 16.1.
HR-MS (ESI+): *m/z* for $C_{15}H_{25}^+$, $[M-Br]^+$ calculated: 205.1951; found: 205.1958.
The spectroscopic data are in accordance with those reported in the literature.[12]

6. Crystallographic Data

1*H*-1-Hydroxy-2-methylquinolin-4-one (5a)

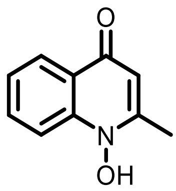

Crystallization was carried out by dissolving the compound in methanol at 40 °C. Slow evaporating resulted in crystal formation at room temperature.

CCDC Number	2367756
Empirical formular	$C_{10}H_9NO_2$
Moiety formular	$C_{10}H_9NO_2$
Formular weight	175.19
Temperature	120(2) K
Wavelength, radiation type	0.71073 Å, MoKα
Diffractometer	STOE IPDS 2T
Crystal system	Monoclinic
Space group name, number	P n, (7)
Unit cell dimensions	a = 7.7085(6) Å α = 90°
	b = 4.9495(4) Å β = 91.268(7)°
	c = 10.6020(9) Å γ = 90°
Volume	404.40(6) $Å^3$
Number of reflections	3510
And range used for lattice parameters	3.23° <=Θ<= 28.66°
Z	2
Density (calculated)	1.439 Mg/m^3
Absorption coefficient	0.101 mm^{-1}
Absorption correction	None
F(000)	184
Crystal size, colour and form	0.060 0.110 0.360 mm^3, colorless block
Theta range for data collection	3.234 bis 28.422°.
Index ranges	-9<=h<=10, -6<=k<=6, -14<=l<=14
Number of reflections:	
collected	2435
independent	1652 [R_{int} = 0.0228]
observed [I>2sigma(I)]	1425
Completeness to theta = 25.2°	99.9 %
Refinement method	Full-matrix least-squares on F^2
Data / restraints / parameters	1652 / 2 / 122, Hydrogen localized
Goodness-of-fit on F^2	1.029
Final R indices [I>2sigma(I)]	R1 = 0.0435, wR2 = 0.0946
R indices (all data)	R1 = 0.0557, wR2 = 0.1016
Largest diff. peak and hole	0.238 und -0.191 $eÅ^{-3}$

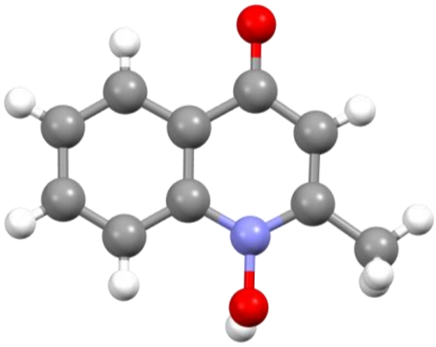

Figure S7: Molecular structure of compound **5a**.

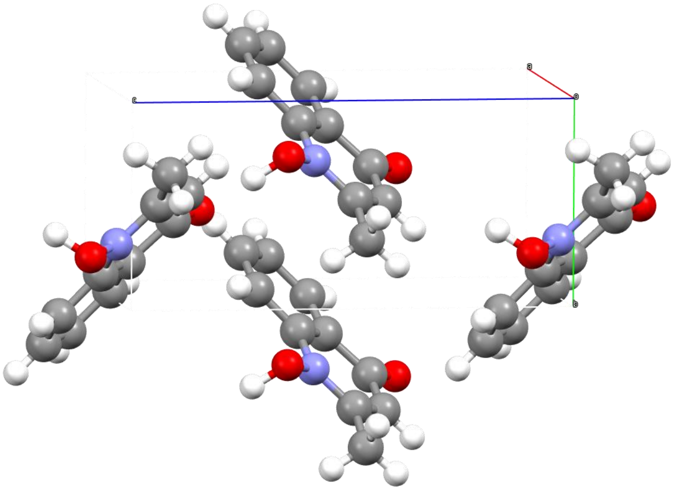

Figure S8: Packing of compound **5a**.

7. NMR Spectra

^{1}H NMR spectrum (400 MHz, DMSO-d_6) of **1a**

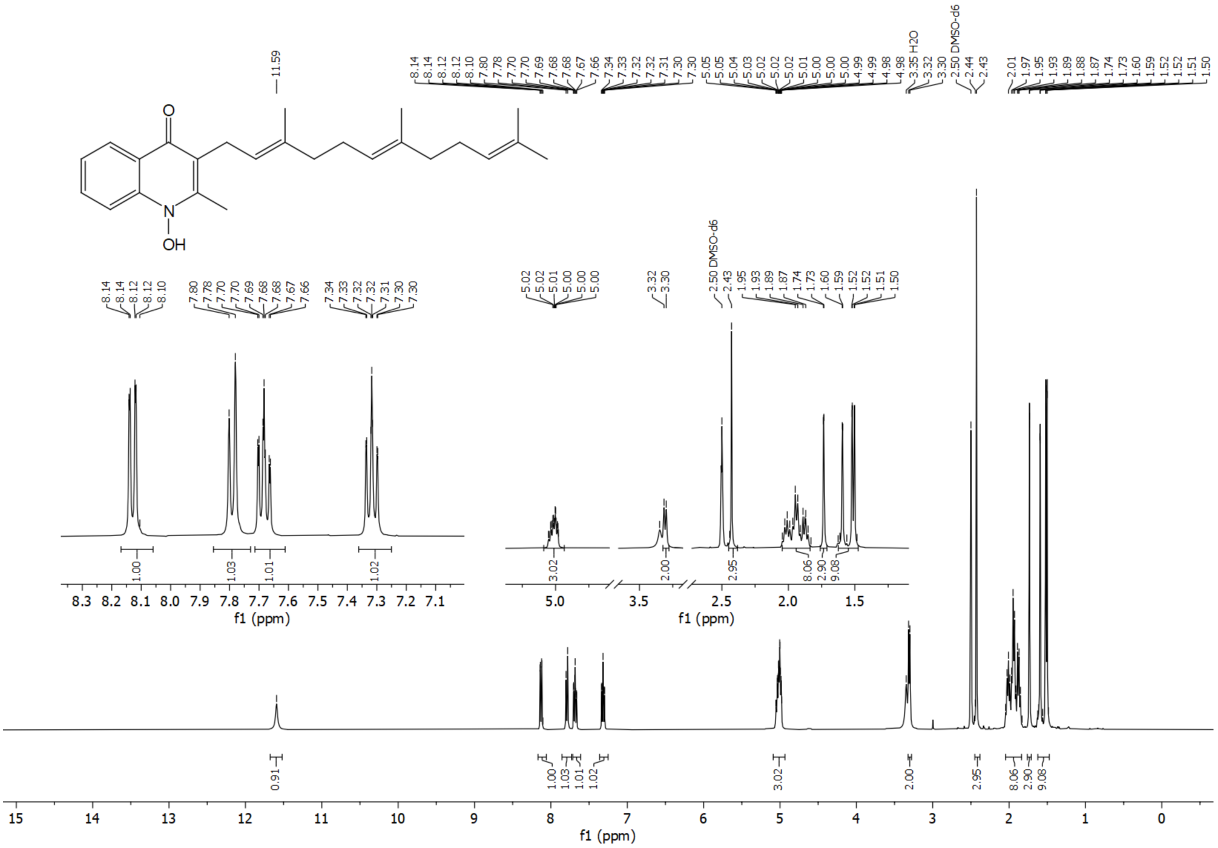

^{13}C NMR spectrum (101 MHz, DMSO-d_6) of **1a**

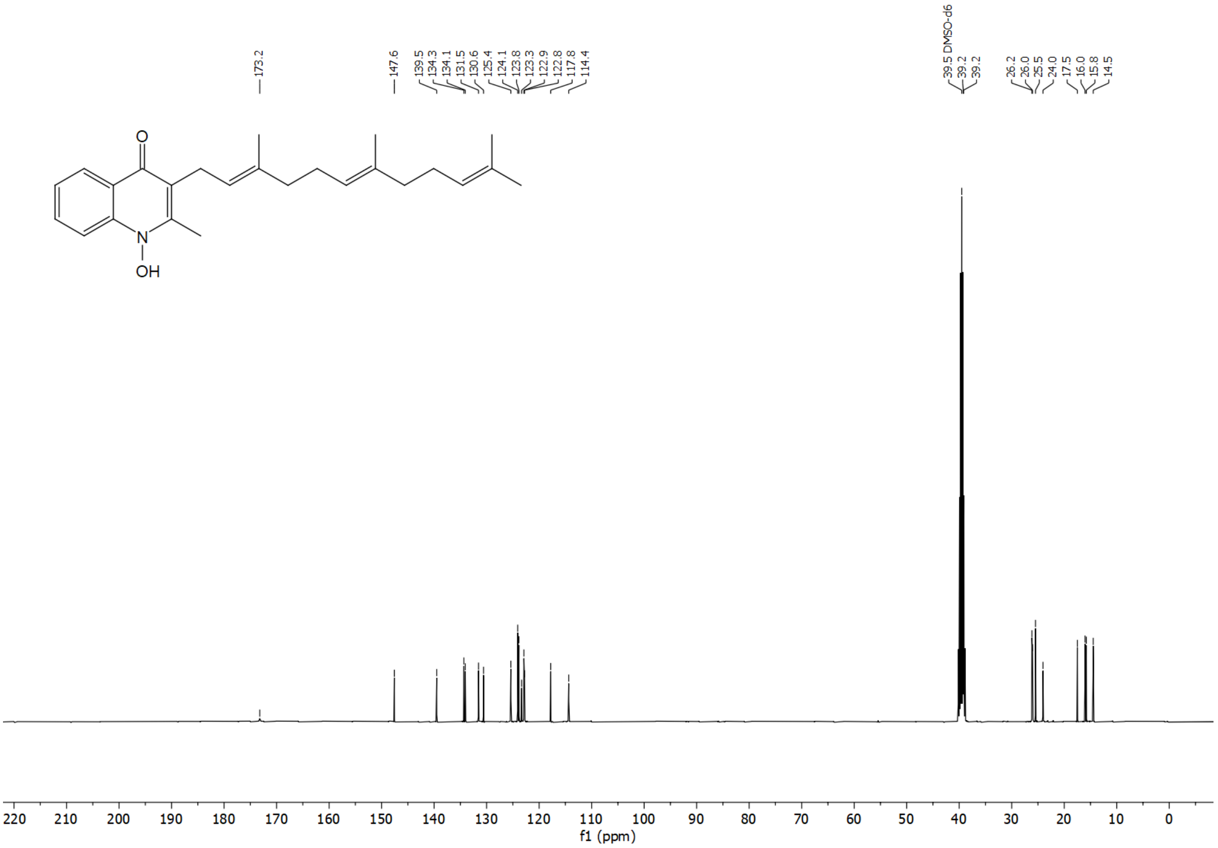

^{1}H NMR spectrum (600 MHz, DMSO-d_6) of **2**

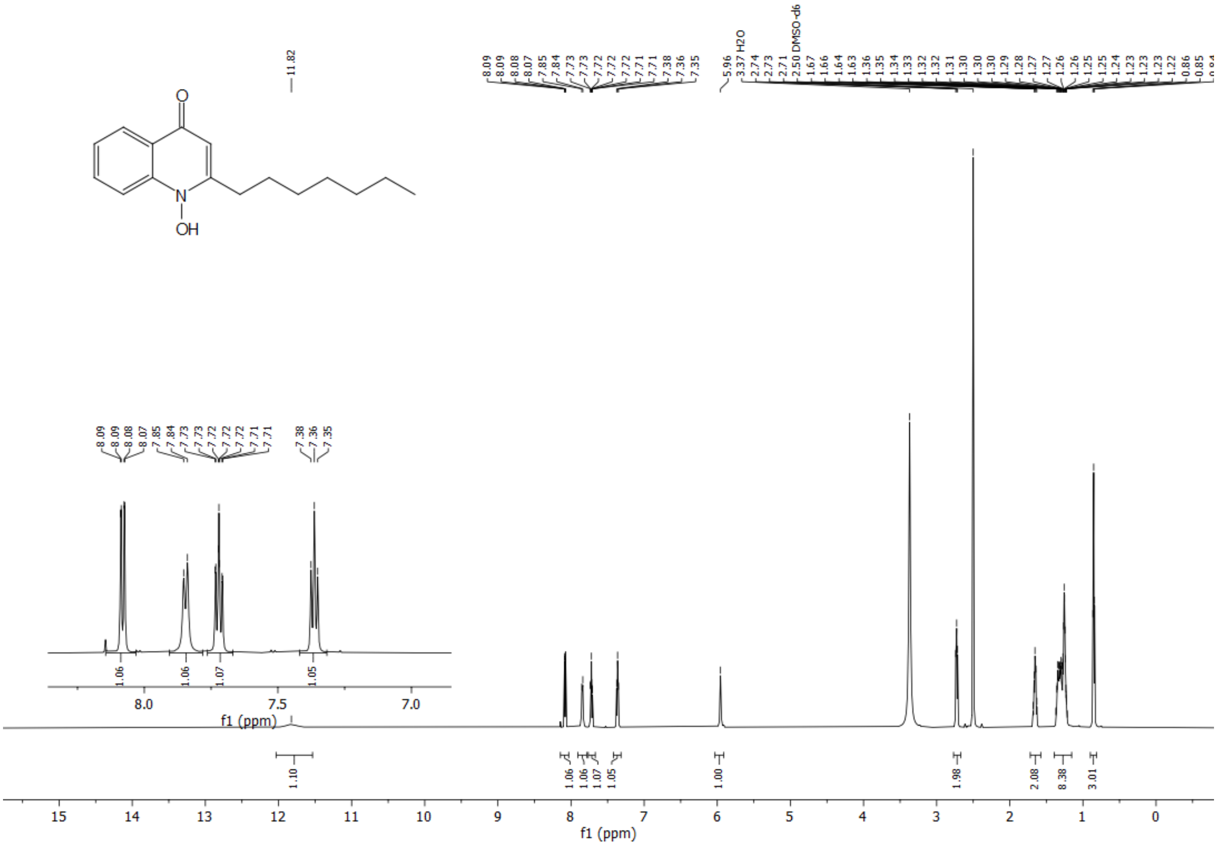

^{13}C NMR spectrum (151 MHz, DMSO-d_6) of **2**

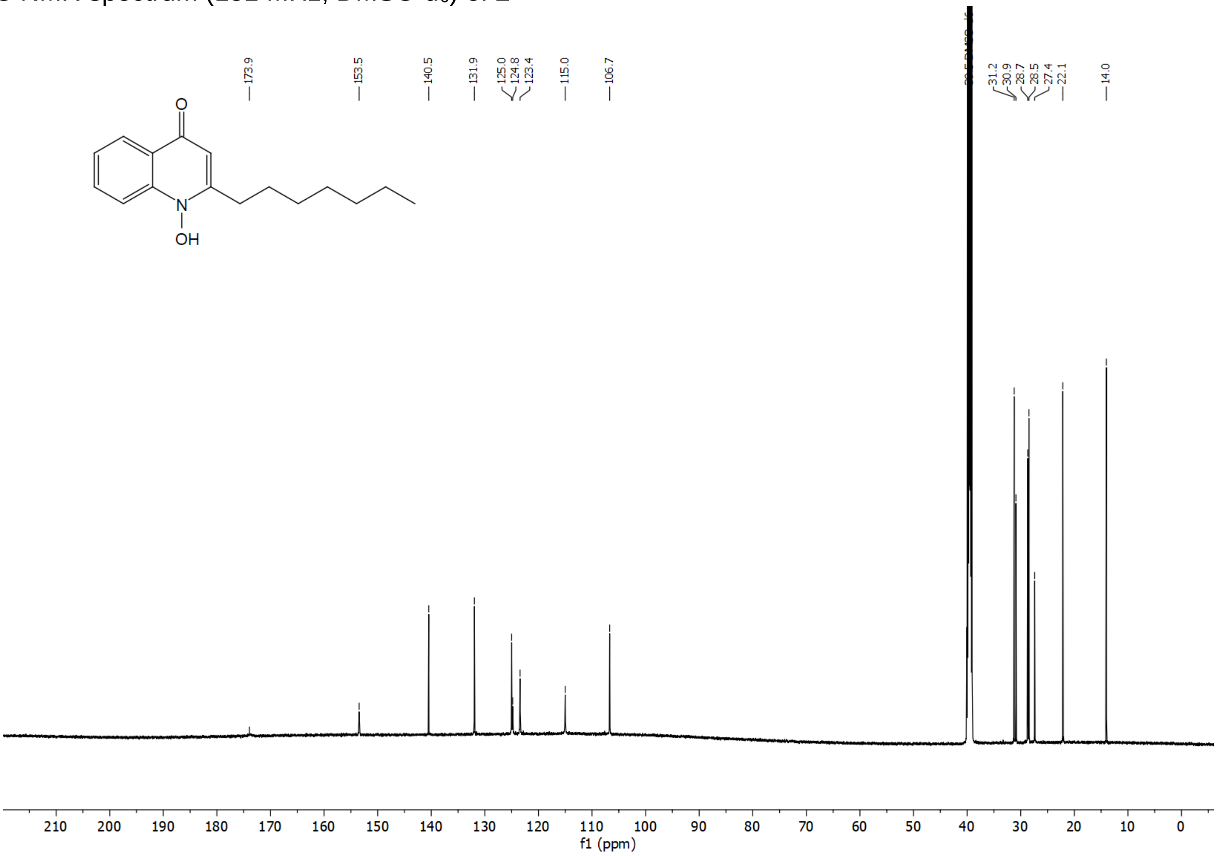

^{1}H NMR spectrum (400 MHz, $CDCl_3$) of **4a**

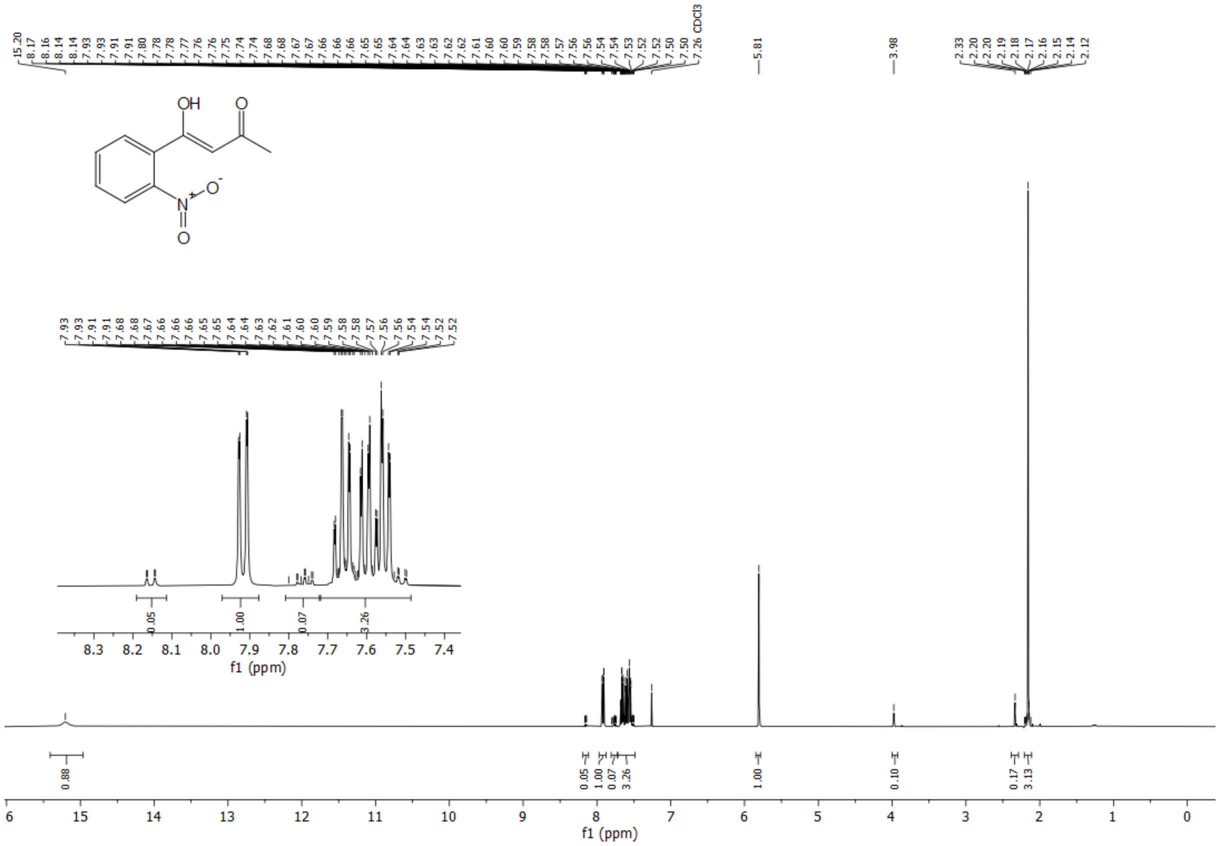

^{13}C NMR spectrum (101 MHz, $CDCl_3$) of **4a**

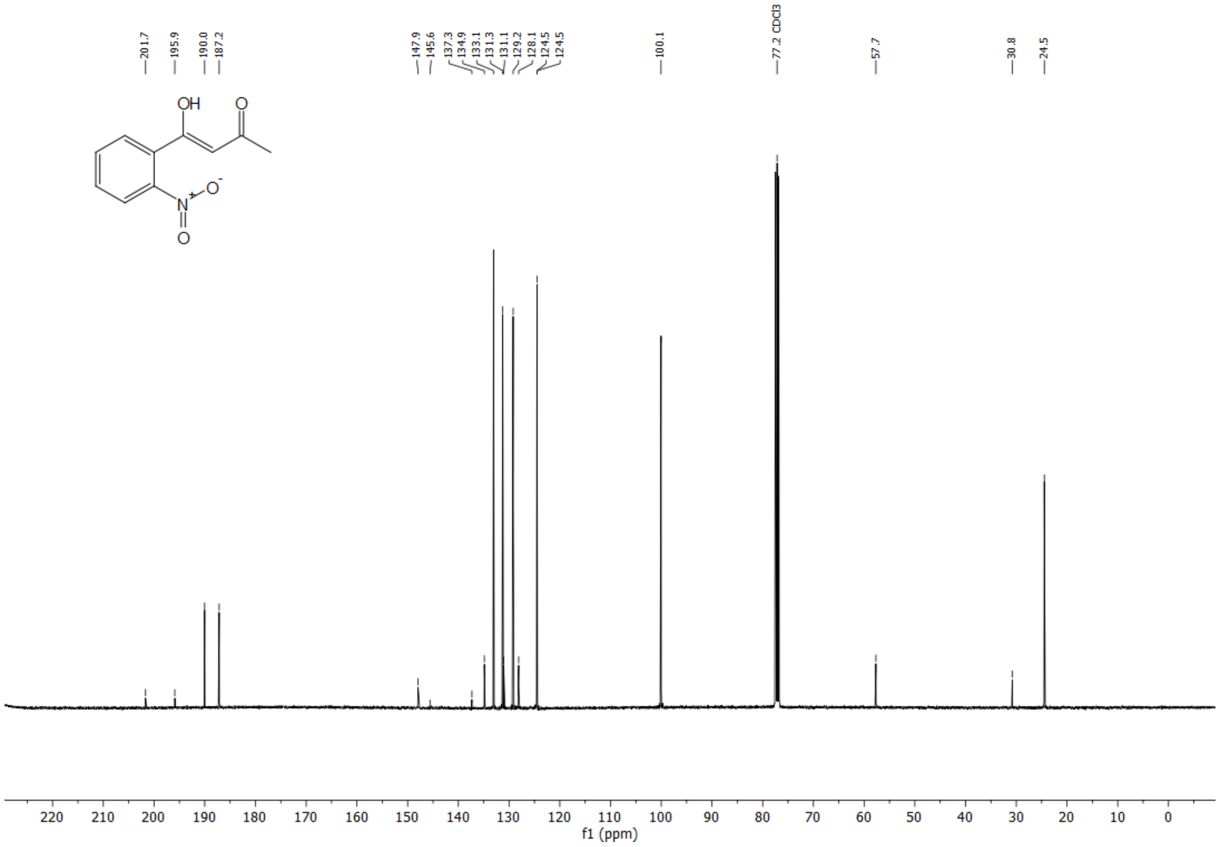

^{1}H NMR spectrum (400 MHz, DMSO-d_6) of **4c**

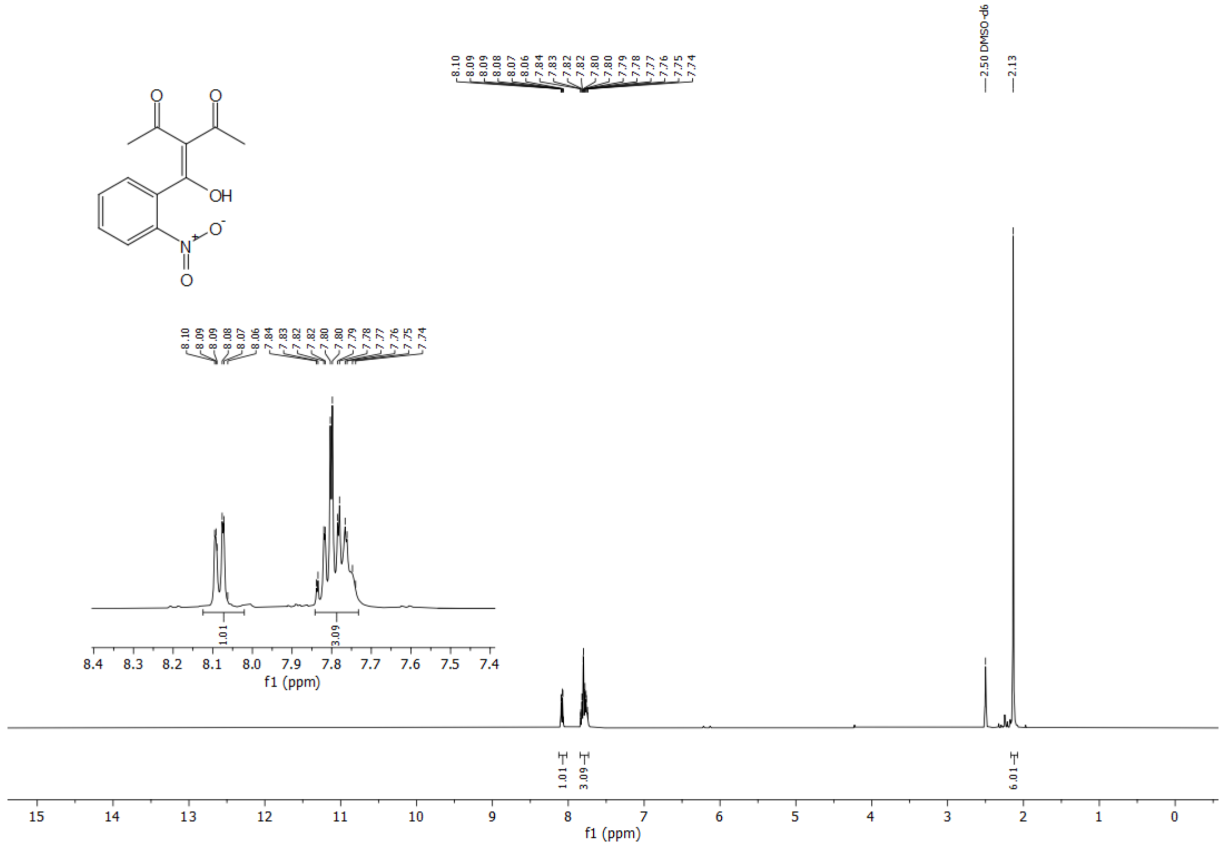

^{13}C NMR spectrum (101 MHz, DMSO-d_6) of **4c**

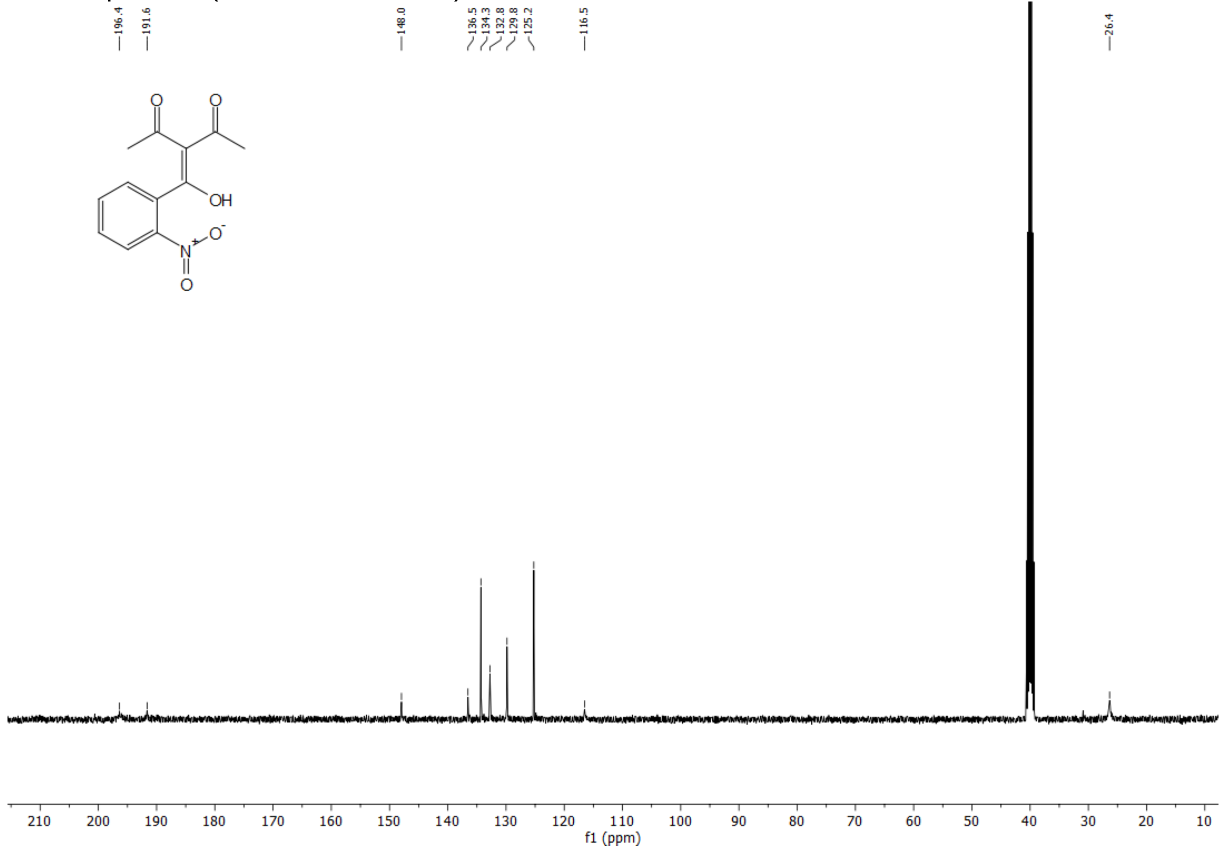

^{1}H NMR spectrum (400 MHz, $CDCl_3$) of **4d**

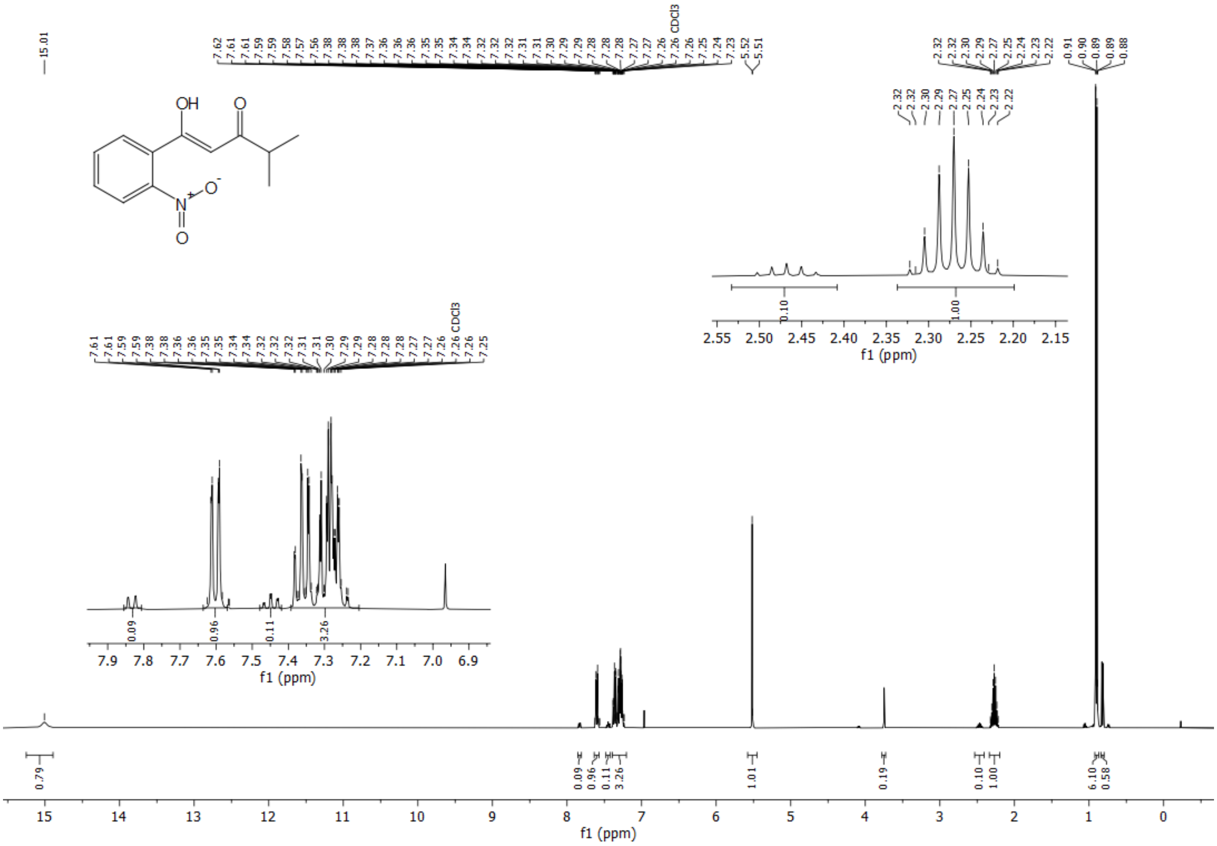

^{13}C NMR spectrum (101 MHz, $CDCl_3$) of **4d**

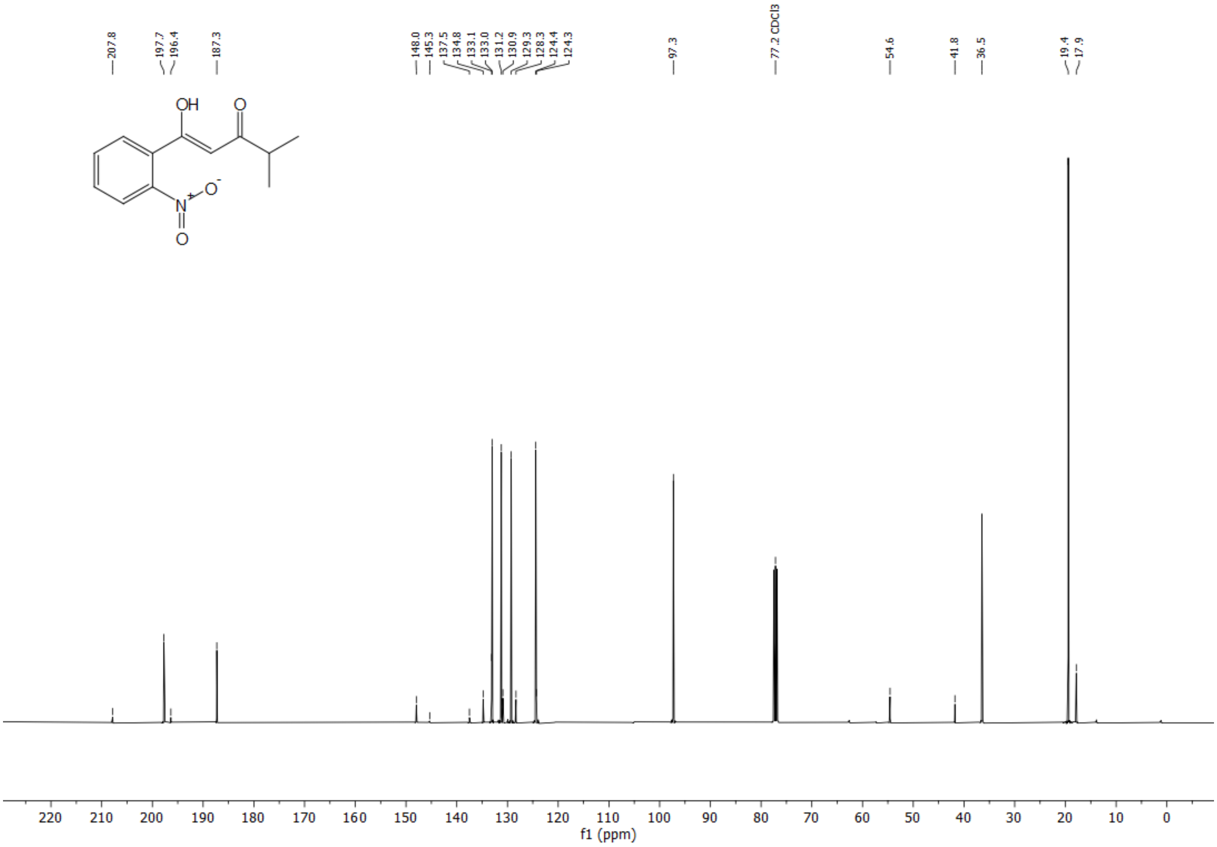

^{1}H NMR spectrum (400 MHz, $CDCl_3$) of **4e**

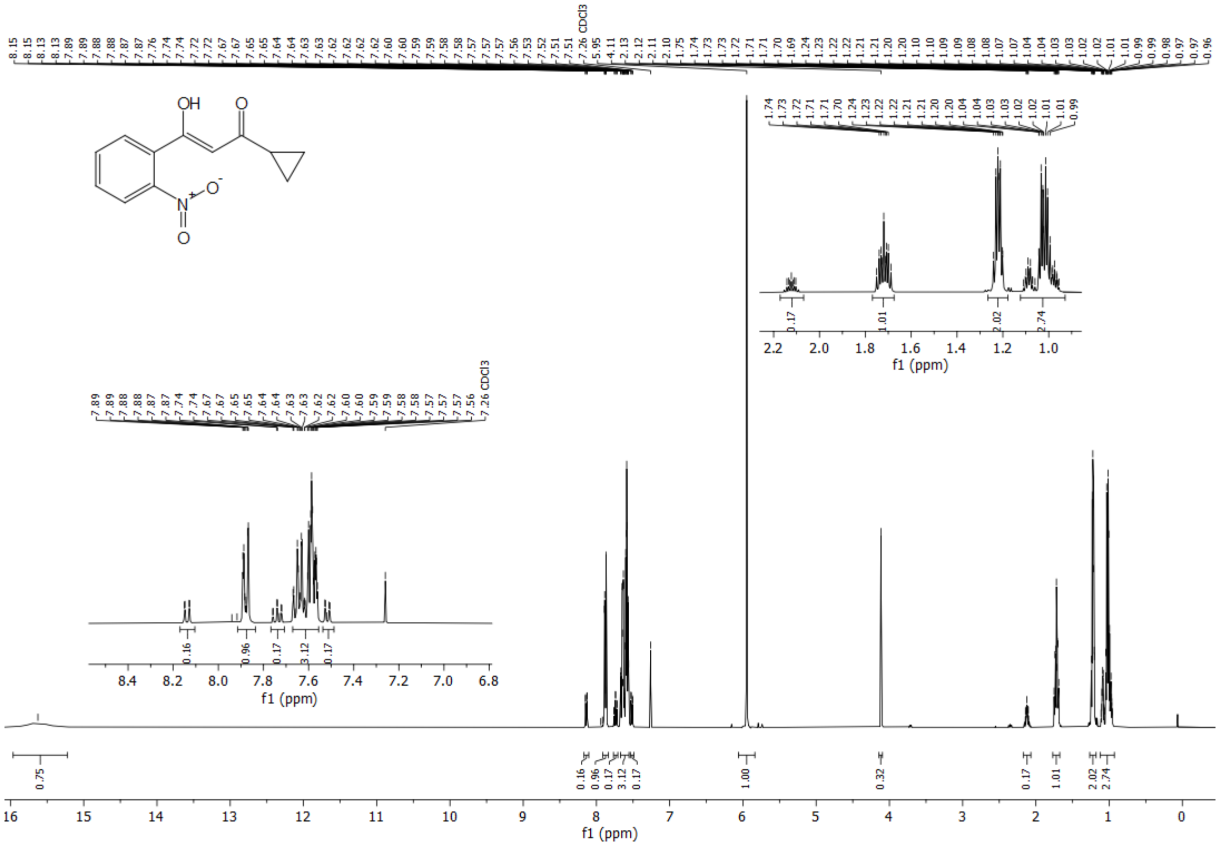

^{13}C NMR spectrum (101 MHz, $CDCl_3$) of **4e**

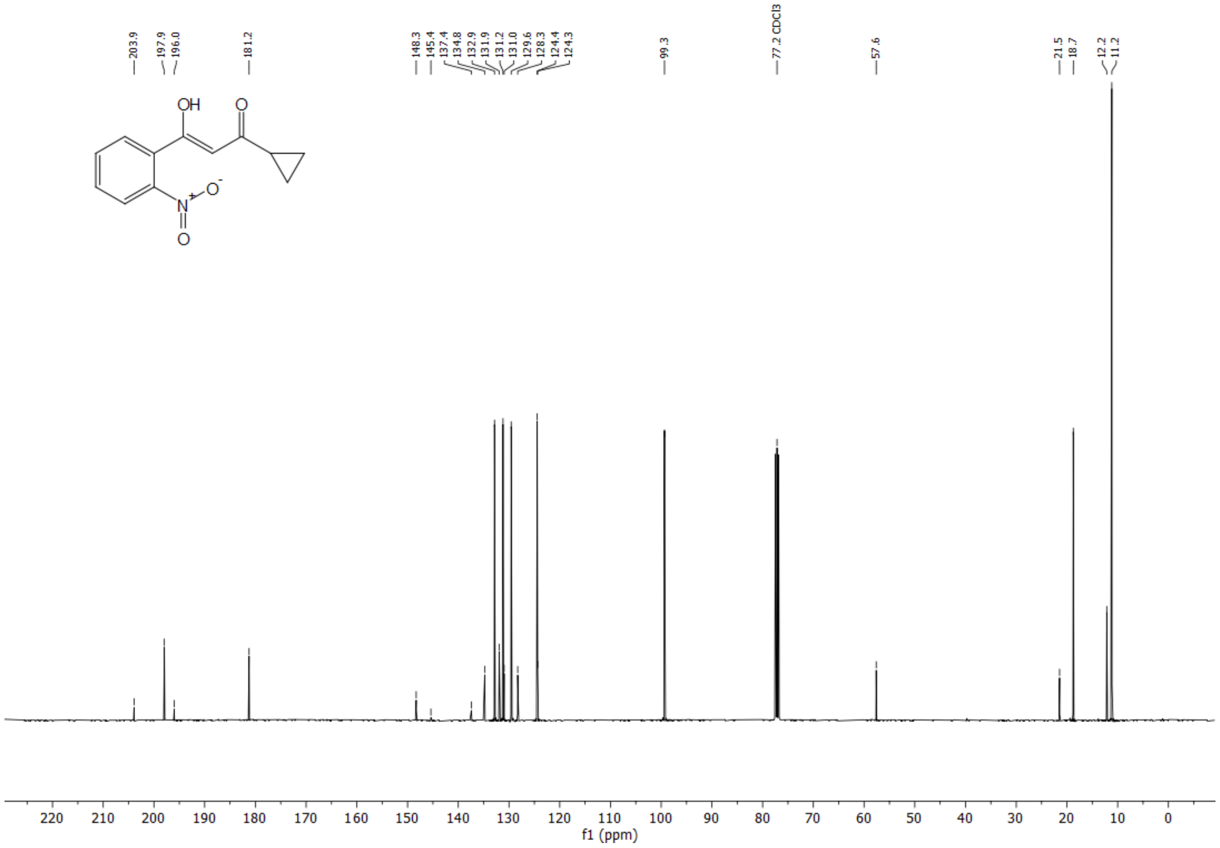

^1H NMR spectrum (400 MHz, $CDCl_3$) of **4f**

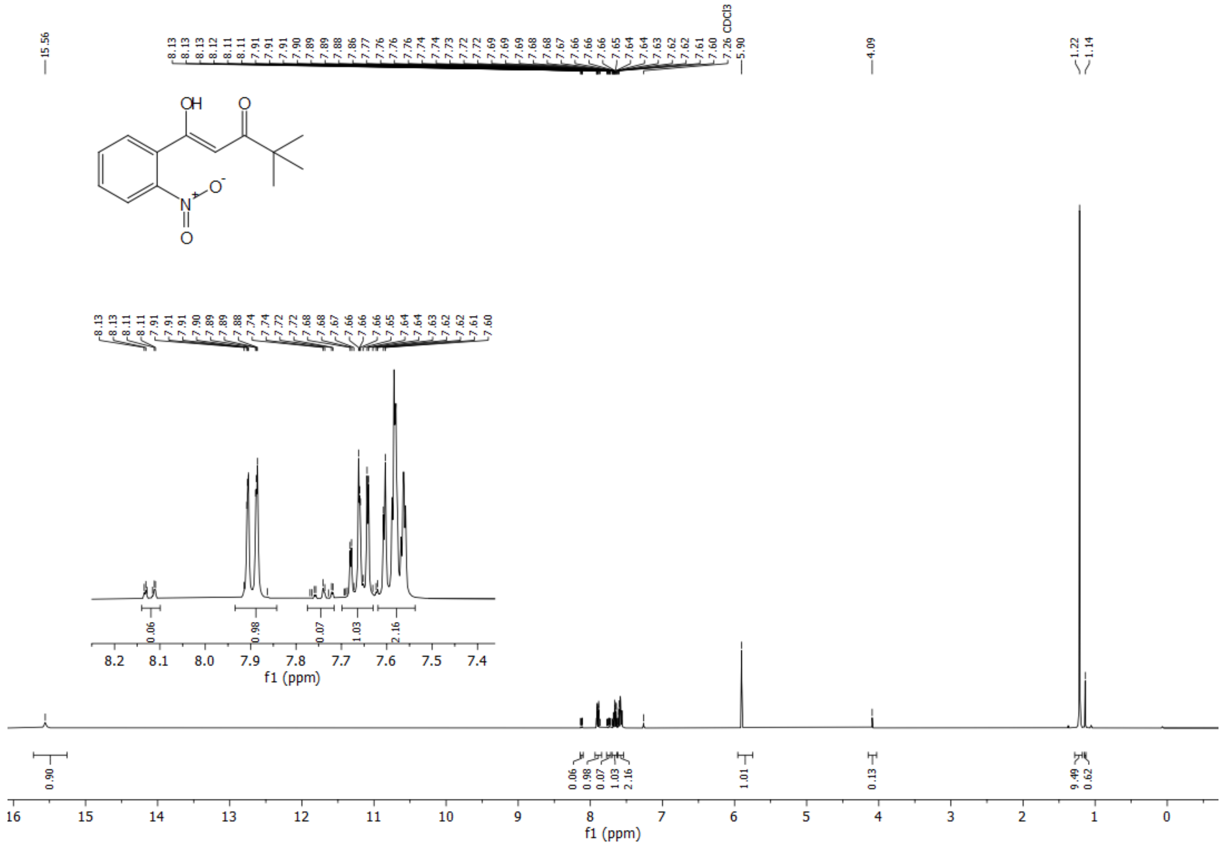

^{13}C NMR spectrum (101 MHz, $CDCl_3$) of **4f**

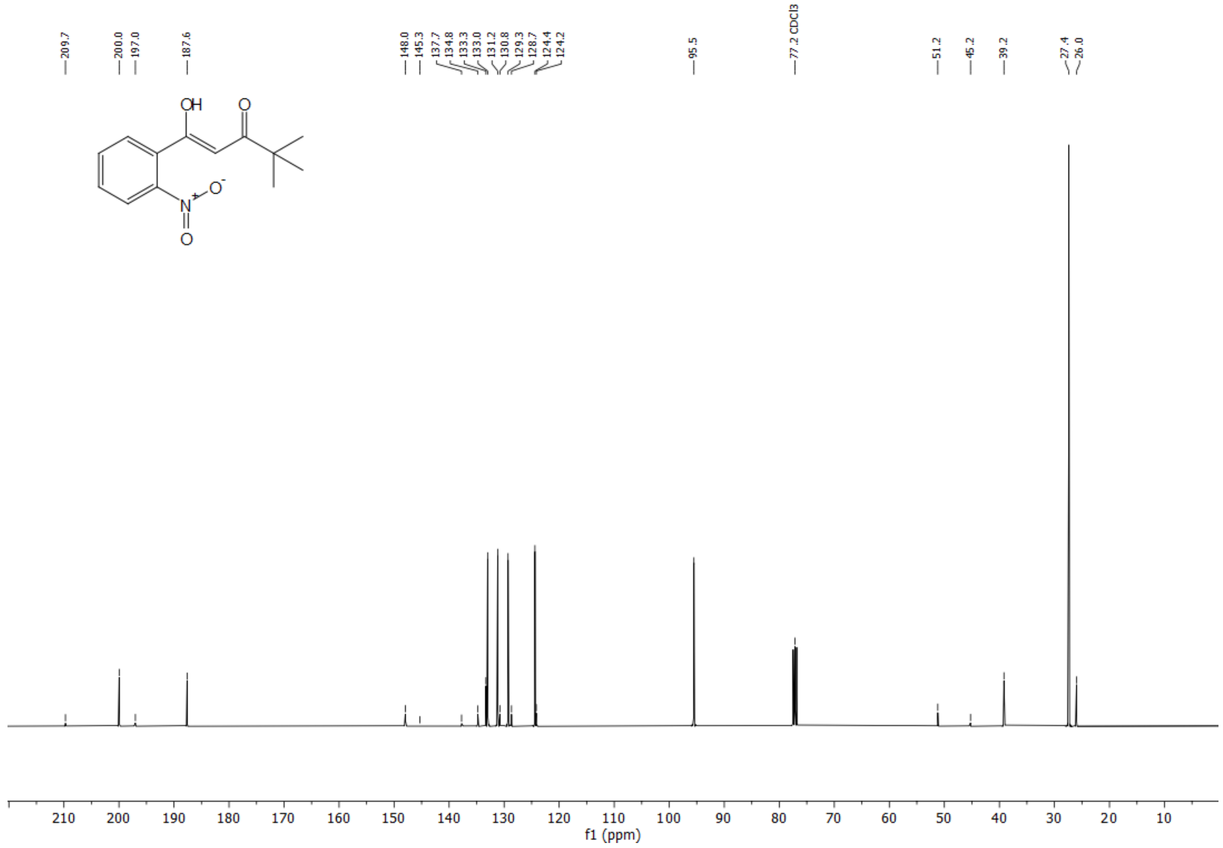

^{1}H NMR spectrum (400 MHz, DMSO-d_6) of **4g**

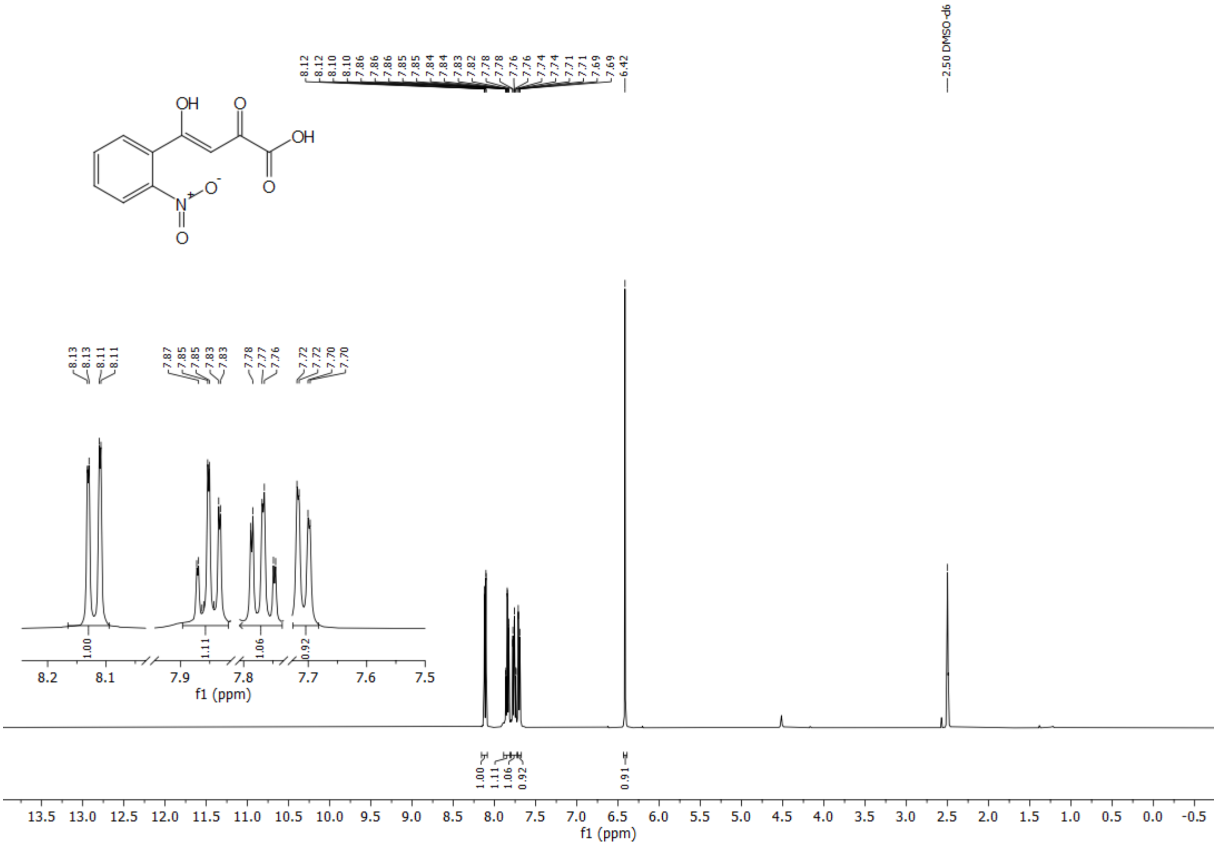

^{13}C NMR spectrum (101 MHz, DMSO-d_6) of **4g**

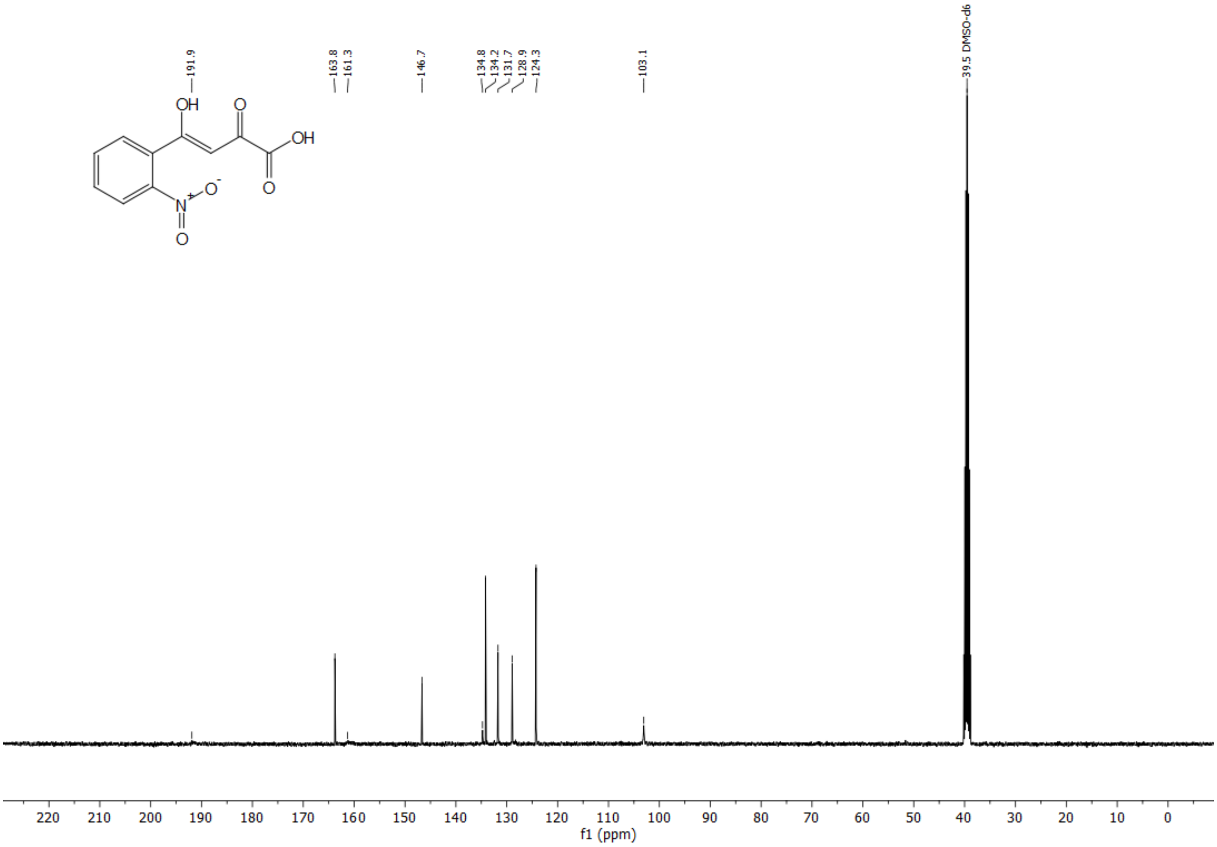

^{1}H NMR spectrum (400 MHz, $CDCl_3$) of **4h**

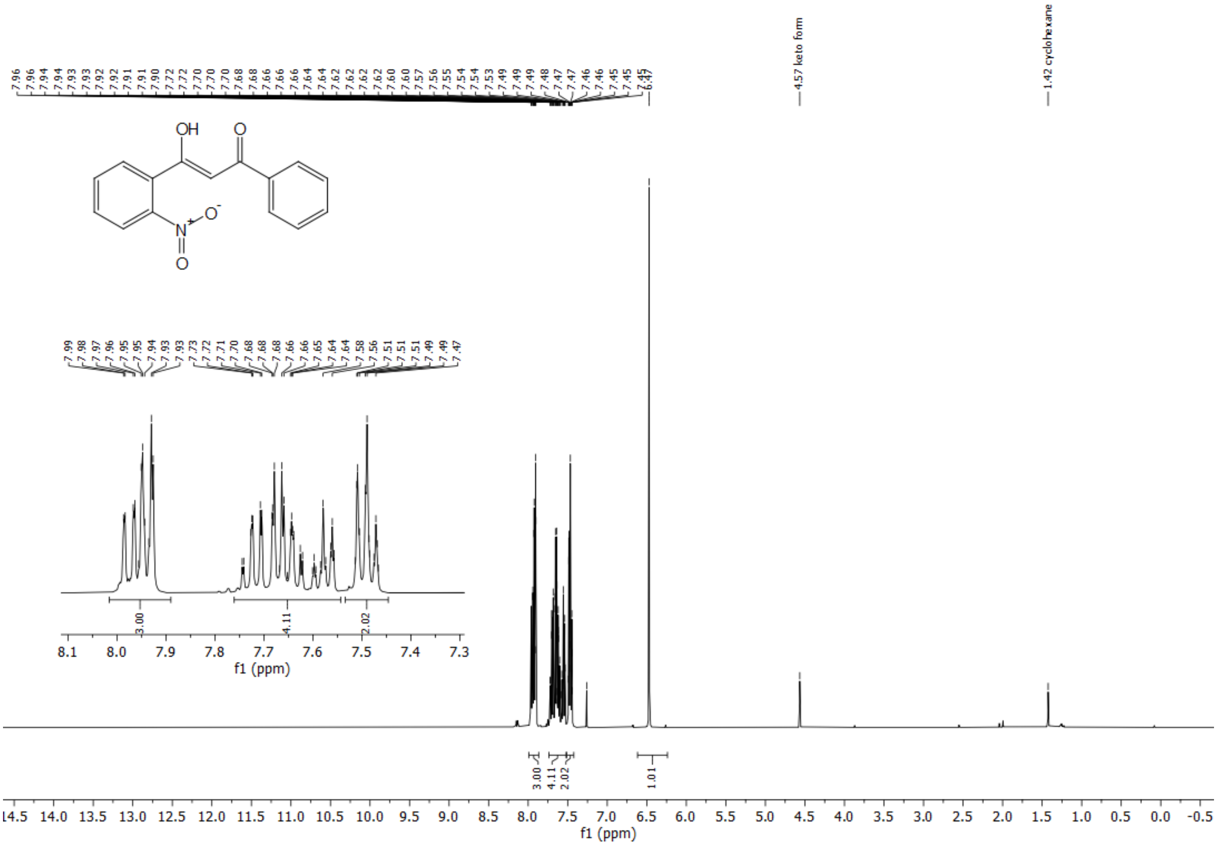

^{13}C NMR spectrum (101 MHz, $CDCl_3$) of **4h**

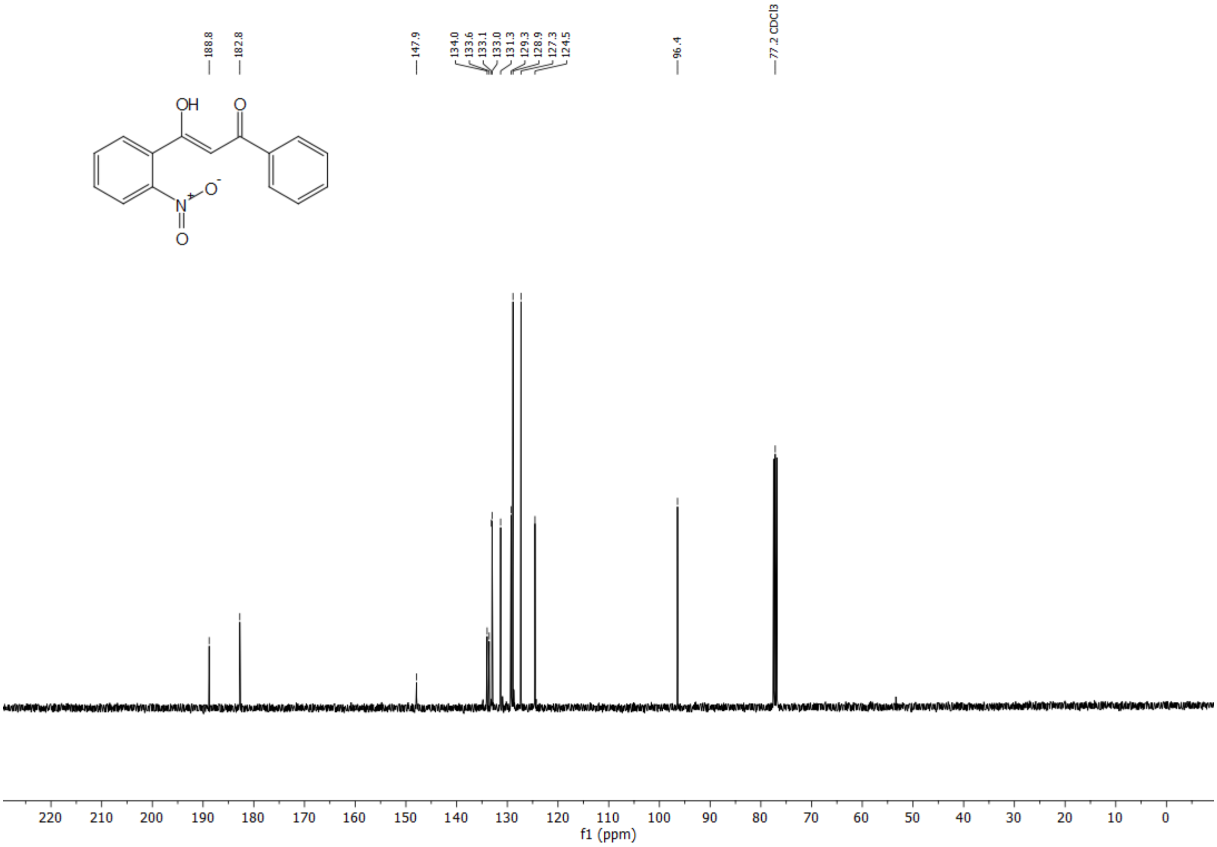

^{1}H NMR spectrum (400 MHz, $CDCl_3$) of **4i**

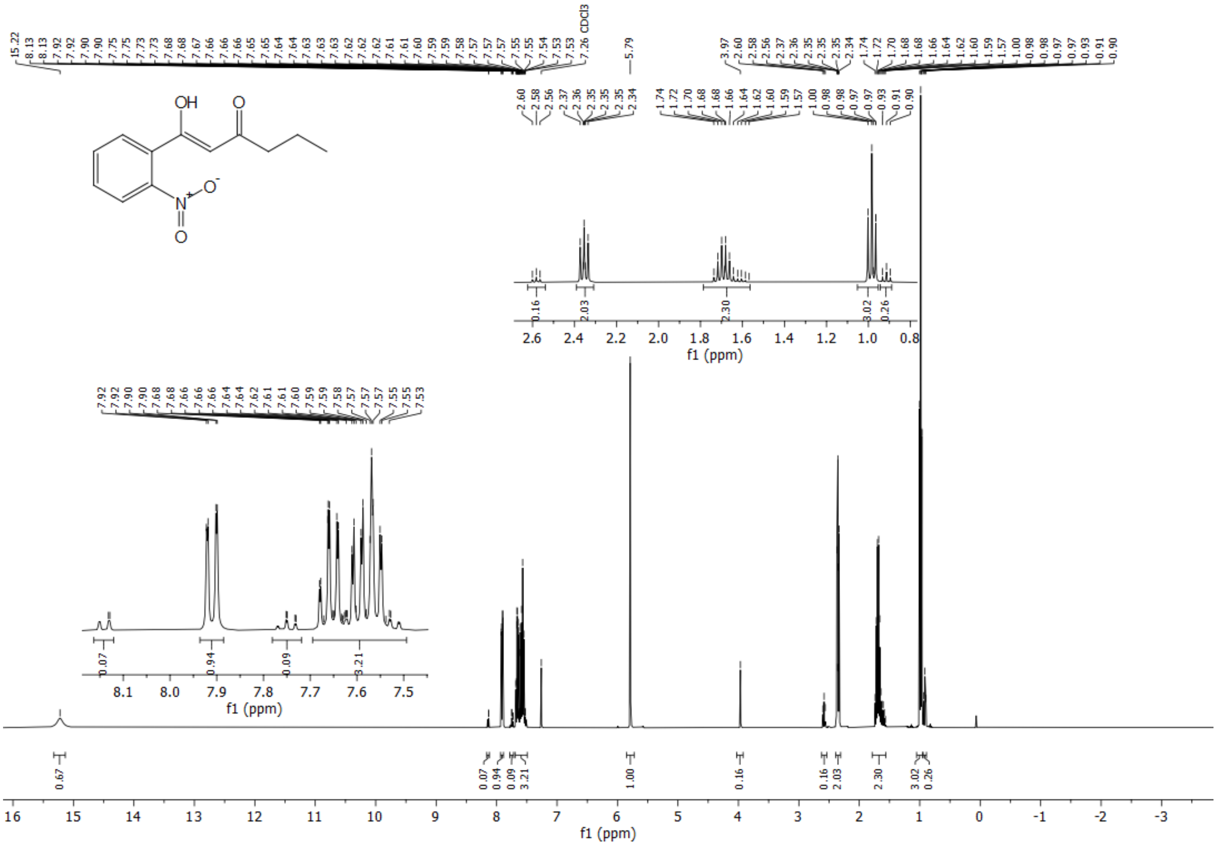

^{13}C NMR spectrum (101 MHz, $CDCl_3$) of **4i**

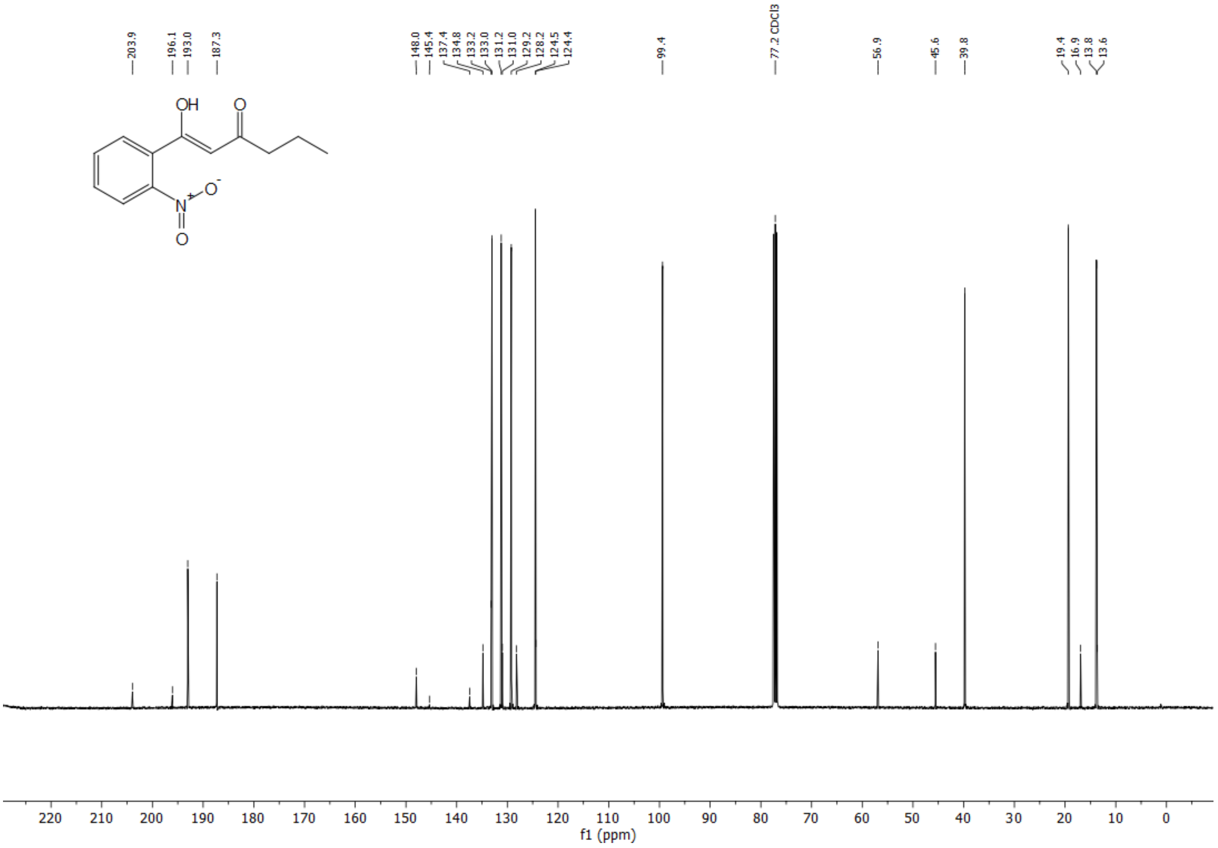

^{1}H NMR spectrum (400 MHz, $CDCl_3$) of **4j**

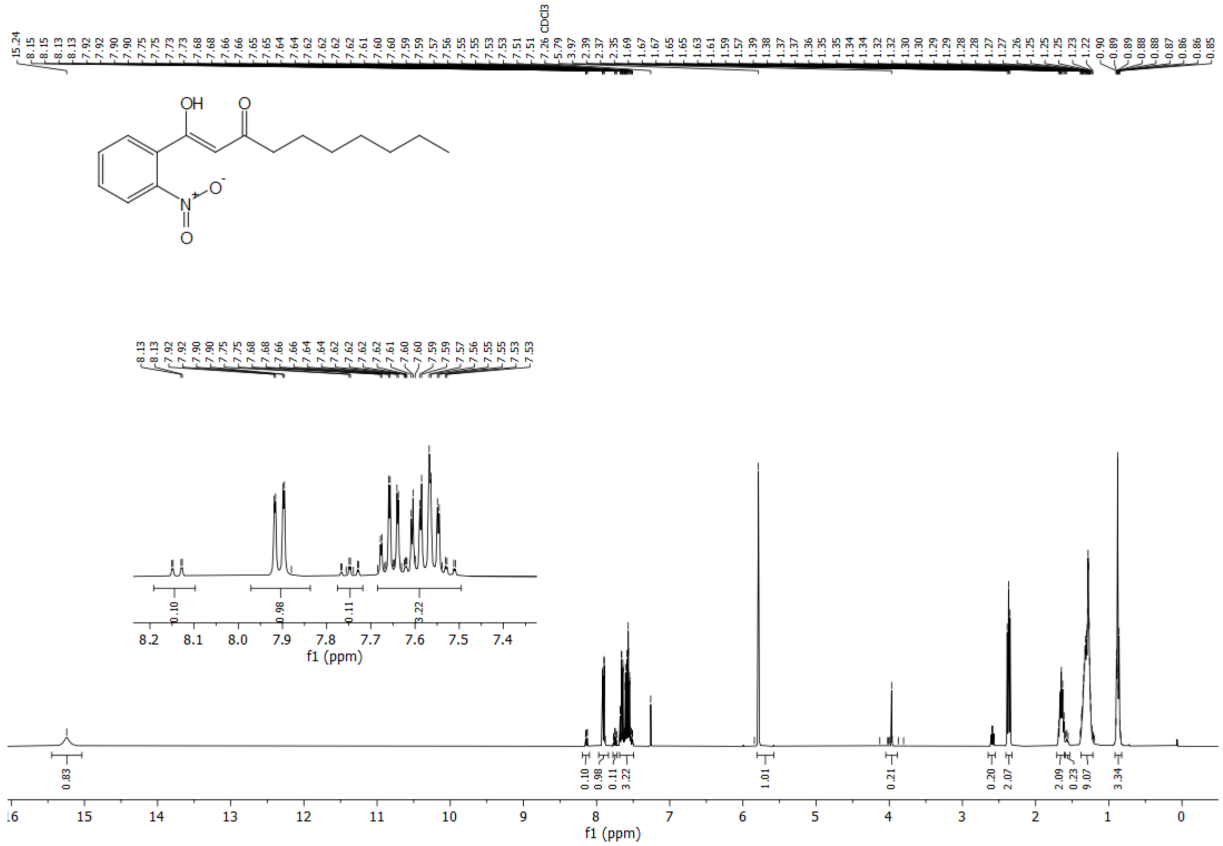

^{13}C NMR spectrum (101 MHz, $CDCl_3$) of **4j**

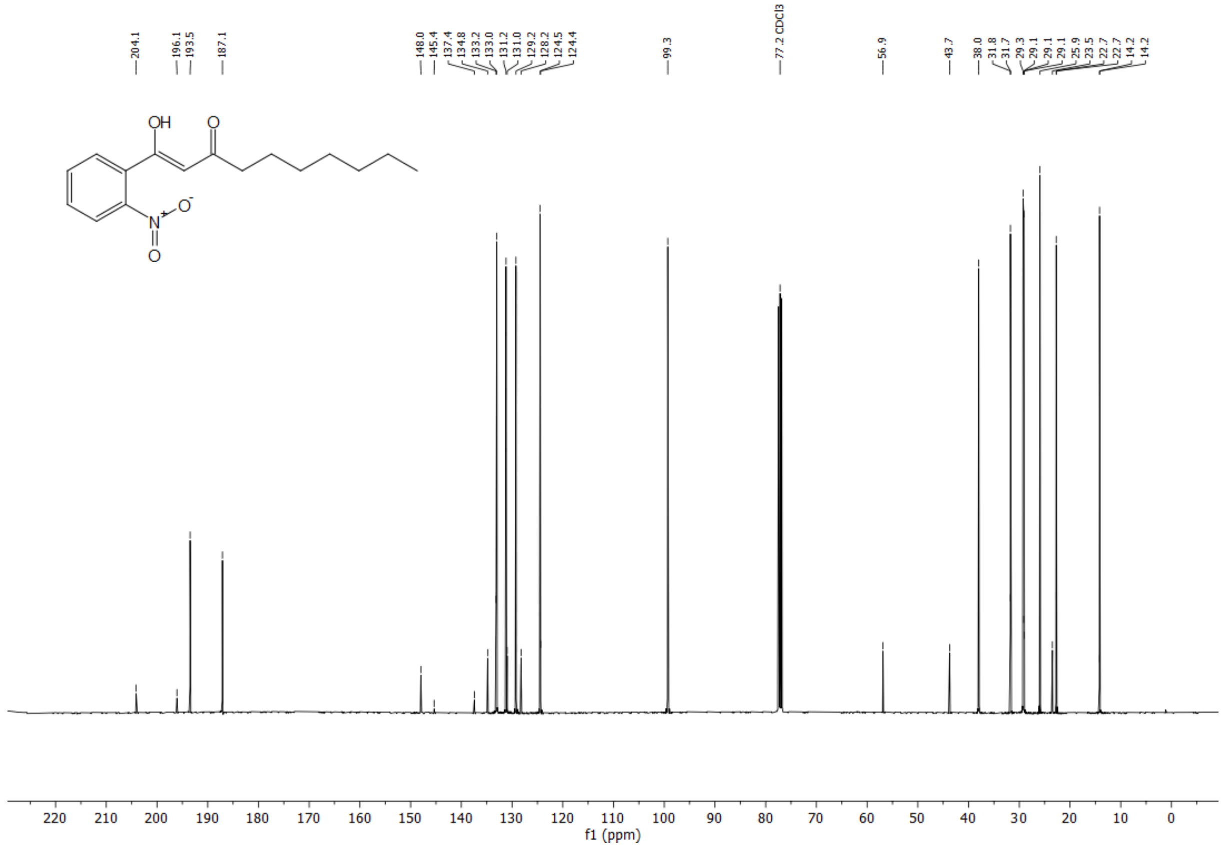

^{1}H NMR spectrum (400 MHz, $CDCl_3$) of **4k**

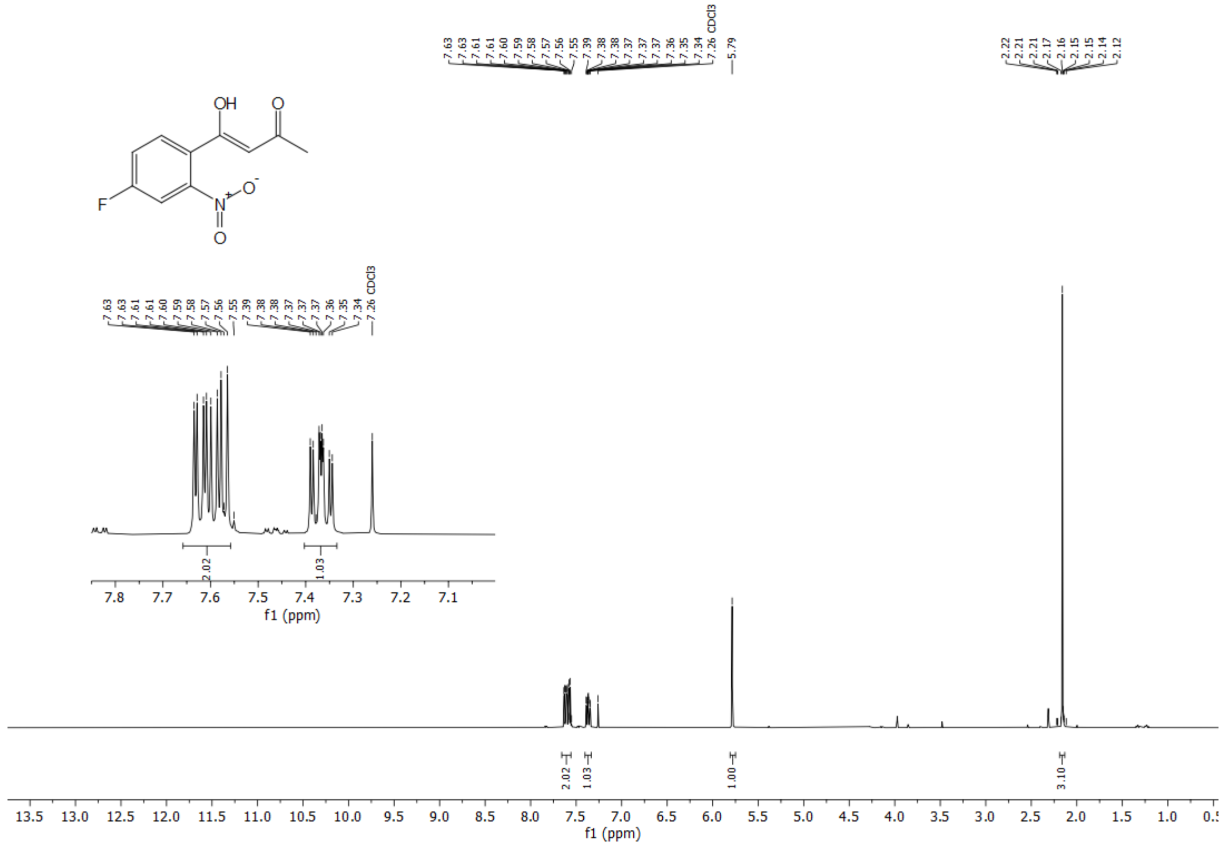

^{13}C NMR spectrum (101 MHz, $CDCl_3$) of **4k**

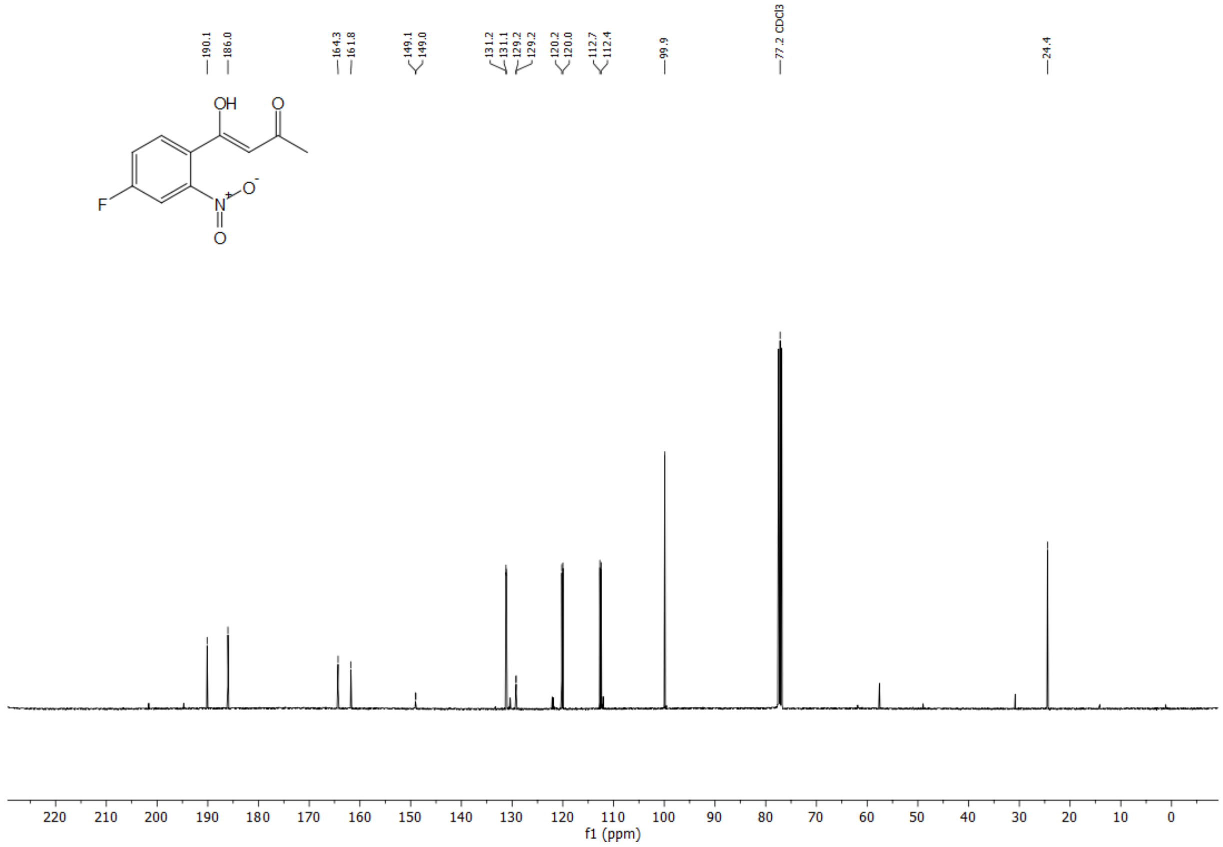

^{19}F NMR spectrum (376 MHz, CDCl$_3$) of **4k**

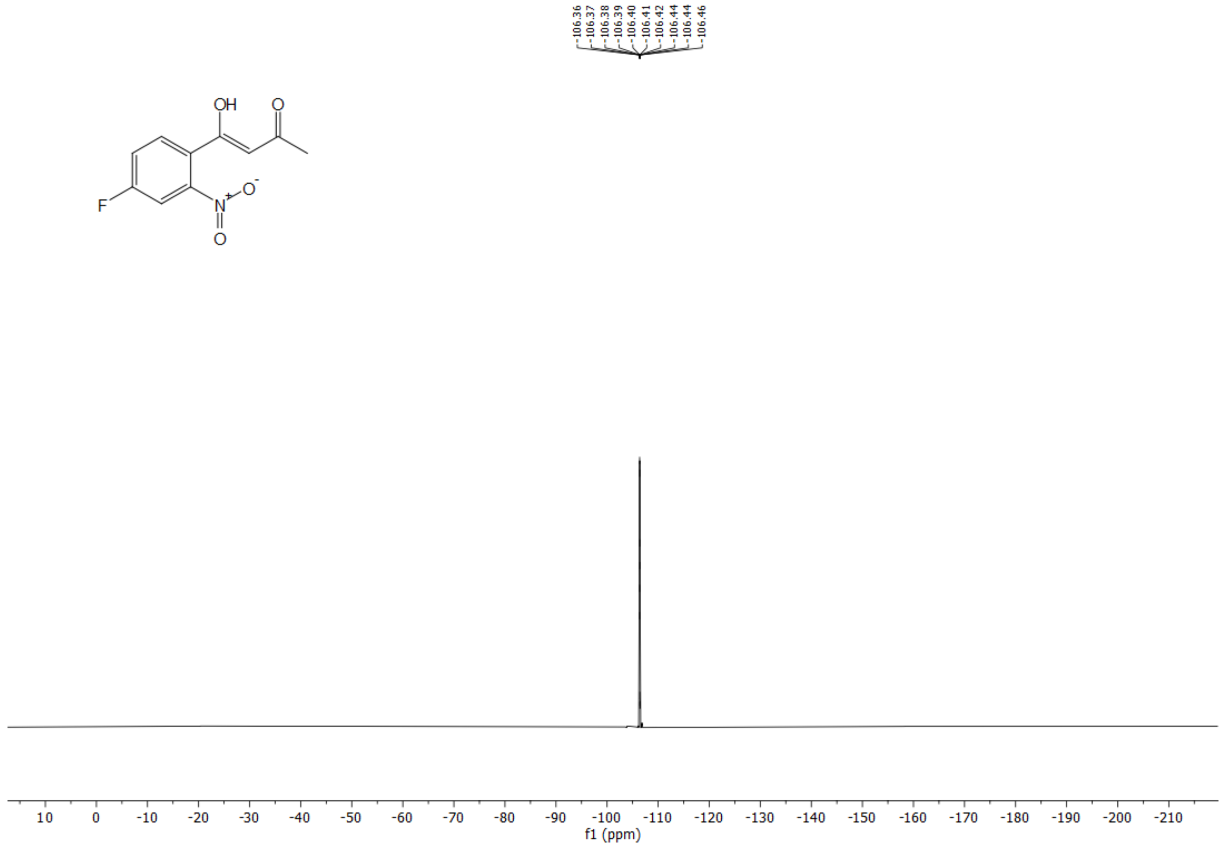

^{1}H NMR spectrum (400 MHz, CDCl$_3$) of **4l**

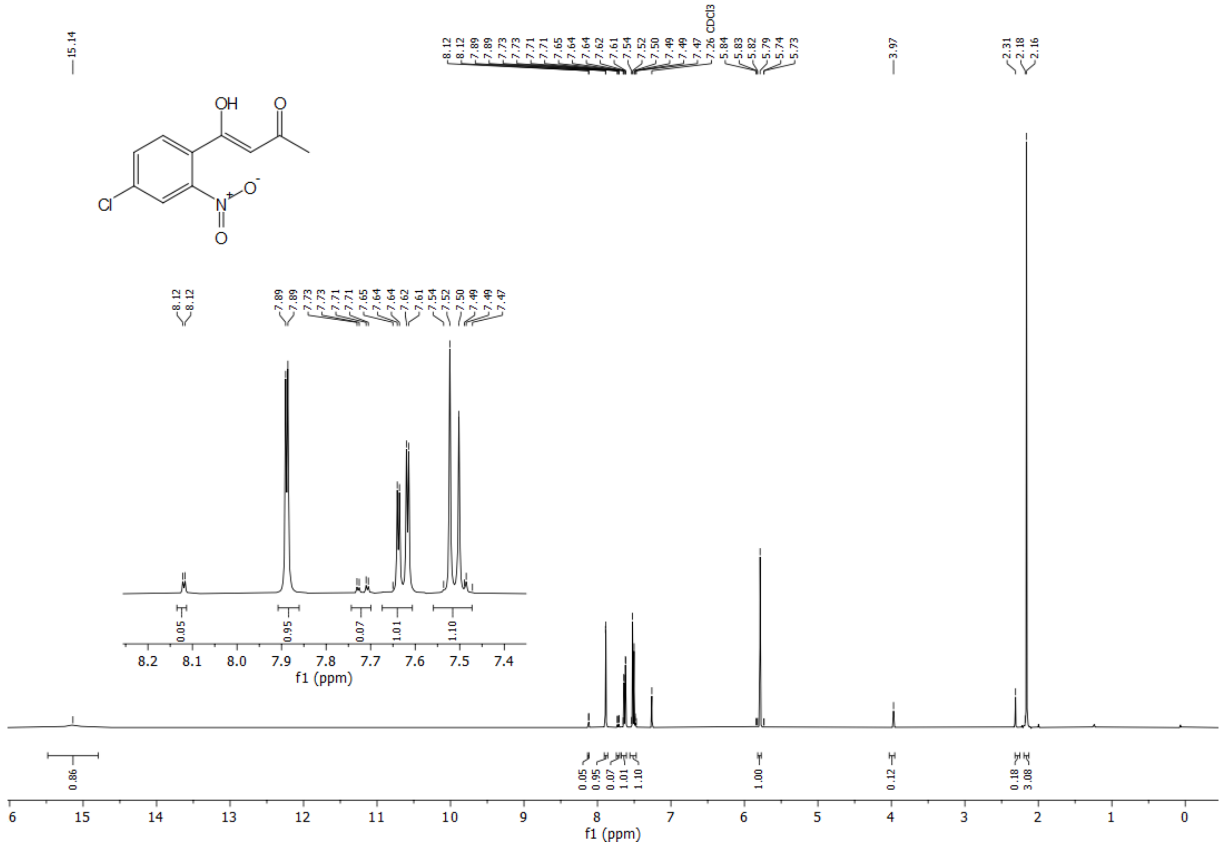

^{13}C NMR spectrum (101 MHz, $CDCl_3$) of **4l**

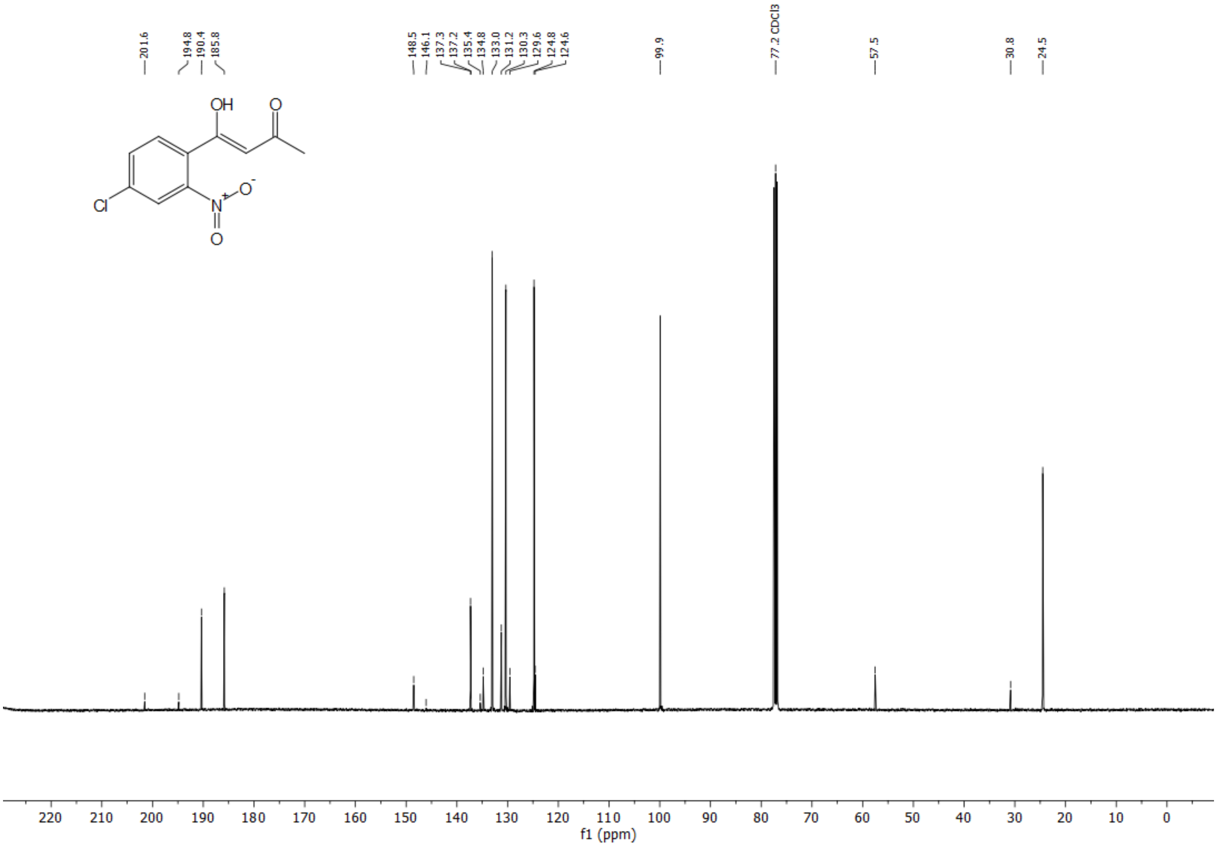

^{1}H NMR spectrum (400 MHz, $CDCl_3$) of **4m**

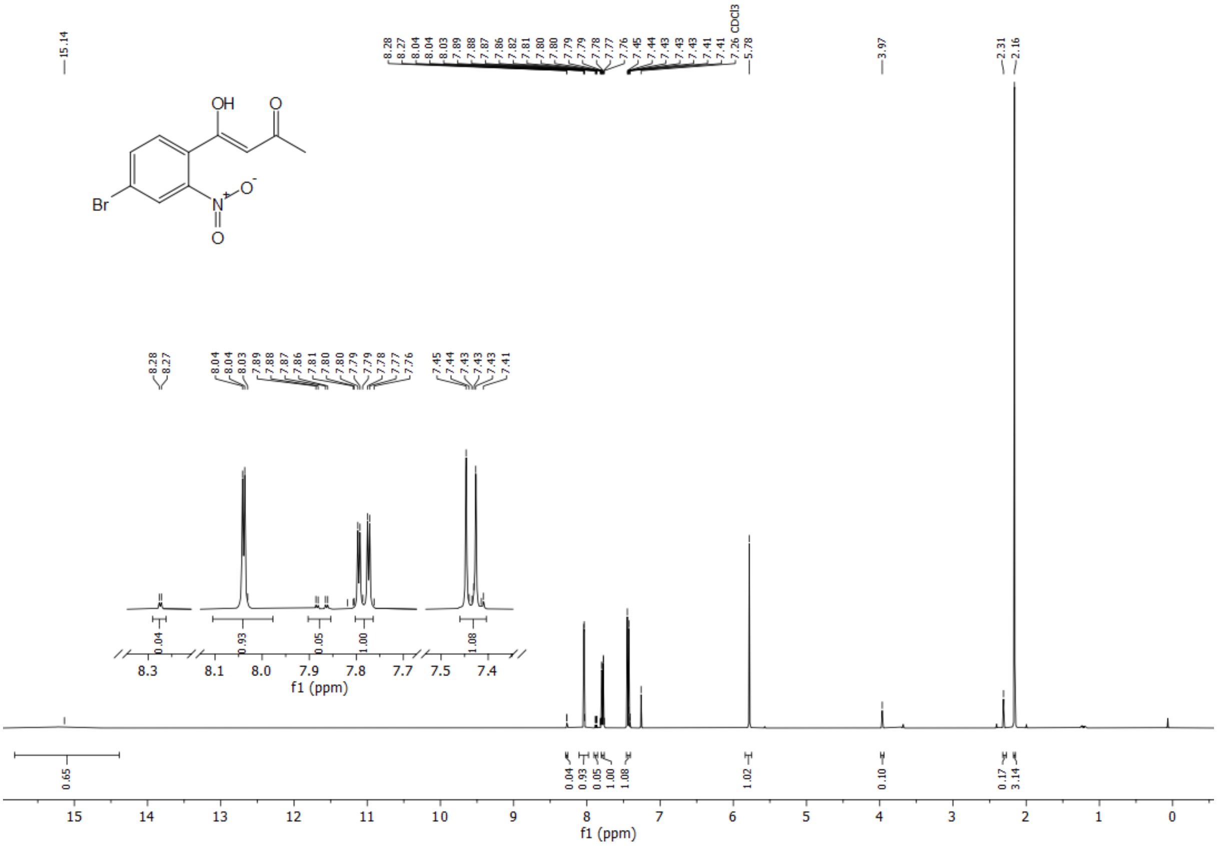

^{13}C NMR spectrum (101 MHz, $CDCl_3$) of **4m**

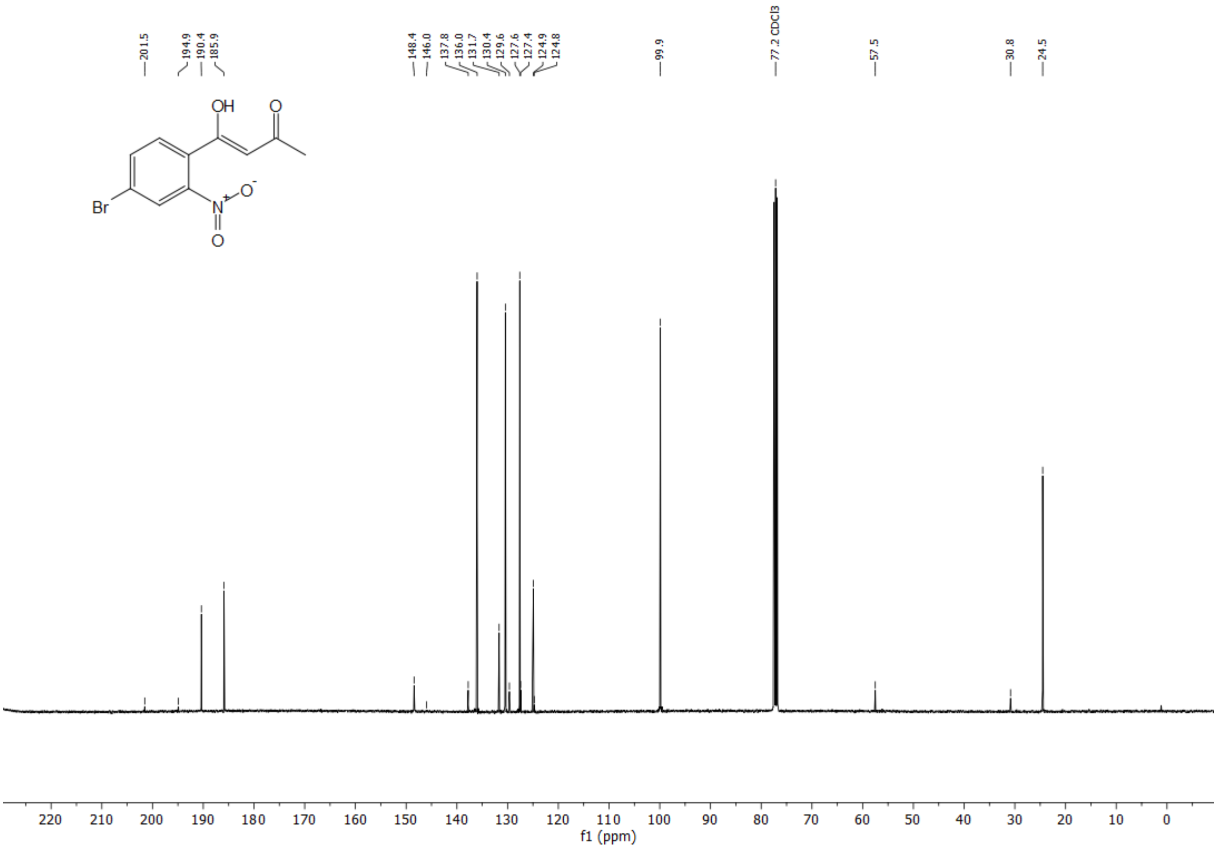

^{1}H NMR spectrum (400 MHz, $CDCl_3$) of **4n**

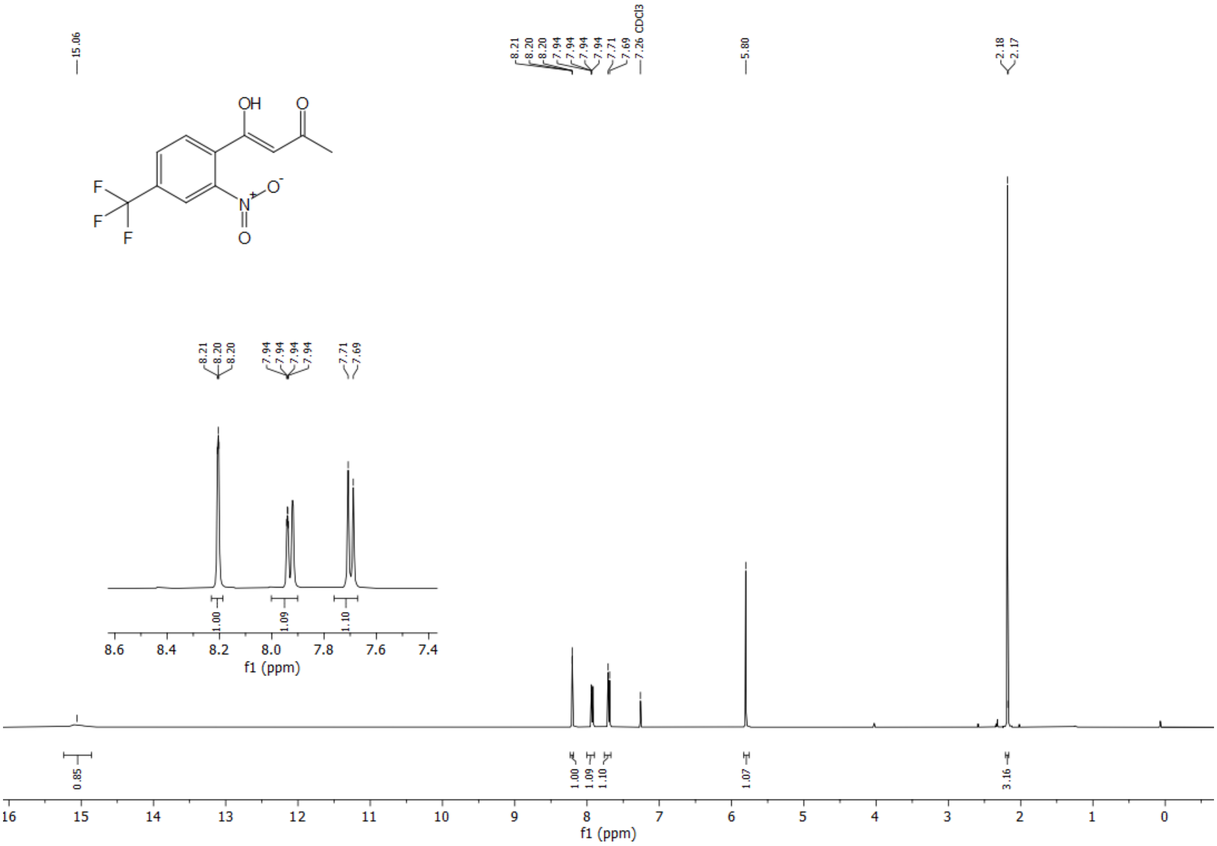

^{13}C NMR spectrum (101 MHz, $CDCl_3$) of **4n**

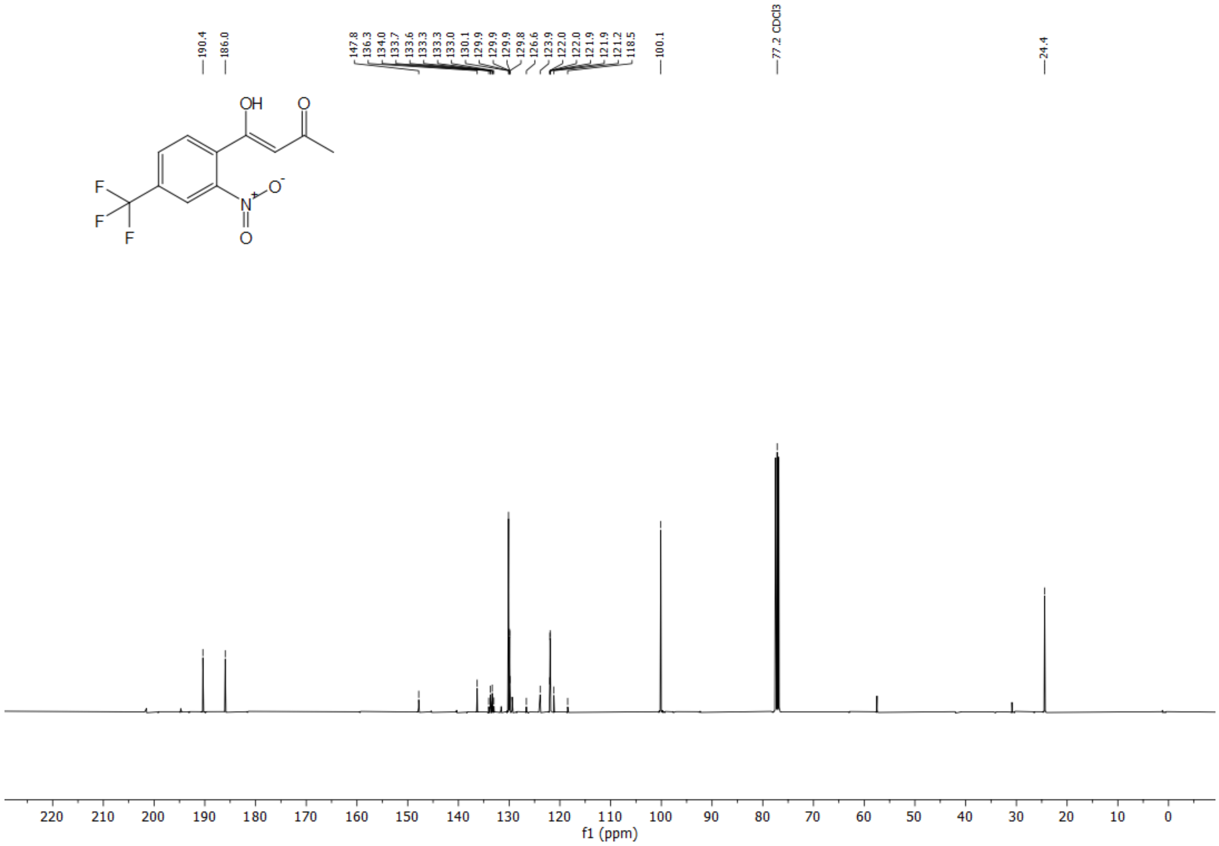

^{19}F NMR spectrum (376 MHz, $CDCl_3$) of **4n**

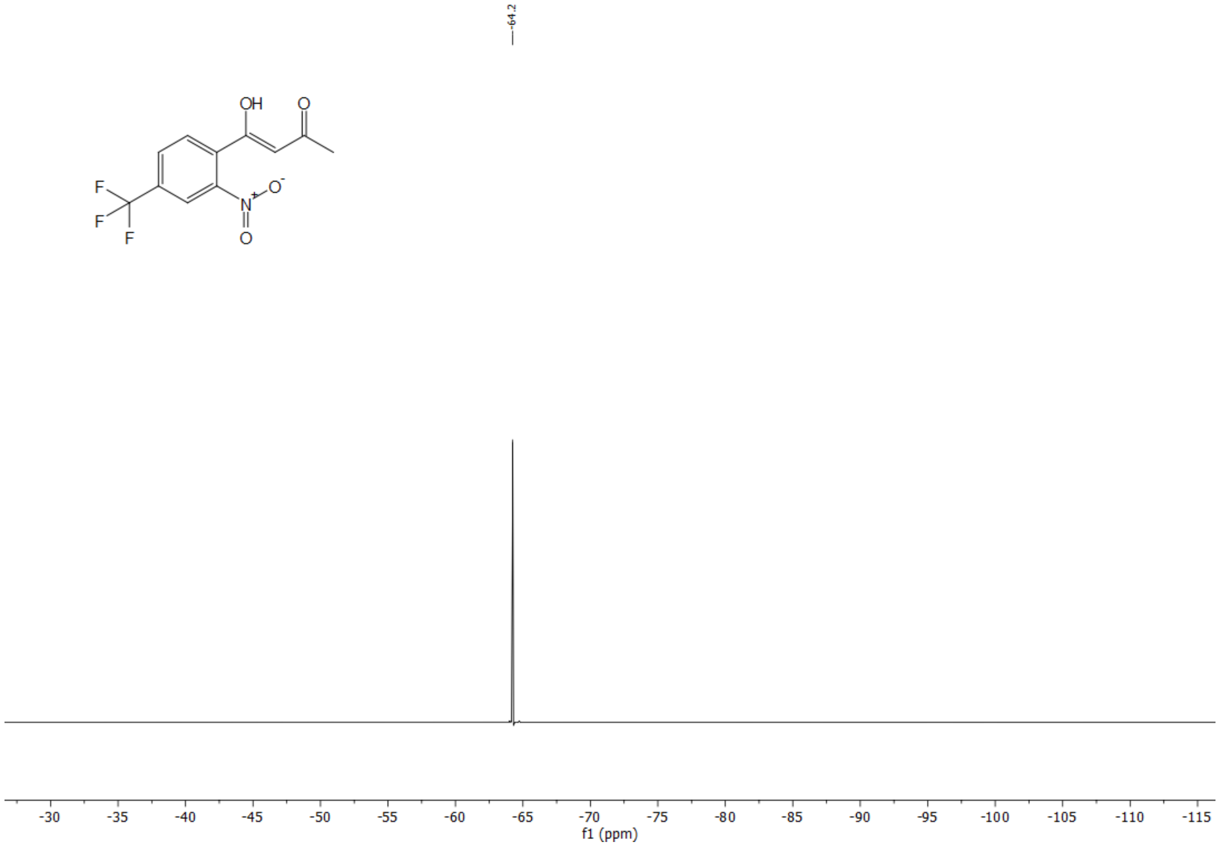

^{1}H NMR spectrum (400 MHz, $CDCl_3$) of **4o**

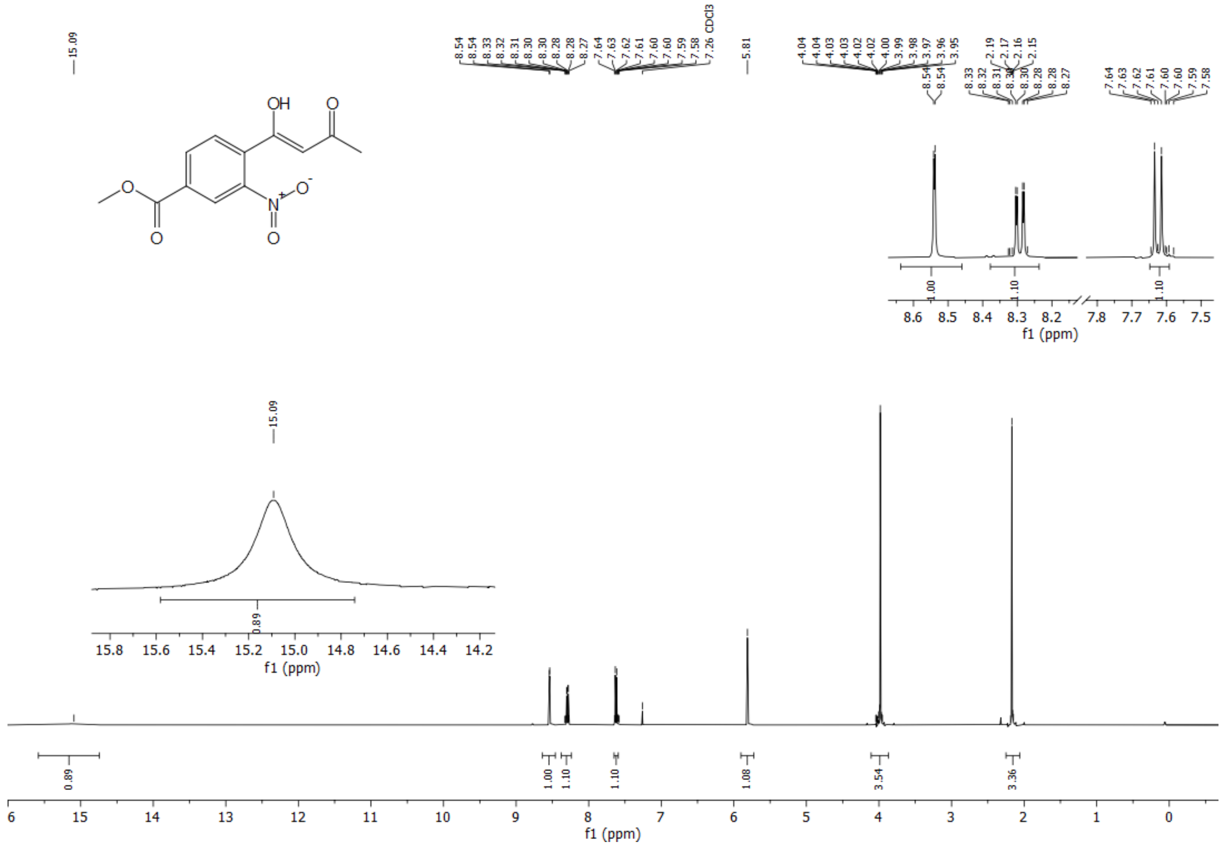

^{13}C NMR spectrum (101 MHz, $CDCl_3$) of **4o**

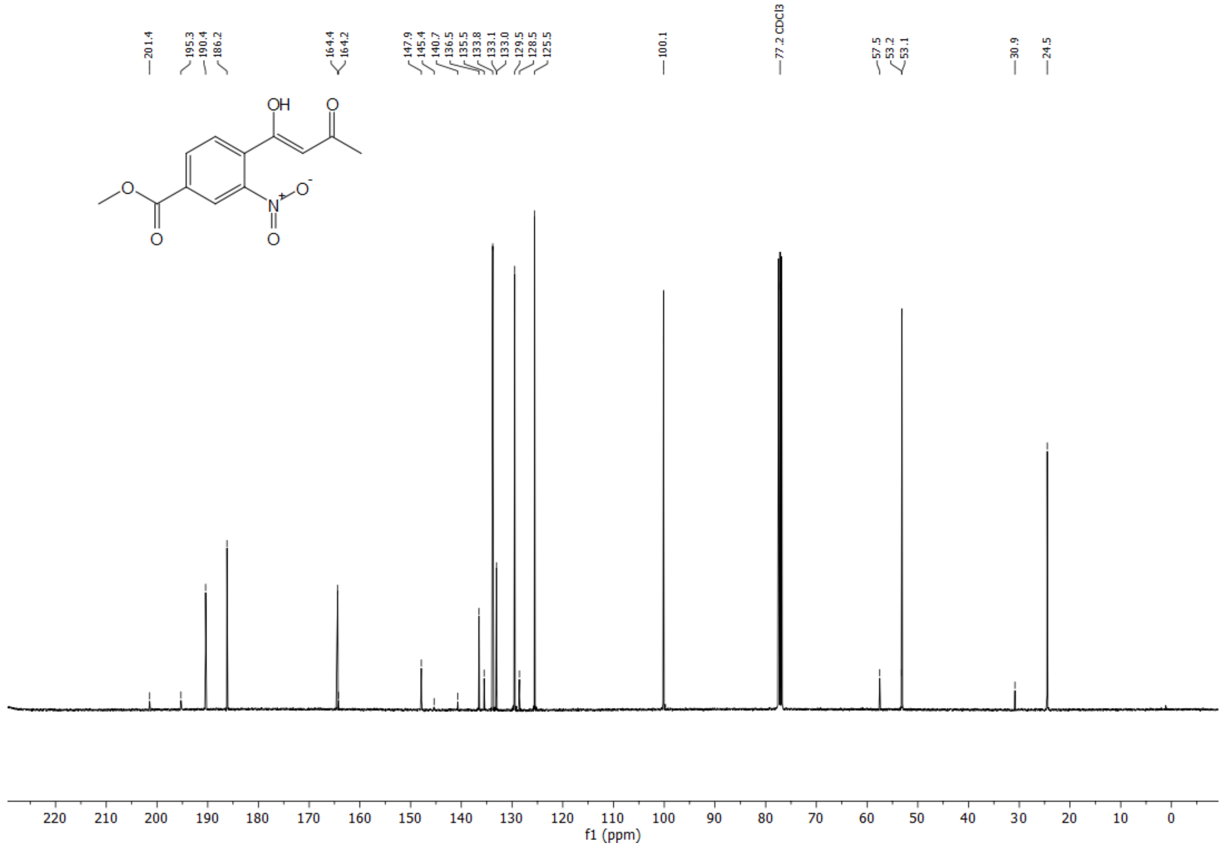

^1H NMR spectrum (400 MHz, $CDCl_3$) of **4p**

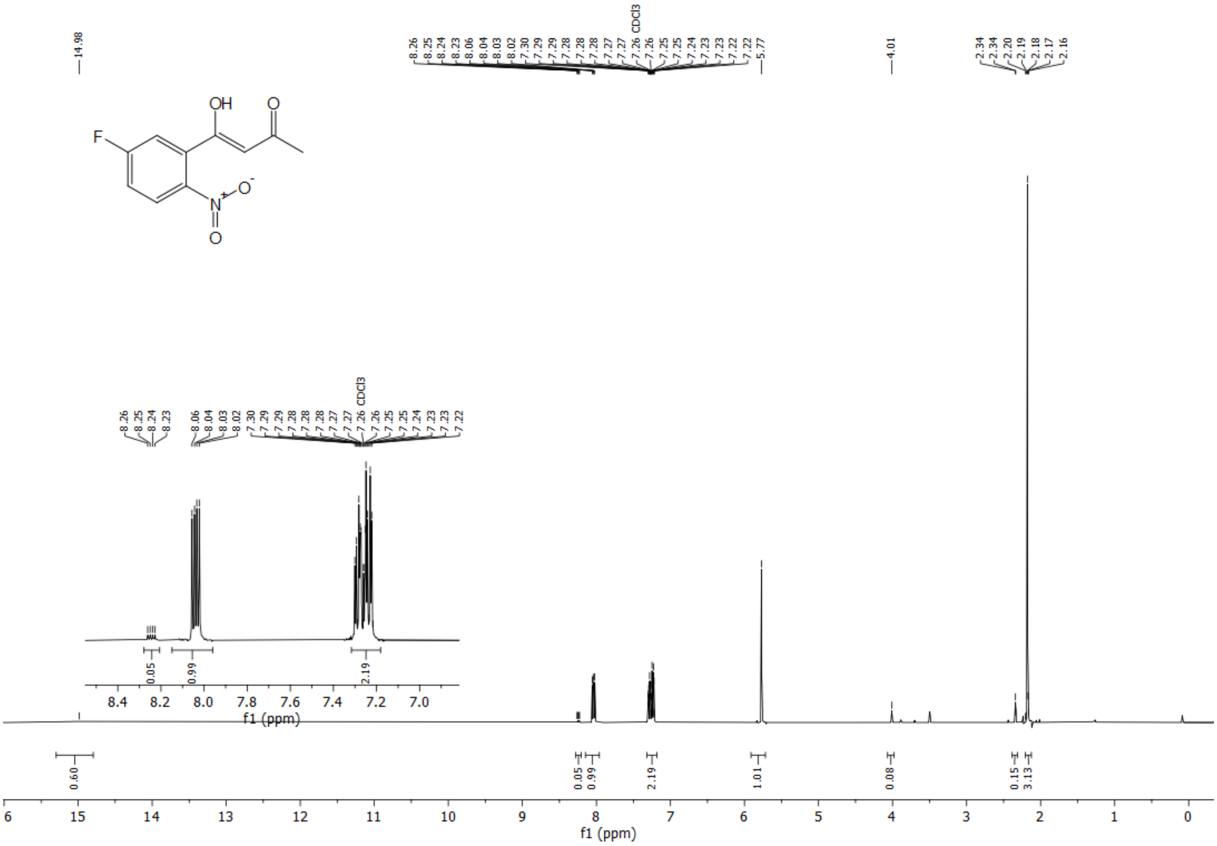

^{13}C NMR spectrum (101 MHz, $CDCl_3$) of **4p**

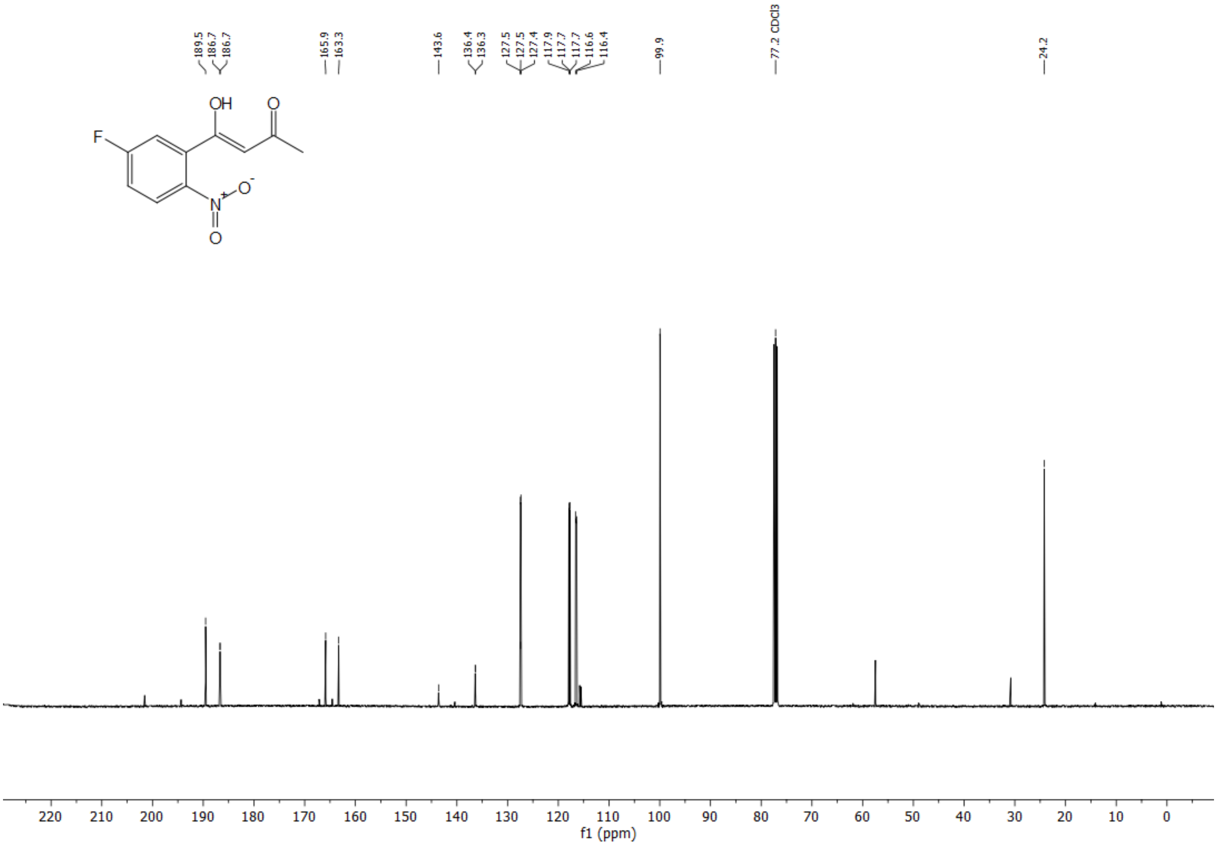

^{19}F NMR spectrum (376 MHz, $CDCl_3$) of **4p**

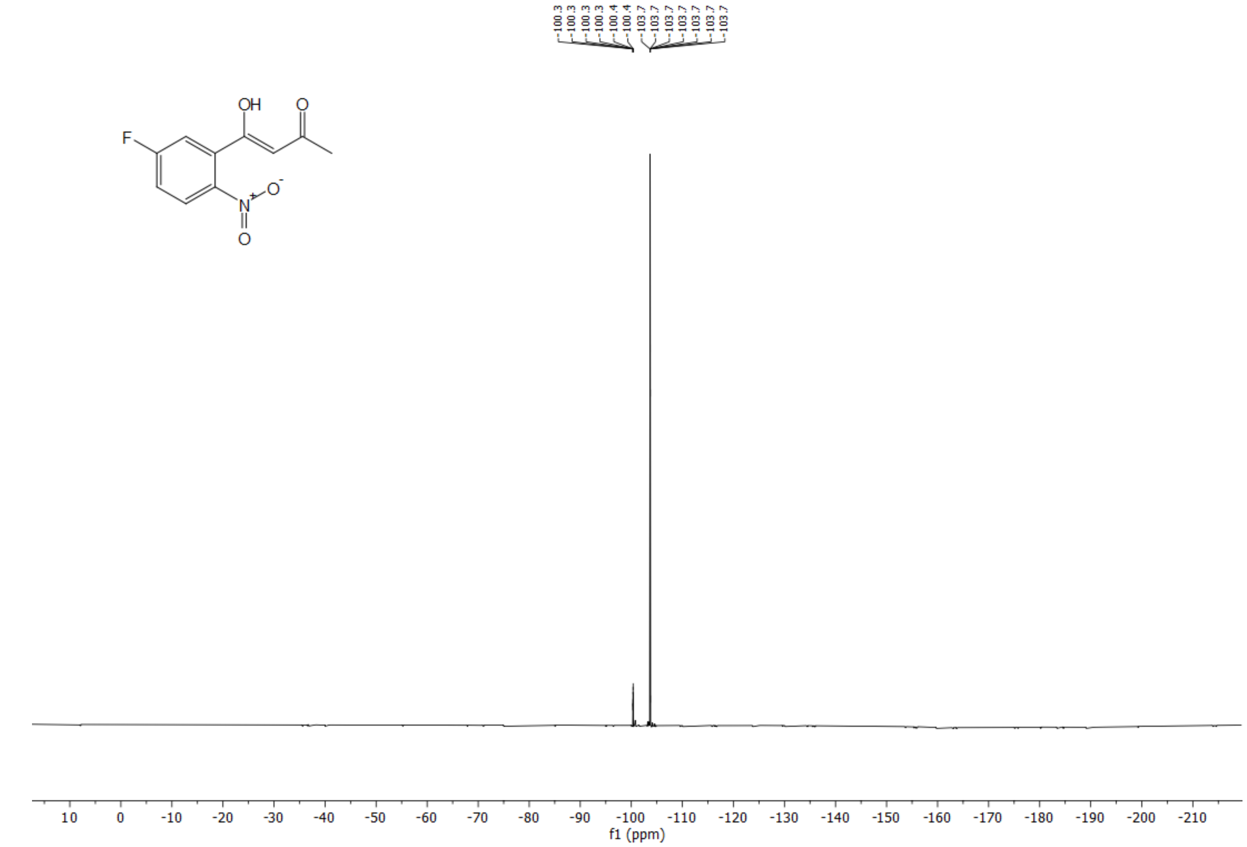

^{1}H NMR spectrum (400 MHz, $CDCl_3$) of **4q**

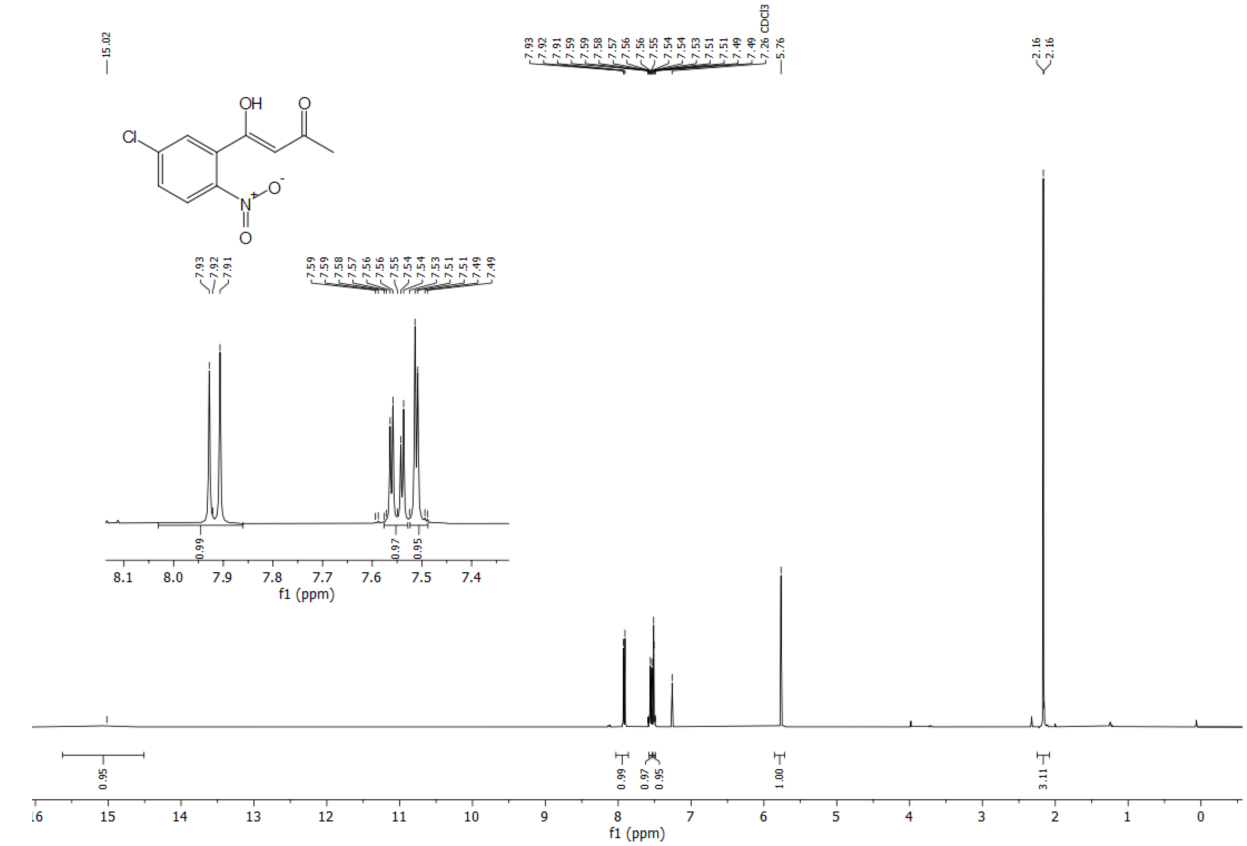

^{13}C NMR spectrum (101 MHz, $CDCl_3$) of **4q**

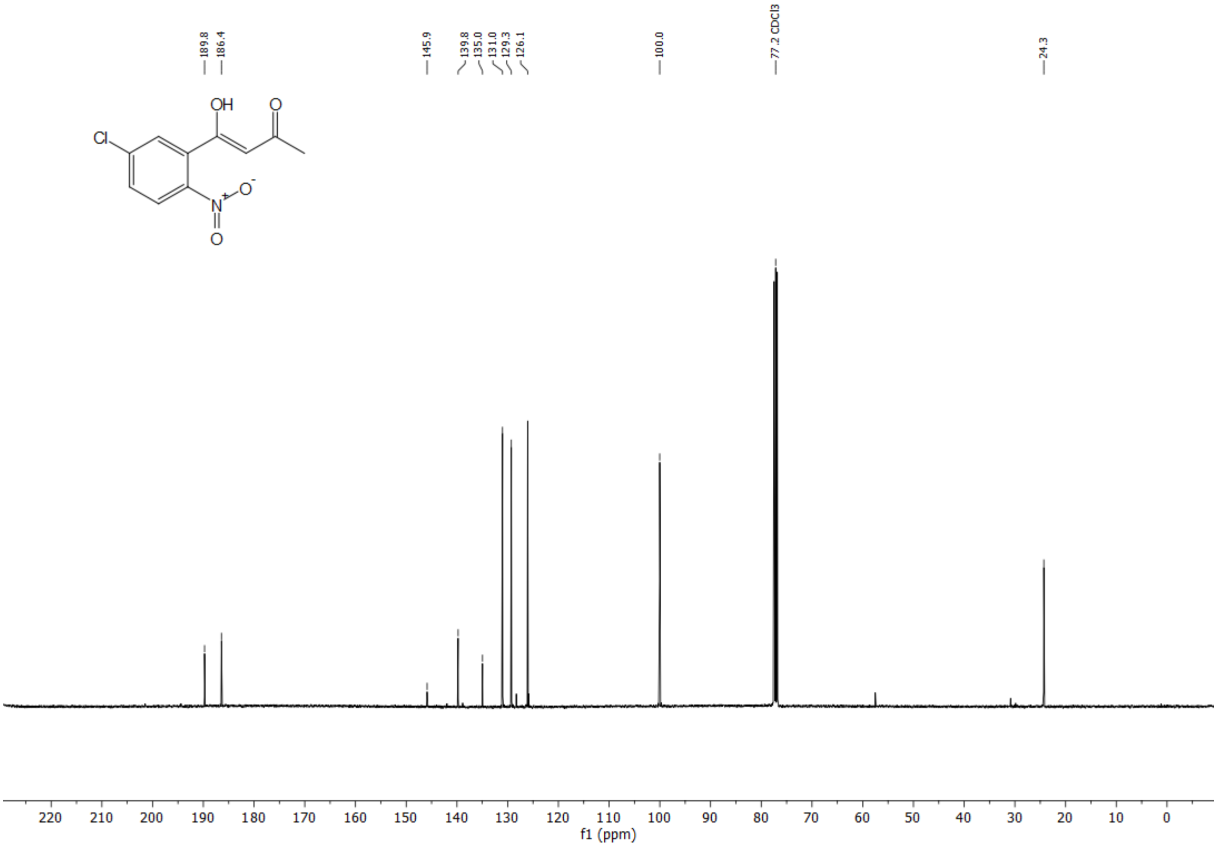

^{1}H NMR spectrum (400 MHz, $CDCl_3$) of **4r**

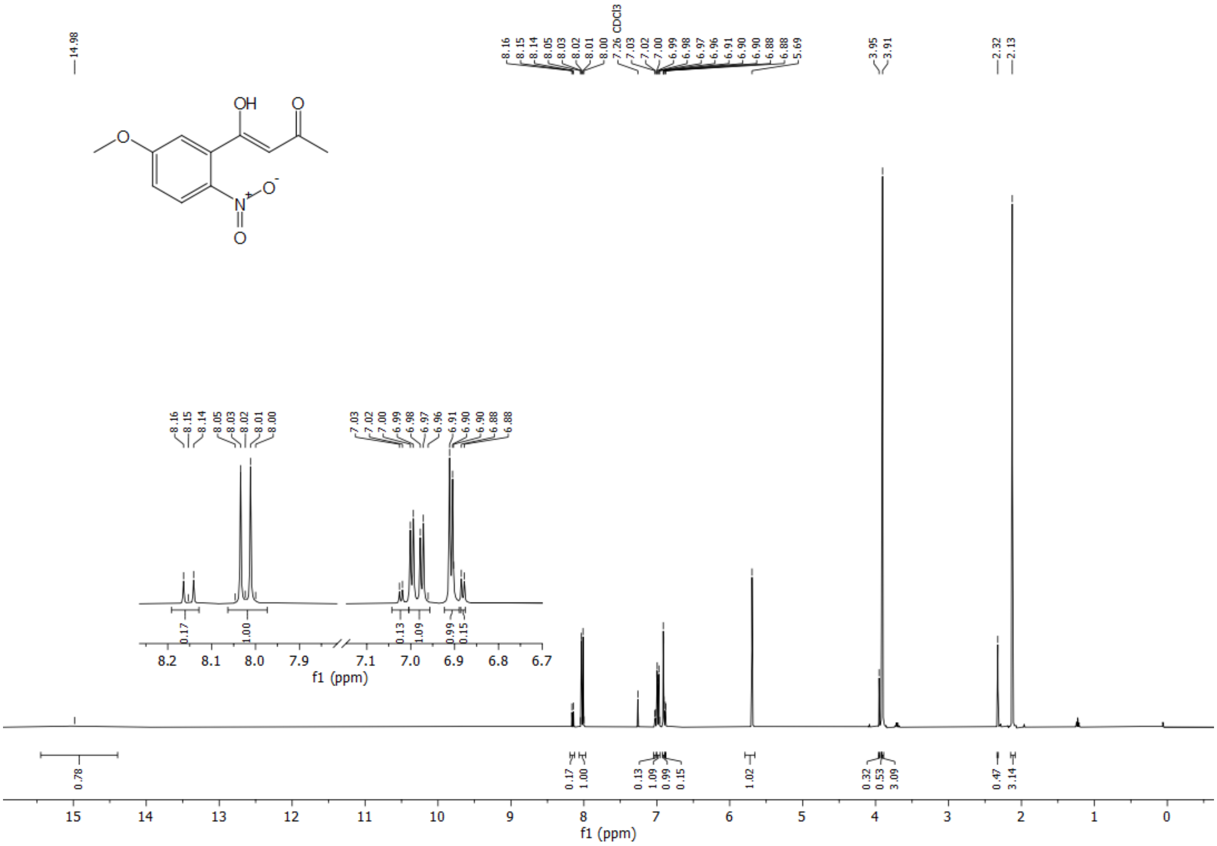

^{13}C NMR spectrum (101 MHz, CDCl$_3$) of **4r**

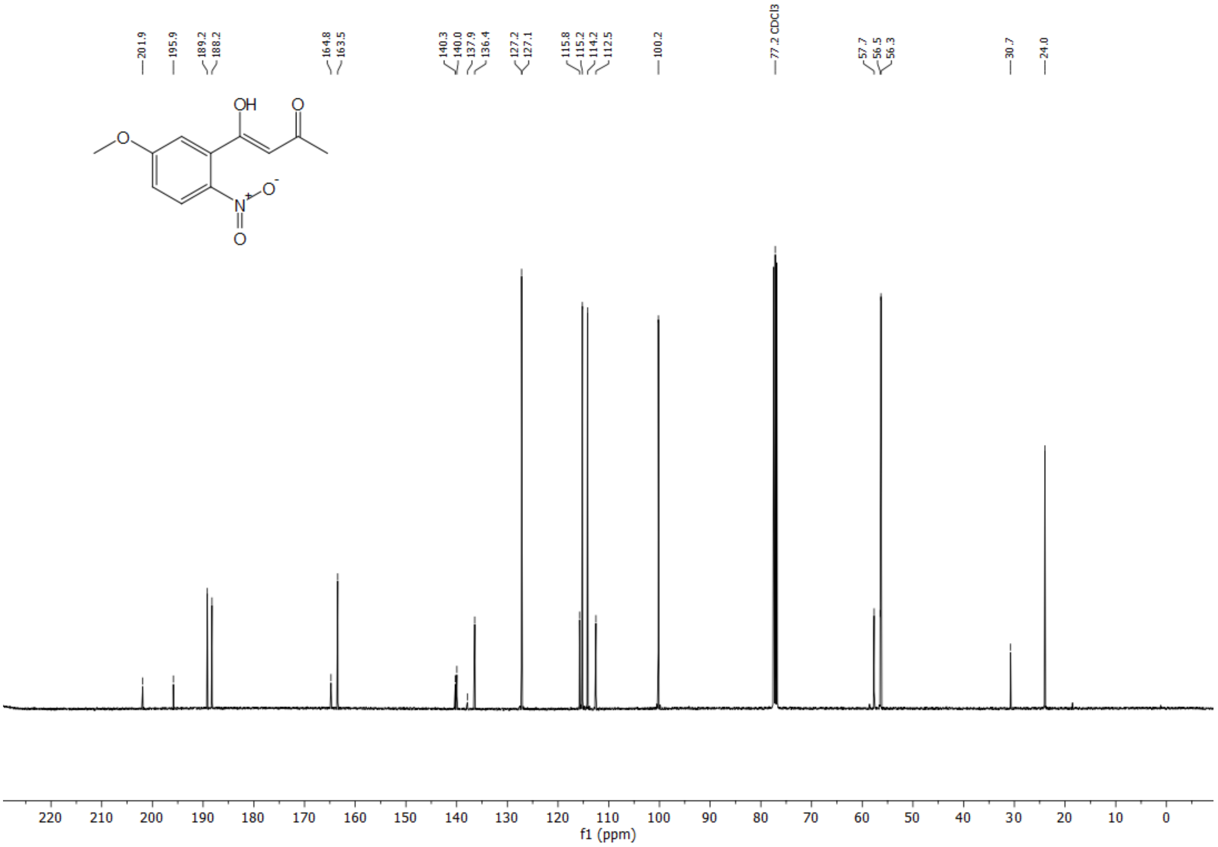

^{1}H NMR spectrum (400 MHz, CDCl$_3$) of **4s**

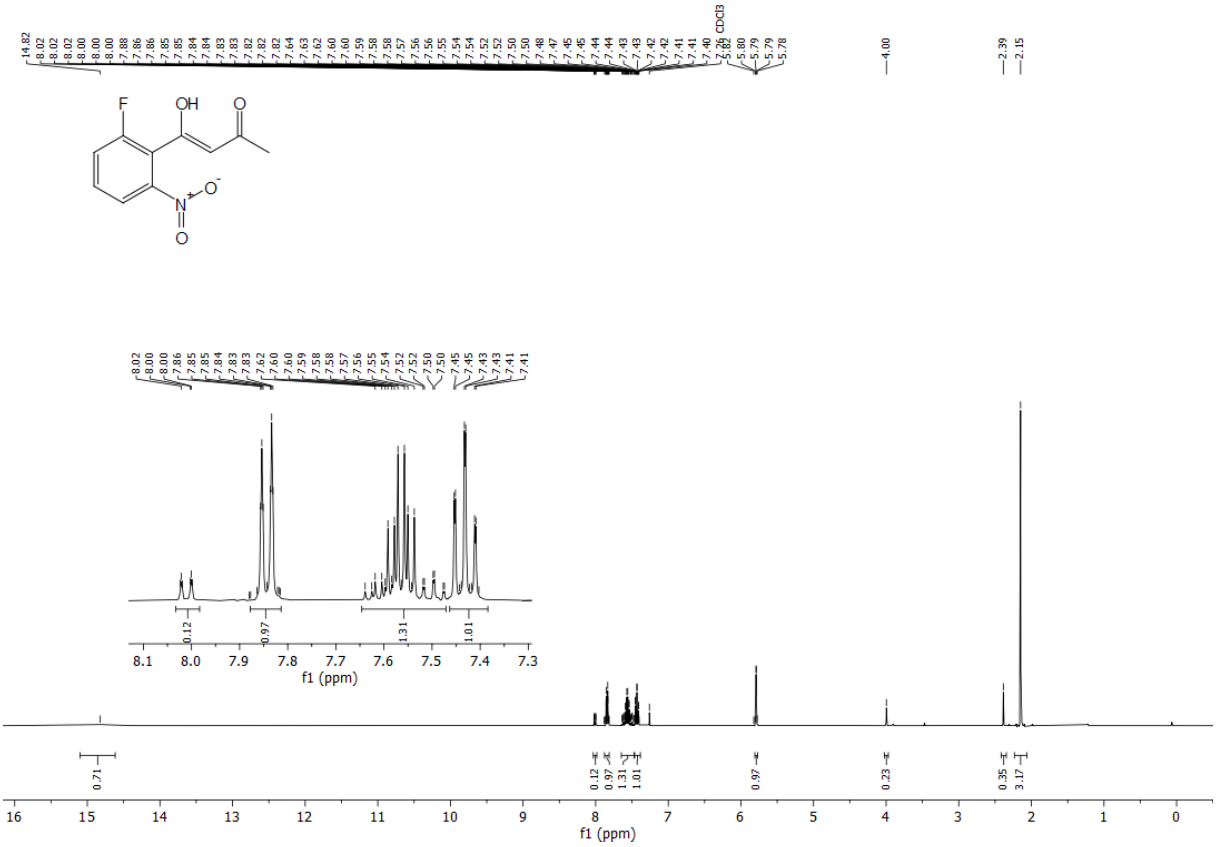

^{13}C NMR spectrum (101 MHz, $CDCl_3$) of **4s**

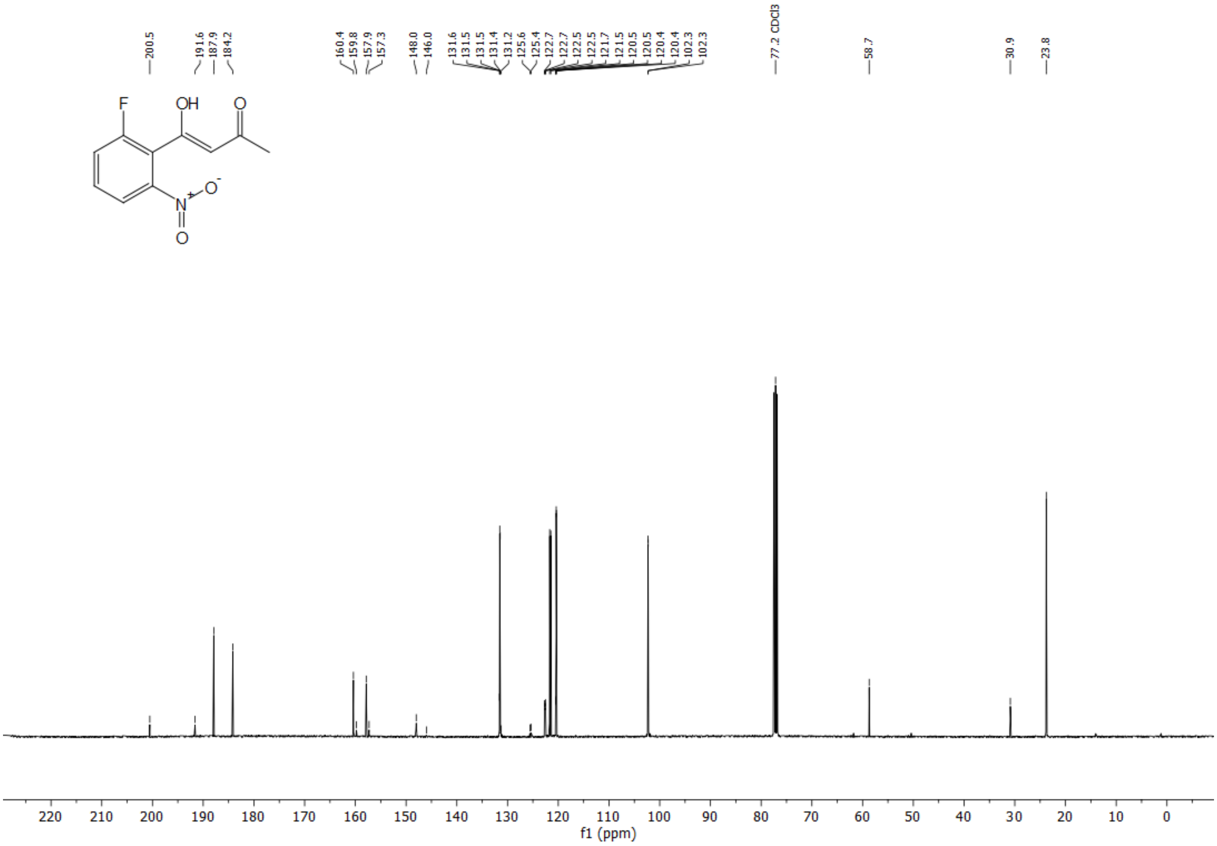

^{19}F NMR spectrum (376 MHz, $CDCl_3$) of **4s**

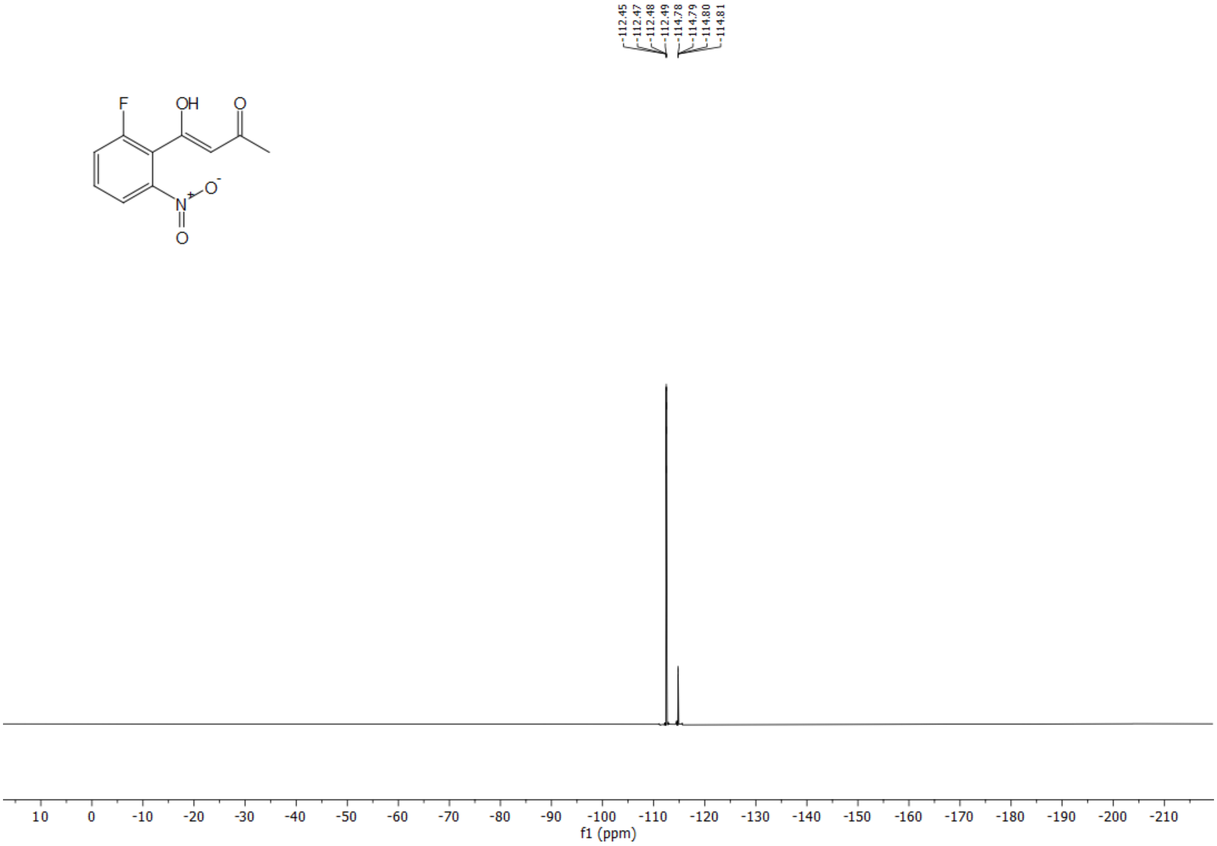

^{1}H NMR spectrum (400 MHz, $CDCl_3$) of **4t**

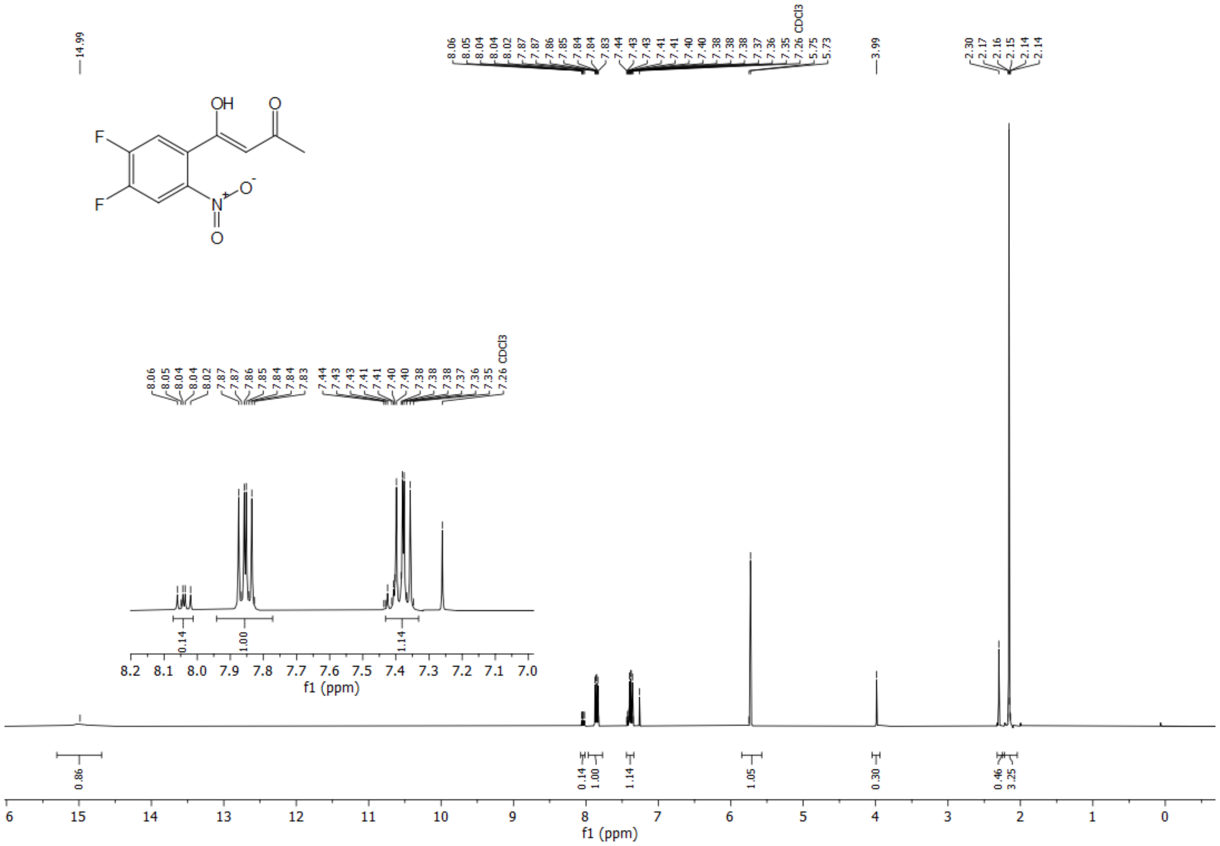

^{13}C NMR spectrum (101 MHz, $CDCl_3$) of **4t**

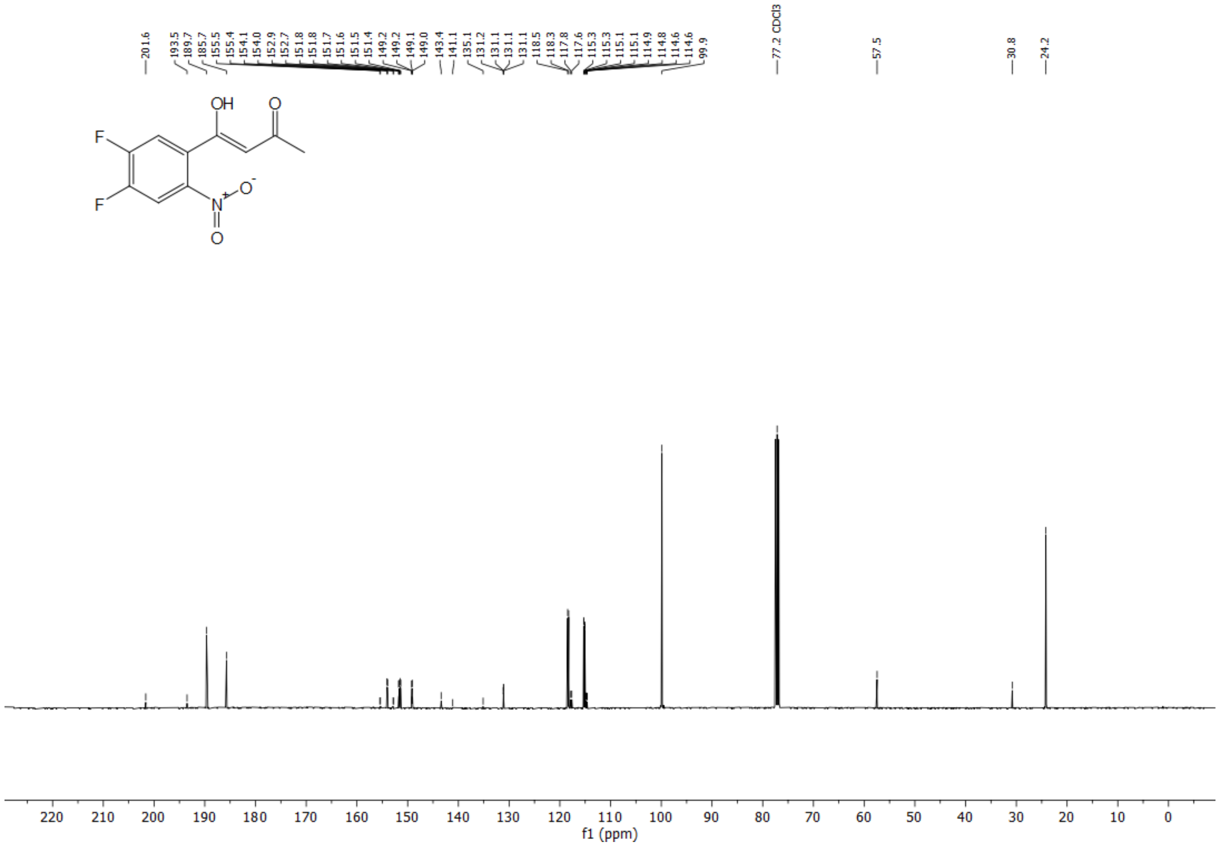

^{19}F NMR spectrum (376 MHz, $CDCl_3$) of **4t**

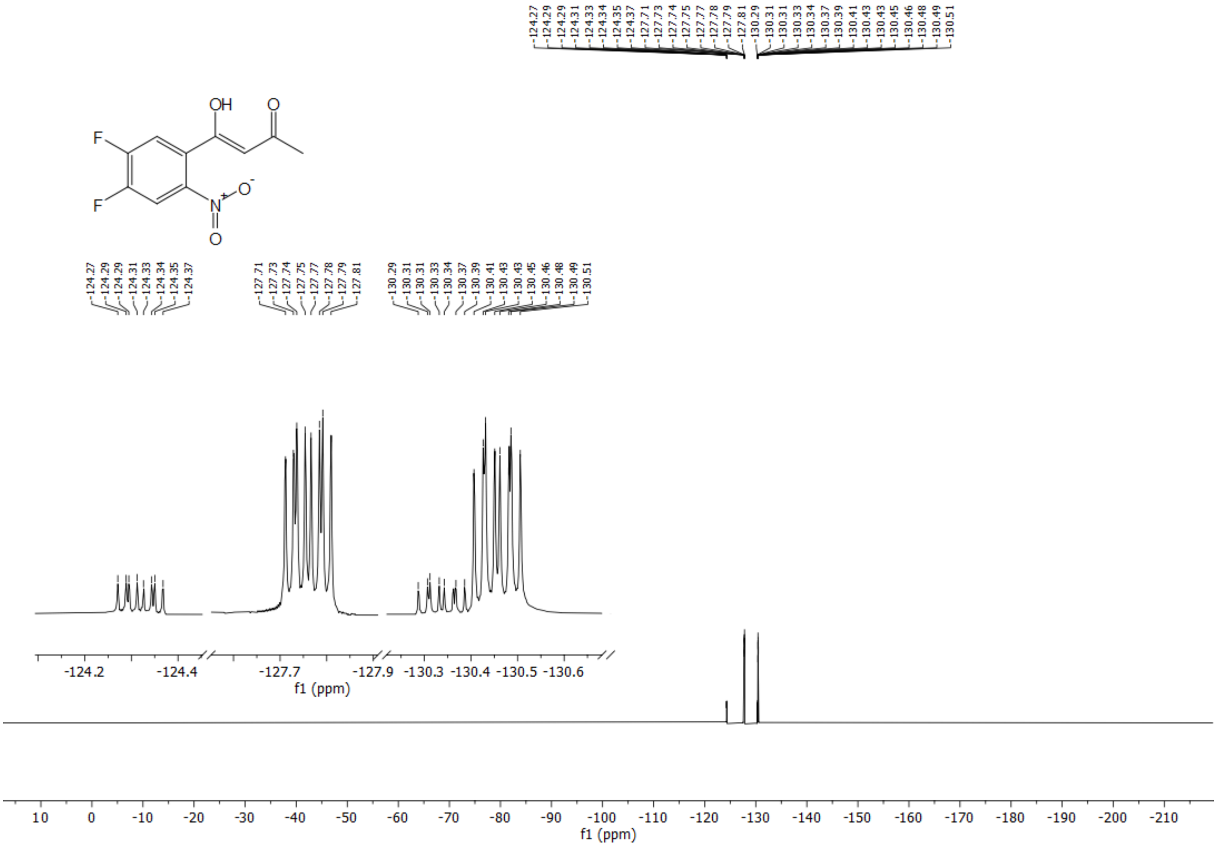

^{1}H NMR spectrum (400 MHz, $CDCl_3$) of **4u**

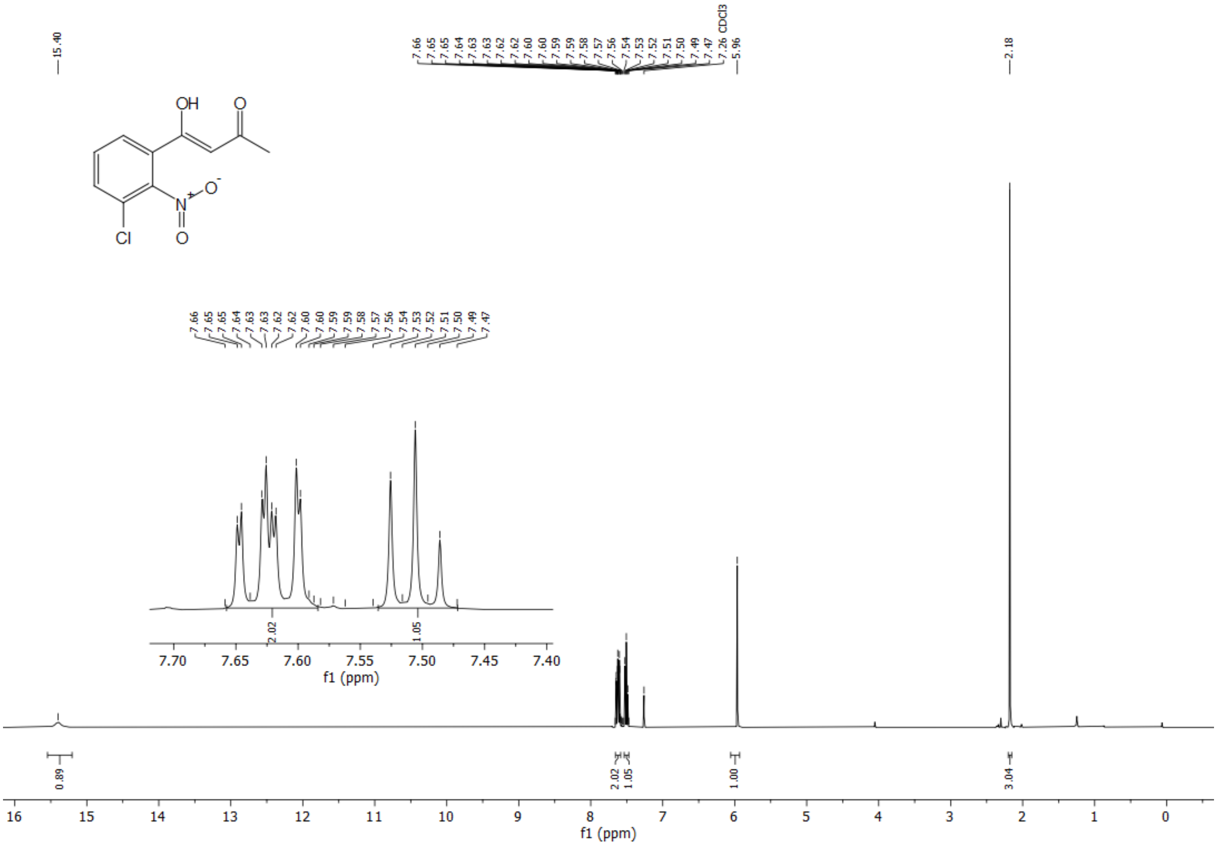

^{13}C NMR spectrum (101 MHz, CDCl$_3$) of **4u**

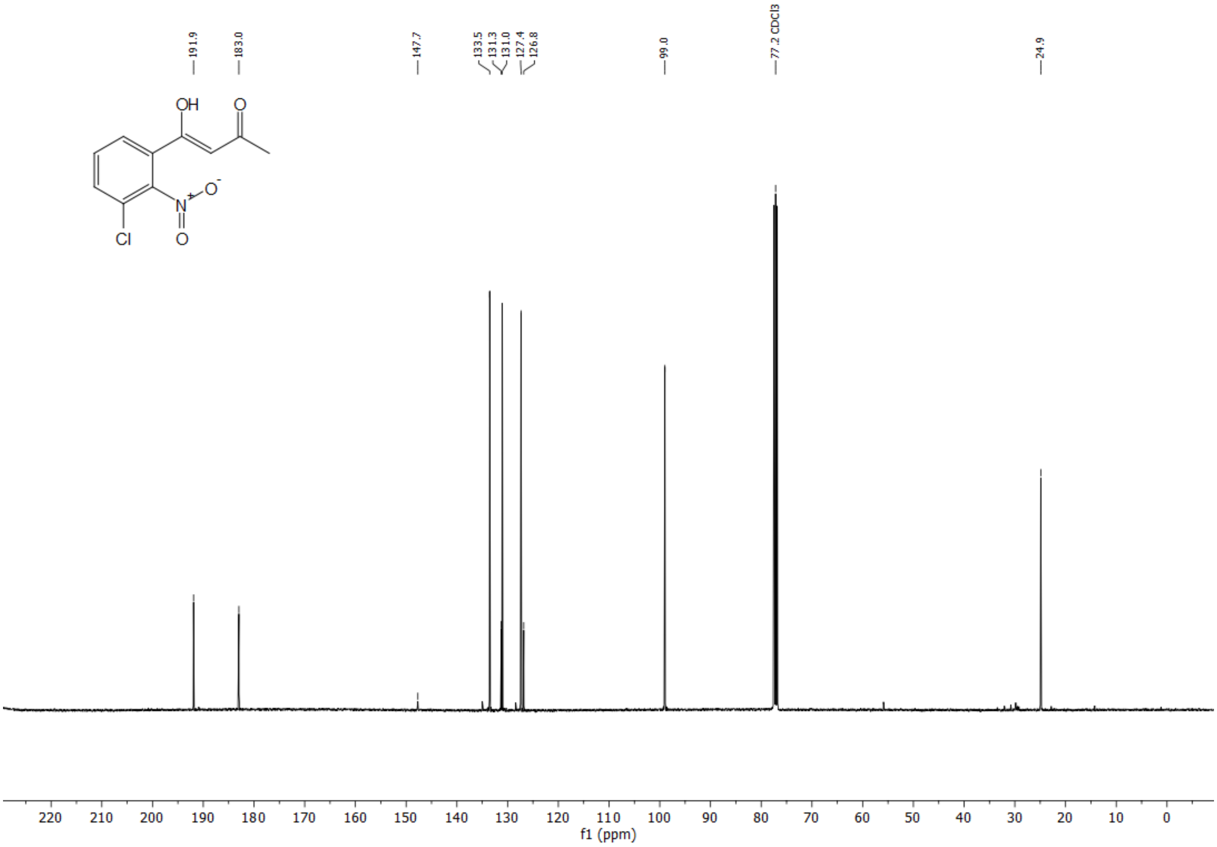

^{1}H NMR spectrum (400 MHz, CDCl$_3$) of **4v**

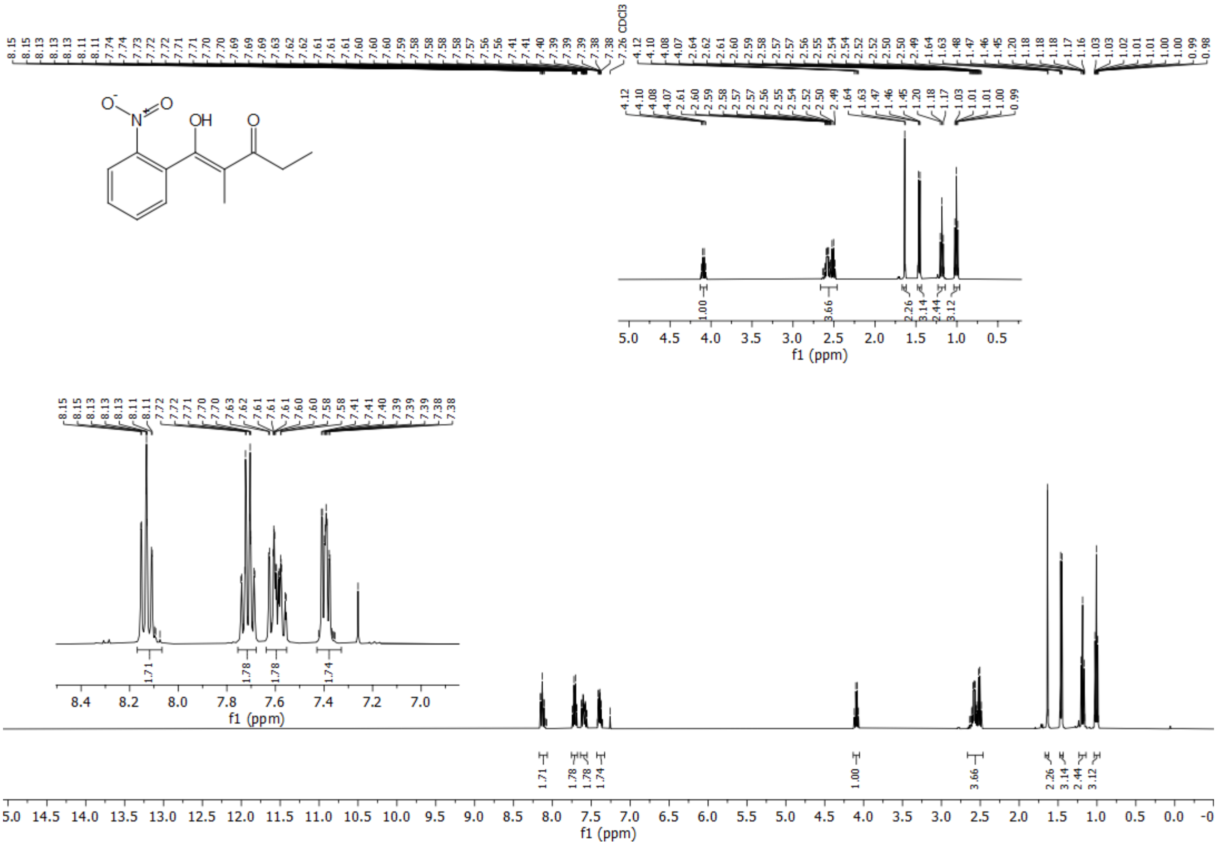

^{13}C NMR spectrum (101 MHz, $CDCl_3$) of **4v**

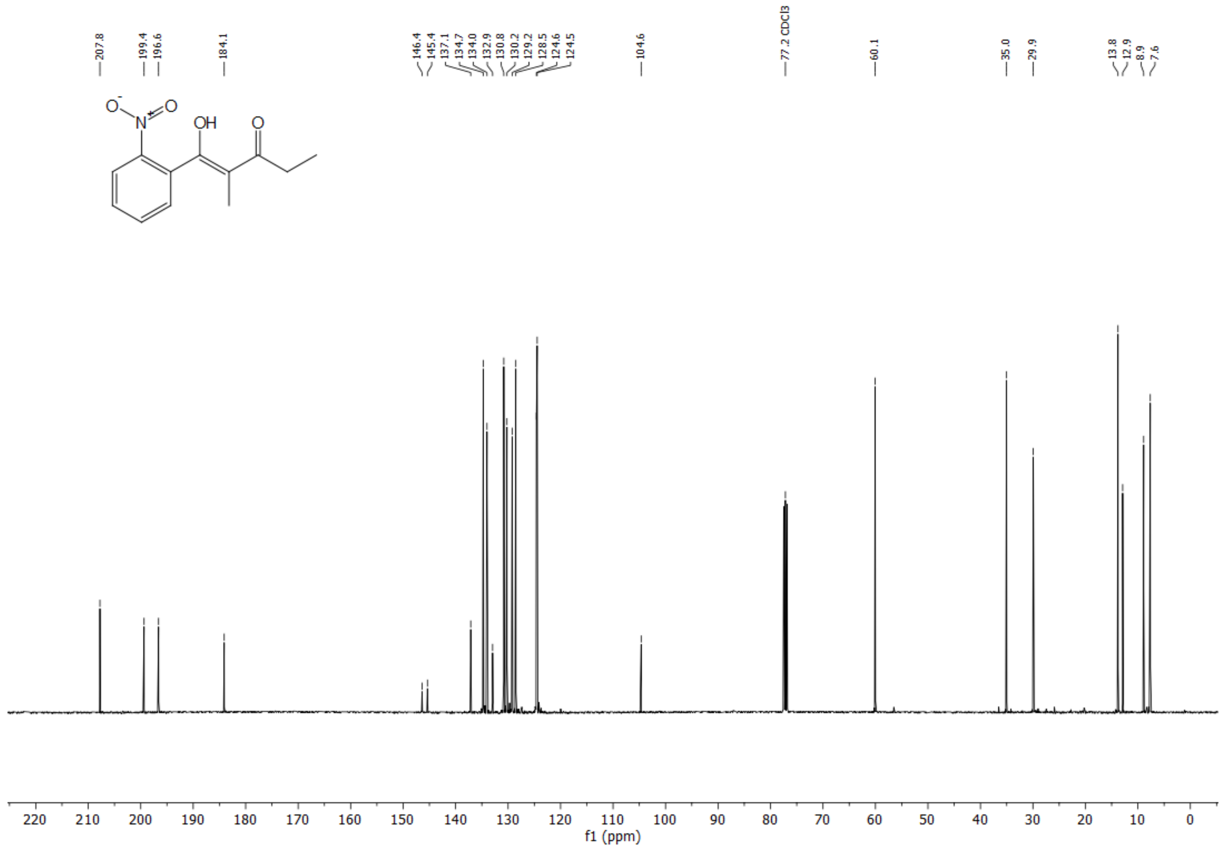

^{1}H NMR spectrum (400 MHz, $CDCl_3$) of **4x**

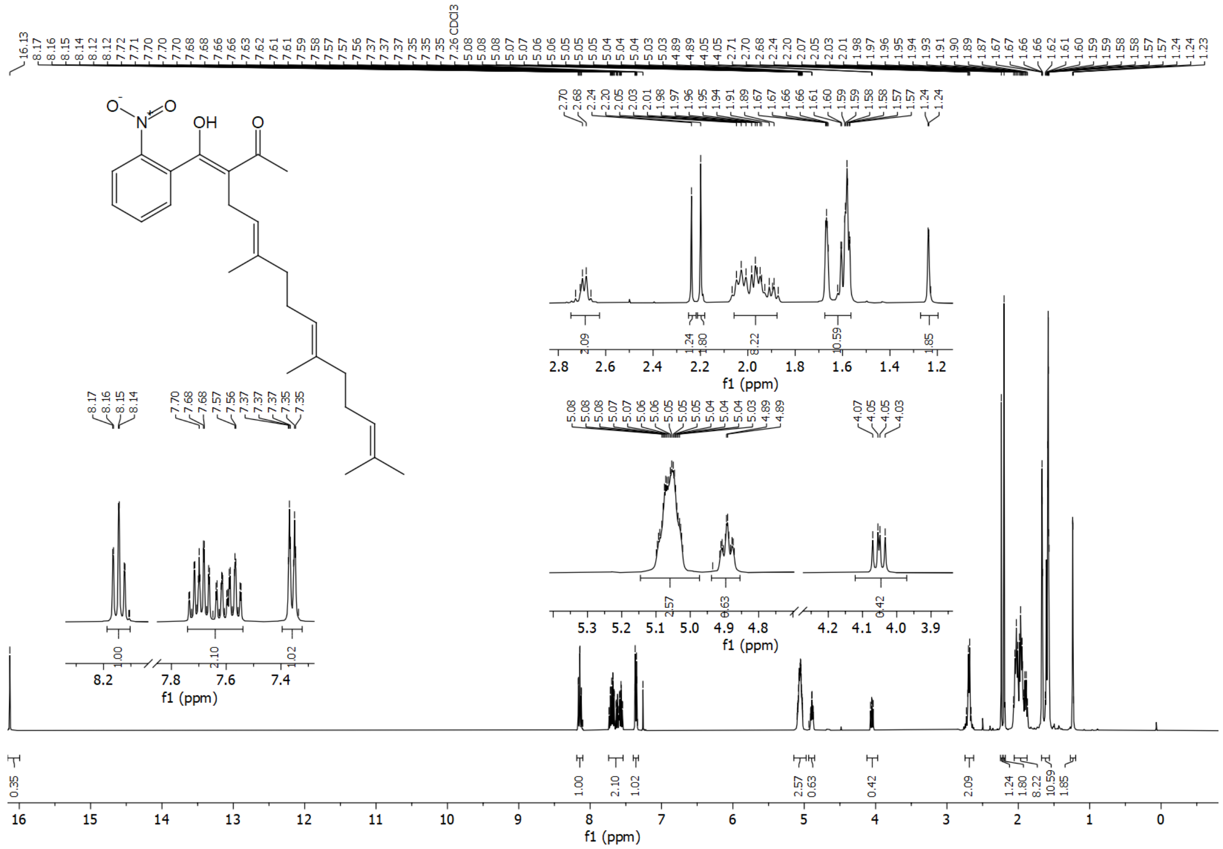

^{13}C NMR spectrum (101 MHz, $CDCl_3$) of **4x**

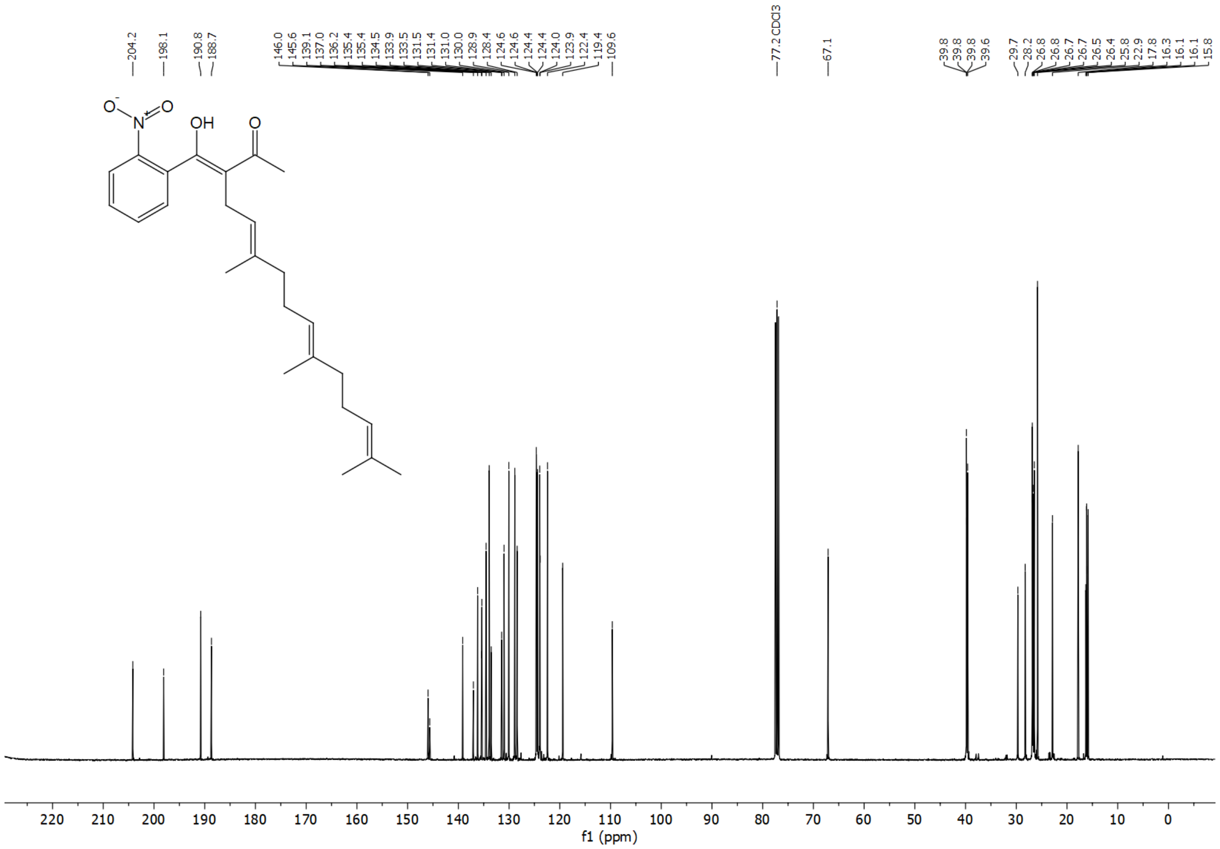

^{1}H NMR spectrum (400 MHz, DMSO-d_6) of **4y**

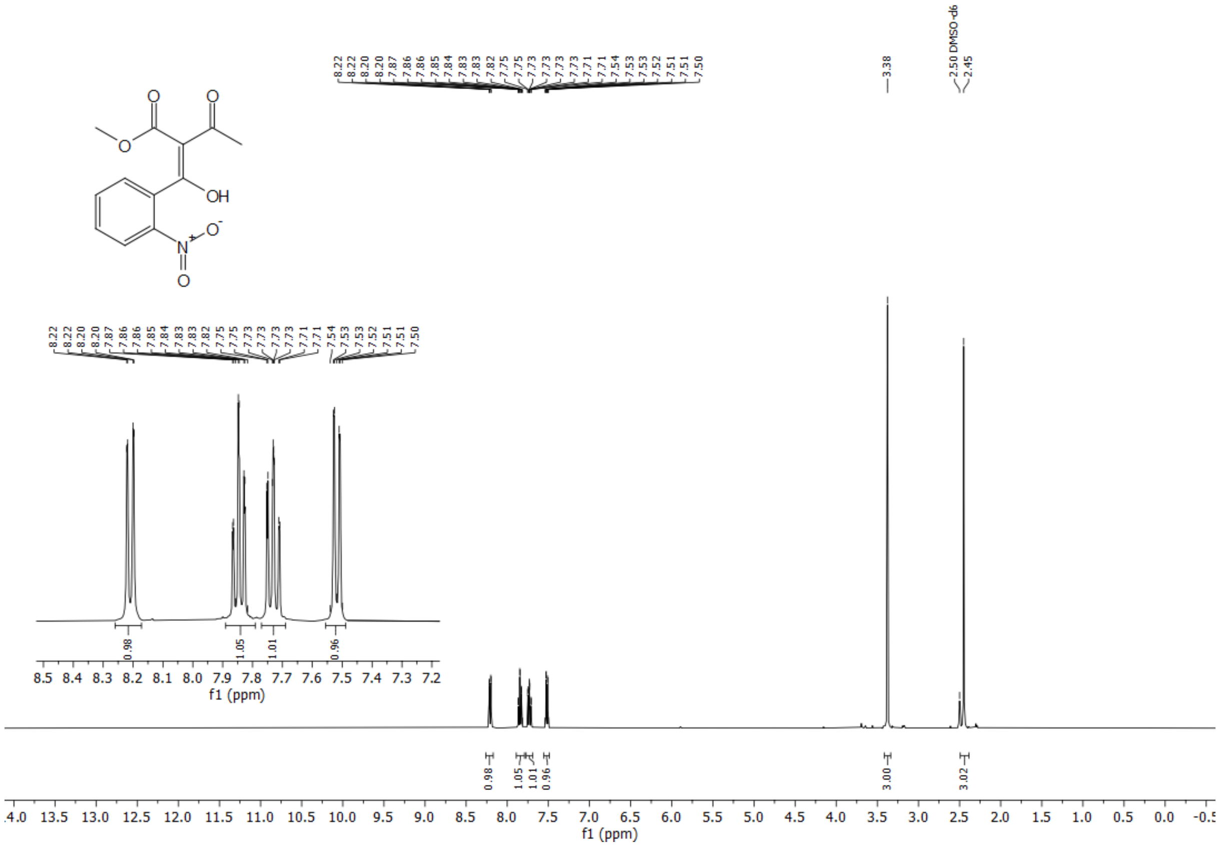

^{13}C NMR spectrum (101 MHz, DMSO-d_6) of **4y**

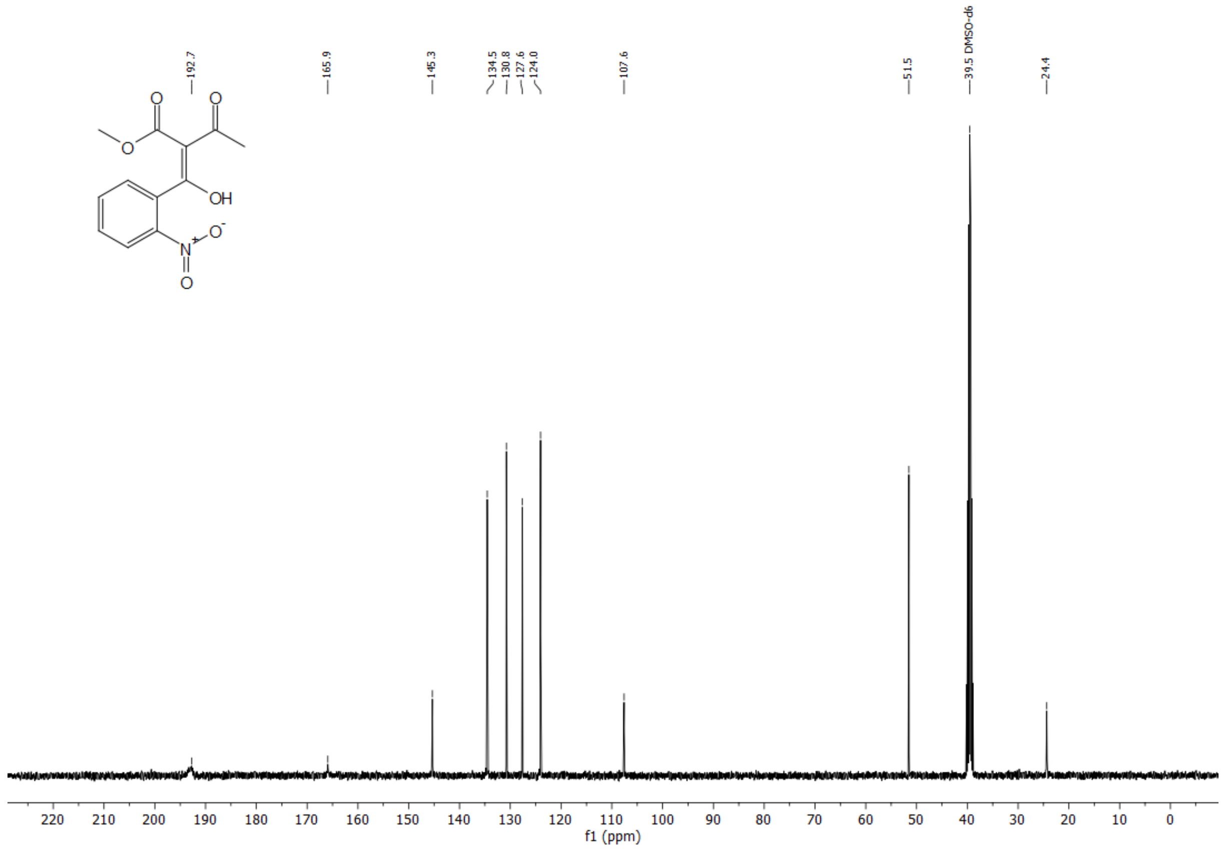

^{1}H NMR spectrum (400 MHz, $CDCl_3$) of **4z**

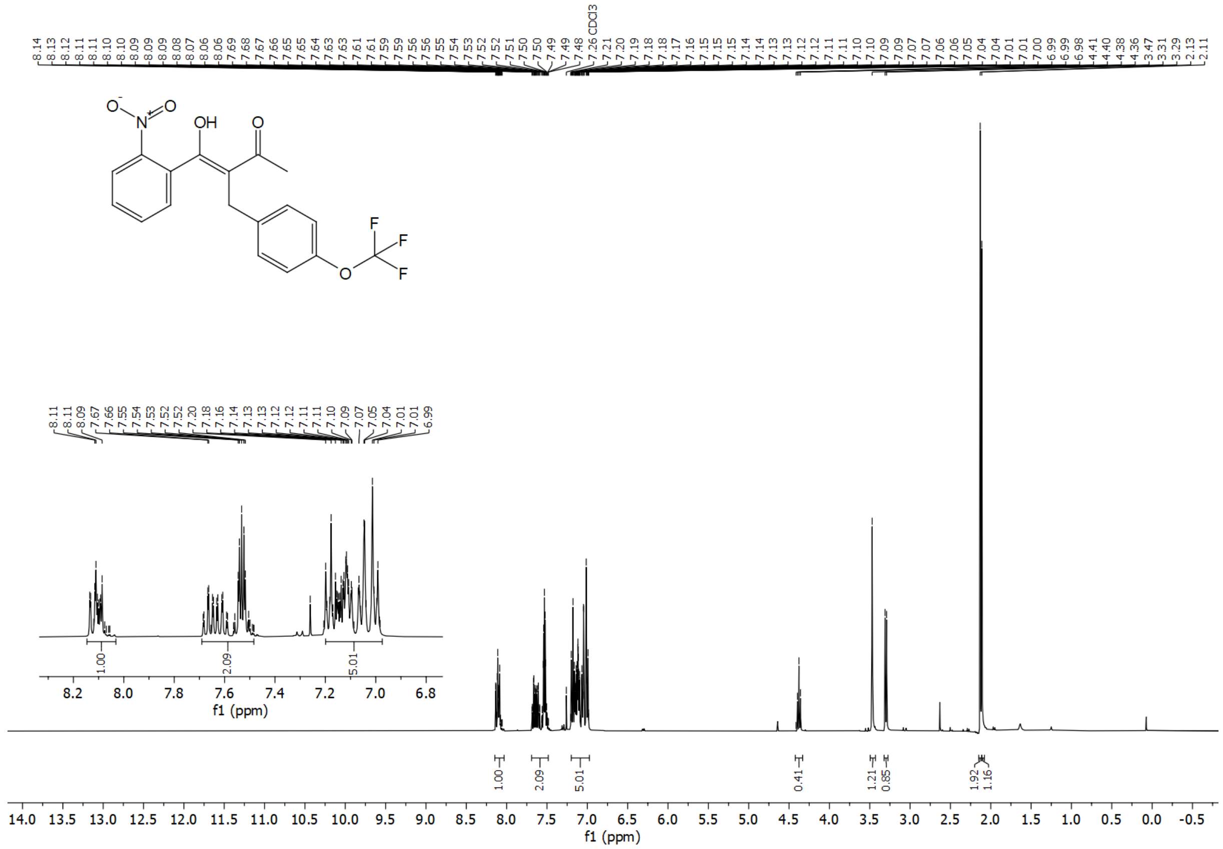

^{13}C NMR spectrum (101 MHz, $CDCl_3$) of **4z**

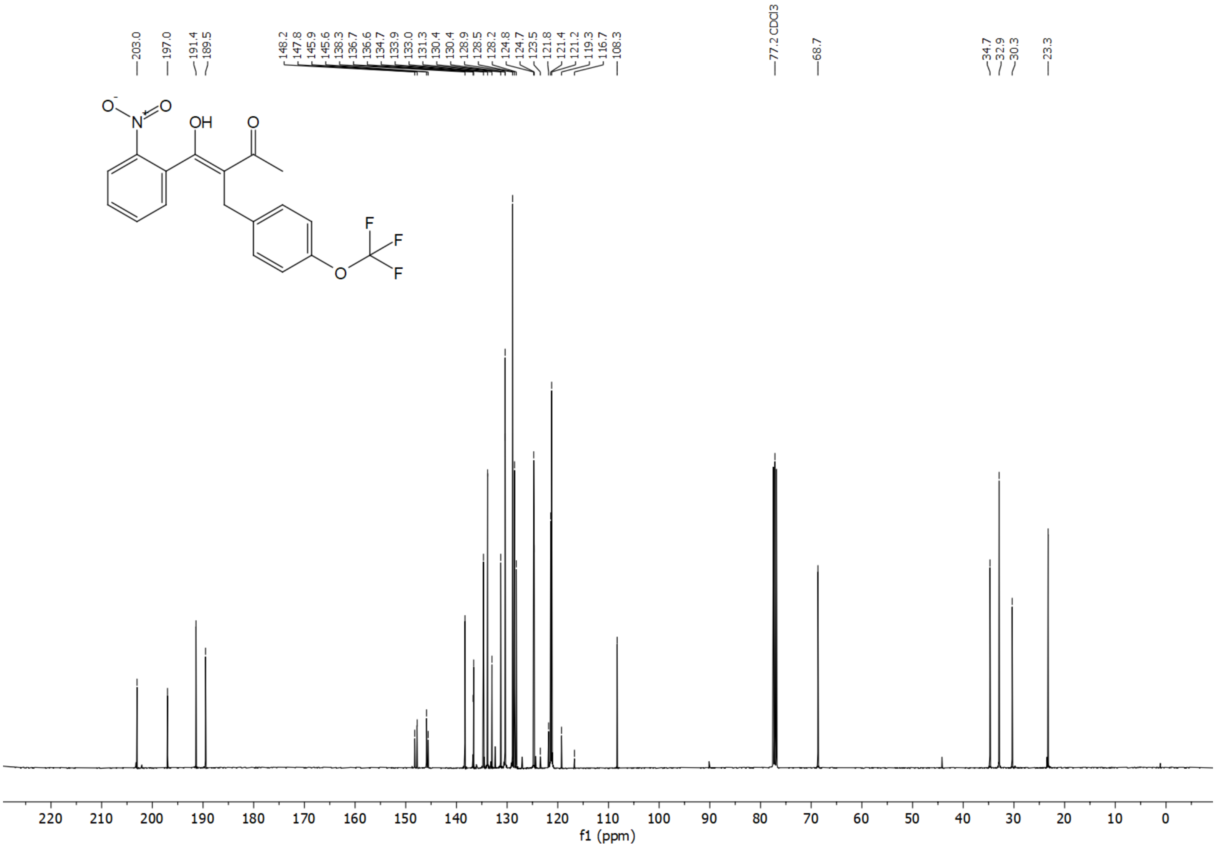

^{19}F NMR spectrum (376 MHz, $CDCl_3$) of **4z**

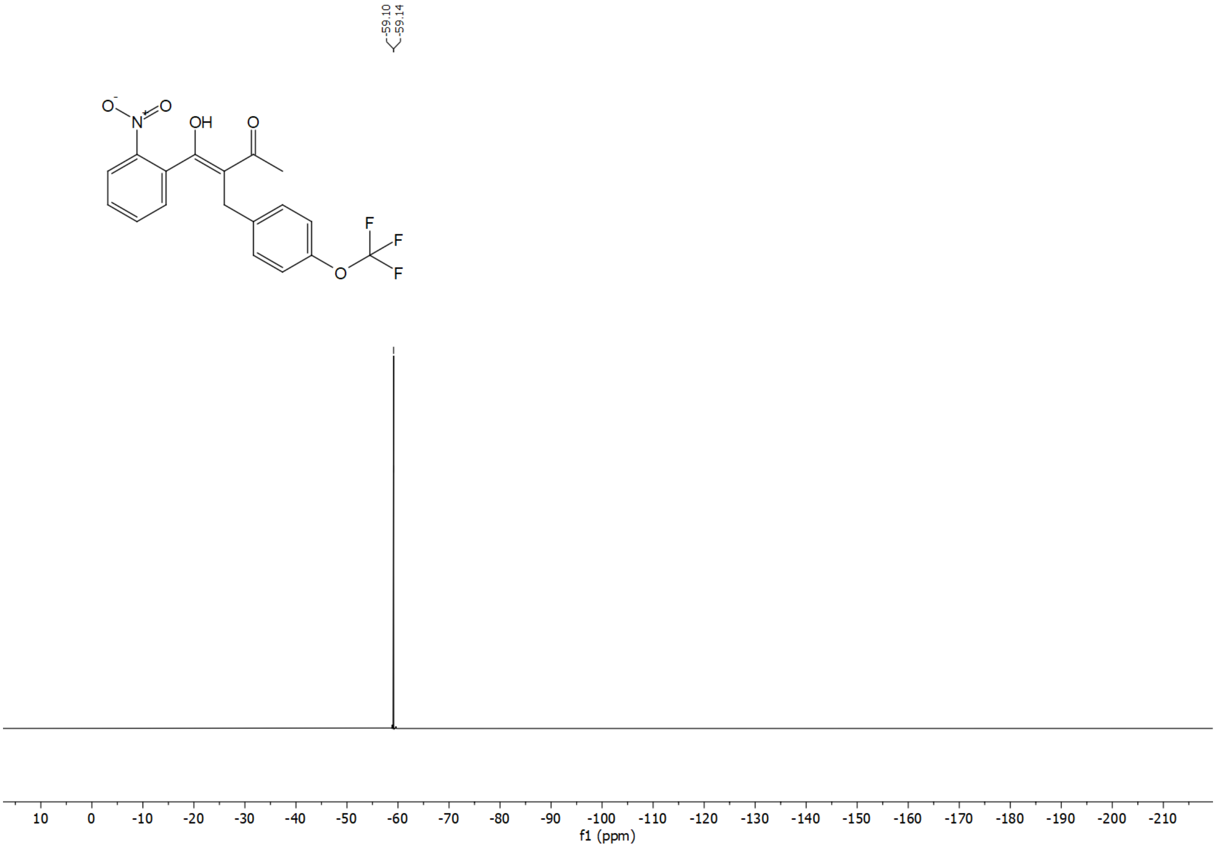

^{1}H NMR spectrum (400 MHz, $CDCl_3$) of **4aa**

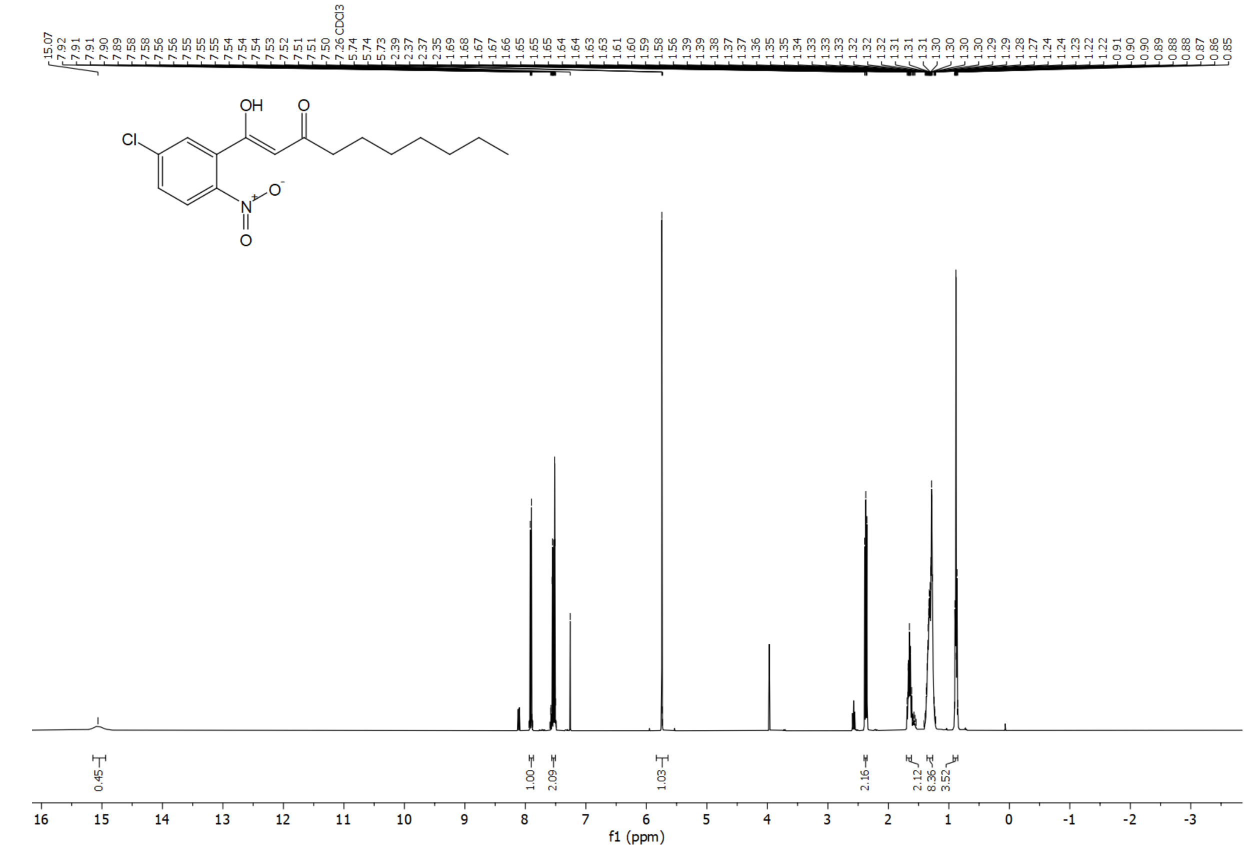

^{13}C NMR spectrum (101 MHz, $CDCl_3$) of **4aa**

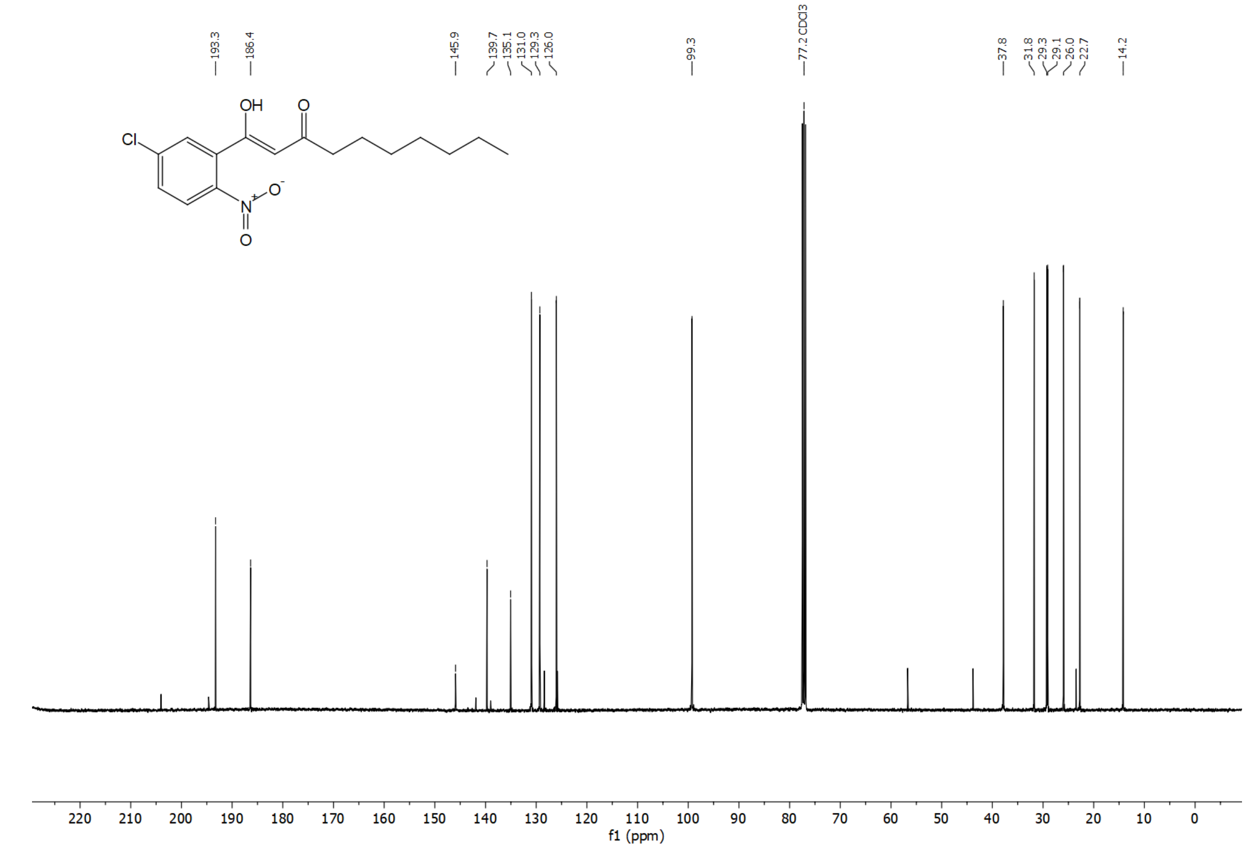

^{1}H NMR spectrum (400 MHz, DMSO-d_6) of **5a**

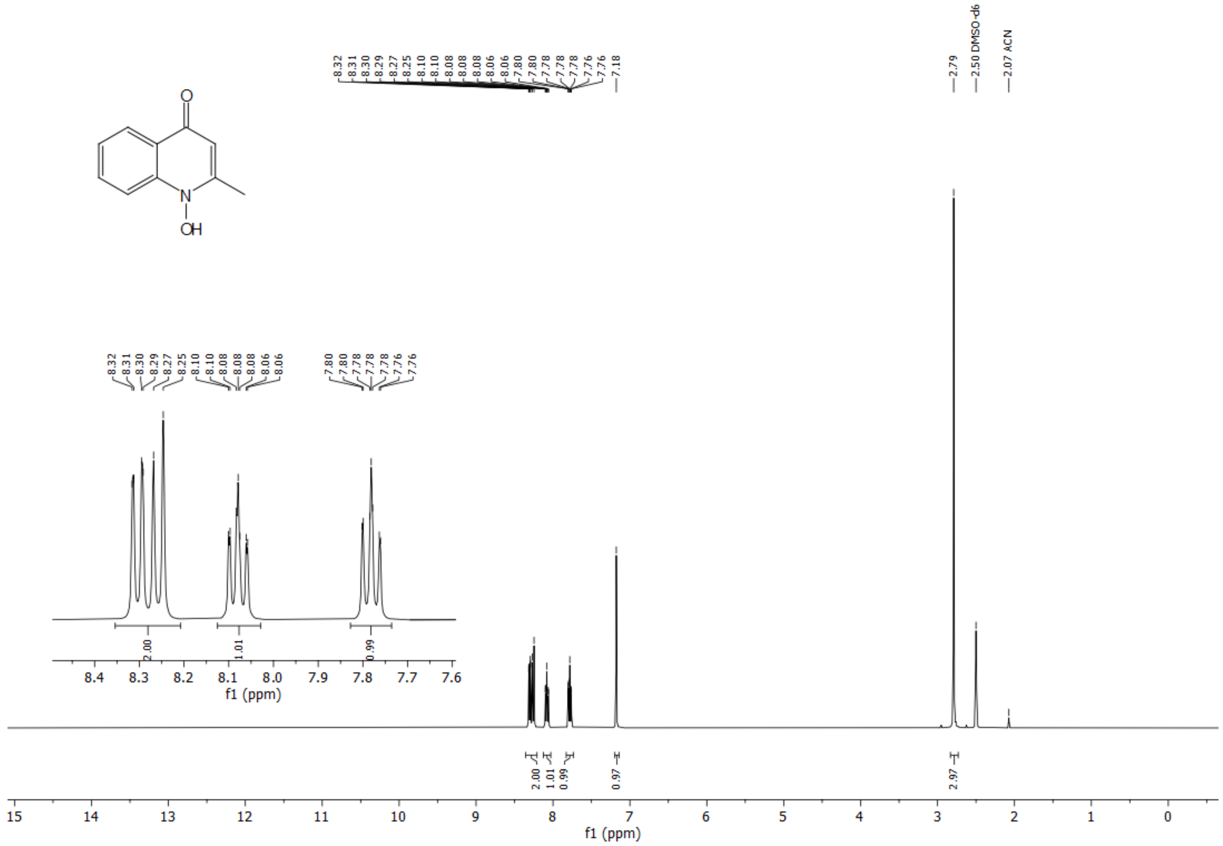

^{13}C NMR spectrum (101 MHz, DMSO-d_6) of **5a**

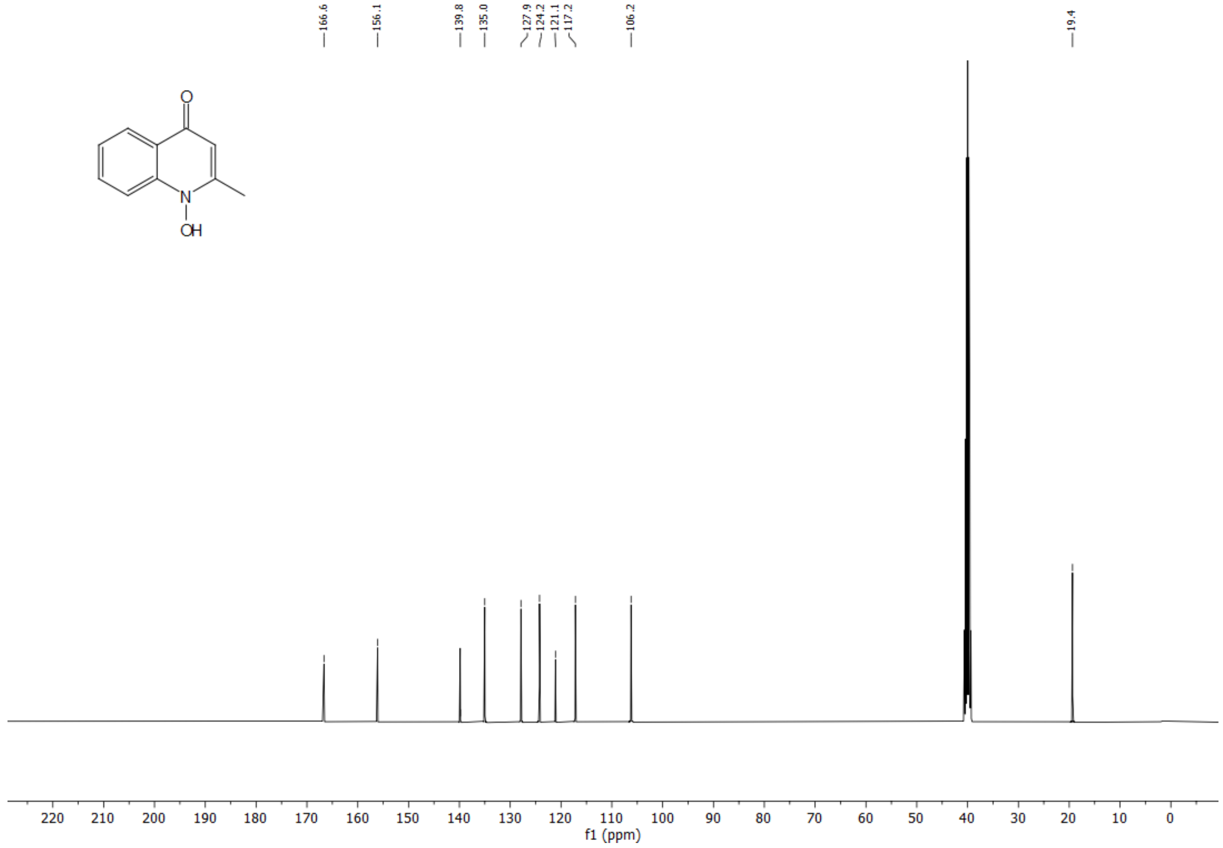

^{1}H NMR spectrum (600 MHz, DMSO-d_6) of **5c**

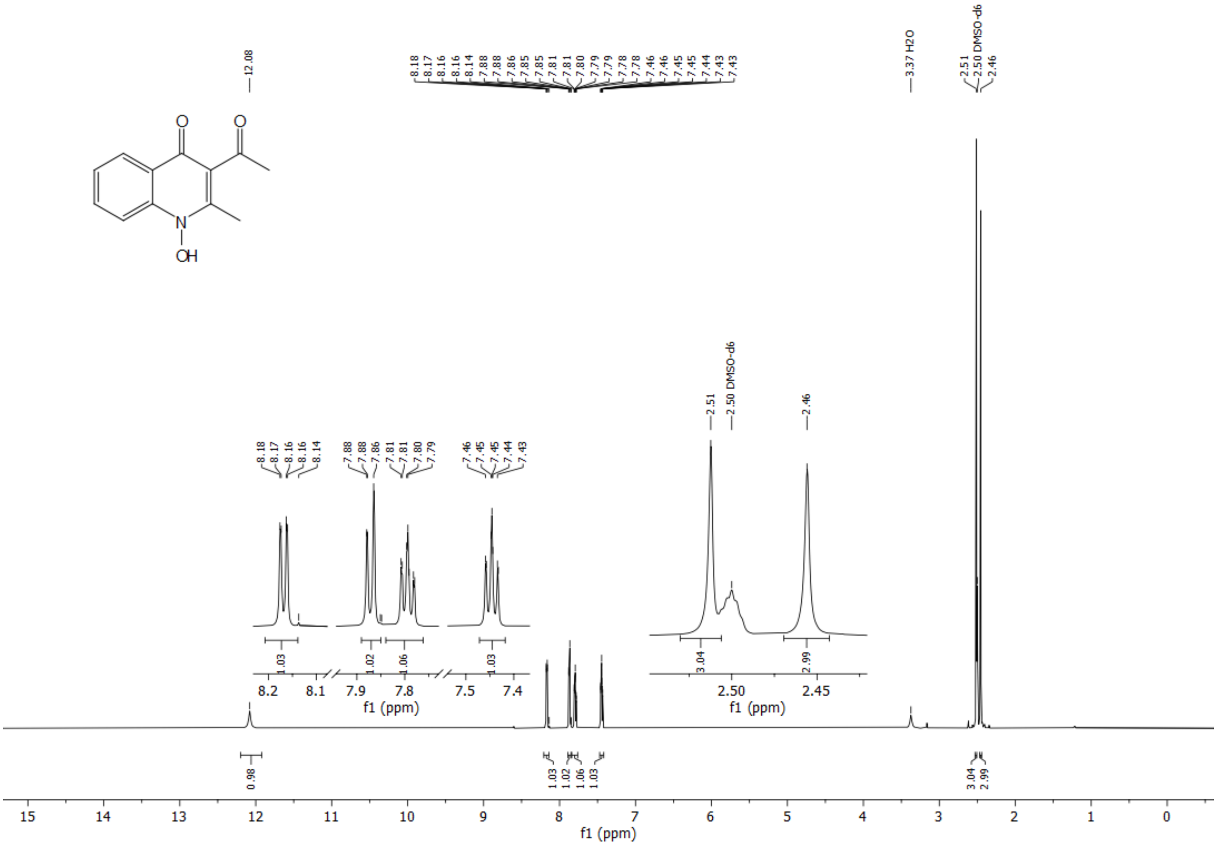

^{13}C NMR spectrum (151 MHz, DMSO-d_6) of **5c**

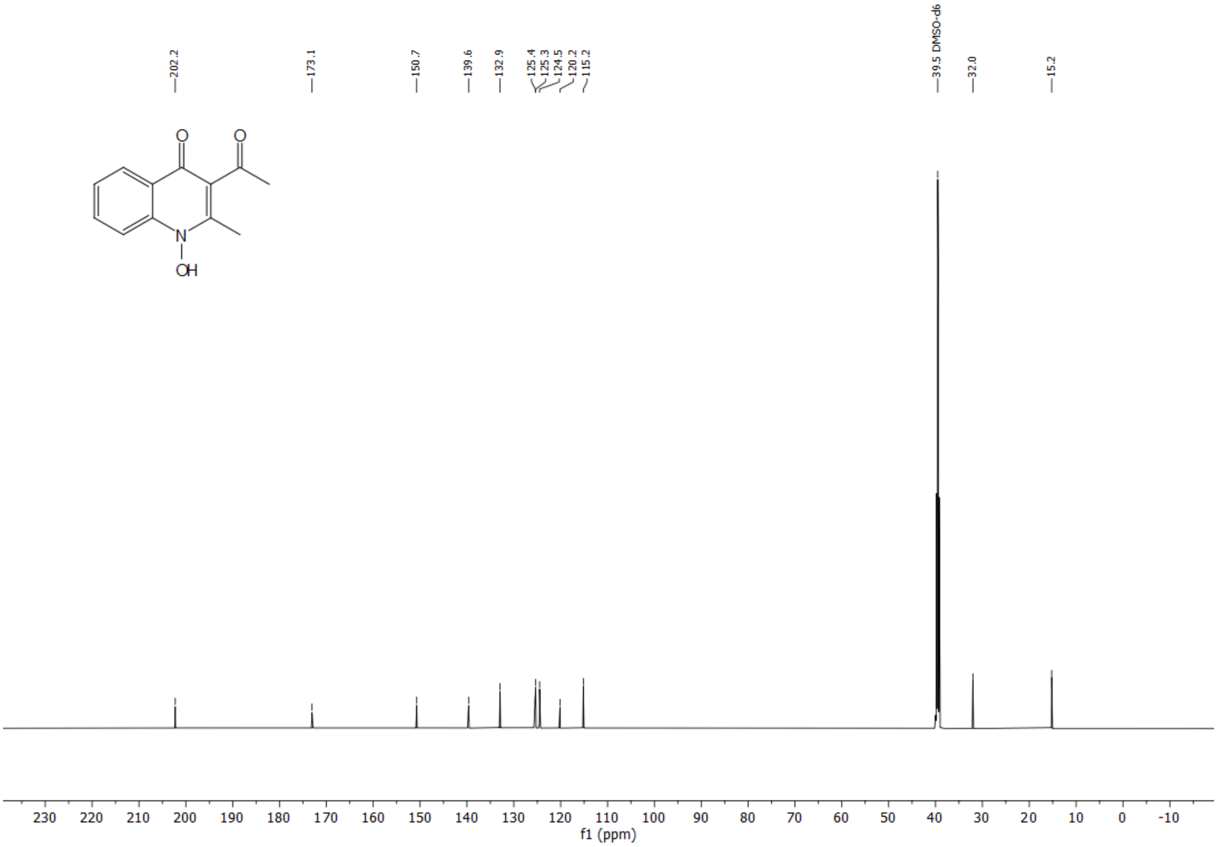

^{1}H NMR spectrum (400 MHz, D_2O+NaOD) of **5d**

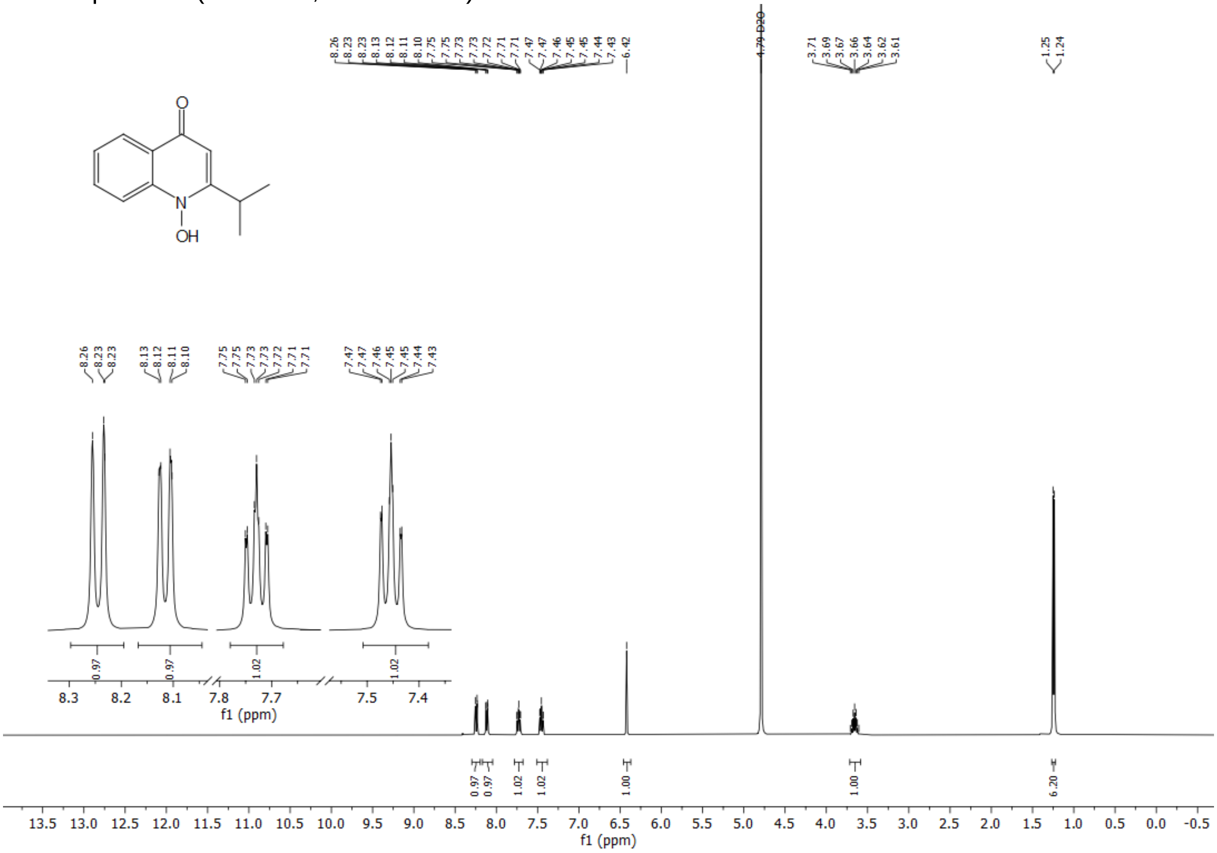

^{13}C NMR spectrum (101 MHz, D_2O+NaOD) of **5d**

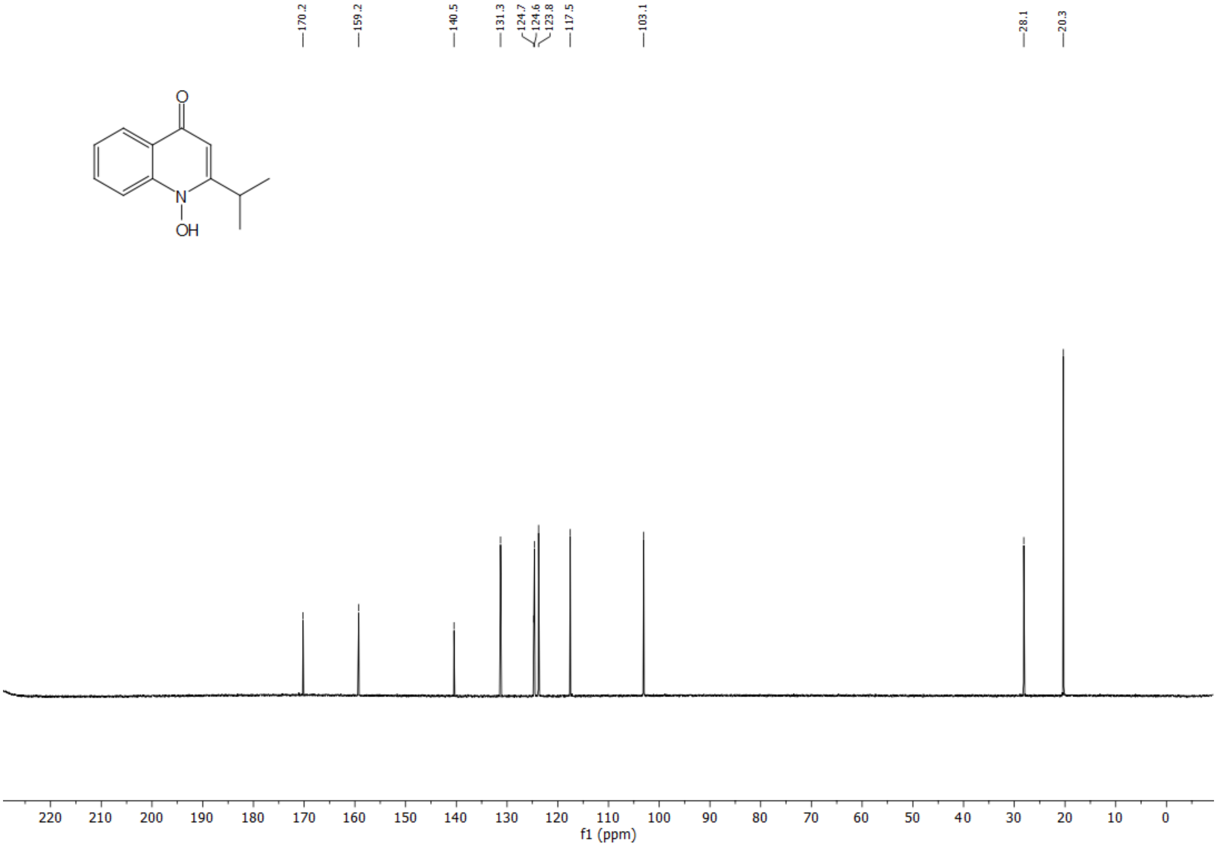

^{1}H NMR spectrum (400 MHz, D_2O+NaOD) of **5e**

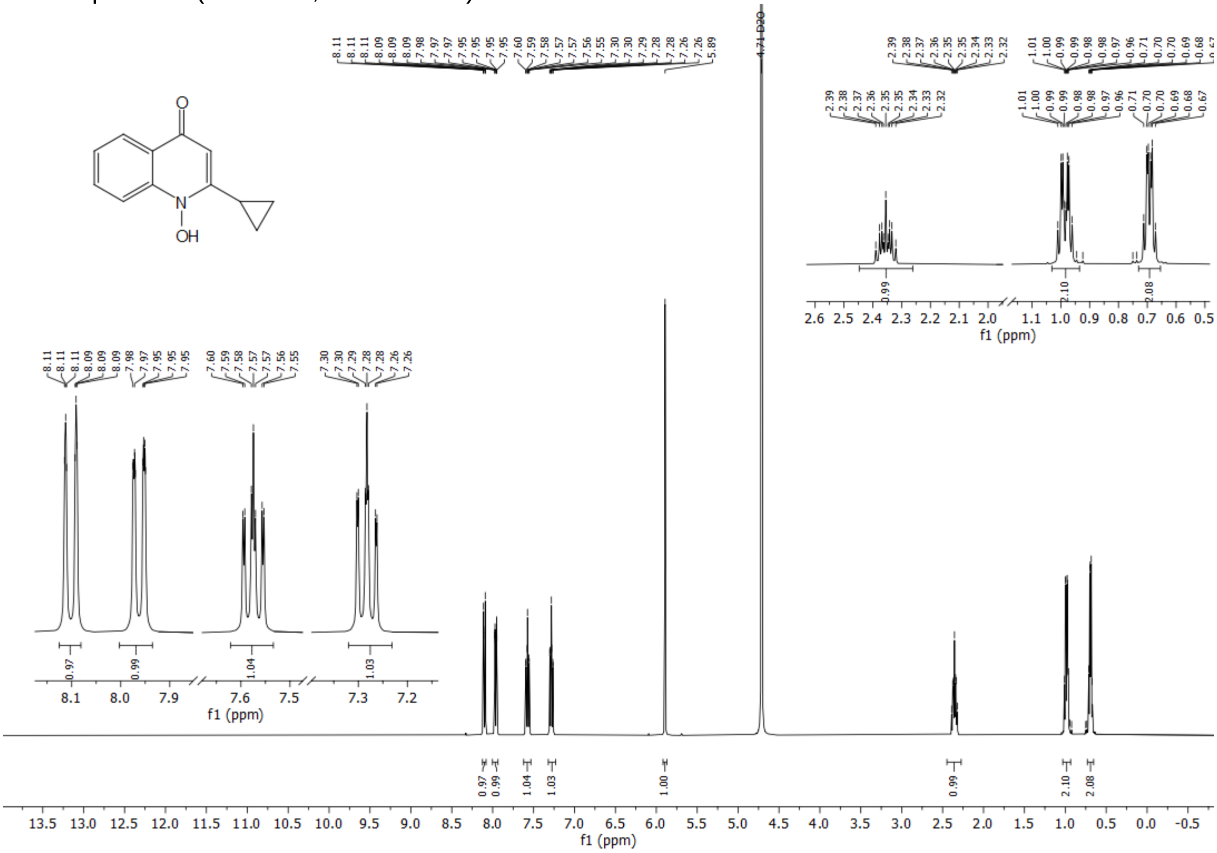

^{13}C NMR spectrum (101 MHz, D_2O+NaOD) of **5e**

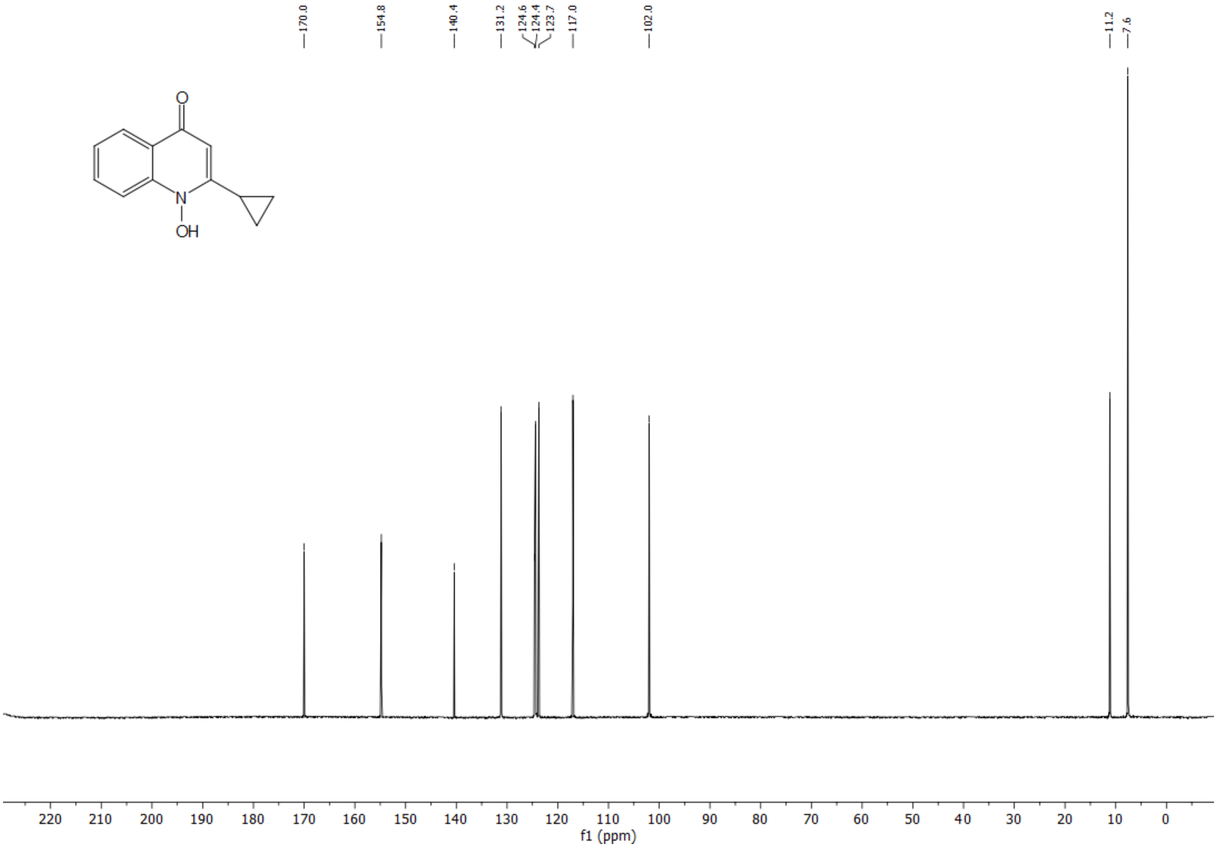

^{1}H NMR spectrum (400 MHz, D_2O+NaOD) of **5f**

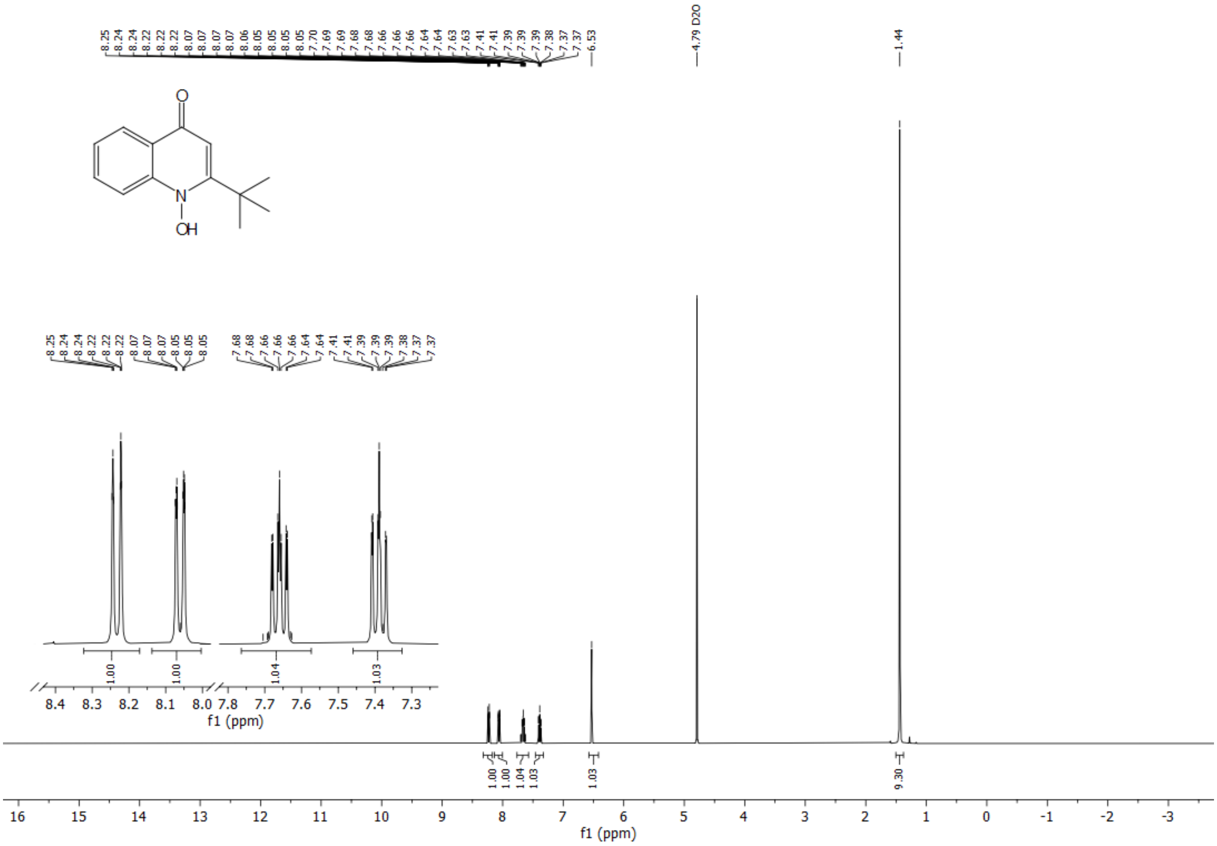

^{13}C NMR spectrum (101 MHz, D_2O+NaOD) of **5f**

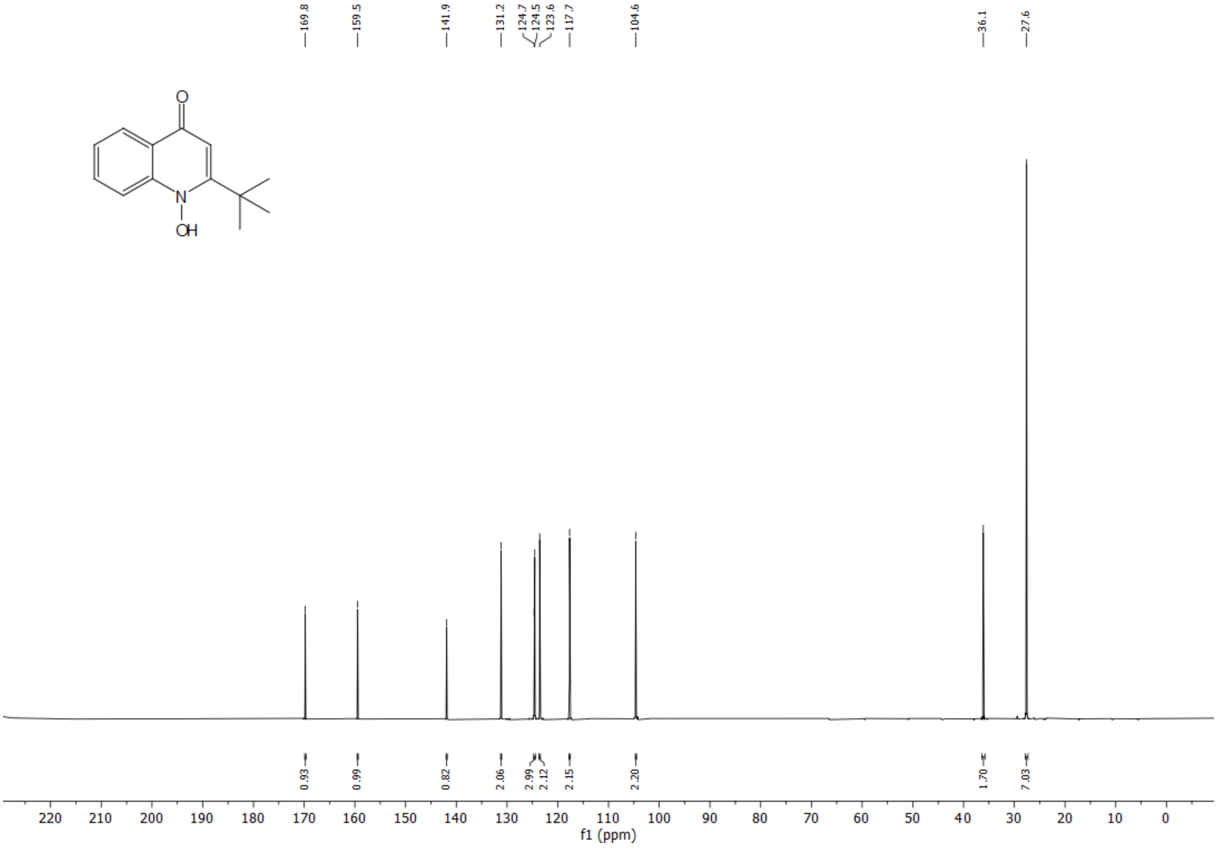

^{1}H NMR spectrum (600 MHz, DMSO-d_6) of **5g**

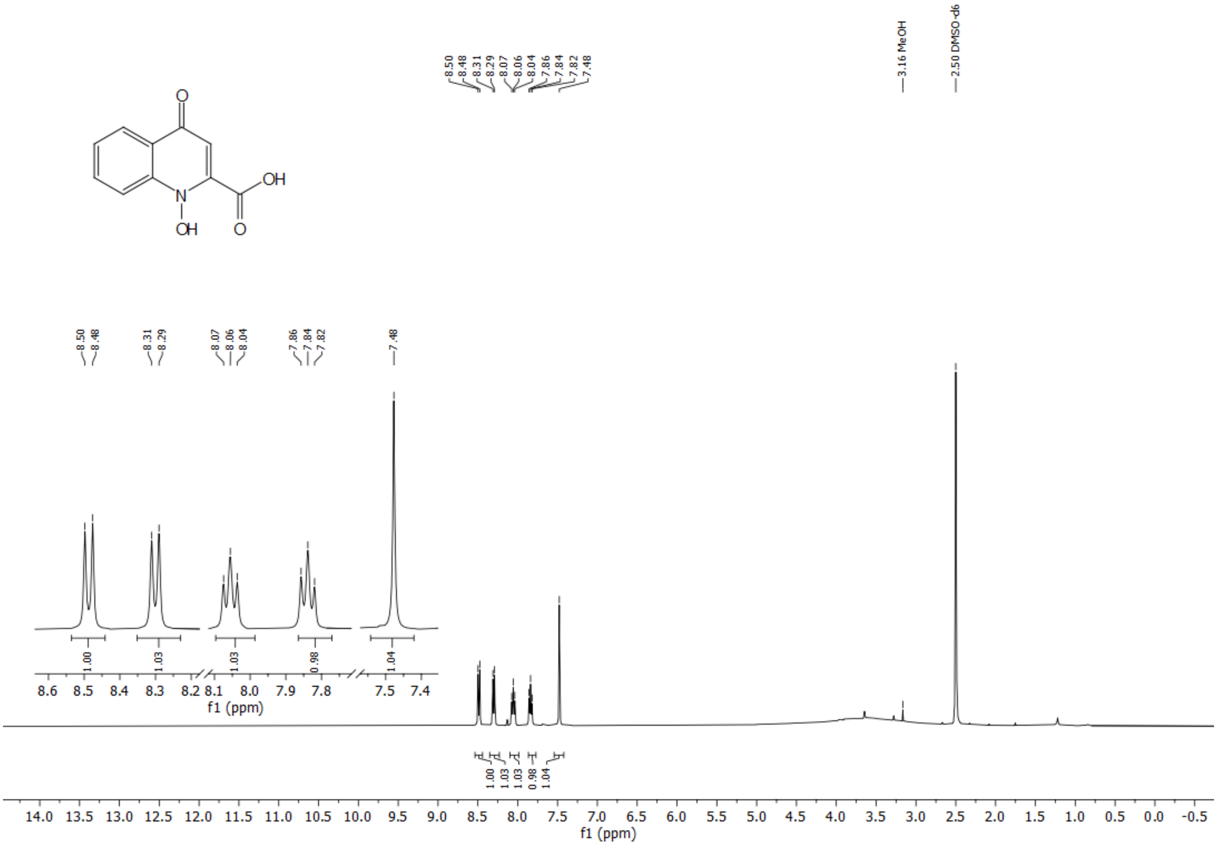

^{13}C NMR spectrum (151 MHz, DMSO-d_6) of **5g**

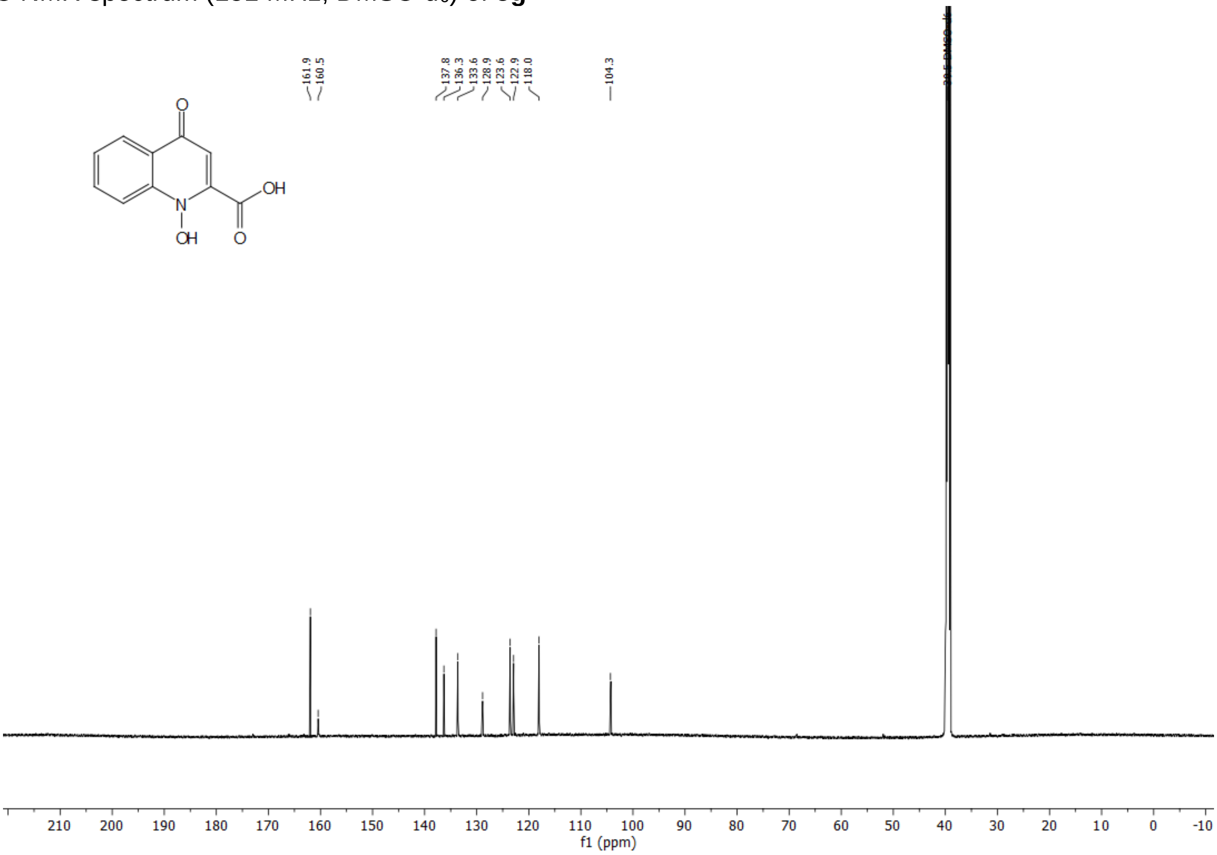

^{1}H NMR spectrum (600 MHz, DMSO-d_6) of **5h**

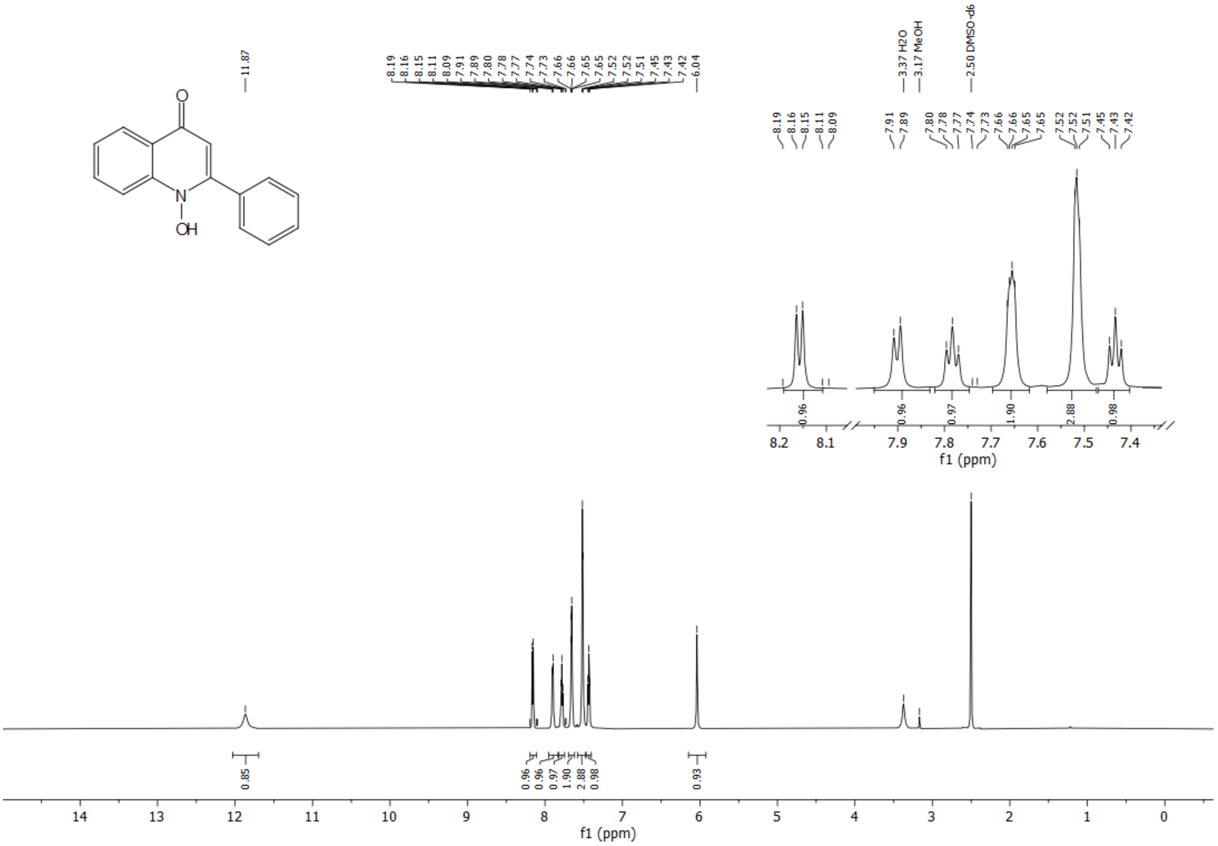

^{13}C NMR spectrum (151 MHz, DMSO-d_6) of **5h**

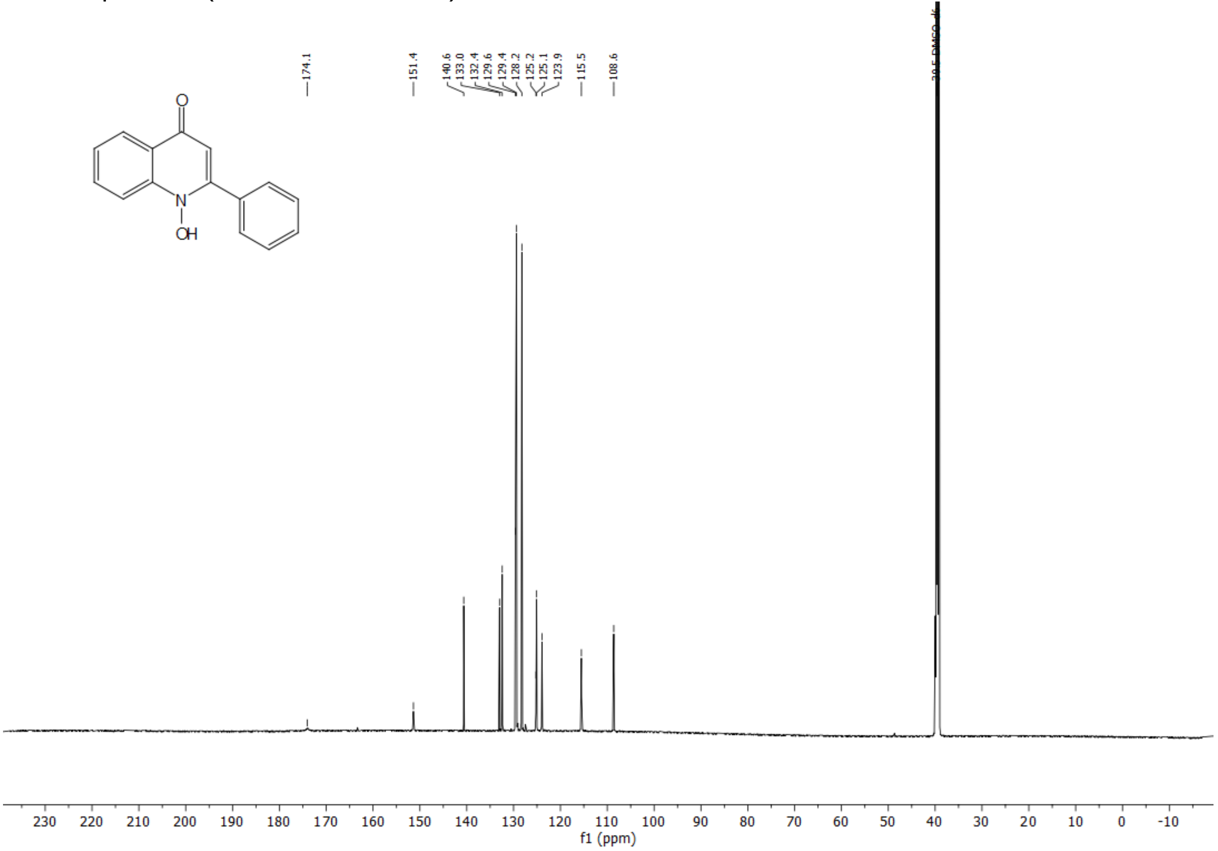

^{1}H NMR spectrum (400 MHz, D_2O+NaOD) of **5i**

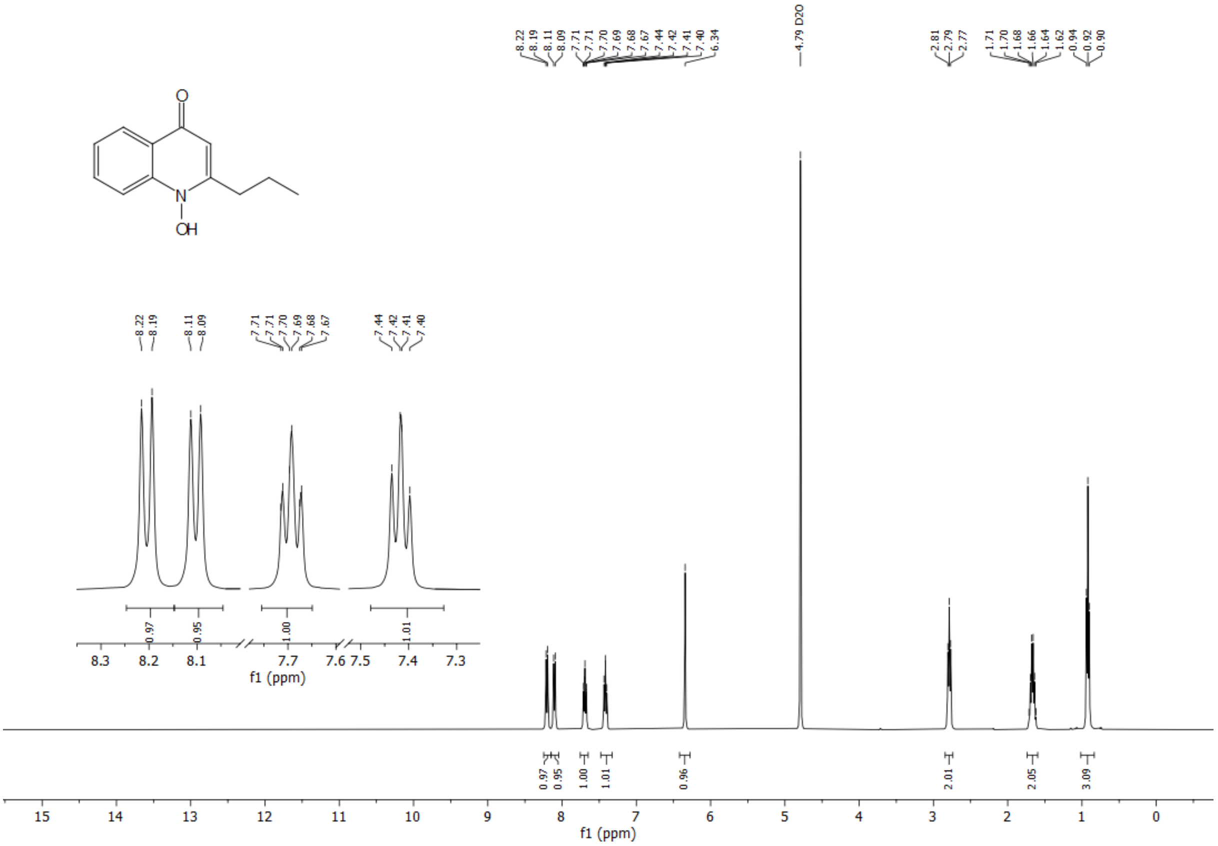

^{13}C NMR spectrum (101 MHz, D_2O+NaOD) of **5i**

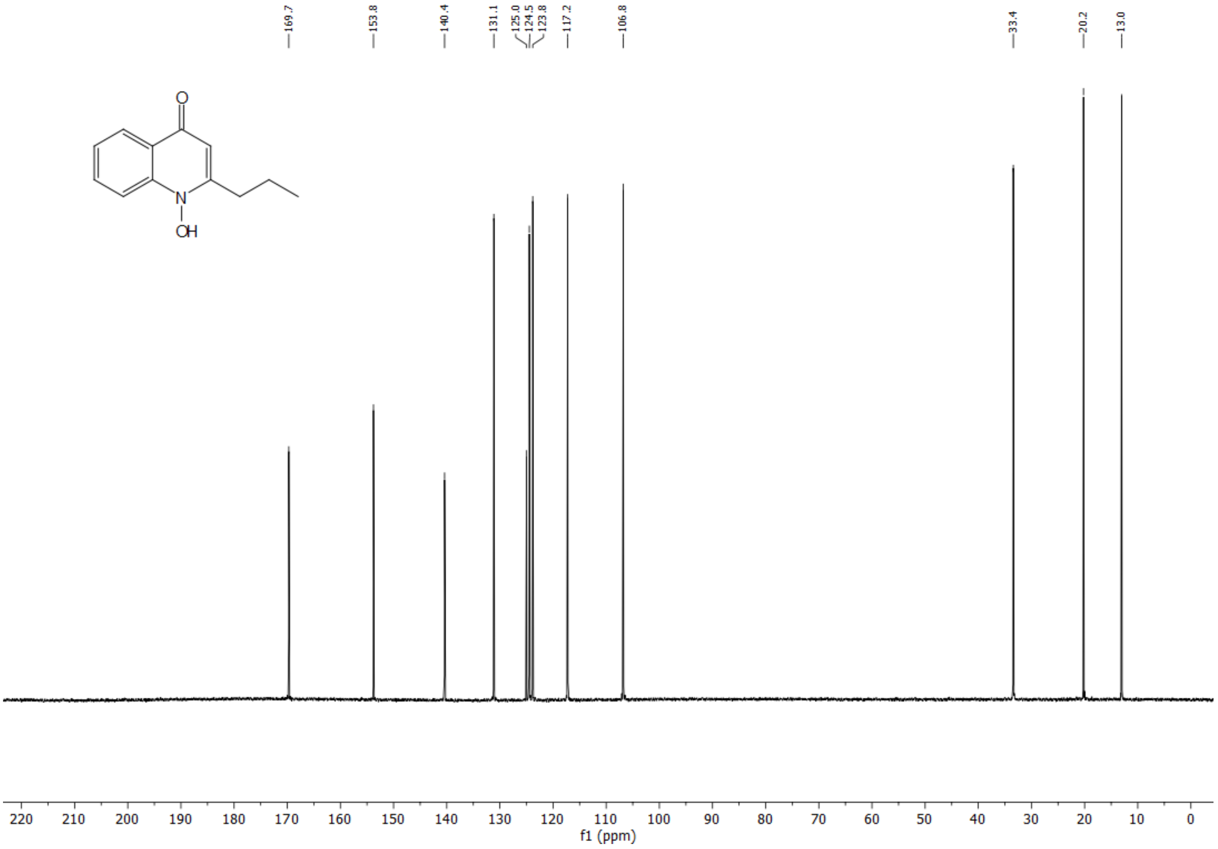

^{1}H NMR spectrum (400 MHz, DMSO-d_6) of **5k**

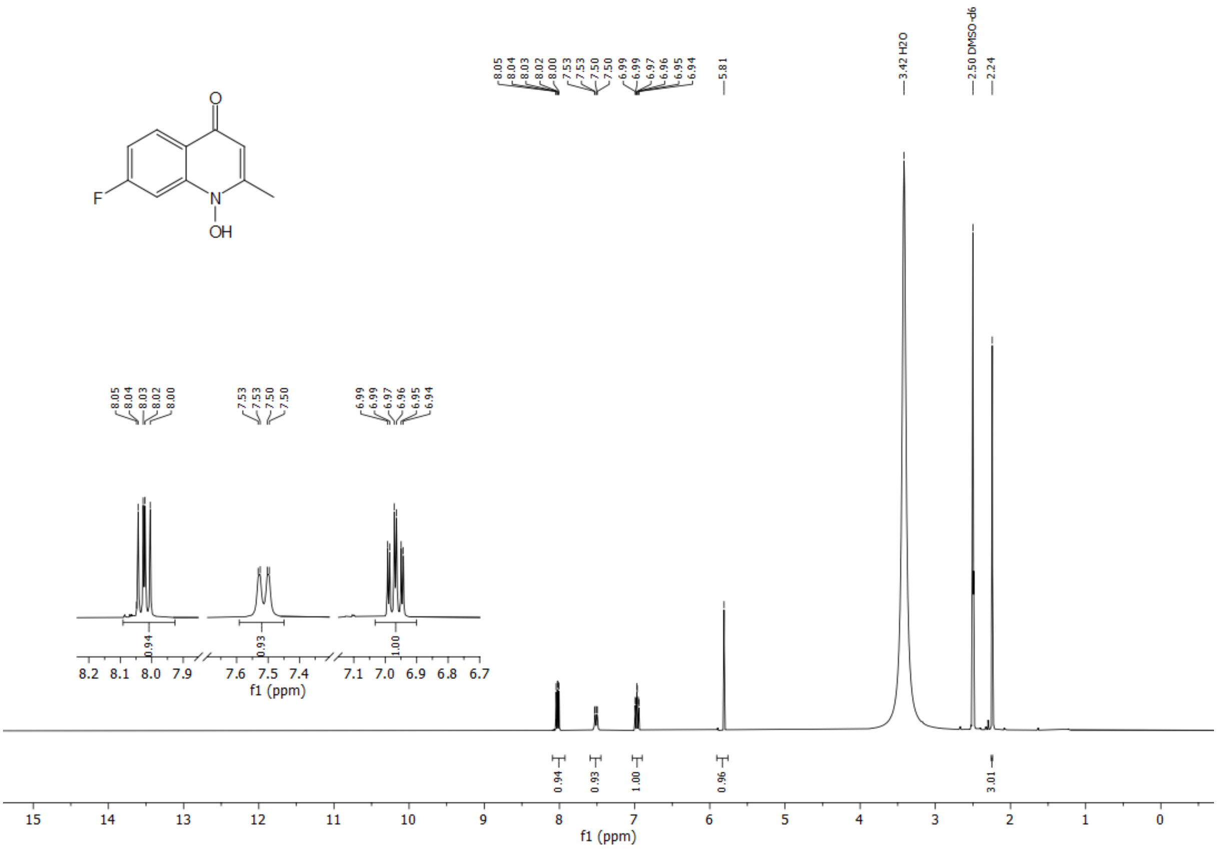

^{13}C NMR spectrum (101 MHz, DMSO-d_6) of **5k**

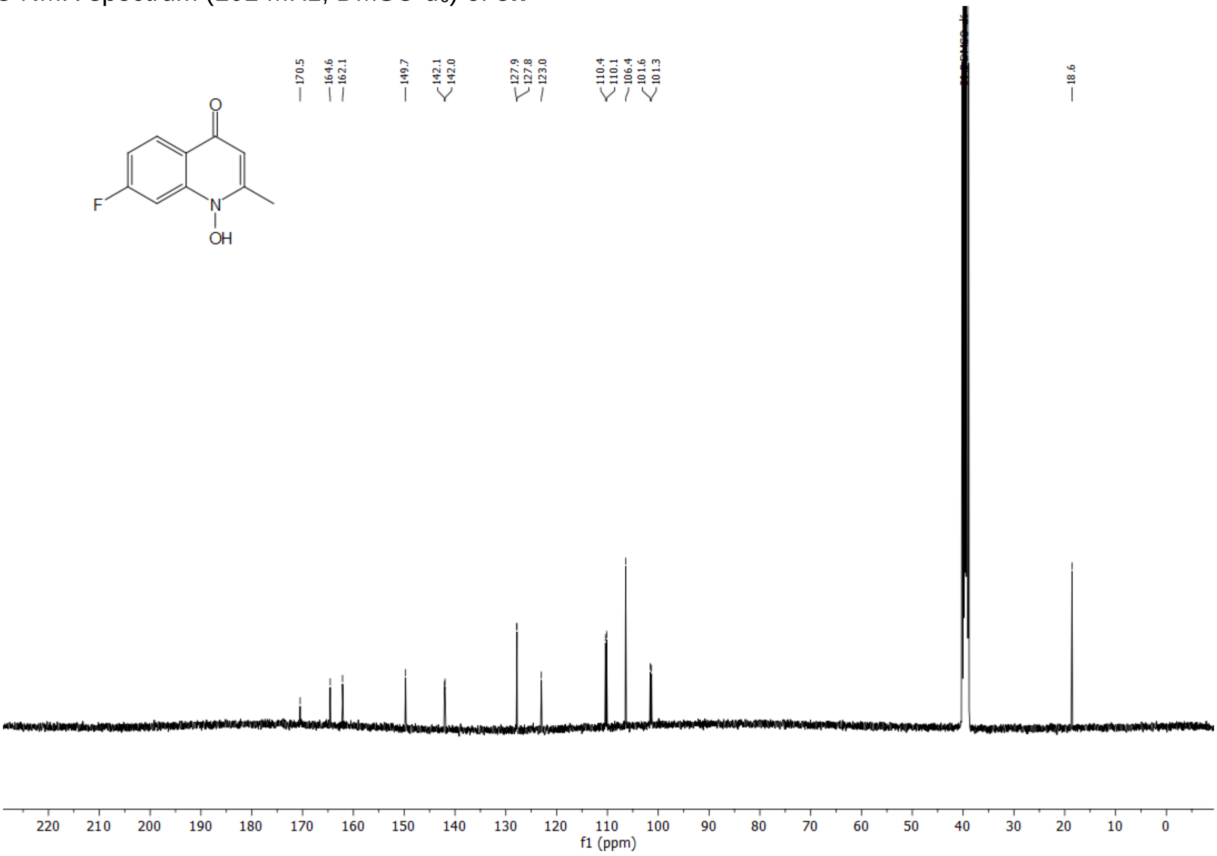

^{19}F NMR spectrum (376 MHz, DMSO-d_6) of **5k**

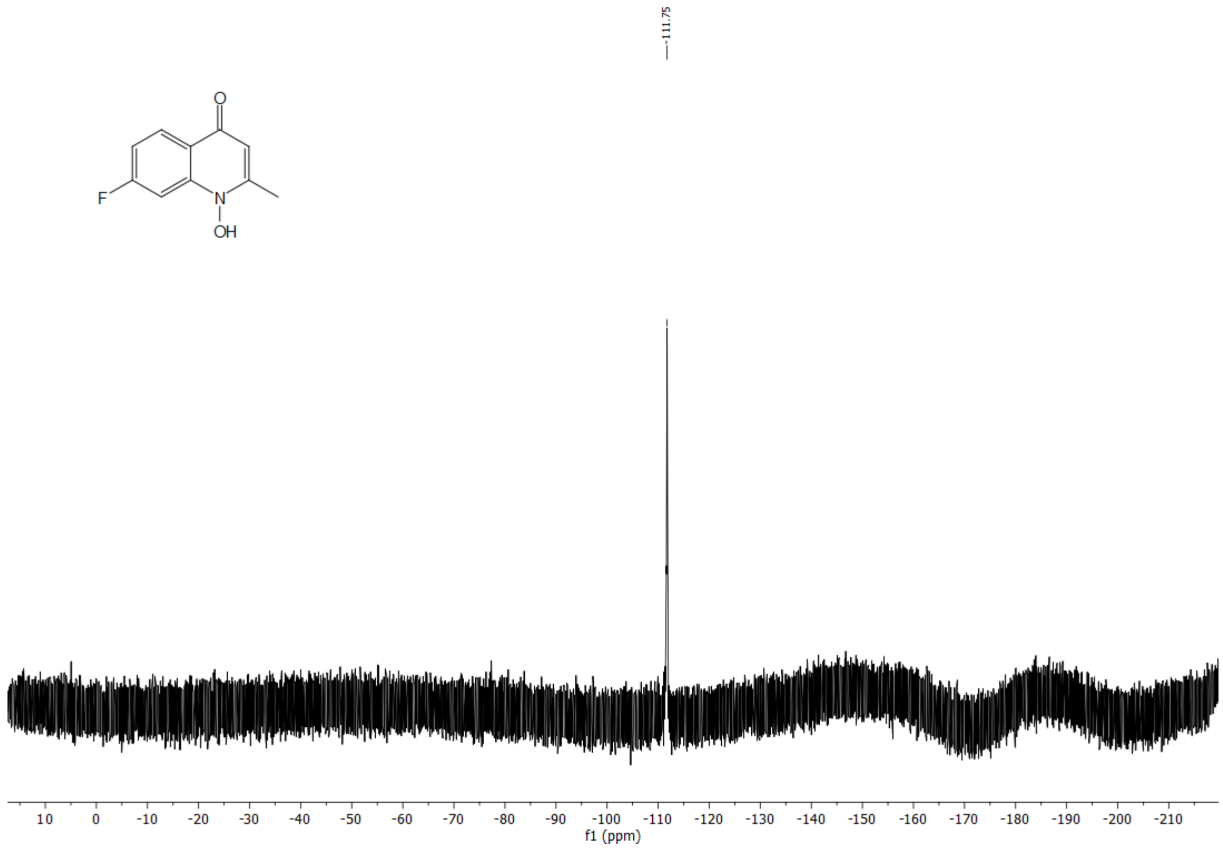

^{1}H NMR spectrum (400 MHz, DMSO-d_6) of **5l**

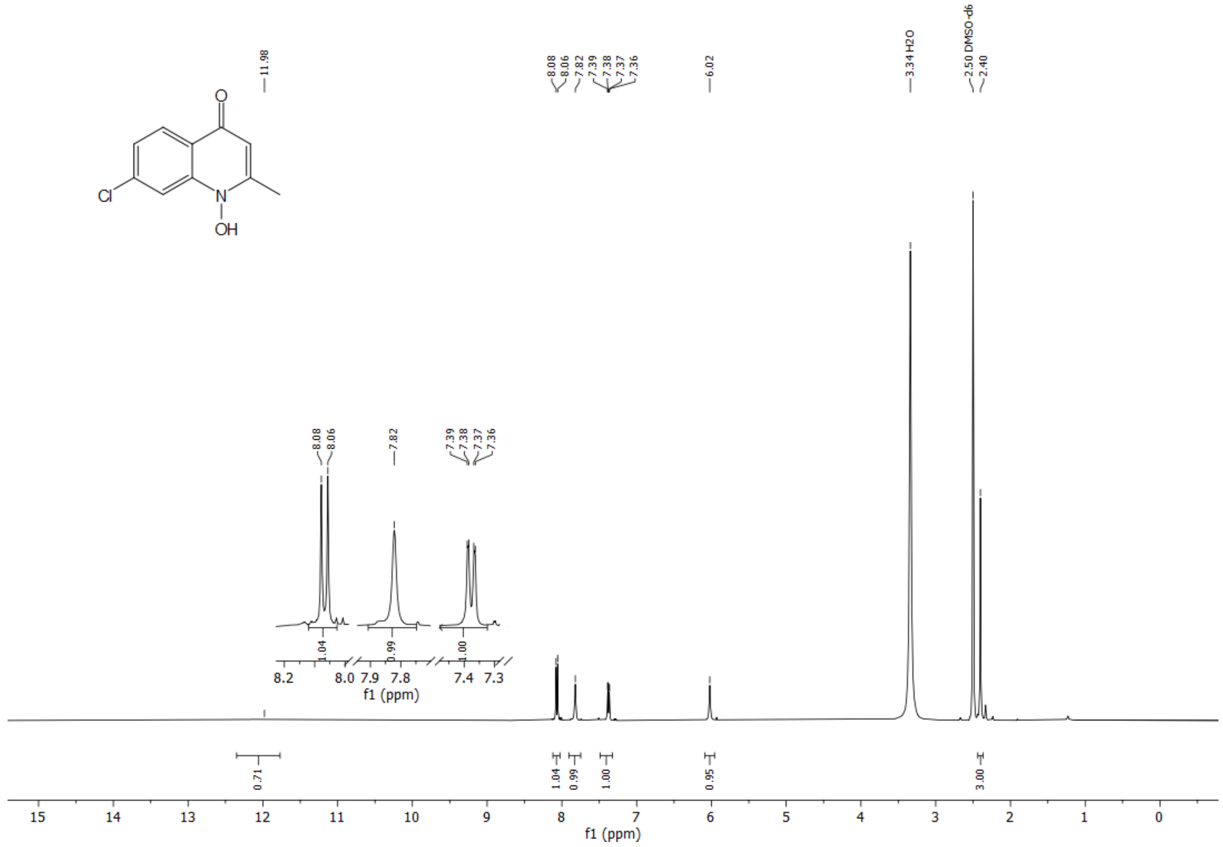

^{13}C NMR spectrum (101 MHz, DMSO-d_6) of **5l**

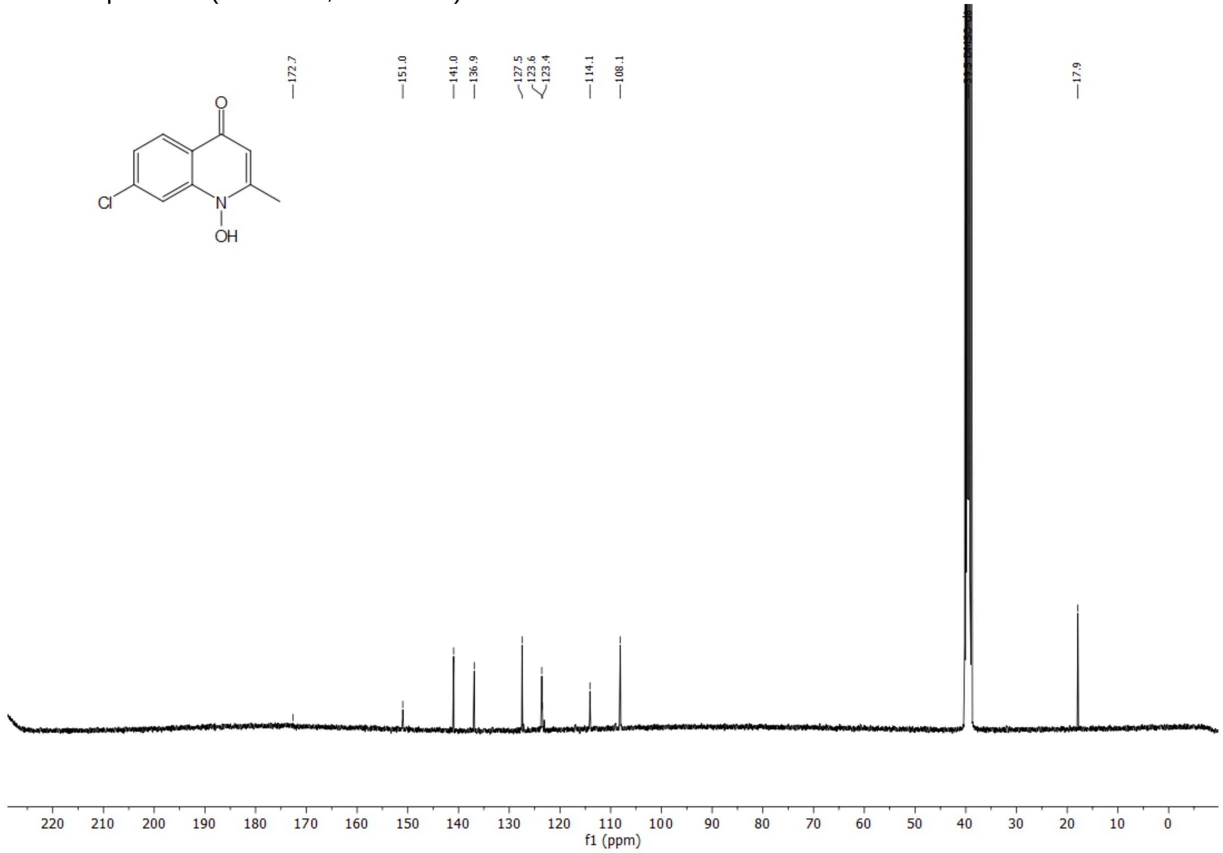

^{1}H NMR spectrum (400 MHz, D_2O+NaOD) of **5m**

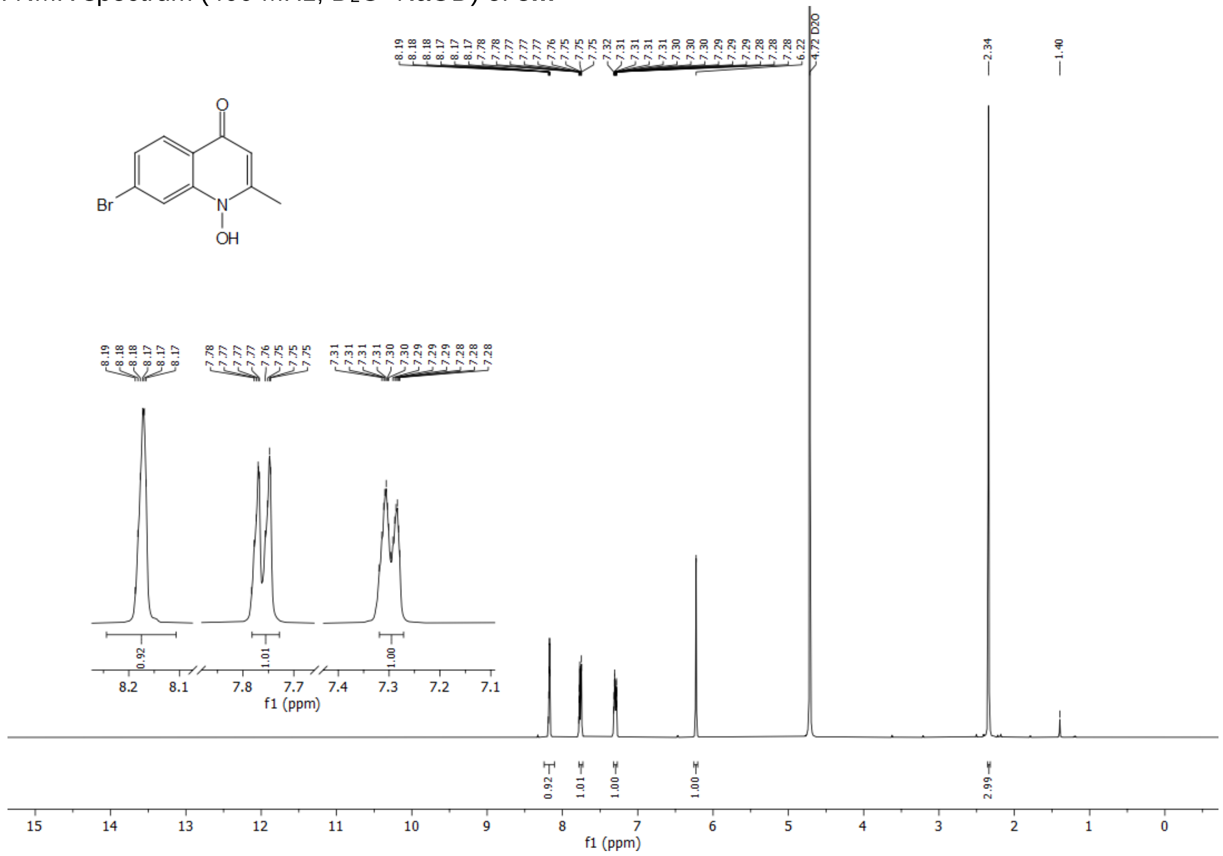

^{13}C NMR spectrum (101 MHz, D_2O+NaOD) of **5m**

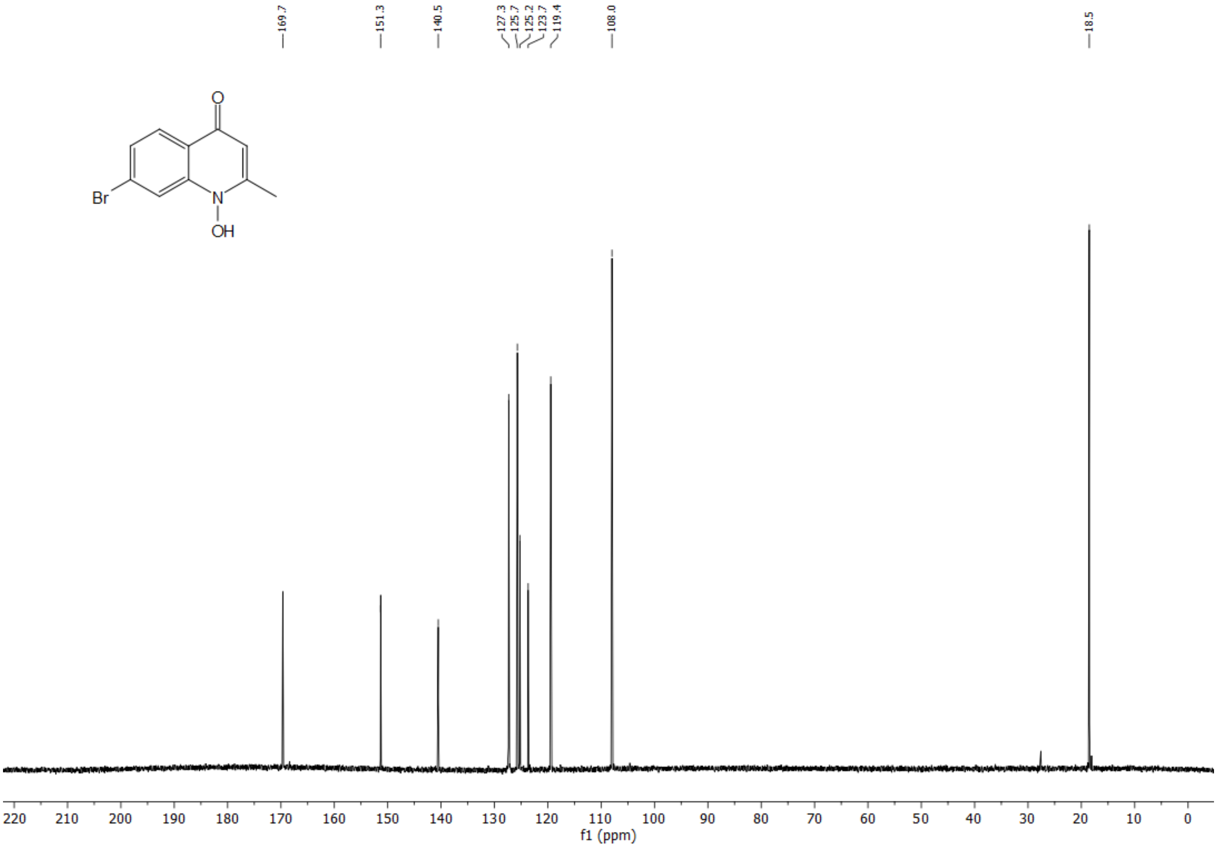

^{1}H NMR spectrum (600 MHz, DMSO-d_6) of **5n**

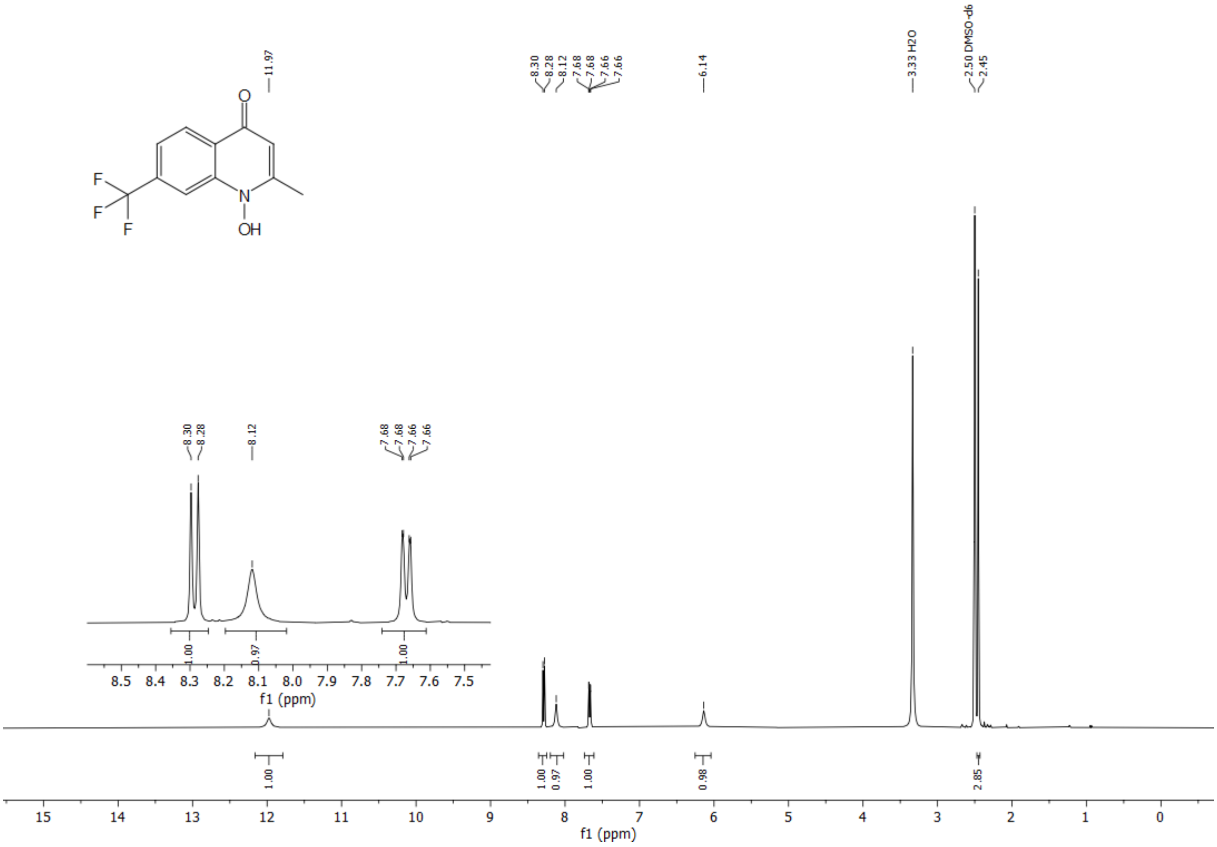

^{13}C NMR spectrum (151 MHz, DMSO-d_6) of **5n**

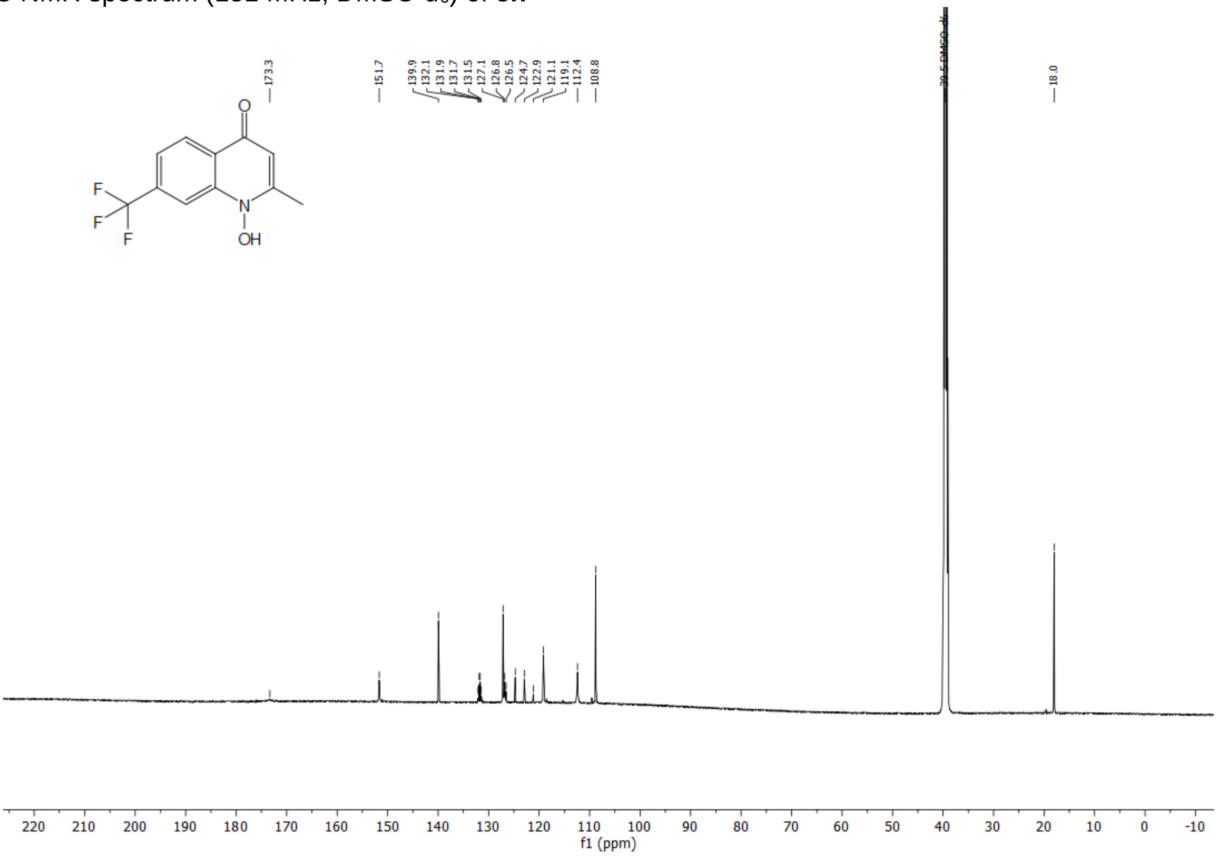

^{19}F NMR spectrum (376 MHz, DMSO-d_6) of **5n**

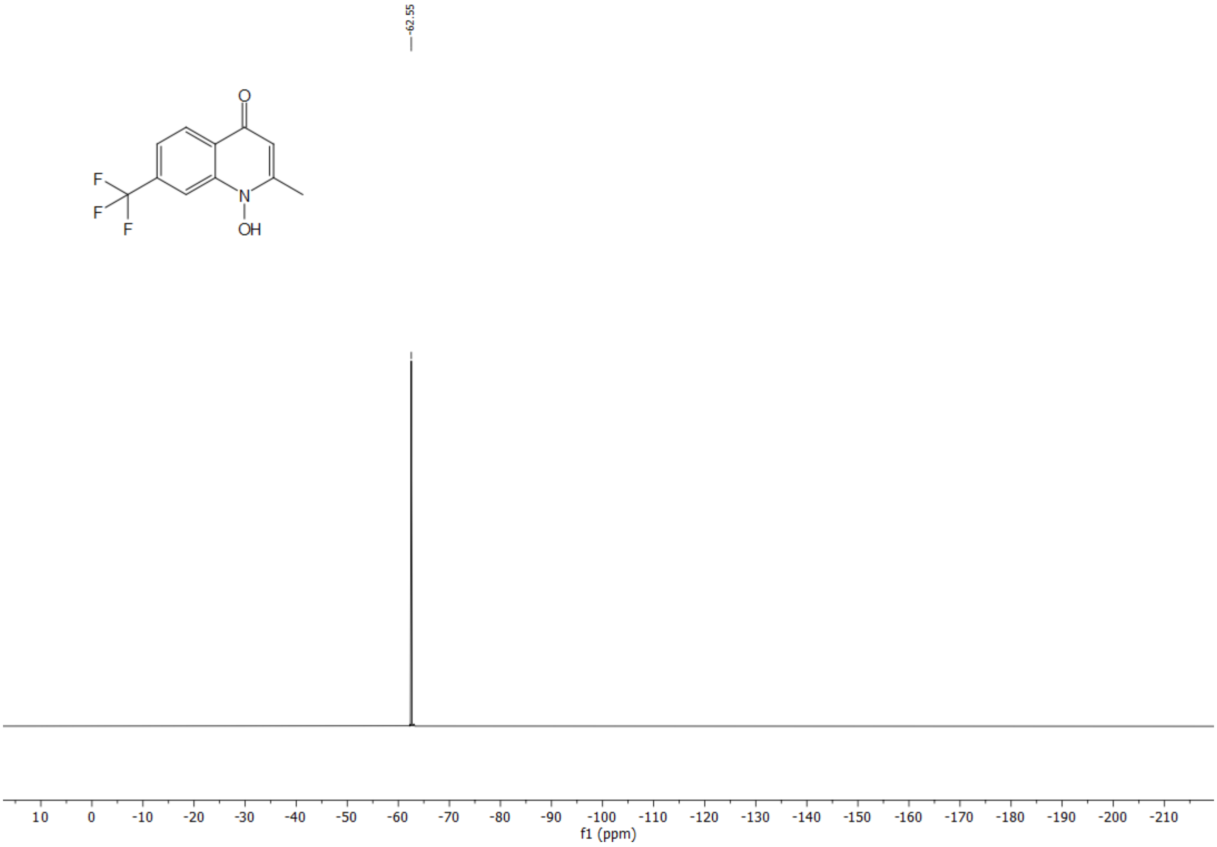

^{1}H NMR spectrum (600 MHz, DMSO-d_6) of **5o**

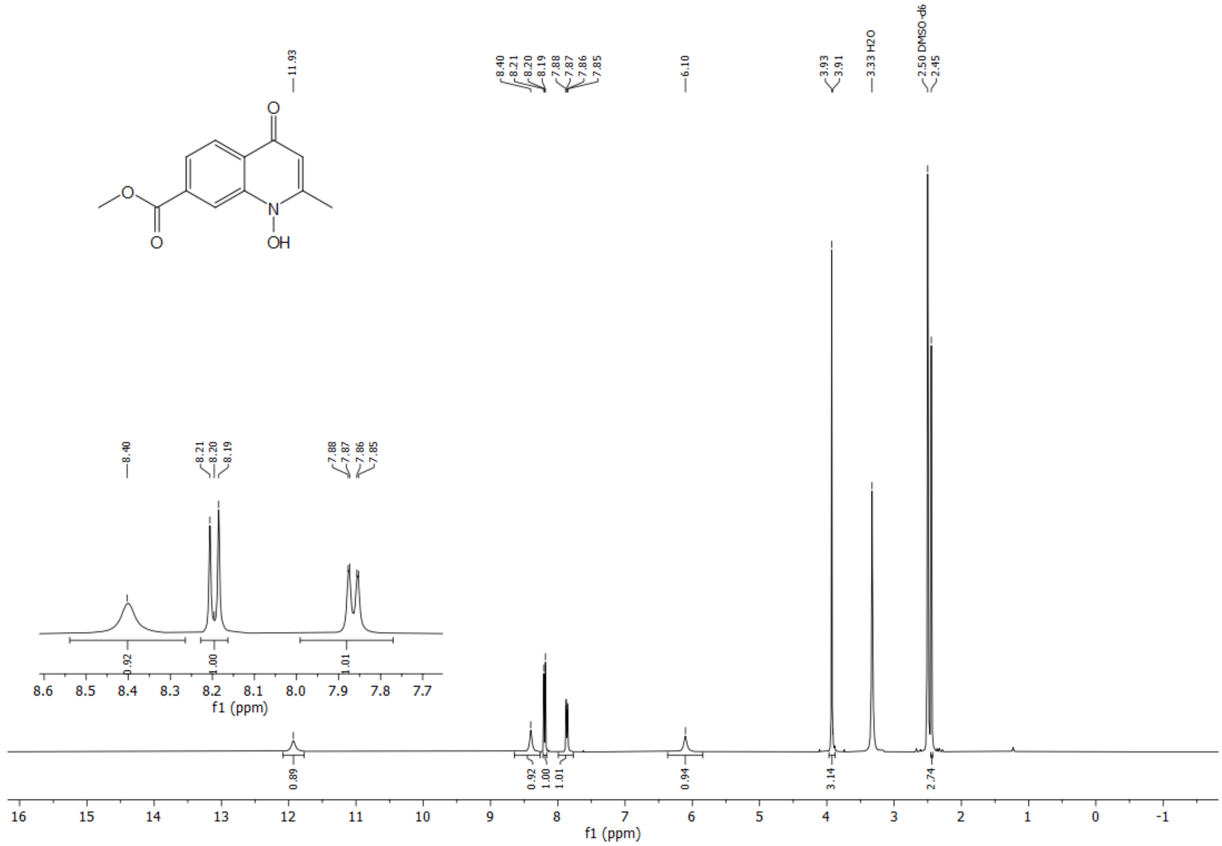

^{13}C NMR spectrum (151 MHz, DMSO-d_6) of **5o**

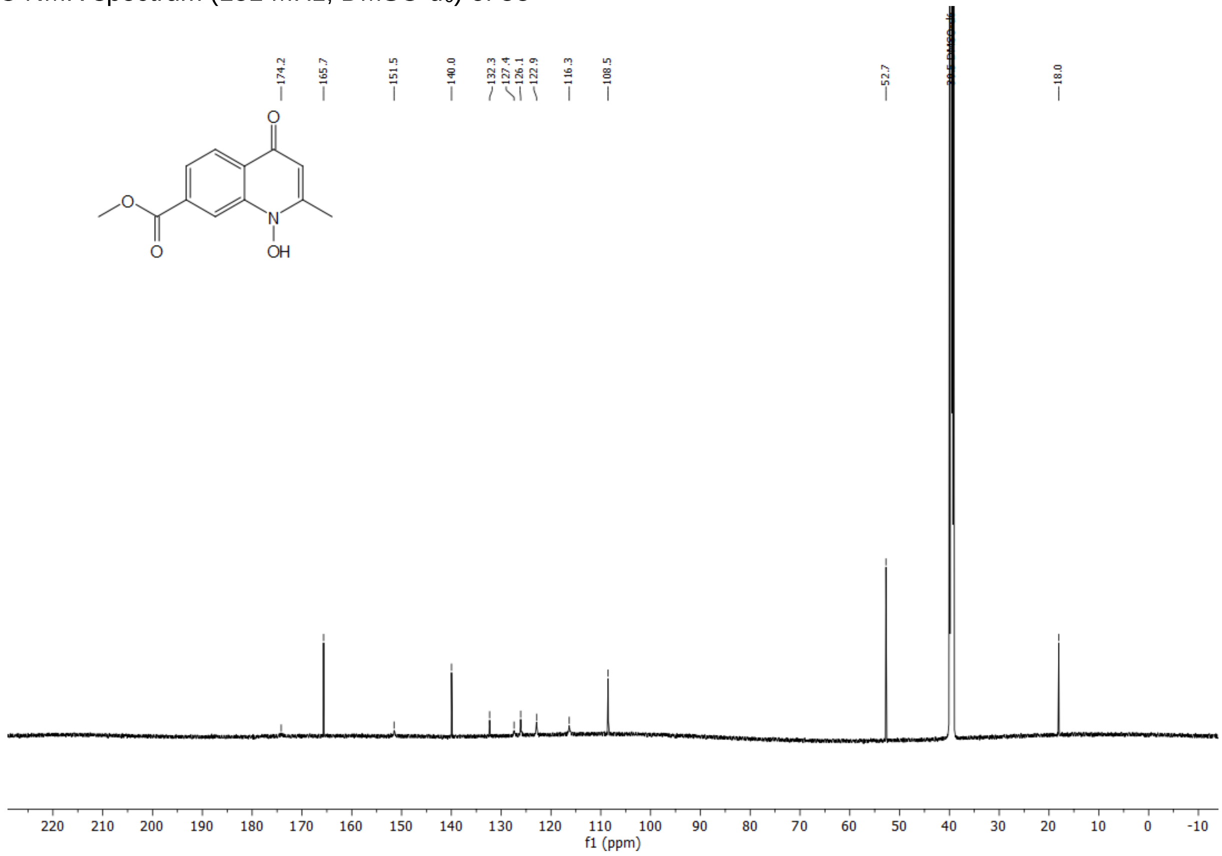

^{1}H NMR spectrum (400 MHz, DMSO-d_6) of **5p**

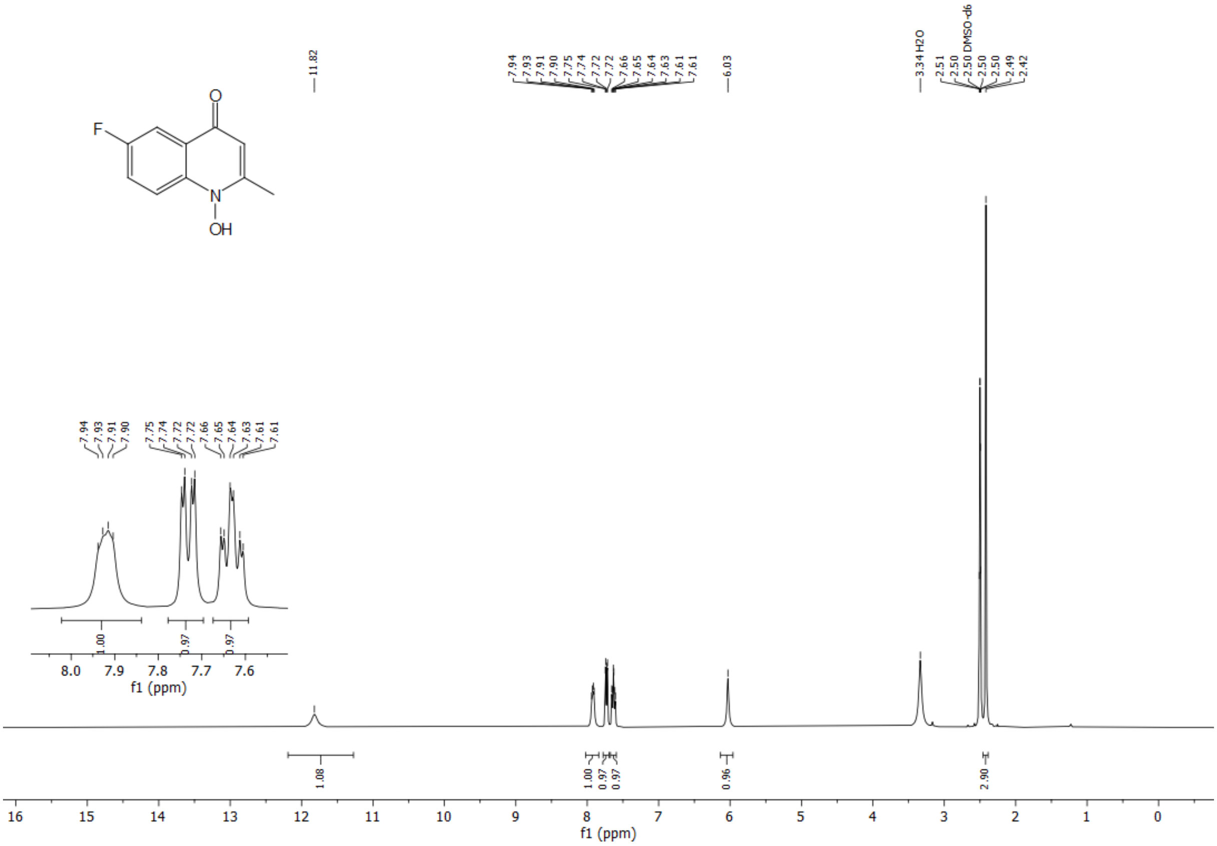

^{13}C NMR spectrum (101 MHz, DMSO-d_6) of **5p**

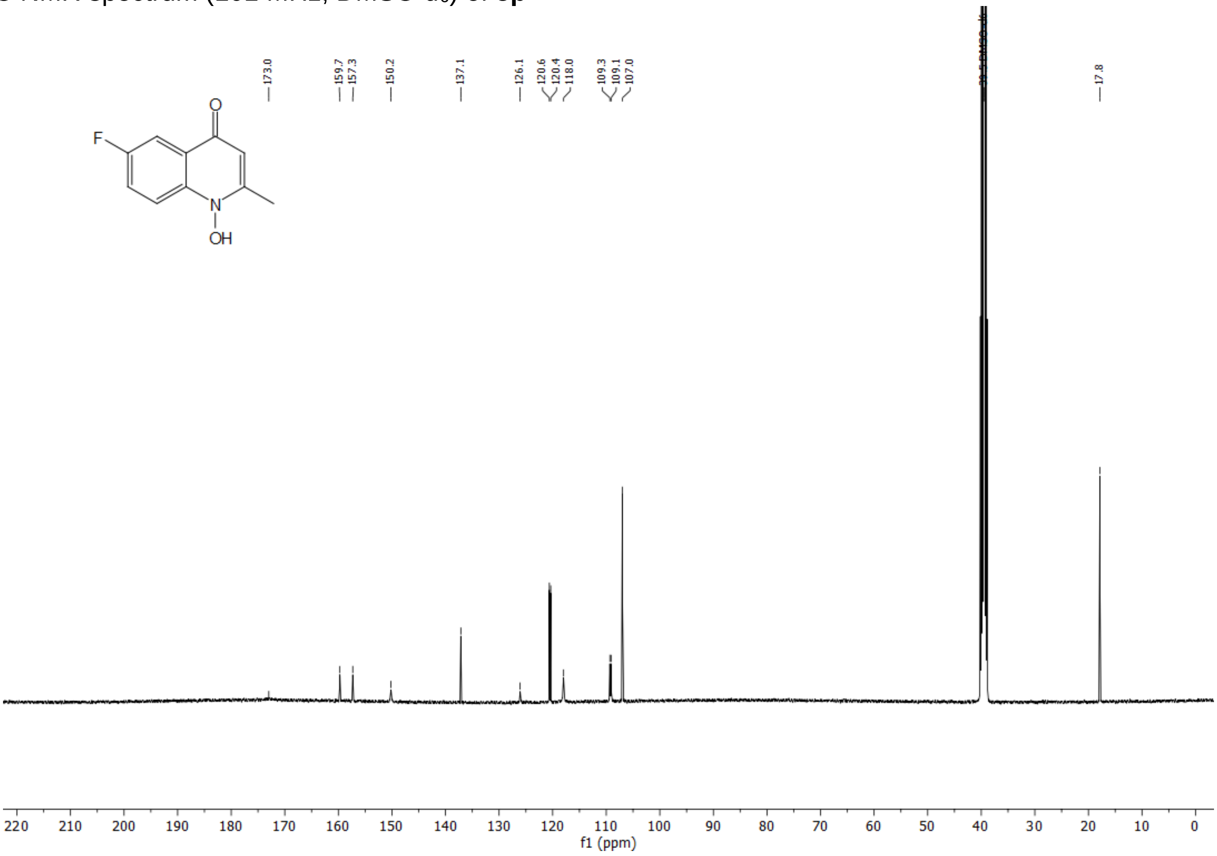

^{19}F NMR spectrum (376 MHz, DMSO-d_6) of **5p**

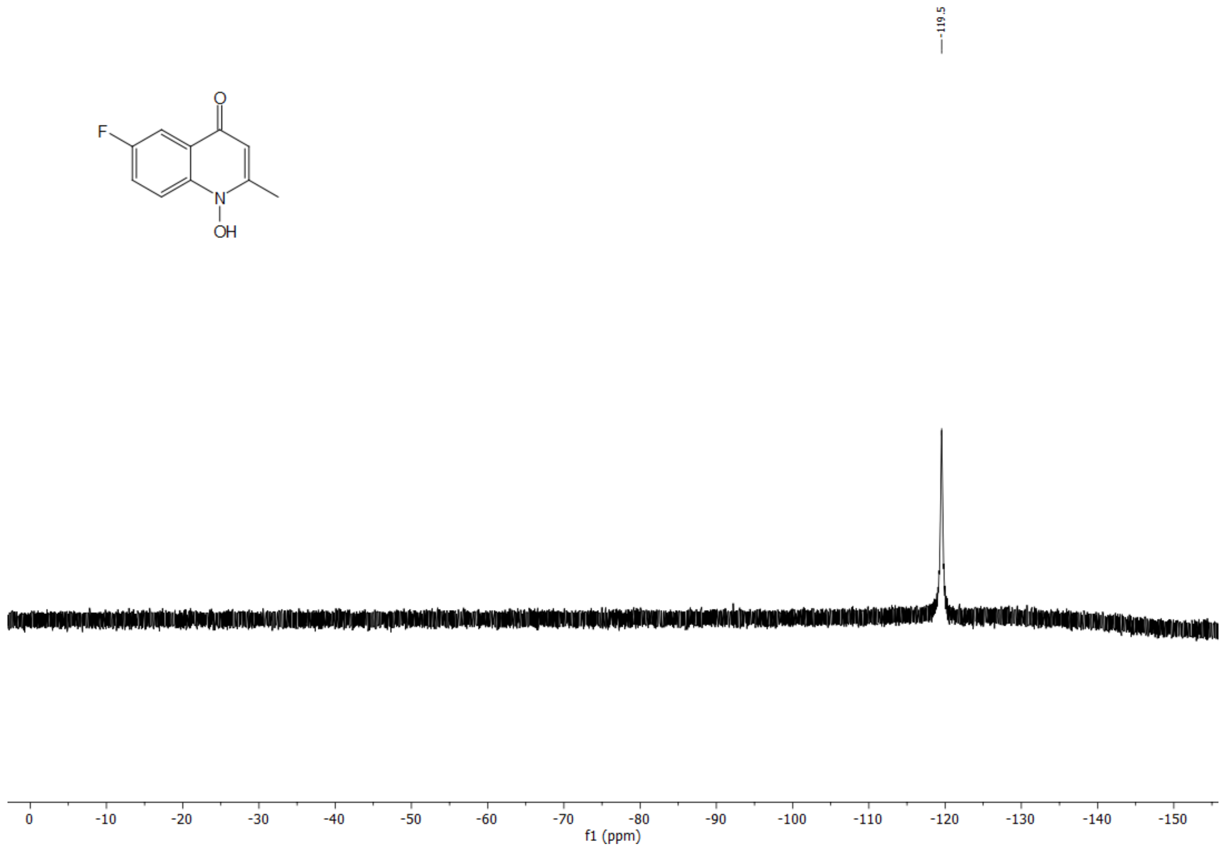

^{1}H NMR spectrum (400 MHz, DMSO-d_6) of **5q**

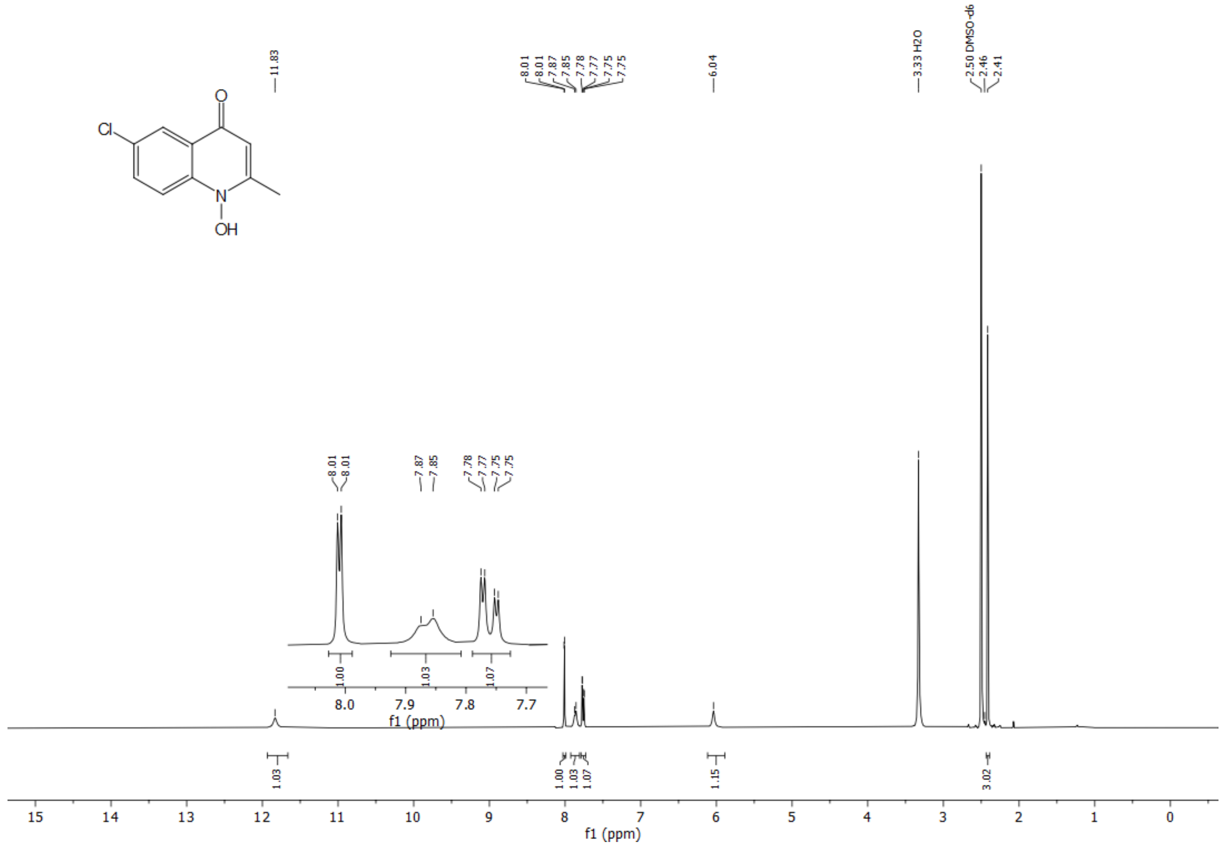

^{13}C NMR spectrum (101 MHz, DMSO-d_6) of **5q**

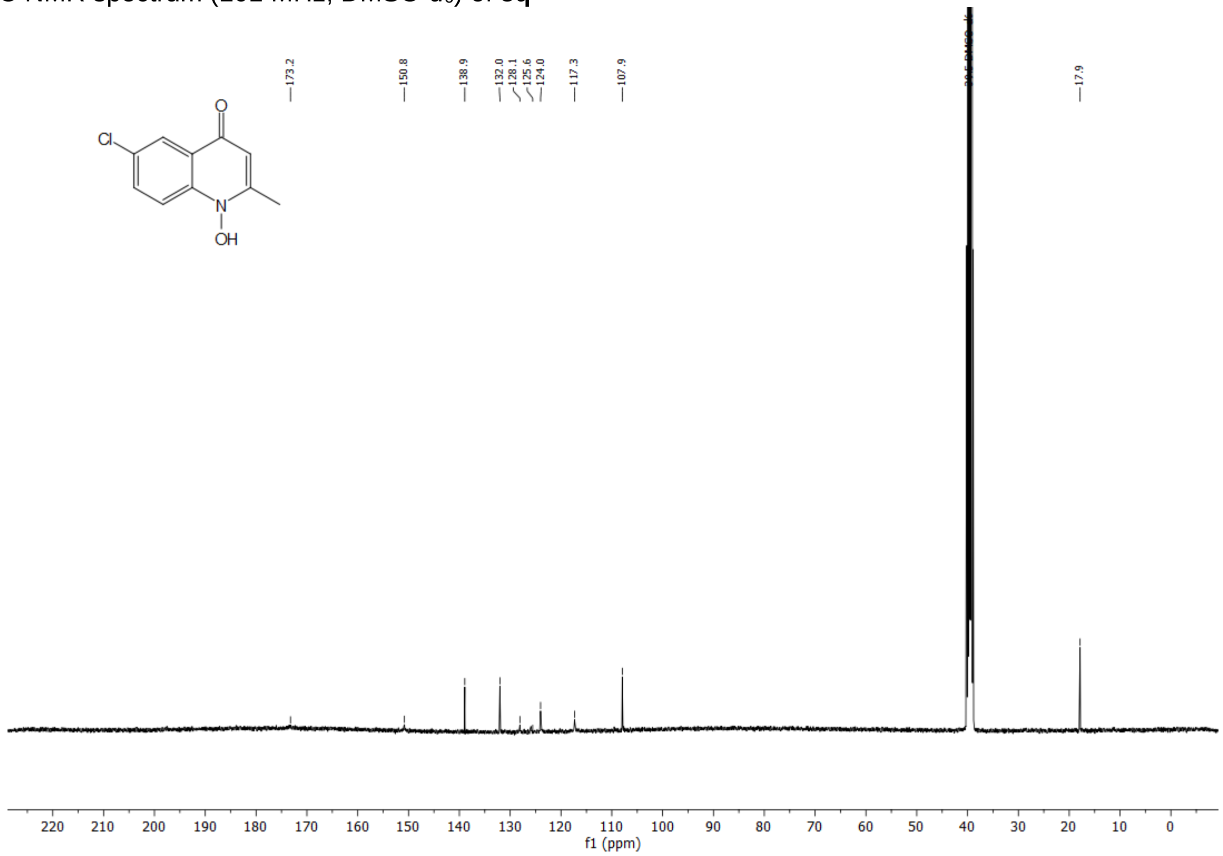

^{1}H NMR spectrum (400 MHz, DMSO-d_6) of **5r**

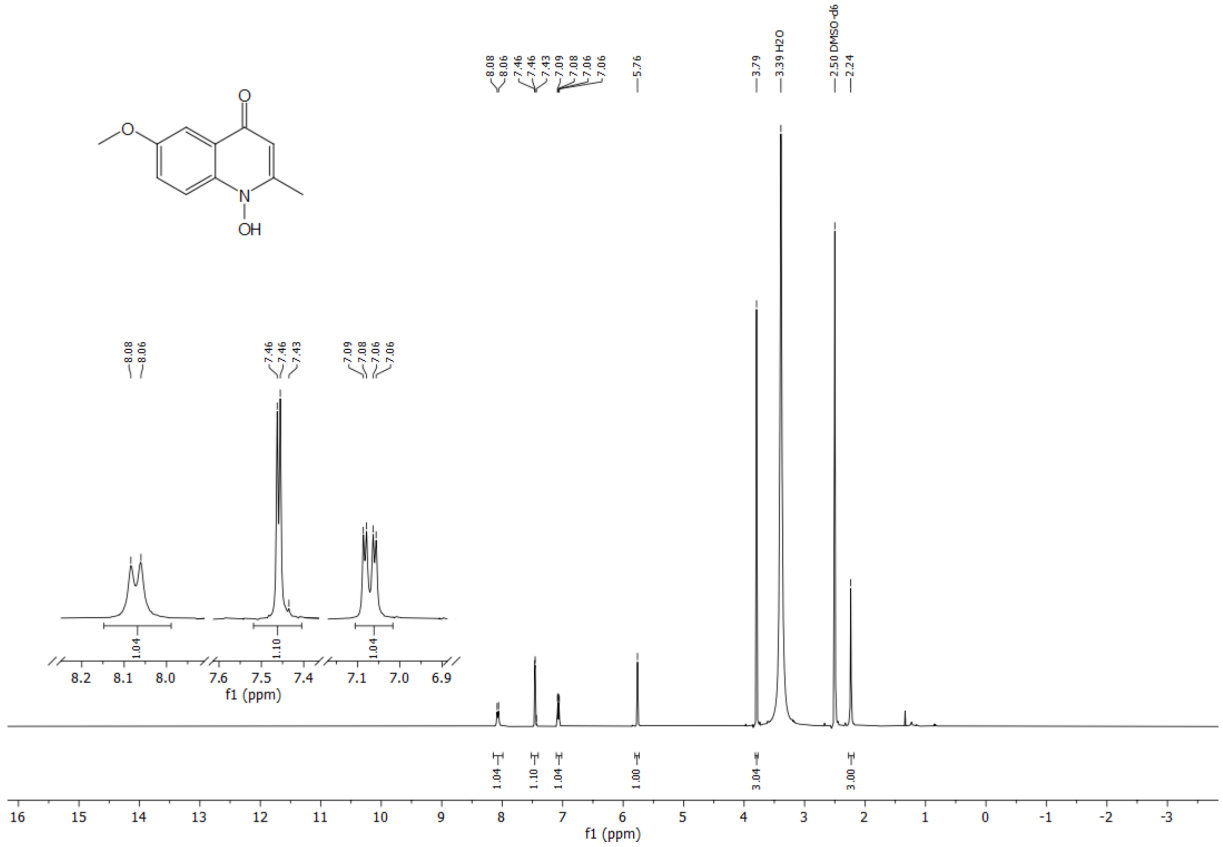

^{13}C NMR spectrum (101 MHz, DMSO-d_6) of **5r**

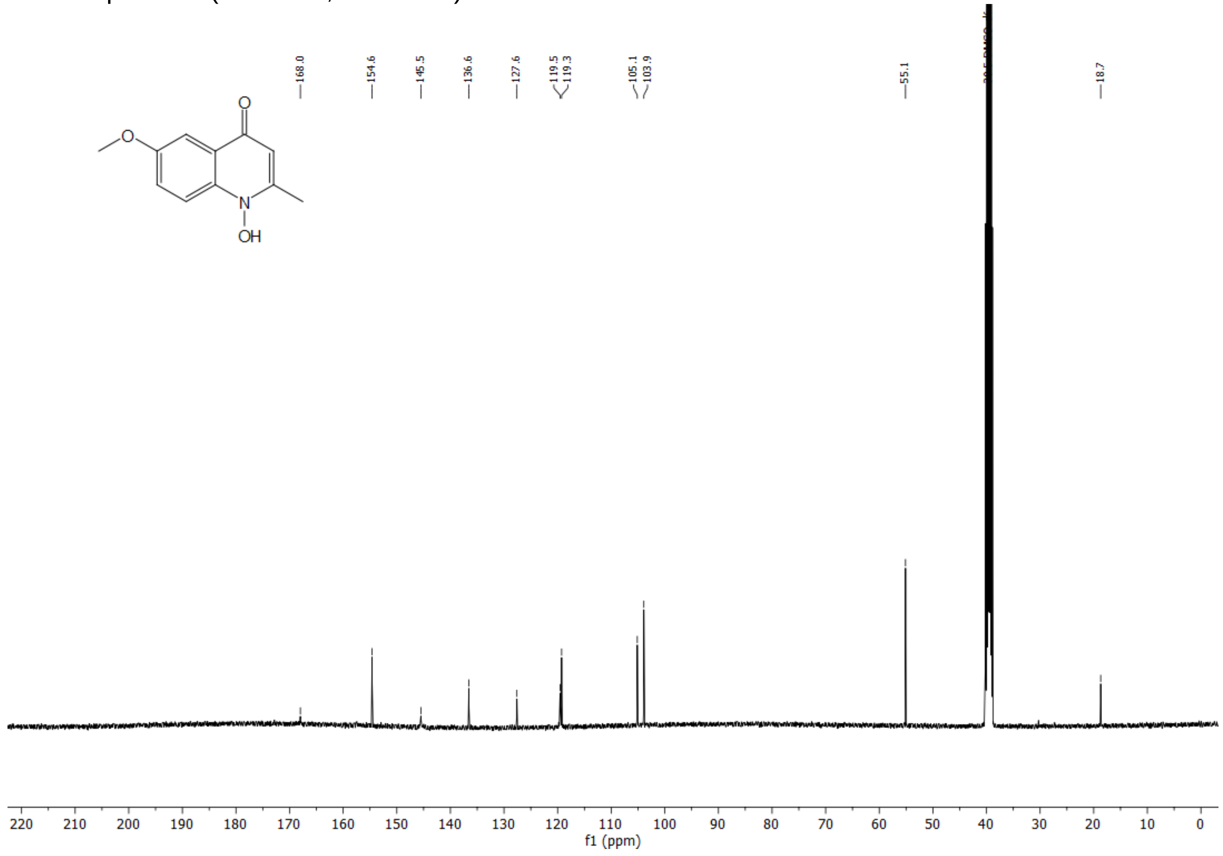

^{1}H NMR spectrum (400 MHz, D_2O+NaOD) of **5s**

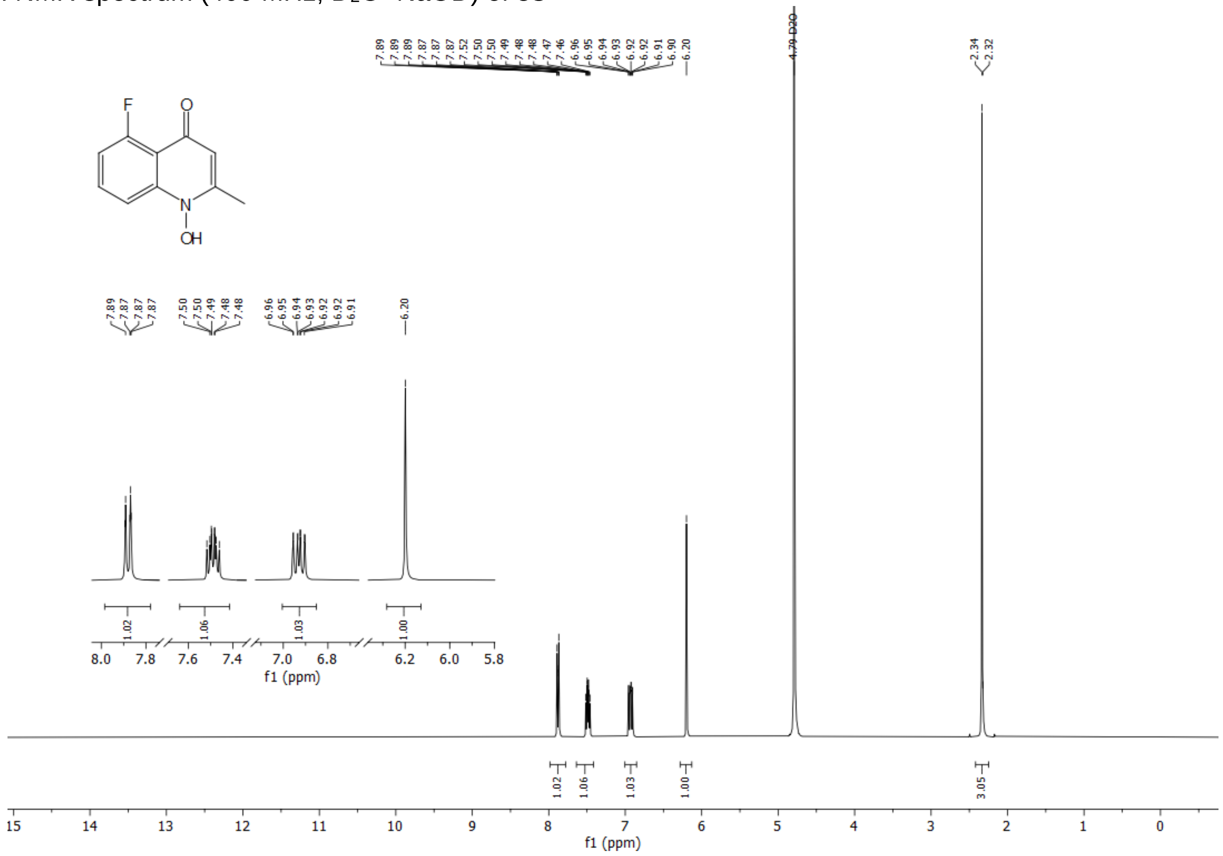

^{13}C NMR spectrum (101 MHz, D_2O+NaOD) of **5s**

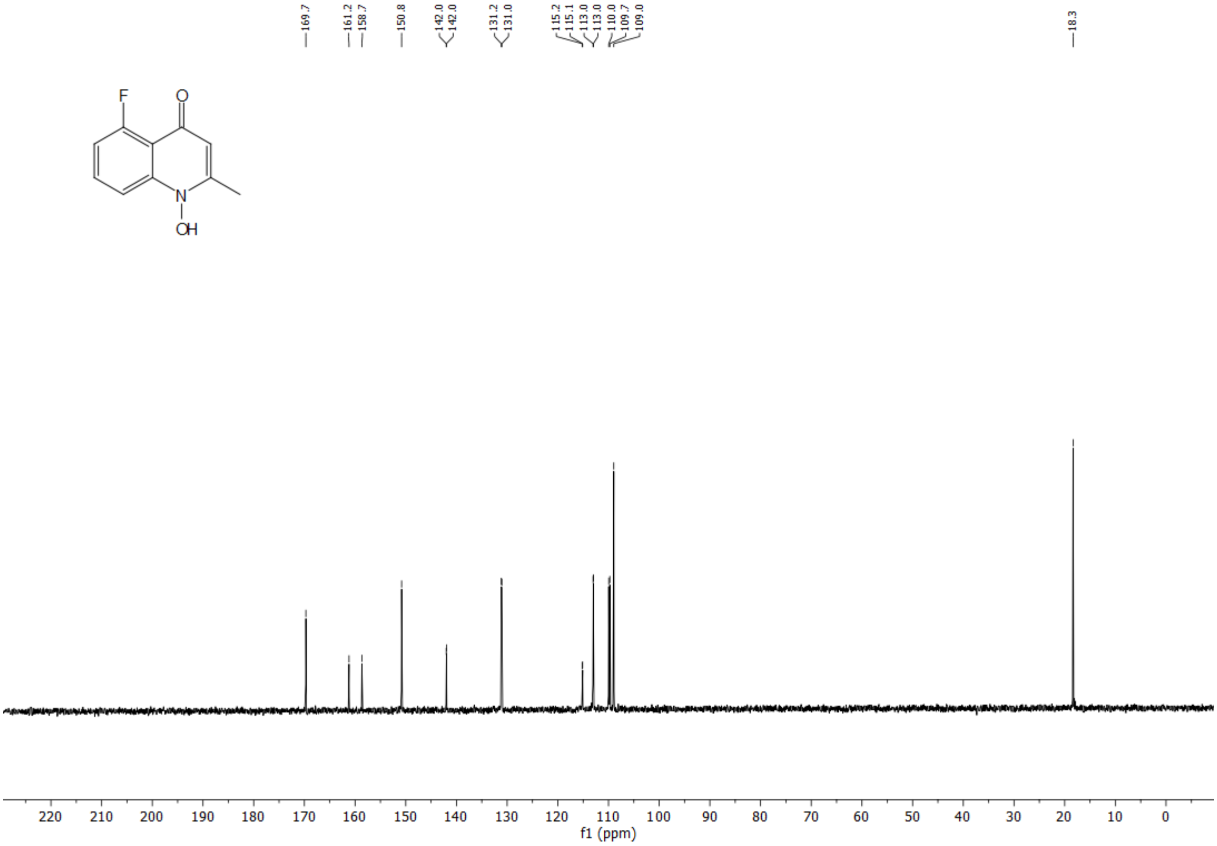

^{19}F NMR spectrum (376 MHz, D_2O+NaOD) of **5s**

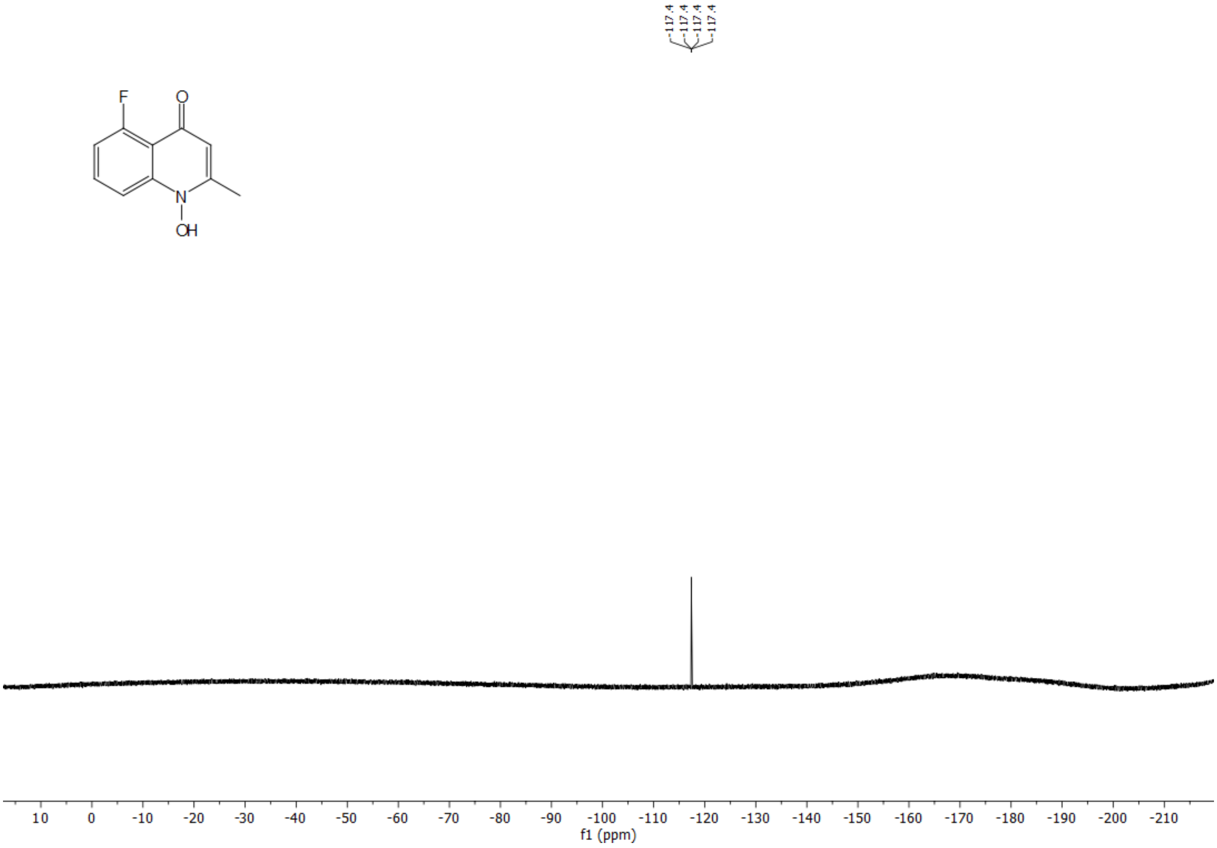

^{1}H NMR spectrum (400 MHz, DMSO-d_6) of **5t**

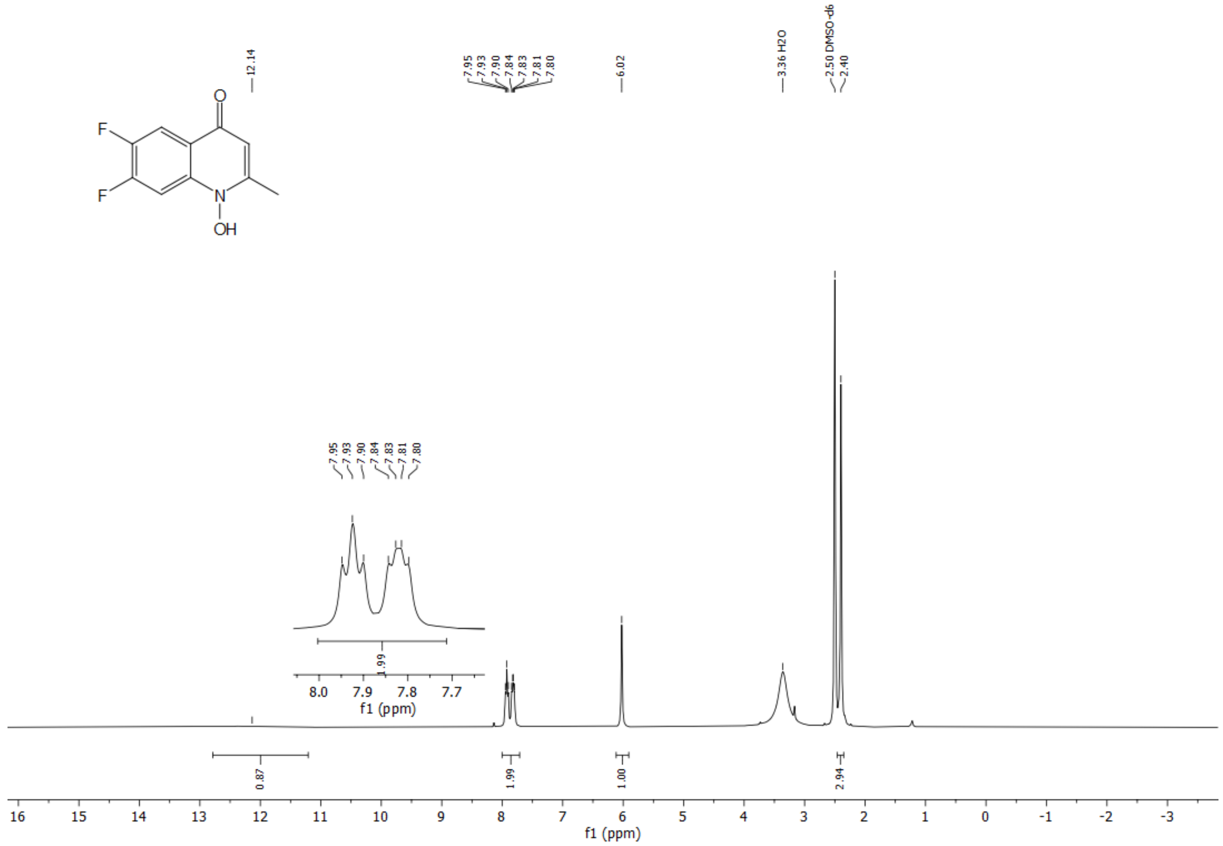

^{13}C NMR spectrum (101 MHz, DMSO-d_6) of **5t**

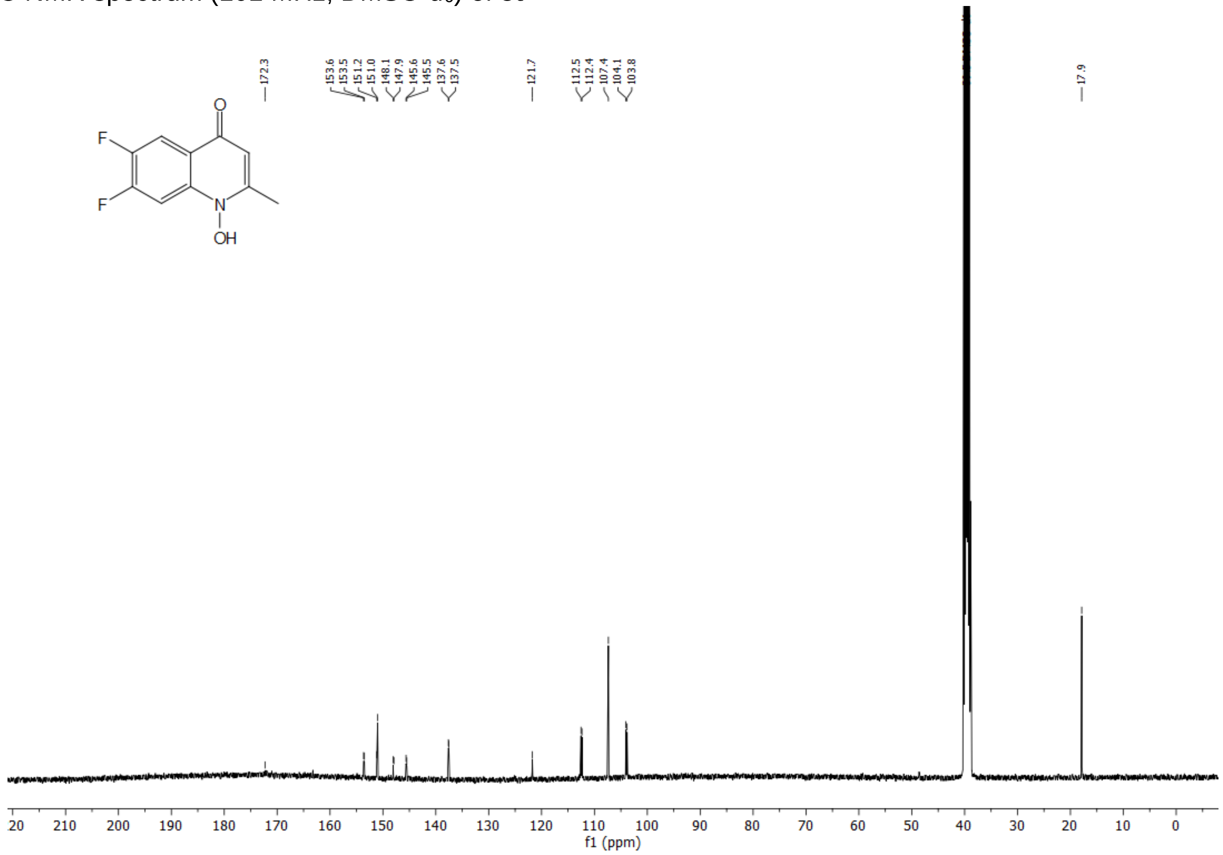

^{19}F NMR spectrum (376 MHz, DMSO-d_6) of **5t**

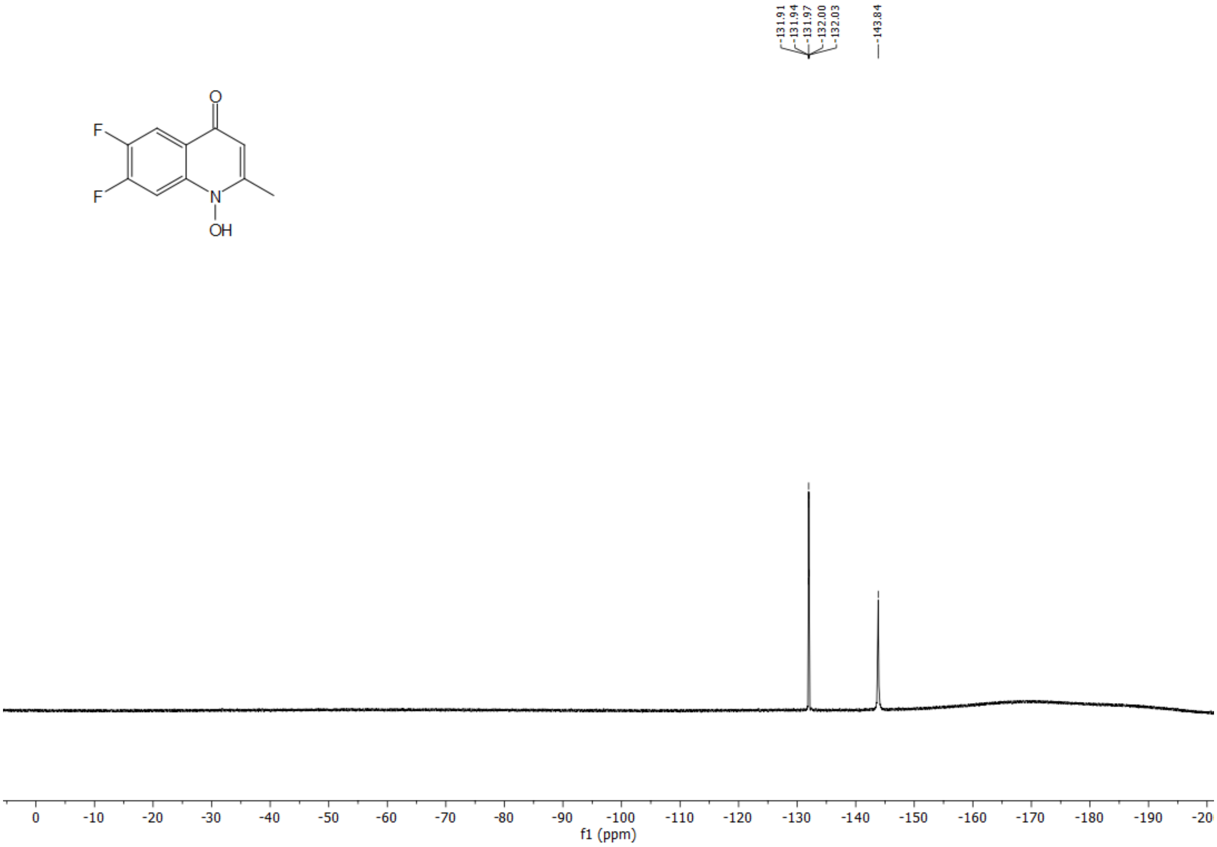

^{1}H NMR spectrum (400 MHz, DMSO-d_6) of **5u**

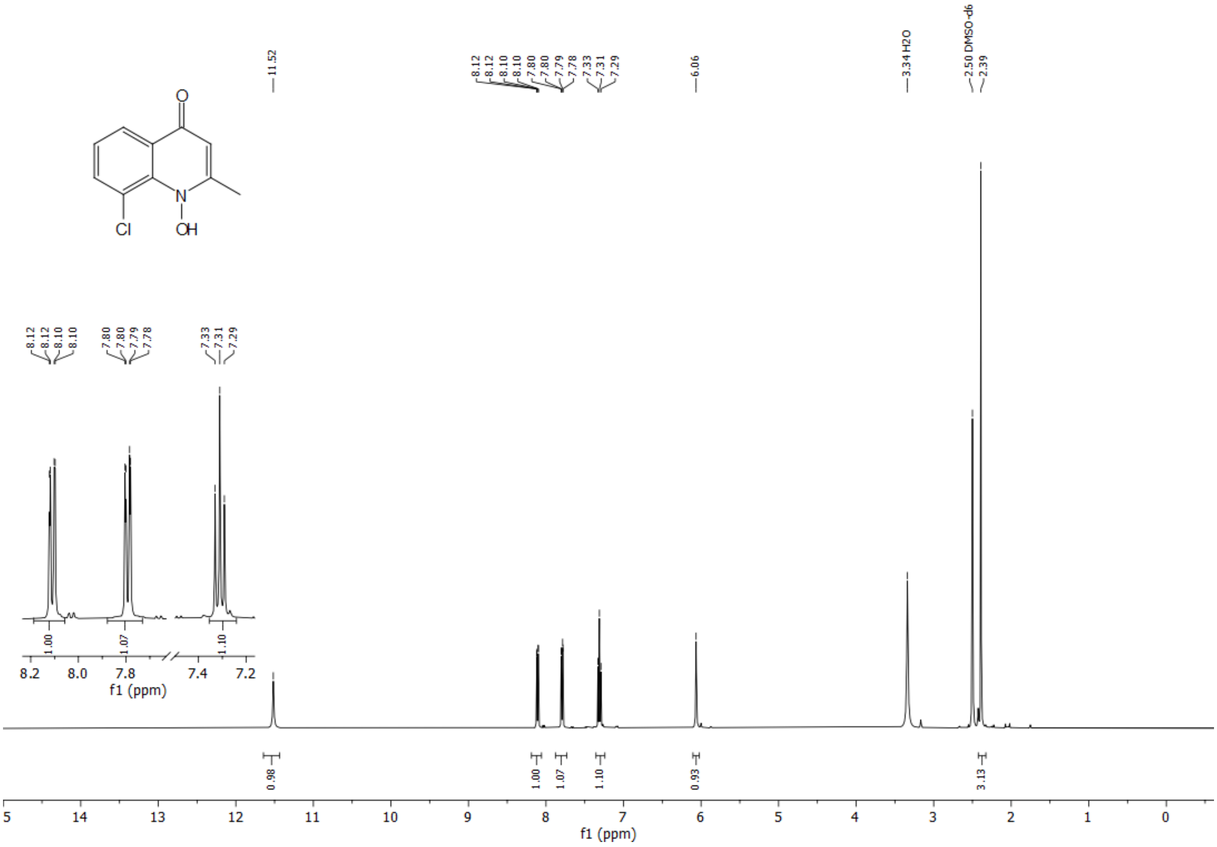

^{13}C NMR spectrum (101 MHz, DMSO-d_6) of **5u**

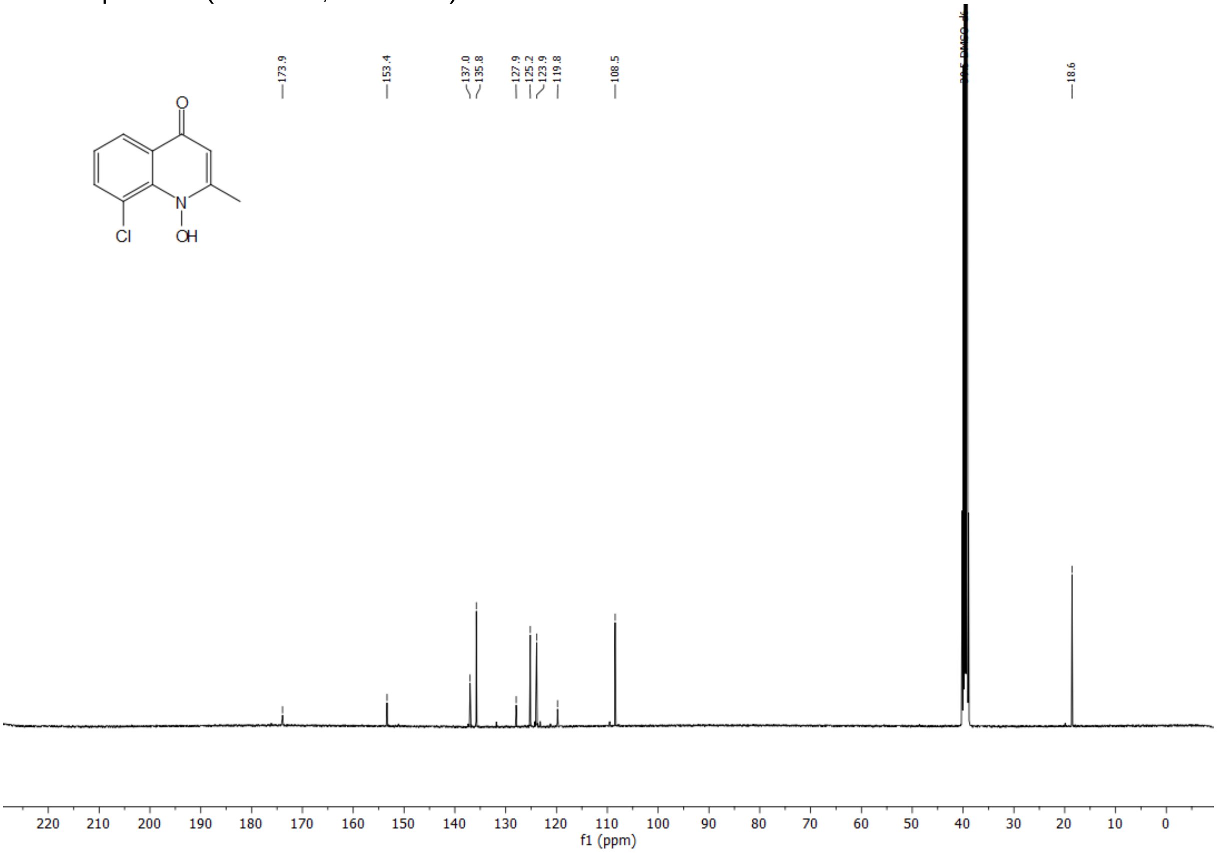

^{1}H NMR spectrum (400 MHz, D_2O+NaOD) of **5v**

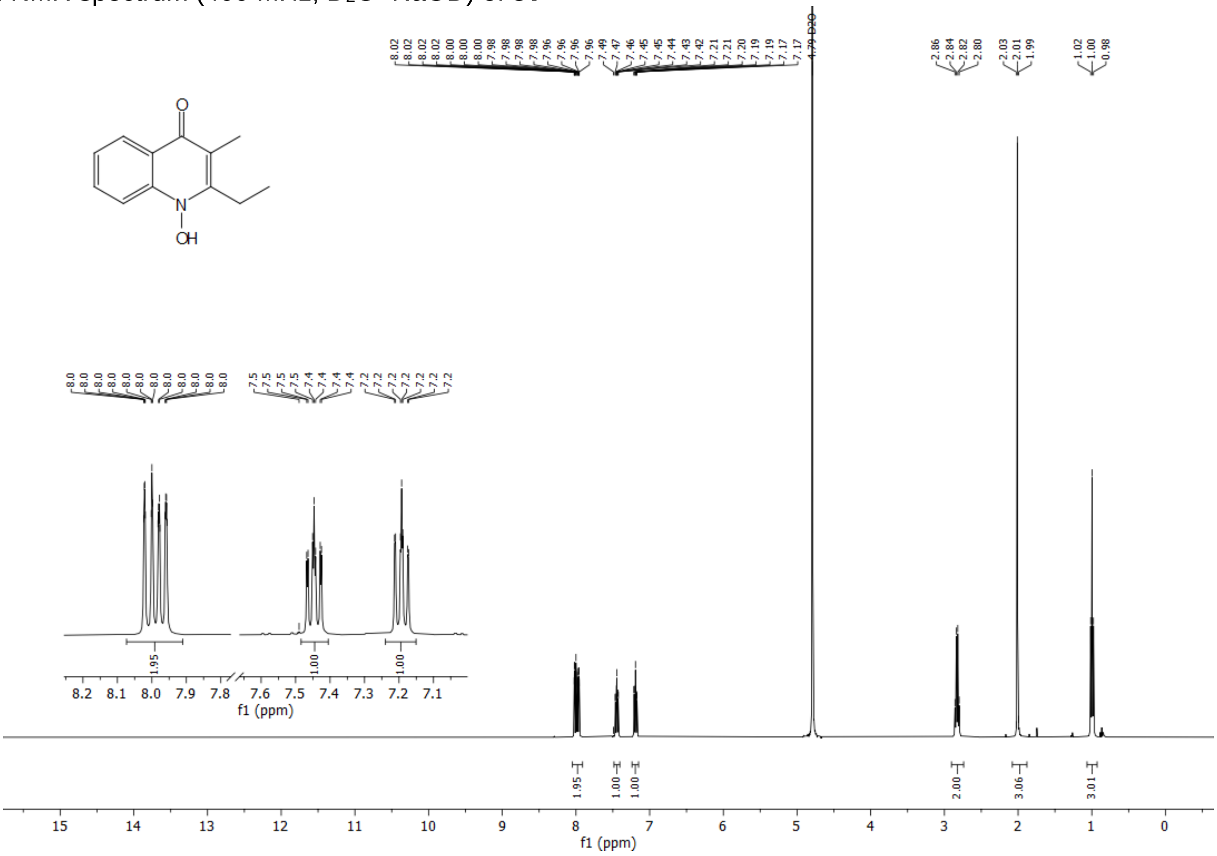

^{13}C NMR spectrum (101 MHz, D_2O+NaOD) of **5v**

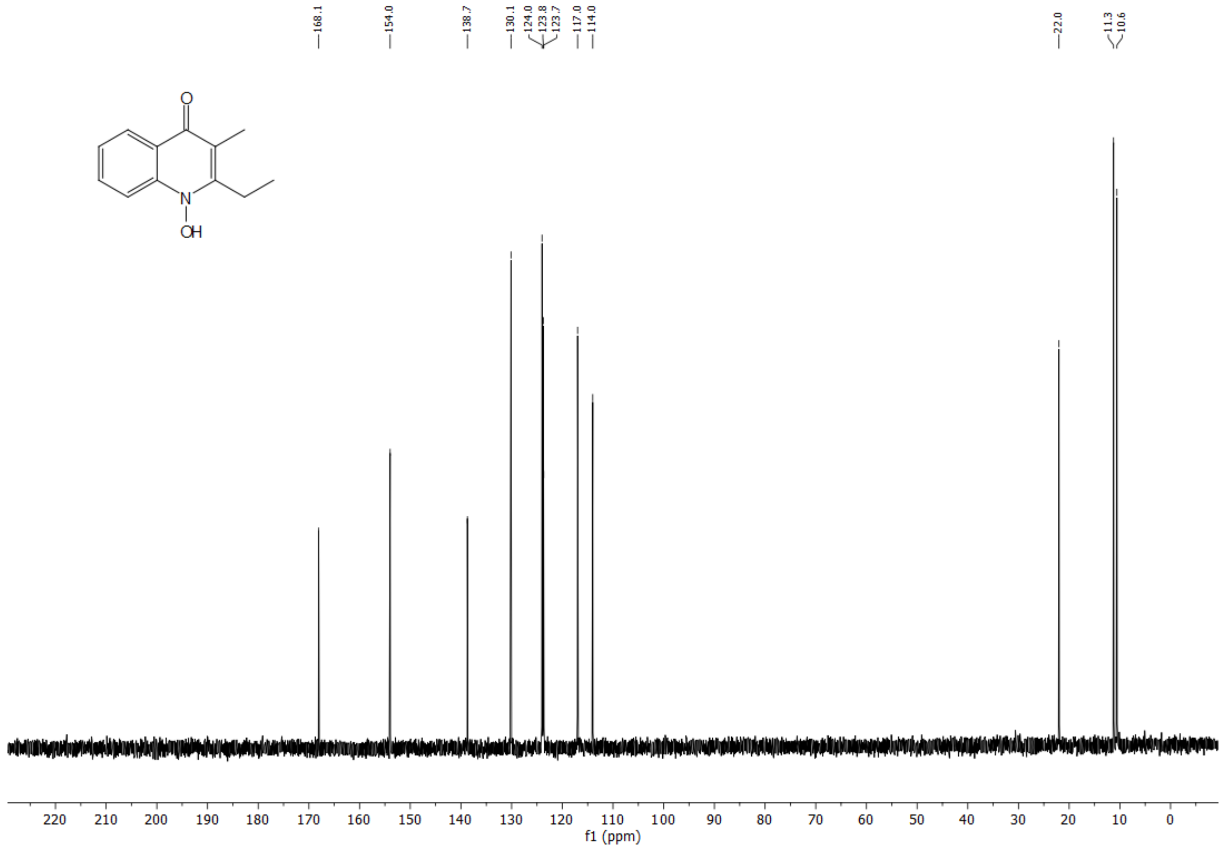

^{1}H NMR spectrum (600 MHz, DMSO-d_6) of **5w**

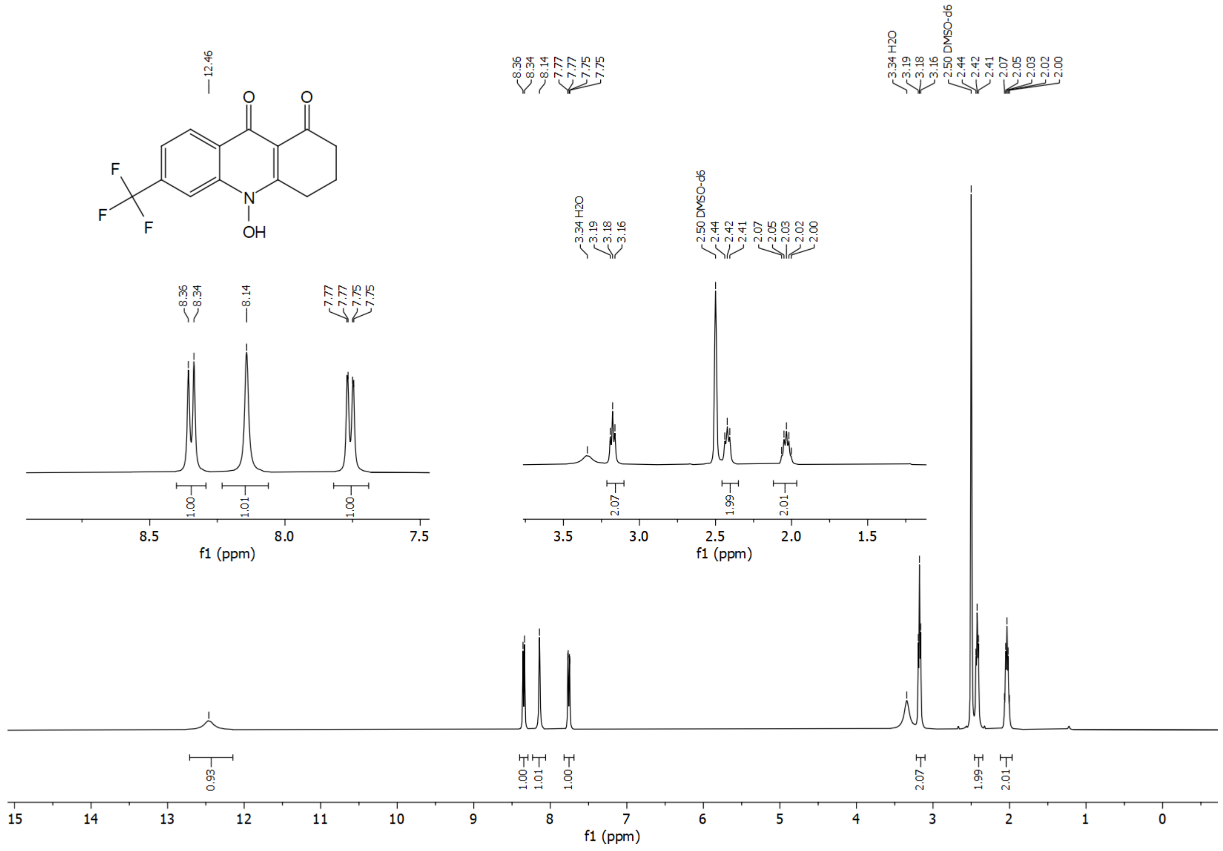

^{13}C NMR spectrum (151 MHz, DMSO-d_6) of **5w**

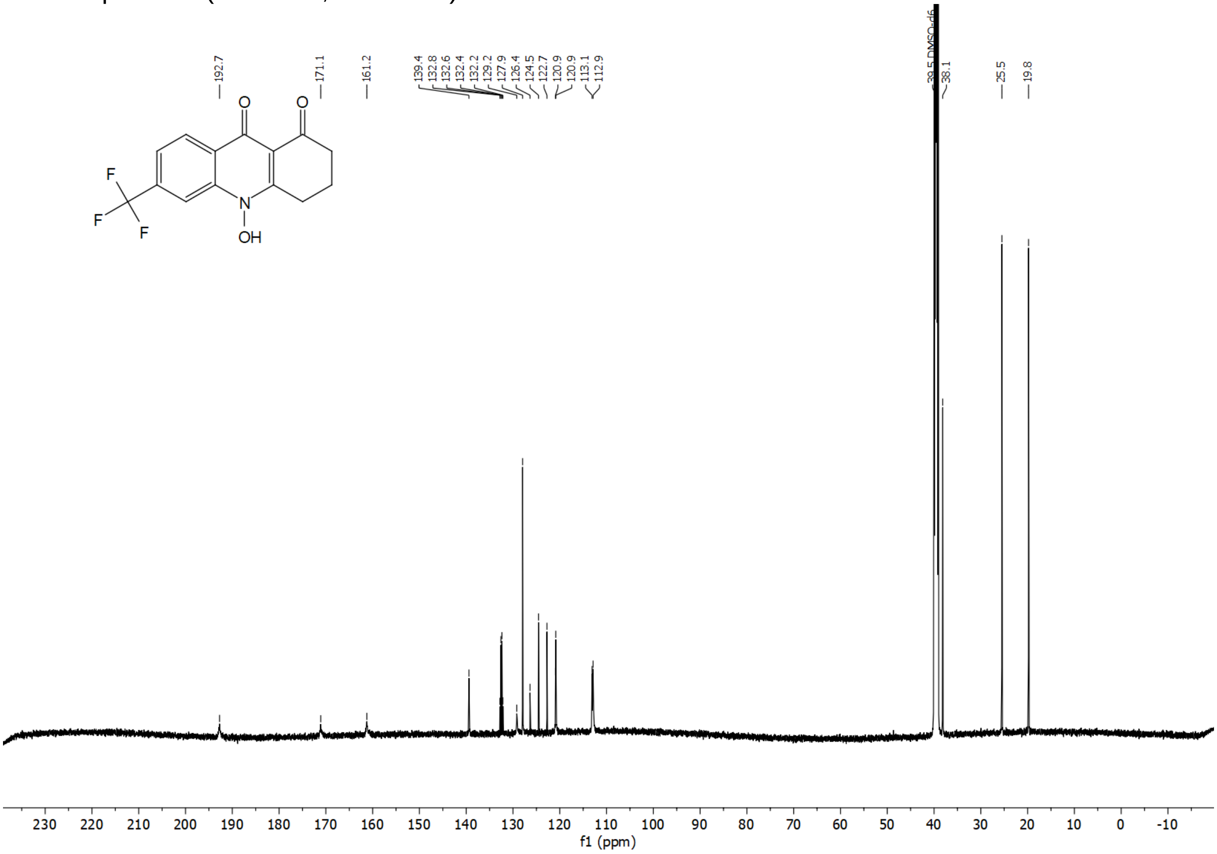

^{19}F NMR spectrum (376 MHz, DMSO-d_6) of **5w**

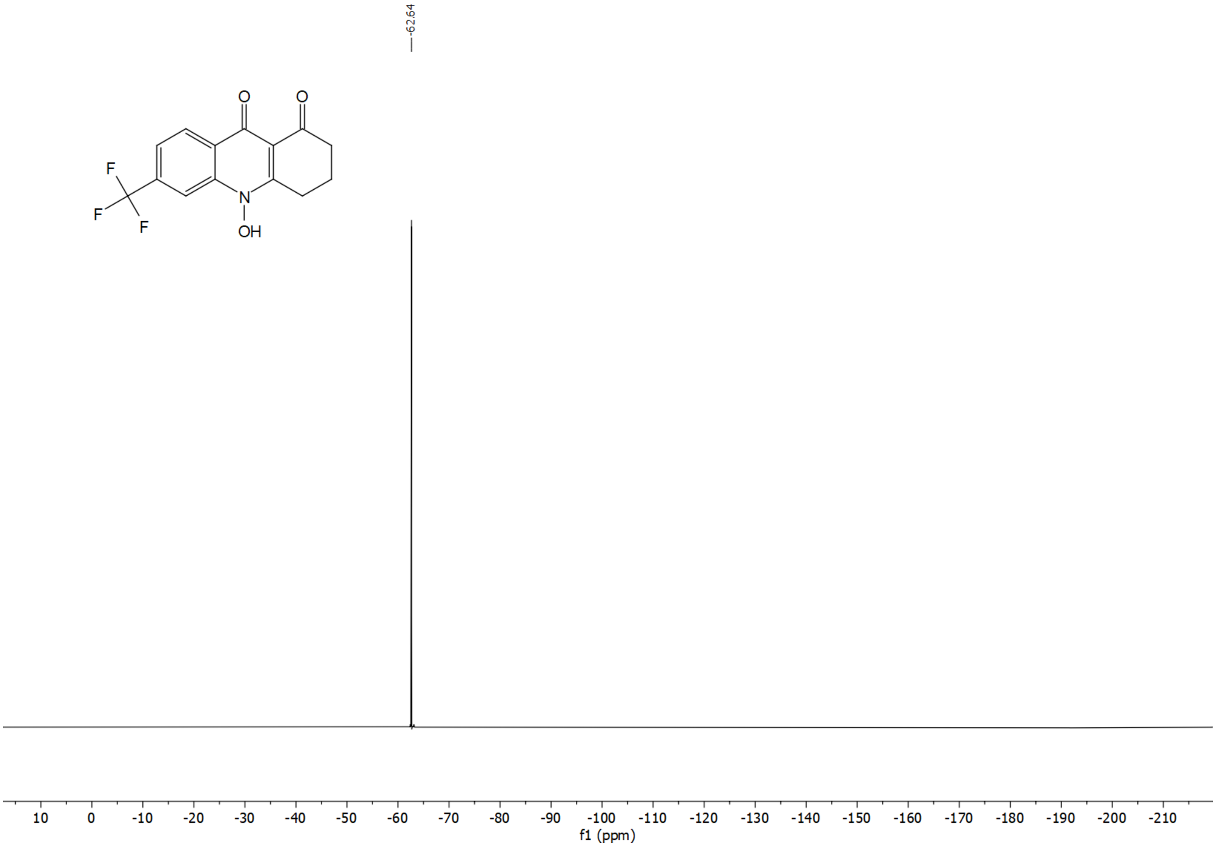

^{1}H NMR spectrum (400 MHz, DMSO-d_6) of **5y**

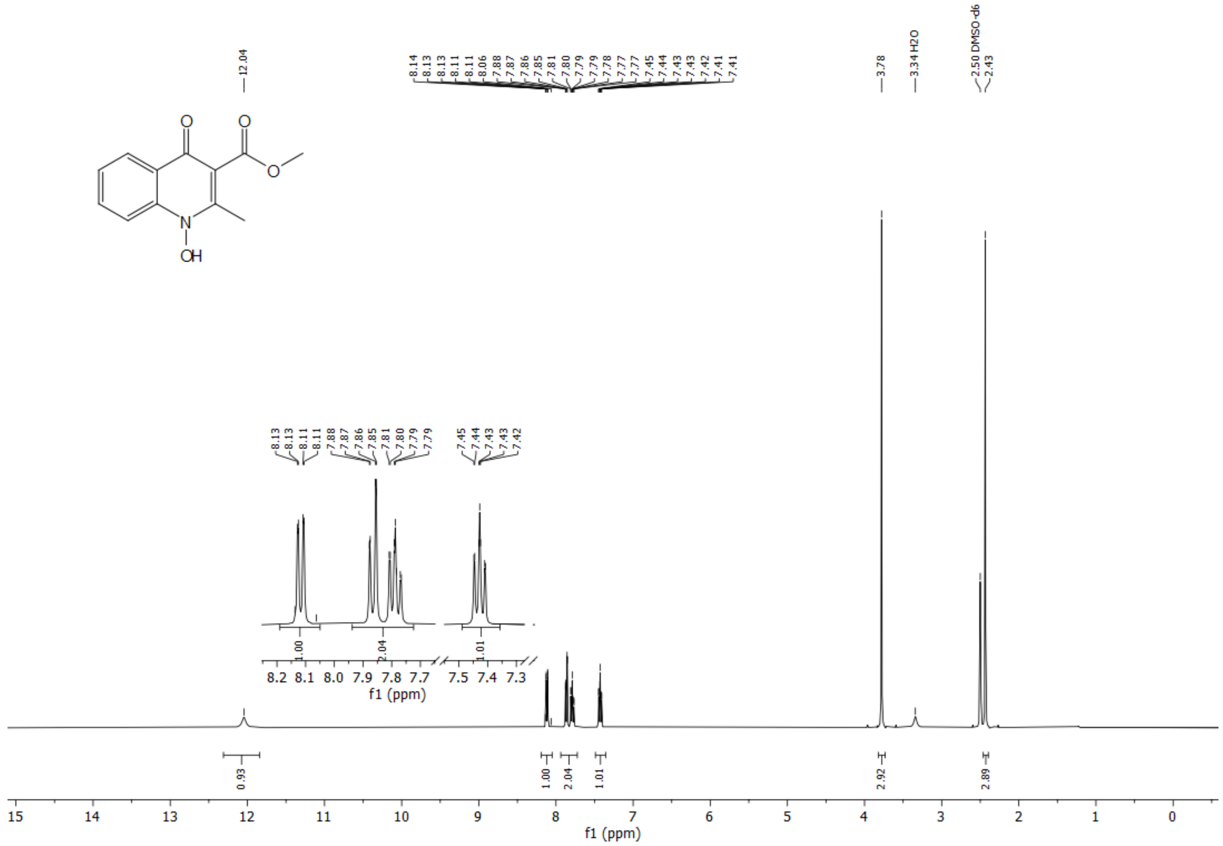

^{13}C NMR spectrum (101 MHz, DMSO-d_6) of **5y**

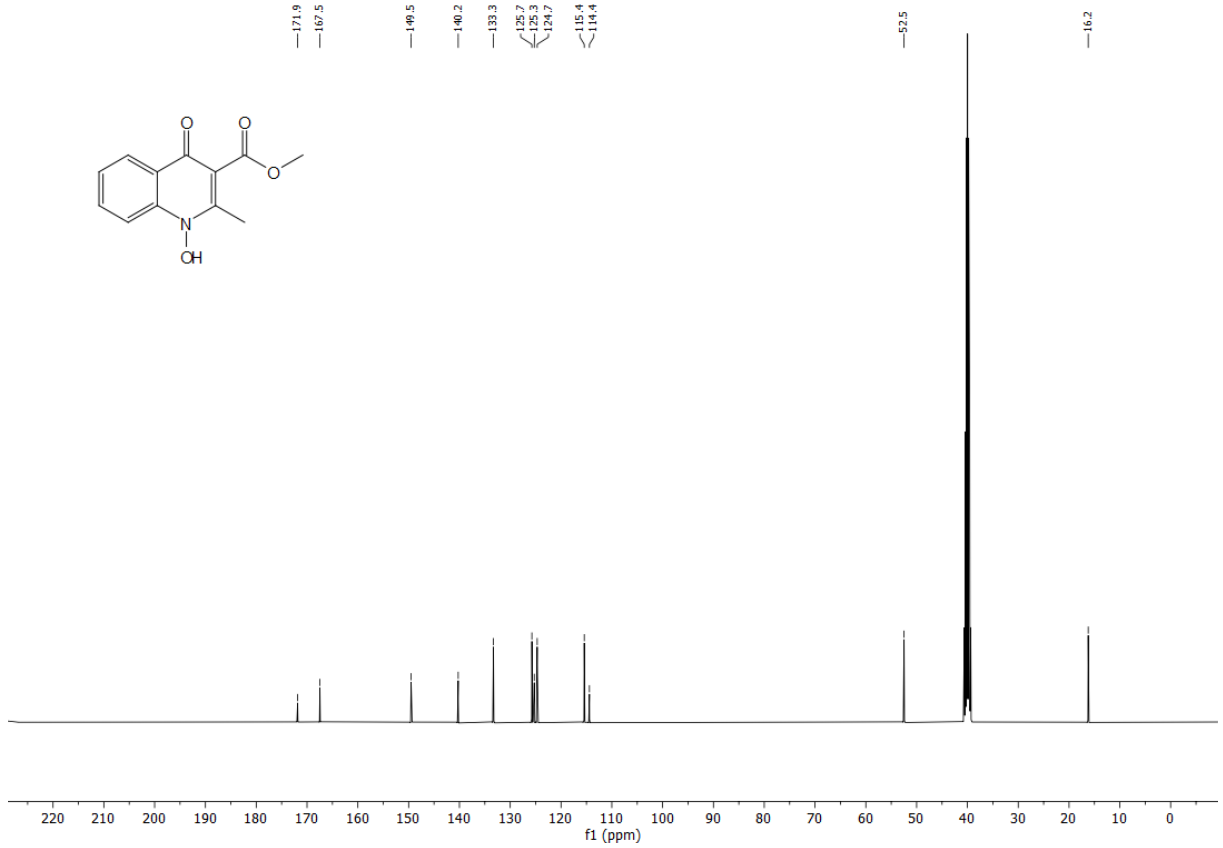

^{1}H NMR spectrum (400 MHz, DMSO-d_6) of **5z**

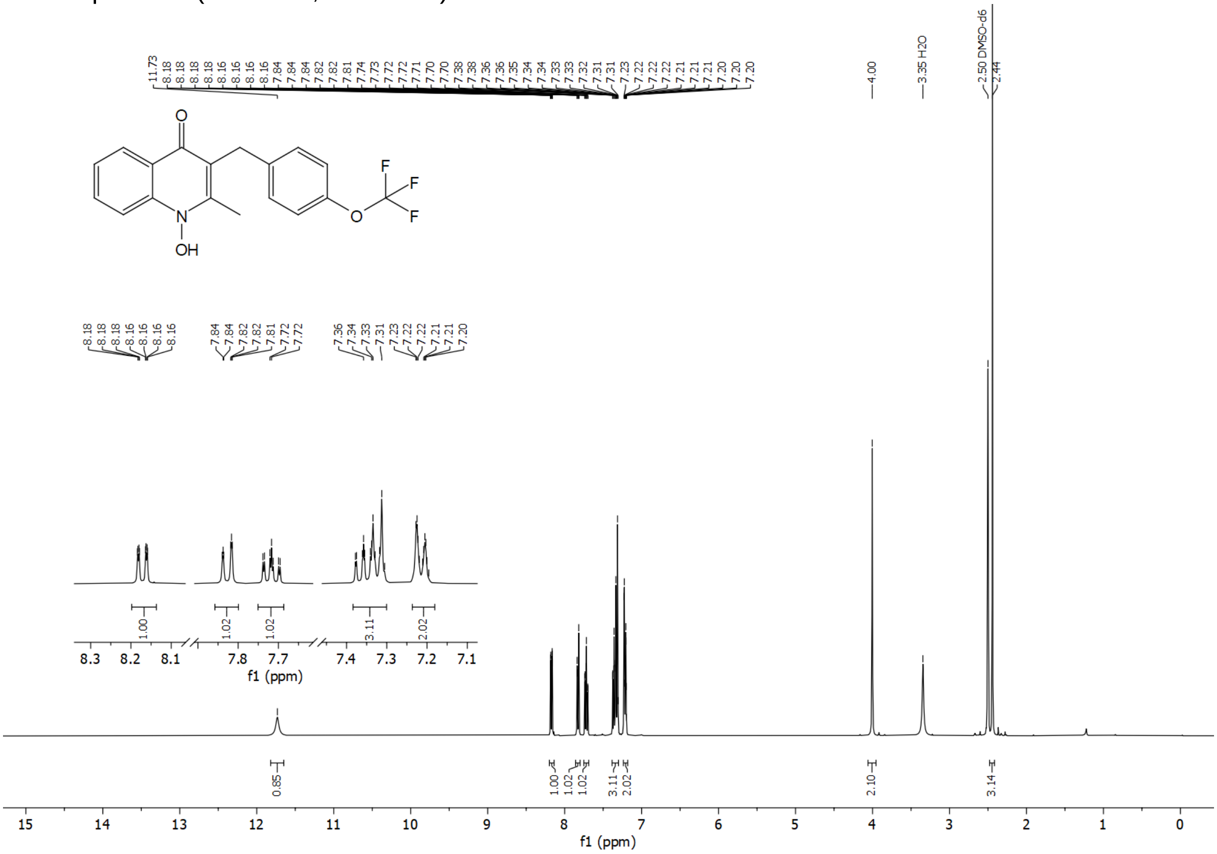

^{13}C NMR spectrum (101 MHz, DMSO-d_6) of **5z**

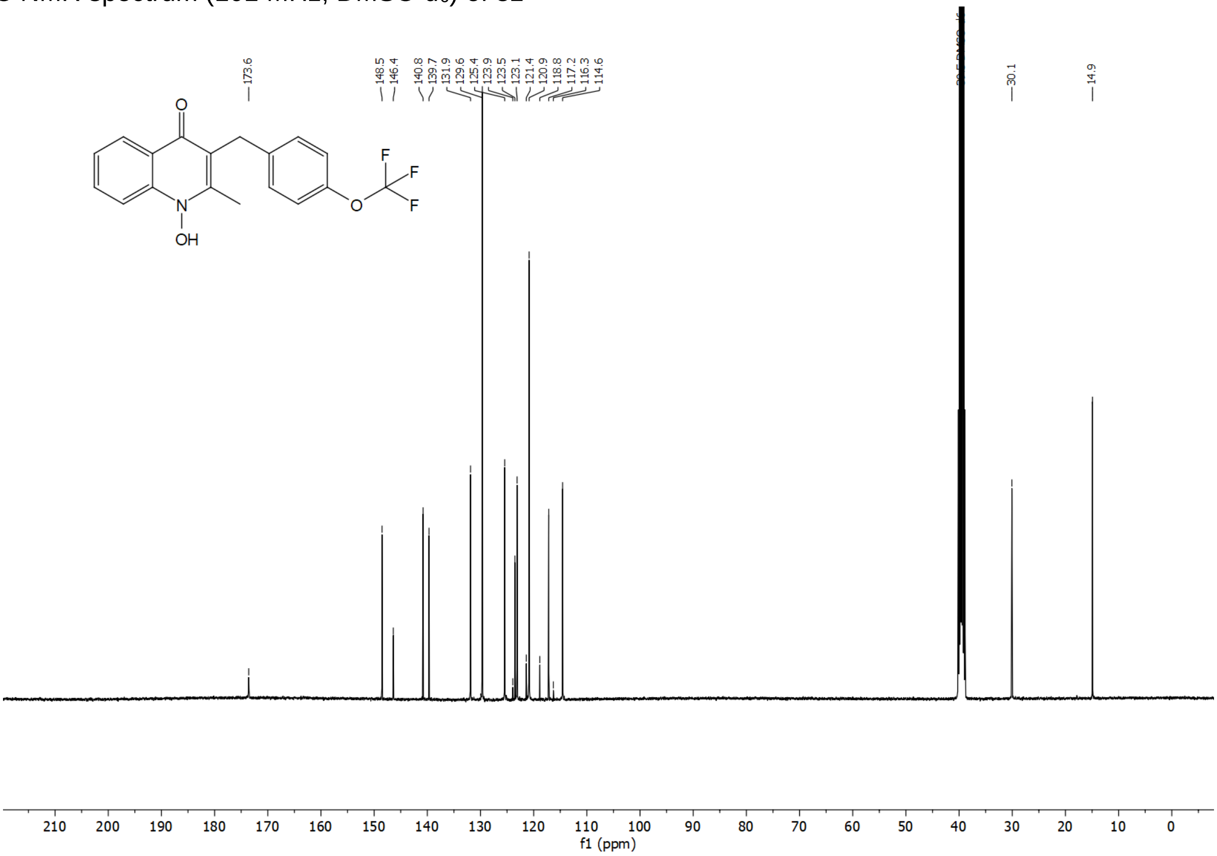

^{19}F NMR spectrum (376 MHz, DMSO-d_6) of **5z**

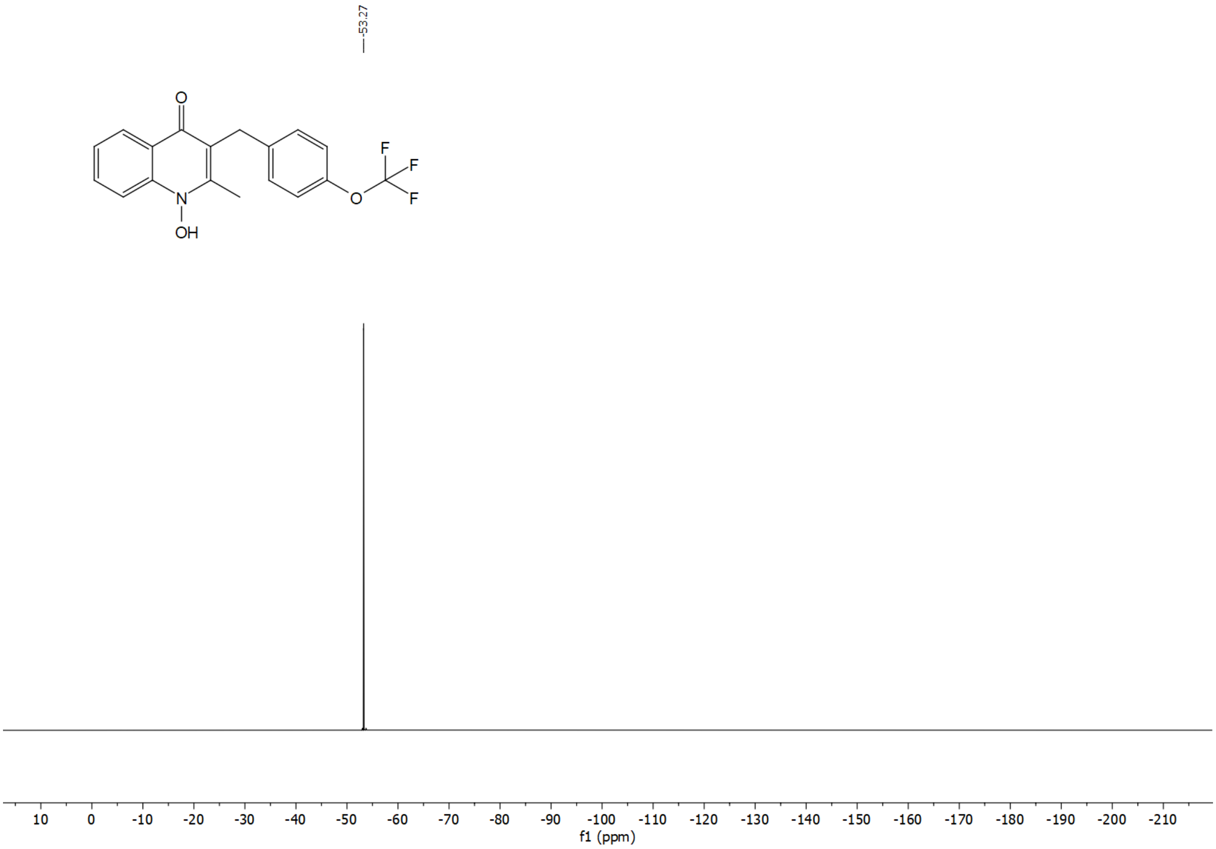

^{1}H NMR spectrum (400 MHz, DMSO-d_6) of **5aa**

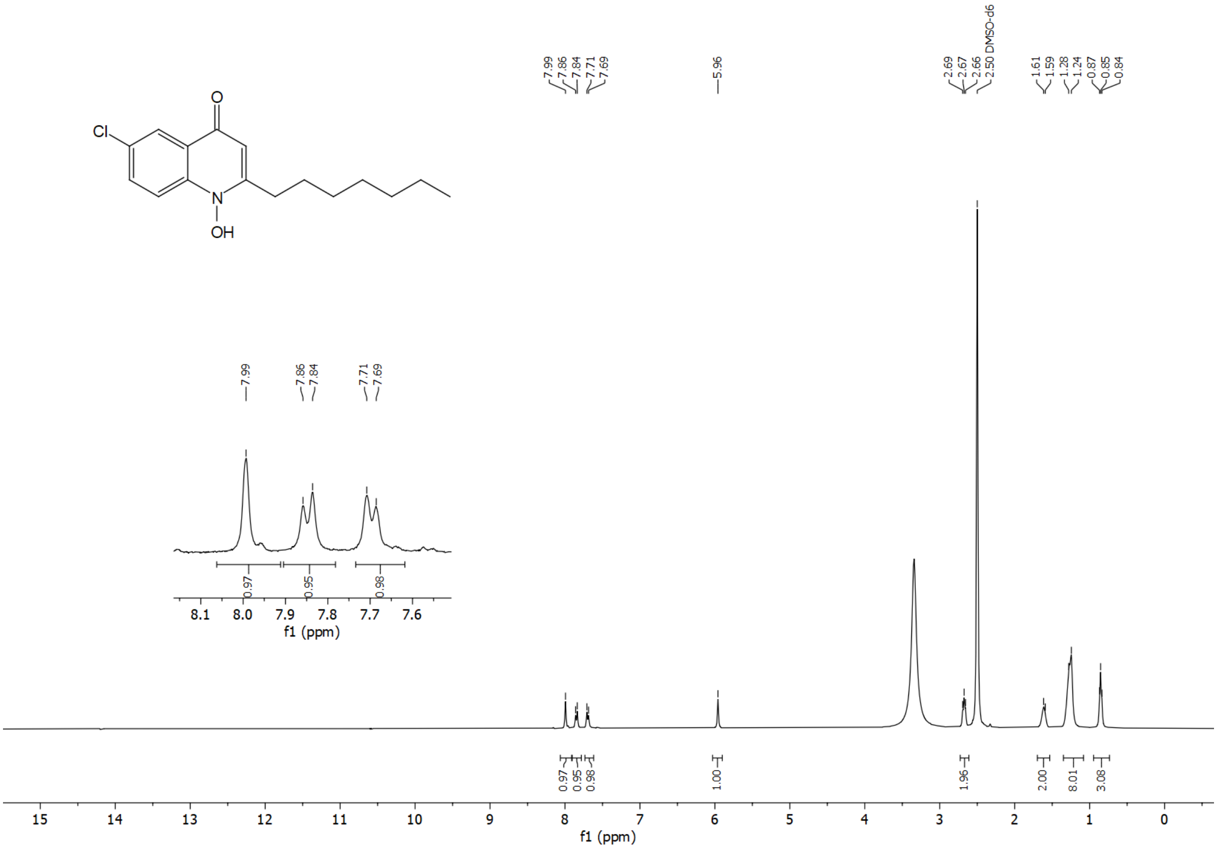

^{13}C NMR spectrum (101 MHz, DMSO-*d*$_6$) of **5aa**

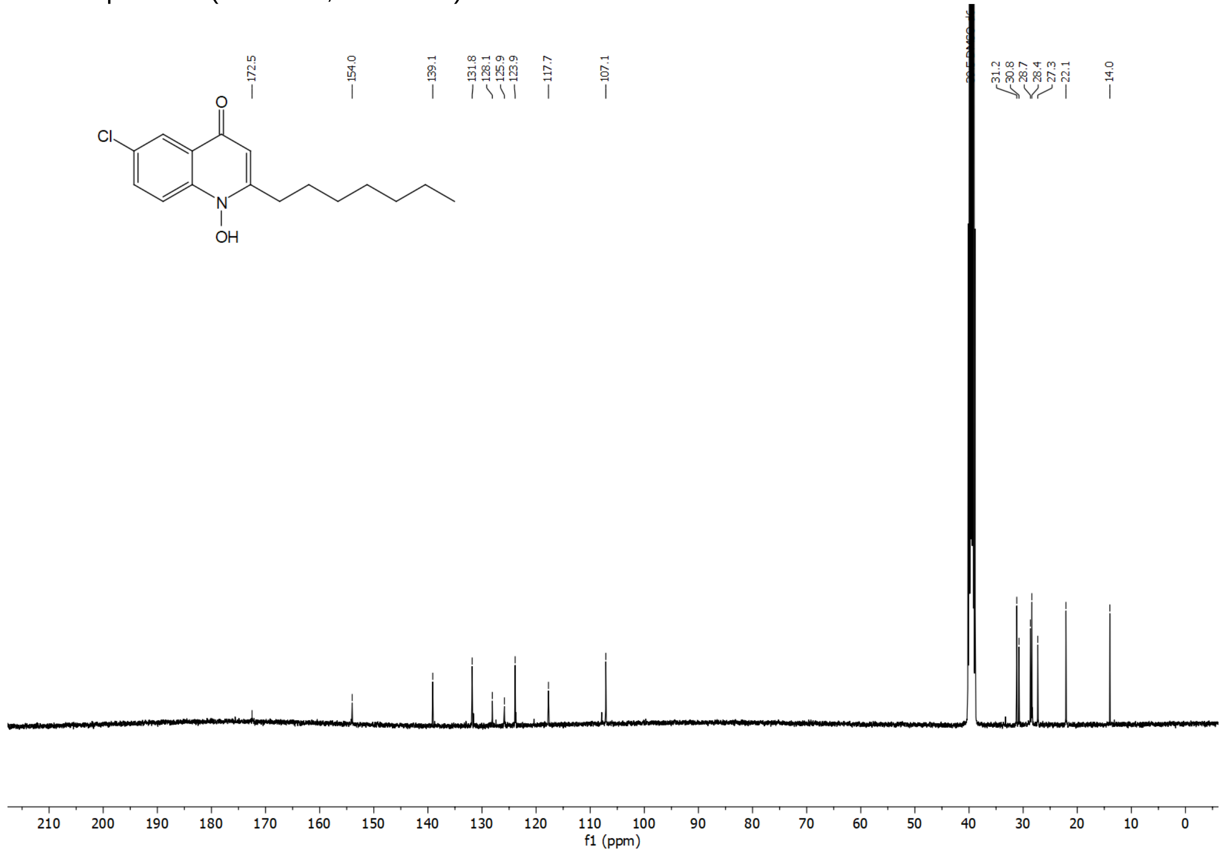

^{1}H NMR spectrum (400 MHz, DMSO-*d*$_6$) of **6a**

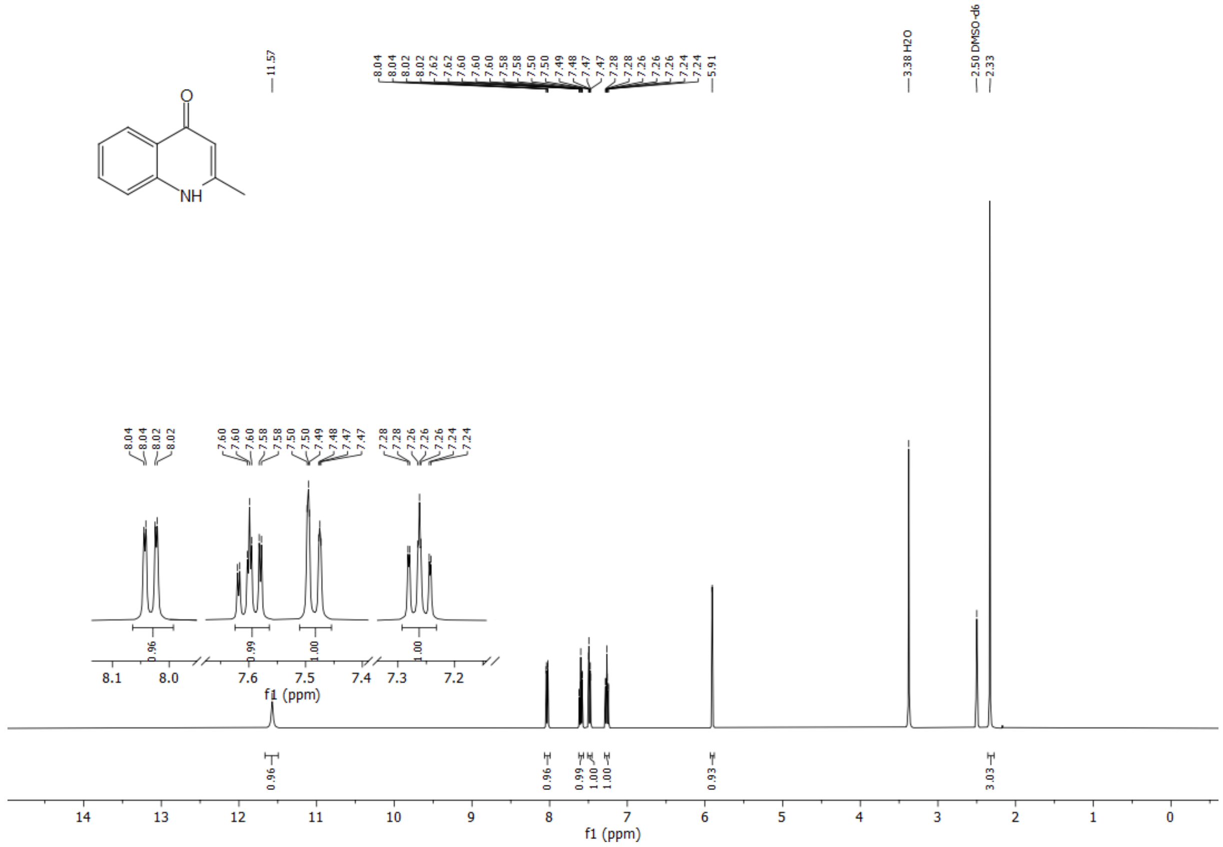

^{13}C NMR spectrum (101 MHz, DMSO-d_6) of **6a**

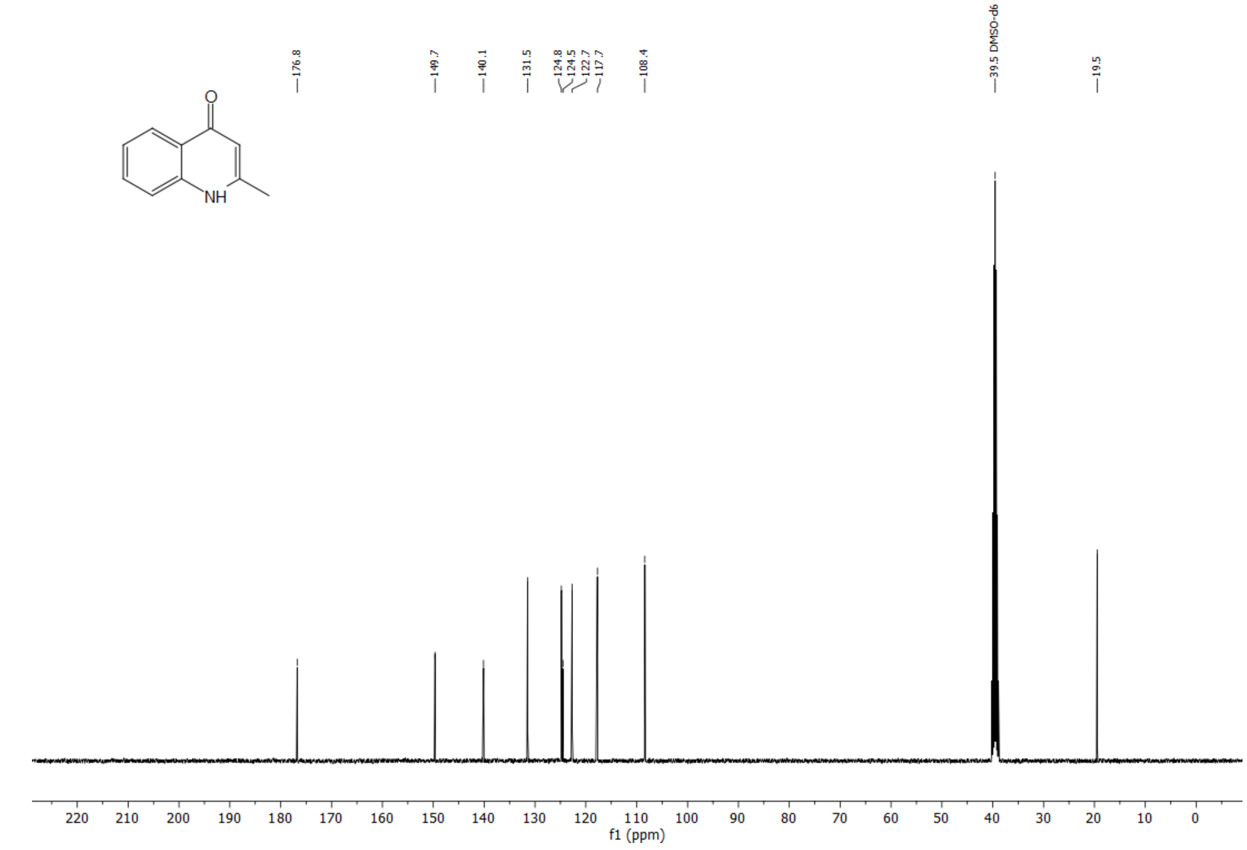

^{1}H NMR spectrum (400 MHz, $CDCl_3$) of **7a**

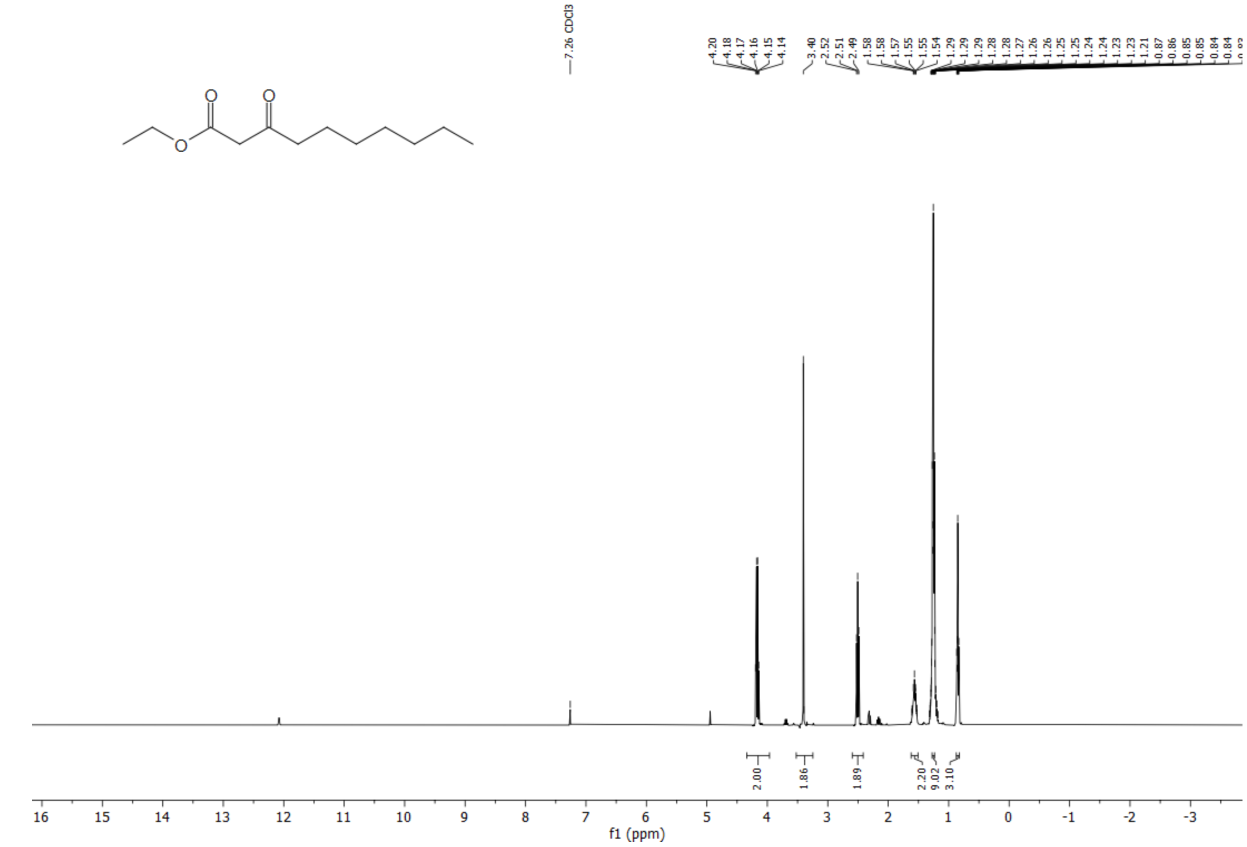

[13] C NMR spectrum (101 MHz, CDCl3) of **7a**

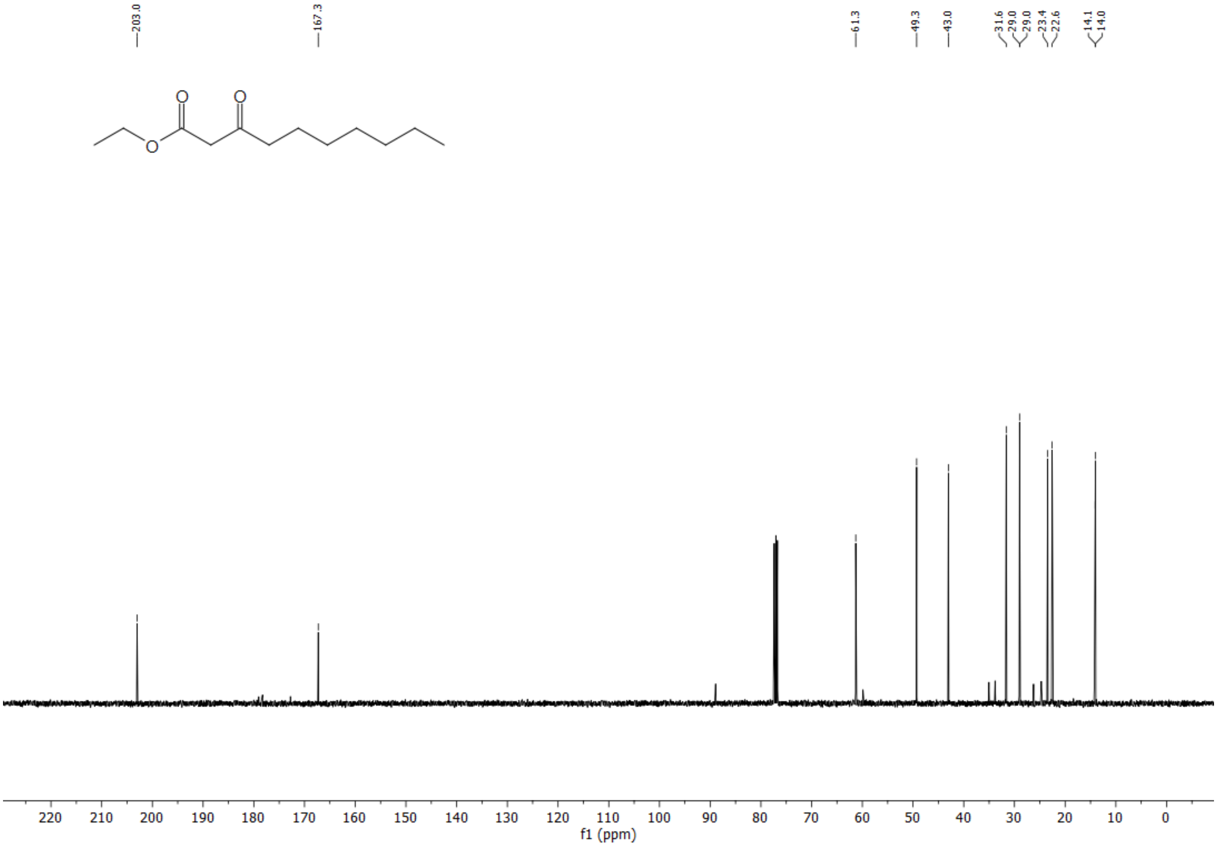

[1] H NMR spectrum (400 MHz, CDCl3) of **8a**

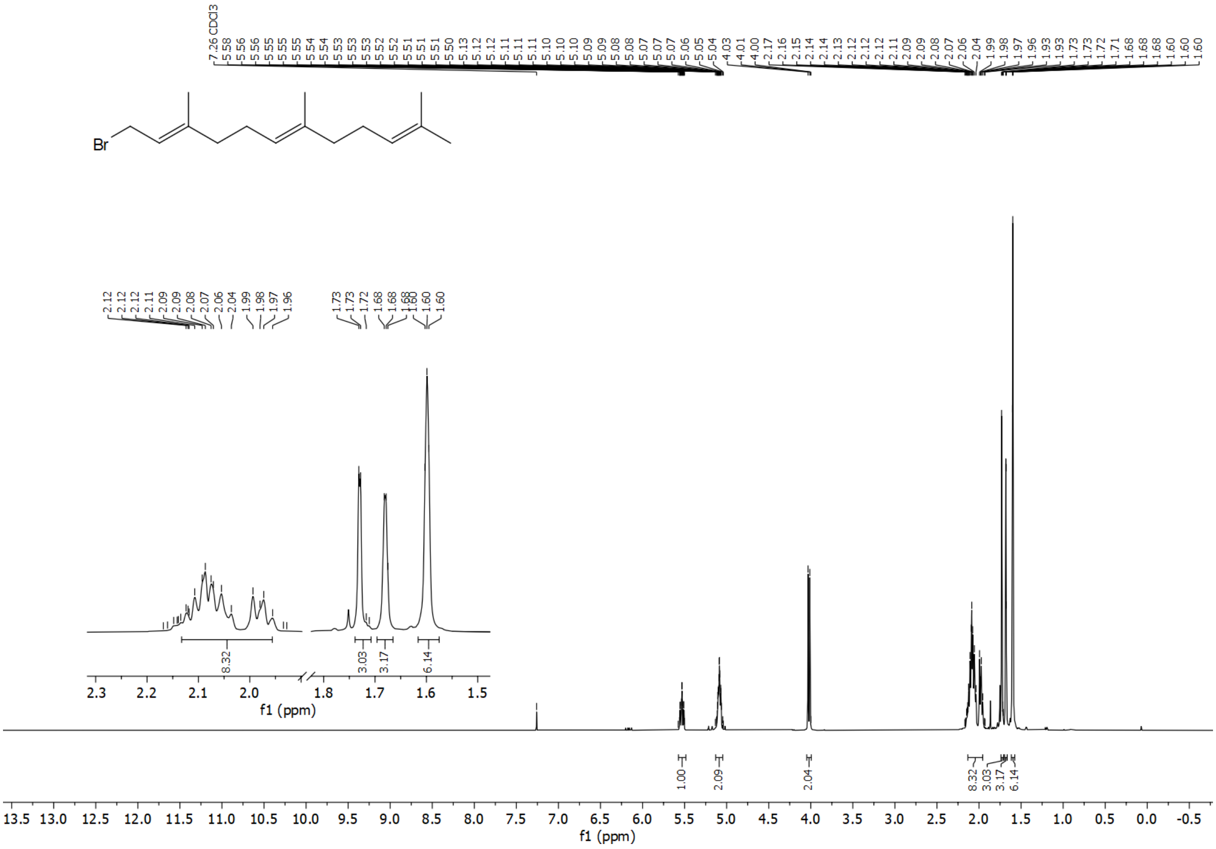

^{13}C NMR spectrum (101 MHz, $CDCl_3$) of **8a**

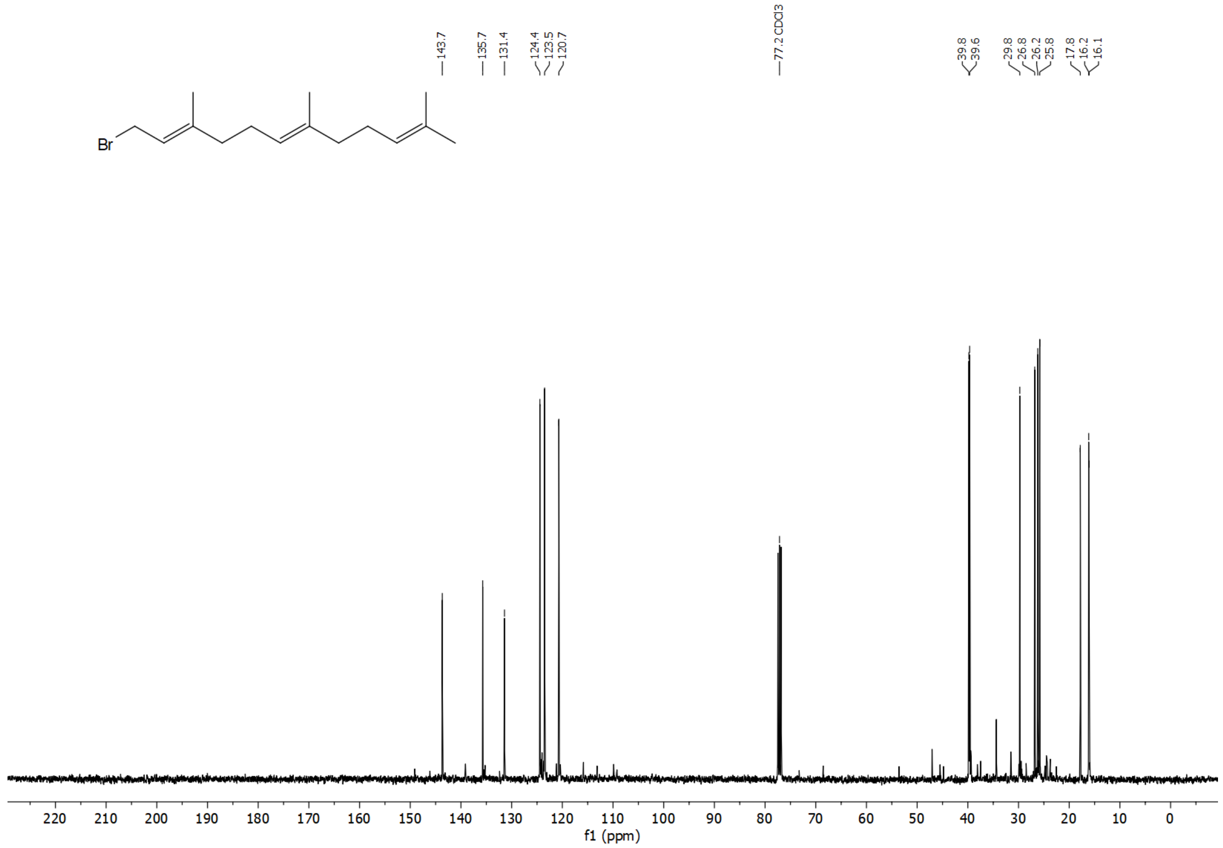

8. References

(1) Fulmer, G. R.; Miller, A. J. M.; Sherden, N. H.; Gottlieb, H. E.; Nudelman, A.; Stoltz, B. M.; Bercaw, J. E.; Goldberg, K. I. NMR Chemical Shifts of Trace Impurities: Common Laboratory Solvents, Organics, and Gases in Deuterated Solvents Relevant to the Organometallic Chemist. *Organometallics* **2010**, *29* (9), 2176–2179. DOI: 10.1021/om100106e.

(2) Gütz, C.; Klöckner, B.; Waldvogel, S. R. Electrochemical Screening for Electroorganic Synthesis. *Org. Process Res. Dev.* **2016**, *20* (1), 26–32. DOI: 10.1021/acs.oprd.5b00377.

(3) Pollok, D.; Gleede, B.; Stenglein, A.; Waldvogel, S. R. Preparative Batch-Type Electrosynthesis: A Tutorial. *Aldrichimica Acta* **2021**, *54*, 3–15.

(4) Riahi, A.; Shkoor, M.; Fatunsin, O.; Yawer, M. A.; Hussain, I.; Fischer, C.; Langer, P. Regioselective synthesis of amino- and nitroarenes based on [3+3] cyclocondensations of 1,3-bis(silyloxy)-1,3-butadienes. *Tetrahedron* **2009**, *65* (45), 9300–9315. DOI: 10.1016/j.tet.2009.09.014.

(5) Bhatt, A.; Gurukumar, K. R.; Basu, A.; Patel, M. R.; Kaushik-Basu, N.; Talele, T. T. Synthesis and SAR optimization of diketo acid pharmacophore for HCV NS5B polymerase inhibition. *Eur. J. Med. Chem.* **2011**, *46* (10), 5138–5145. DOI: 10.1016/j.ejmech.2011.08.028.

(6) Andrews, P. C.; Gee, W. J.; Junk, P. C.; MacLellan, J. G. Systematic study of the formation of the lanthanoid cubane cluster motif mediated by steric modification of diketonate ligands. *Dalton Trans.* **2011**, *40* (45), 12169–12179. DOI: 10.1039/C1DT10580A.

(7) Höfle, G.; Kunze, B. Biosynthesis of aurachins A-L in Stigmatella aurantiaca: a feeding study. *J. Nat. Prod.* **2008**, *71* (11), 1843–1849. DOI: 10.1021/np8003084.

(8) Li, J.; Sun, W.; Saalim, M.; Wei, G.; Zaleta-Pinet, D. A.; Clark, B. R. Isolation of 2-Alkyl-4-quinolones with Unusual Side Chains from a Chinese Pseudomonas aeruginosa Isolate. *J. Nat. Prod.* **2020**, *83* (7), 2294–2298. DOI: 10.1021/acs.jnatprod.0c00026.

(9) Walker, H. G.; Hauser, C. R. The Acetylation of o-, m- and p-Nitroacetophenones by the Boron Trifluoride Method 1. *J. Am. Chem. Soc.* **1946**, *68* (12), 2742–2743. DOI: 10.1021/ja01216a525.

(10) Wang, D.; Sun, P.; Jia, P.; Peng, J.; Yue, Y.; Chen, C. Transition-Metal-Free One-Pot Tandem Synthesis of 4-Quinolone and 4H-Thiochromen-4-one Derivatives Through Sequential Nucleophilic Addition–Elimination–SNAr Reaction. *Synthesis* **2017**, *49* (18), 4309–4320. DOI: 10.1055/s-0036-1588466.

(11) Rahaman, M.; Ali, M. S.; Jahan, K.; Hinz, D.; Belayet, J. B.; Majinski, R.; Hossain, M. M. Synthetic Scope of Brønsted Acid-Catalyzed Reactions of Carbonyl Compounds and Ethyl Diazoacetate. *J. Org. Chem.* **2021**, *86* (9), 6138–6147. DOI: 10.1021/acs.joc.0c02972.

(12) Reichl, K. D.; Dunn, N. L.; Fastuca, N. J.; Radosevich, A. T. Biphilic Organophosphorus Catalysis: Regioselective Reductive Transposition of Allylic Bromides via P(III)/P(V) Redox Cycling. *J. Am. Chem. Soc.* **2015**, *137* (16), 5292–5295. DOI: 10.1021/jacs.5b01899.
